# Growth following adversity is rare: Evidence from a multi-informant longitudinal study of children and adolescents

**DOI:** 10.1016/j.jrp.2025.104628

**Published:** 2025-06-18

**Authors:** Cavan V. Bonner, Benjamin L. Hankin, Jami F. Young, Brent W. Roberts

**Affiliations:** a University of Illinois at Urbana-Champaign, United States; b Department of Child and Adolescent Psychiatry and Behavioral Sciences, Children’s Hospital of Philadelphia, United States; c Department of Psychiatry, University of Pennsylvania Perelman School of Medicine, United States

**Keywords:** Effortful control, Self-regulation, Negative emotionality, Adversity, Personality development, Adolescent development, Temperament development, Post-traumatic growth, Post-adversity growth, Latent growth curve model

## Abstract

The idea that adversity is necessary for psychological growth pervades cultural narratives and lay theories. We empirically tested this notion with a multi-informant, longitudinal study of children and adolescents (*n* = 682). Initial adversity was not associated with change in effortful control and emotional stability, while increasing adversity was negatively correlated with growth. However, a small sub-group of individuals still managed to grow despite adversity. The narrative that adversity is crucial for growth likely originated, and continues to survive, because scholars and laypeople focus on this minority who grow despite adversity, while overlooking the overall null or negative association. The accumulated evidence suggests that researchers should look elsewhere for the life experiences that reliably lead to growth and not distress.

How do children and adolescents become more self-regulated and emotionally stable? A common lay belief is that young people need to face and successfully overcome adversity in order to achieve psychosocial maturity. Public intellectuals have warned that coddling children and young adults from adversity will stunt their maturation (Lukianoff & Haidt, 2019), and have argued that adversity is crucial to the development of character (Brooks, 2015). Similar arguments have been made in psychological science about the ubiquitous benefits of adversity for psychological growth (Linley & Joseph, 2004; Tedeschi & Calhoun, 2004).

Initial support for the notion of post-adversity growth came from cross-sectional surveys of adults who had previously experienced adversity. Many of these samples reported remarkably high rates of retrospectively *perceived* post-adversity growth (i.e., exceeding 50 %) in at least one domain (see Jayawickreme & Blackie, 2014; Linley & Joseph, 2004 for reviews). However, when the assessment of retrospective, perceived growth was directly compared to growth assessed with prospective, longitudinal methods, they turned out to be unrelated and distinct phenomena (Frazier et al., 2009).

Recent research that assessed psychological functioning before and after adversity with one-to-two-year longitudinal studies suggests that there is little evidence for positive average effects of adversity on psychological growth in domains such as life satisfaction, generativity, meaning, gratitude, compassion, or spirituality (Infurna et al., 2022; Mangelsdorf et al., 2019), five-factor personality traits (Blackie & Hudson, 2023), wisdom (Dorfman et al., 2022), and character strengths (Gander & Wagner, 2022). In fact, adversity is typically linked to negative changes in psychological functioning. Increases in adversity are associated with decreases in effortful control throughout adolescence (Serrano et al., 2022). Adversity predicted decreases in effortful control and emotional stability during adolescence (Laceulle et al., 2012), and decreases in agreeableness, conscientiousness, and emotional stability in adulthood (Löckenhoff et al., 2009; Shiner et al., 2017).

While the main effect of adversity is typically null or negative, this does not preclude the empirical existence of growth following adversity. It is possible that a minority do “grow” following adversity even if the average effect of adversity on growth is near zero. In fact, by definition, this is the most likely scenario if the average effect of adversity is zero, because approximately equal numbers of people would be increasing and decreasing following the experience of adversity. The widespread cultural belief that adversity results in growth may come from the fact that a measurable minority of the people who experience adversity still grow in the aftermath of the experience. For this to be true, we should find a sizeable group of people growing after the experience of adversity.

To our knowledge, only three prospective, longitudinal studies have gone beyond average effects to directly examine how many people experience post-adversity psychological growth (i.e., Frazier et al., 2009; Fraley et al, 2021; Infurna et al., 2022). Infurna et al. found average decreases or null effects following adversity. However, post-adversity growth was rare but observable: 11 % of the sample were classified as experiencing enduring post-adversity growth. In the second study, changes in avoidant and anxious attachment following negative life events were studied over a 2-year period (Fraley et al., 2021). Improvement in attachment avoidance or anxiety were observed for a minority (6–19 %) who experienced aversive life events such as job loss, illness, and a family member passing away. Finally, Frazier et al. examined change over the course of two months in college students who experienced a traumatic event between the assessments. The prevalence of reliable increases ranged between 5–25 % across domains such as relationship quality, meaning in life, life satisfaction, gratitude, and spirituality.

Though these three studies provide informative estimates of the prevalence of growth following adversity, they focused on adult samples and excluded measures of self-regulation and emotional stability in favor of constructs core to the Post Traumatic Growth literature, such as spirituality and gratitude. Scientific and lay perspectives (Brooks, 2015; B. J. Ellis et al., 2022; Liu, 2015; Lukianoff & Haidt, 2019; Oshri, 2023; Rutter, 2006) on growth following adversity have frequently focused on children and adolescents, but we still lack empirical data on the prevalence of growth following adversity from prospective longitudinal studies of these populations. Therefore, the present research aims to extend recent discussions about the prevalence of growth following adversity to child and adolescent populations.

Moreover, it is critical to test the effect of adversity on constructs associated with adaptive functioning and lay theories of post-adversity character development, such as effortful control and emotional stability (Snyder et al., 2015). Finally, the three prior studies and most research examining adversity have relied almost exclusively on self-reports of adversity and growth, which may bias the estimates of growth following adversity due to common method biases (Podsakoff et al., 2003).

In the present research, we investigated the prevalence of post-adversity growth in effortful control and emotional stability with a large, non-clinical community sample of children and adolescents who were enrolled in the 3-year Genes, Environment, and Mood (GEM) study (Hankin et al., 2015). Unlike prior studies, the GEM study has observer-based ratings of adversity and parental reports on effortful control and emotional stability. We tested four sequential, pre-registered research questions (https://osf.io/3g9rd/?view_only=109fc43818014bf597bd7cca0704fd49). (1) How do effortful control and emotional stability change over time? (2) Is adversity correlated with changes in effortful control and emotional stability? (3) What proportion of youth exhibited meaningful increases in effortful control and emotional stability following exposure to adversity? And (4) what other factors — such as attachment security, parenting practices, and peer support — predict growth following adversity? Because this research is exploratory, we did not have *a priori* hypotheses about these research questions.

## 1. Method

### 1.1. Participants and procedure

The data come from the Genes, Environment, and Mood (GEM) sample (see Hankin et al., 2015 for full demographic information). The GEM sample is comprised of children and adolescents who were recruited through letters sent to the homes of families by participating school districts at two sites (Denver metropolitan area and central New Jersey). Cohorts of youth in the third (*n* = 209), sixth (*n* = 249), and ninth grades (*n* = 224) were enrolled and completed three in-person laboratory assessments with their caretaker at baseline, 18 months, and 36 months. A total of 682 cases provided usable data at the first wave; we used full-information maximum-likelihood (FIML) estimation to handle the missing data at later waves.

### 1.2. Measures

All study variables, with the exception of parenting practices and peer support, were assessed at each wave. The latter were only assessed at the second and third wave. Reliability, descriptive statistics, and intercorrelations among the main study variables are presented in Table S1a; descriptive statistics and test–retest stability coefficients for facet variables from supplementary analyses are presented in Table S1b. Materials for some measures are detailed in Supplement C (osf.io/cpdsq); the EATQ-R and YLSI are not currently in the public domain but are available upon request.

#### 1.2.1. Adolescent effortful control and emotional stability

Effortful control and emotional stability were assessed with the Early Adolescent Temperament Questionnaire-Revised (EATQ-R; Ellis & Rothbart, 2001) and the Parent Report Form version of the EATQ-R. Parents and youth were asked to rate the extent to which each item characterized the behavior of the child on a 1–5 Likert scale (1 = “Almost always untrue”; 5 = “Almost always true”). We chose to not aggregate the youth and parent-report data together, and instead treated them as distinct variables in the analyses.

Effortful control was scored as the sum of three facet scale scores — activation control, attention, and inhibitory control — which is in line with the original scoring instructions recommended by the creators of the EATQ-R (Ellis & Rothbart, 2001). Though alternative scoring procedures for the EATQ-R effortful control and emotional stability constructs have recently been suggested (e.g., Latham et al., 2020), we chose to adhere to the original instructions to be consistent with the existing literature (Synder et al., 2015). Example items for effortful control include “If I have a hard assignment to do, I get started right away” (for activation control), “It is easy for me to really concentrate on homework problems” (for attention), and “I can stick with my plans and goals” (for inhibitory control).

Negative emotionality was also scored as the sum of four facet scale scores — fear, frustration, shyness, and aggression — according to the original scoring instructions recommended by the creators of the EATQ-R. We then reverse-scored negative emotionality and its facets so that increases could be interpreted as desirable “growth,” and we refer to it as “emotional stability” (“ES”) throughout the rest of the manuscript. Example items (all reverse coded) for emotional stability include “I worry about my family when I’m not with them” (for fear), “I get very upset if I want to do something and my parents won’t let me” (for frustration), “I am shy” (for shyness), and “When I am angry, I throw or break things” (for aggression).

#### 1.2.2. Adversity

Exposure to adversity was assessed at each wave with the Youth Life Stress Interview (YLSI; Rudolph & Flynn, 2007). The YLSI assessed the severity of stressful events in sixteen life domains, such as academics, peer relationships, caregiver relationship, body image, health, and exposure to violence. A team of three or more raters, who were blind to the identity of the participant, came to consensus on an appropriate severity score for each stress domain. Each domain score ranged between 1 and 5 (1 = “little/no stress”; 2 = “average/normal stress”; 3 = “moderate stress”; 4 = “serious stress”; 5 = “severe stress”). Because we were interested in the cumulative amount of adversity that the youth had been exposed to, we summed the domain scores into a cumulative index of adversity exposure and averaged the sum so that the scores would be scaled from 1 to 5. For the purpose of subsequent analyses, scores greater than 2 were considered indicators of substantial adversity that was beyond “average/normal stress”.

#### 1.2.3. Attachment security to caregiver

Youth-reported attachment anxiety and attachment avoidance to the caregiver who accompanied the child to the study visits was assessed at each wave with the Experiences in Close Relationships-Relationship Structures instrument (Fraley et al., 2011).

#### 1.2.4. Peer support

We used the two items from the prosocial behavior subscale of the Revised Peer Experiences Questionnaire (De Los Reyes & Prinstein, 2004) to measure the receipt of prosocial behavior from peers (i.e., peer support). The items were “Another kid helped me when I was having a problem” and “Another kid stuck up for me when was being picked on or excluded.” Both items were rated on a 1–5 frequency scale (1 = “Never”; 5 = “A few times a week”).

#### 1.2.5. Youth-perceived parenting style

We assessed the quality of youth-perceived parenting style with ten items from the Parenting Styles Scale (Lamborn et al., 1991). The items were originally referred to as measures of a unidimensional “Acceptance/Involvement” construct; for brevity we refer to this as youth-perceived parenting style throughout the rest of the manuscript. Because half of the items were binary, true–false questions, while the other half were polytomous, we re-scaled the polytomous item responses to be binary (the scoring is detailed in Supplement C). Example items include “I can count on him/her to help me out if I have some kind of problem” and “When he/she wants me to do something, he/she explains why.”

### 1.3. Attrition

517 youth provided complete data on effortful control and emotional stability at the final wave; approximately 24 % of the initial sample was missing from the final assessment. Youth who had dropped out before the final wave mostly differed from those who stayed in terms of socioeconomic status indicators: they were more likely to come from a family that received food stamps (*z* = 3.26, *p* = 0.001, *h* = 0.27), receive free or reduced school lunch (*z* = 4.98, *p* < 0.001, *h* = 0.42), and have parents with lower educational attainment (*t* = − 4.47, *p* < 0.001, *d* = − 0.42). Youth who dropped out were also more likely to identify with a non-white ethnicity (*z* = 3.82, *p* < 0.001, *h* = 0.32) or race (*z* = 3.78, *p* < 0.001, *h* = 0.33). There were no significant differences on effortful control, emotional stability, or the attachment security variables (see Table S2).

### 1.4. Open practices

Research questions and analyses were pre-registered prior to the first and last author accessing and analyzing the data (https://osf.io/3g9rd/?view_only=109fc43818014bf597bd7cca0704fd49). We note all deviations from the pre-registered analysis plan in Table S1 of Supplement A following the template provided by Willroth and Atherton (2024; osf.io/283x6). All analyses were conducted in R (R Core Team, 2024) with *psych* (Revelle, 2024), *corrr* (Kuhn et al., 2022), *rempsyc* (Thériault, 2023), *flextable* (Gohel et al., 2024), and *lavaan* (Rosseel, 2012) packages. We cannot publicly share the data because the IRBs did not approve — and the participants did not consent to — sharing the data on a publicly accessible repository. Scholars who are interested in reproducing the results can contact Benjamin L. Hankin.

### 1.5. Analytic strategy

Our analyses primarily relied on univariate and multi-variate latent growth curve (LGC) models to understand the average trajectories and individual differences in effortful control and emotional stability development. We focus on the results for effortful control and emotional stability in the main analyses; however, for research questions 1–3 we also re-ran the analyses for the effortful control and emotional stability facets and report these results in the supplemental materials. We detail the analytic decisions made to address each research question below.

#### 1.5.1. Research question 1

Within the LGC framework, the average slope indicates whether the sample increases or decreases on average over time. These estimates were used to answer our first research question pertaining to whether the sample increased or decreased during the time of the study.

We examined patterns of mean-level change and individual differences in the development of effortful control, emotional stability, and exposure to adversity in childhood and early adolescence with a series of LGC models that compared no growth, unconditional growth, and growth conditioned on grade cohort. We only report the results for the conditional growth models for effortful control and emotional stability in the main text; results for the univariate no growth, unconditional, and facet models are reported in Tables S3 and S6. We focus on the models conditioned on grade cohort because they account for the data’s accelerated cohort structure, and allows us to compare the prevalence of growth between levels of adversity exposure after controlling for initial grade cohort.^1^ Cohort was retained as a covariate in all subsequent bivariate and trivariate models. Time was scaled as 0, 0.5, 1 for the slope factor estimation in order for the coefficient to represent total predicted change, which generated interpretable slope factor scores for the 3rd research question.

#### 1.5.2. Research question 2

We answered our second question by examining the correlations between the effortful control and emotional stability slopes and the experience of adversity in bivariate LGC models. We examined the association between the initial adversity intercept, as well as the association between change in adversity (also estimated as a slope in the bivariate LGC model) with the slopes of effortful control and emotional stability.

#### 1.5.3. Research question 3

Our third research questions was what proportion of youth exhibited meaningful increases in effortful control and emotional stability following exposure to adversity. In order estimate how many youth increased or decreased in effortful control and emotional stability over the course of the study after experiencing adversity, we extracted factor scores from the slope factors of univariate LGC models for effortful control and emotional stability.^2^ In these models, the intercept and slope of effortful control and emotional stability were regressed onto exogenous adversity intercept and slope variables.^3^ The factor scores from the models where the slopes of effortful control and emotional stability were regressed on the exogenous adversity intercept and slope therefore represented the predicted change given an individual’s exposure to initial adversity and change in adversity over the course of the study.

We categorized youth with effortful control and emotional stability slope factor scores that were >/= 1/5th of a standard deviation of the distribution of manifest scores for that construct at the baseline assessment as increasing or decreasing. We specified 1/5th of the SD as the pre-registered “smallest effect size of interest” because that effect size is approximately equivalent to an effect size of *r* = 0.10, which has been suggested as a cutoff for the substantive interpretation of effect sizes based on the average size of spurious but statistically significant correlations that occur in large datasets (Ferguson & Heene, 2021). We derived the construct-specific effect size cut-off from the distribution of that construct’s baseline raw scores because this yielded more conservative cut-offs compared to the distribution of intercepts in the LGC models.

We partitioned the sample into a group who had not experienced initial or increasing adversity, and groups who had experienced initial or increasing adversity. To create the initial adversity group, we selected youth whose baseline adversity scores were greater than 2. Because “2″ scores on the scale were indicators of “average/normal stress,” we interpreted scores greater than 2 as an indication that the child or adolescent had been exposed to a non-normative and consequential amount of adversity. To create the increasing adversity group, we selected youth whose adversity slope factor scores were *>*/= 1/5th of a standard deviation from the baseline distribution of adversity scores.

#### 1.5.4. Research question 4

Finally, we answered our fourth research question by correlating slopes in the LGC model with alternative factors, such as as changes attachment security, youth perceptions of parenting style, and peer support. Though our pre-registered analyses called for conducting these analyses with trivariate LGC models, this ended up only being feasible for the attachment security measures. The other variables were only measured at two waves each, so we estimated latent change score models and used the change scores for further analyses. We first specified univariate LGC models or latent change score models for these constructs (attachment security results are reported in Table S6; parenting style and peer support are reported in Table S11). We then estimated a series of associative, trivariate variable models between the intercepts and slopes of effortful control or emotional stability, each predictor, and adversity (Duncan et al., 2000; Table S12). Additionally, we estimated a series of regression models where the slopes of the parent-reported effortful control or emotional stability were regressed onto the intercepts and slopes of adversity and attachment anxiety, attachment avoidance, peer support, and parenting style. This controls for the effect of adversity on effortful control and emotional stability development and allowed us to test the contribution of additional developmental processes to the development of effortful control and emotional stability above and beyond adversity. The coefficients for the slope of adversity and the additional predictor are reported in Table 5 (full results for the models are reported in Table S12).

#### 1.5.5. Model fit results

The fit for the youth and parent-reported univariate effortful control models were acceptable according to the CFI (Table 1; full results in Table S3), but was not good for the TLI and RMSEA according to conventional criteria (Hu & Bentler, 1999). The fit for the parent-reported emotional stability model followed a similar pattern; however, the fit for the youth-reported emotional stability (and fear) was especially bad according to all indices.^4^ Fit indices for the bivariate models conditioned on cohort are reported in Table 2.

## 2. Results

Raw means, standard deviations, and correlations among the manifest variables are presented in Table S1a; additional manifest correlations for the facet measures are available in Table S1b osf.io/ma2yj.

### 2.1. Research question 1: How do effortful control and emotional stability change over time?

We addressed our first research question by examining the fixed effects for the slopes of self- and informant-reported effortful control and emotional stability. Table 3 reports the fixed-effects for the unstandardized slopes from LGC models for changes in youth and parent-reported effortful control and emotional stability, as well as the standardized mean differences (the single-group pretest–posttest raw score effect size; Morris & DeShon, 2002) between the start and end of the study. Standardized mean differences for all facets are reported in Table S5, and full parameter estimates from all of the univariate models are reported in Table S6.

Effortful control increased in both youth-reported (*b* = 0.12, *p* = 0.002) and parent-reported (*b* = 0.14, *p* < 0.001) models. Emotional stability increased in both youth- reported (*b* = 0.37, *p* < 0.001) and parent-reported (*b* = 0.23, *p* < 0.001) models.

### 2.2. Research question 2: Is adversity correlated with changes in effortful control and emotional stability?

We next tested whether the experience of adversity was associated with changes in effortful control and emotional stability using the bivariate LGC models. As noted above, there was significant variance around the mean slope parameter, indicating that examining the association of individual differences in slopes was warranted. Initial levels of adversity were not associated with change in effortful control (youthreport: *r* = 0.04, *p* = 0.542; parent-report: *r* = 0.01, *p* = 0.834) or emotional stability (youth-report: *r* = 0.04, *p* = 0.570; parent-report: *r* = − 0.07, *p* = 0.194). Increases in adversity were negatively associated with increases in both youth-reported effortful control (*r* = − 0.50, *p* < 0.001) and parent-reported effortful control (*r* = − 0.31, *p* = 0.003). Increases in adversity were negatively associated with increases in youth-reported emotional stability (*r* = − 0.54, *p* < 0.001), while the association was smaller and non-significant for parent-reported emotional stability (*r* = − 0.18, *p* = 0.064). Results for the facet-level models largely mirrored those found in the domains and are reported in Table S8b.

### 2.3. Research question 3: What proportion of youth exhibited meaningful increases in effortful control and emotional stability following exposure to adversity?

Table 4 reports the proportions of participants who increased and decreased in effortful control and emotional stability given the two forms of adversity exposure. About half of the youth who were not exposed to initial or increasing adversity increased in youth-reported (48 %) and parent-reported (49 %) effortful control. We supplemented our pre-registered analyses with two-proportion z-tests to formally compare whether the proportions were different between groups. Among those who experienced substantial adversity at the baseline assessment, the proportion who increased in youth-reported (40 %) and parent-reported (47 %) effortful control were not significantly different from the low-adversity group (*χ*^2^(1) = 2.30, *p* = 0.128; *χ*^2^(1) = 0.08, *p* = 0.768 for youth and parent-report respectively). Among those who experienced increasing adversity over the course of the study, the proportion who increased in youth-reported effortful control (19 %) was significantly smaller (*χ*^2^(1) = 19.37, *p* < 0.001) relative to the no-adversity group, while the proportion for parent-report (38 %) was not significantly different (*χ*^2^(1) = 2.64, *p* = 0.103).

The majority of youth who were not exposed to initial or increasing adversity increased in youth-report (81 %) and parent-reported (72 %) emotional stability. Among those who experienced substantial adversity at the baseline assessment, the proportion who increased in youth-reported (76 %) and parent-reported (66 %) emotional stability were not significantly different from the no-adversity group (*χ*^2^(1) = 1.25, *p* = 0.263; *χ*^2^(1) = 1.73, *p* = 0.187). Among those who experienced increasing adversity over the course of the study, the proportion who increased in youth-reported (49 %) and parent-reported (54 %) emotional stability were both significantly smaller than the low-adversity group (*χ*^2^(1) = 33.25, *p* < 0.001; *χ*^2^(1) = 9.00, *p* = 0.002).

These results provide evidence that the base rate of growth after initial adversity is not significantly different from those who experienced low amounts of adversity over the course of the study. The proportion of youth who grew despite increasing adversity over the course of the study was typically lower but was still sizable. However, it is important to note that because of the low prevalence of high adversity in this sample, all of the adversity-exposed youth made up just 17 % of everyone who increased in youth-reported effortful control (21 % for parent-reported), and 20 % of everyone increased youth-reported emotional stability (21 % for parent-reported).

In exploratory (i.e., not pre-registered) follow-up analyses, we also examined demographic differences between the group who grew despite adversity, and the groups who did not grow following adversity, or grew in the absence of adversity (see Tables S23a–d and S24a–d for details). Youth who grew in effortful control and emotional stability despite adversity did not display robust demographic differences from youth who did not grow after being similarly exposed to adversity. However, youth who grew despite adversity were reliably different from youth who grew in the absence of adversity on several demographic variables: they had significantly less family income (*d*s ranged from 0.27–0.45), their parents tended to have less education (*d*s ranged from 0.28–0.39), and they were also less likely to come from a family where the parents reported being married at baseline (Cohen’s *h*s ranged from 0.24–0.42). The groups who grew in parent and youth-reported emotional stability despite adversity were also more likely to be from families receiving food stamps (*h*s ranged from 0.34–0.41). Finally, the youth who grew despite adversity tended to be about a year older, and in a higher grade at baseline, compared to youth who grew without adversity.

### 2.4. Research question 4: What other factors predict growth in effortful control and emotional stability following adversity?

Decreasing in the attachment dimensions of anxiety and avoidance (i.e., becoming more securely attached) incrementally predicted increases in parent-reported effortful control, while only decreases in attachment avoidance predicted increases in parent-reported emotional stability (see Table 5). Change in parenting style and peer support did not significantly predict growth in parent-reported effortful control or emotional stability.

## 3. Discussion

It is a common cultural narrative that adversity leads to resiliency and growth. Our aim was to examine this relation more closely and possibly identify where the narrative might have emerged from, given the nature of growth, adversity, and their interplay. Our first question was basic, but also necessary for the idea to exist — do children and adolescents grow in characteristics associated with resiliency and character, such as emotional stability and effortful control? This basic question is often overlooked in the rush to test whether experiences impart change in personality, which is problematic because in the absence of measurable change the follow-up questions would make little sense. The sample showed signs of mean-level improvement on both emotional stability and effortful control, and a sizable subgroup showed evidence for growth in these qualities. These findings supported moving to the next set of research questions.

Our second research question focused on the relation between adversity and growth in emotional stability and effortful control. Like many recent studies, we found no relationship between initial adversity and subsequent growth as well as a robust negative relationship between change in adversity and change in both effortful control and emotional stability (e.g., Infurna et al., 2022; Laceulle et al., 2012; Serrano et al., 2022). When combined with prior research, our findings help to clarify the relationship between adversity and growth. The idea that adversity leads to growth has little or no empirical basis. Study after study has now shown either null or negative relationships between adversity and a host of qualities associated with resiliency and character.

Given the null or negative relation between adversity and growth in qualities like emotional stability and effortful control, how did the cultural narrative that adversity leads to growth originate, and how does it continue to survive despite mounting empirical evidence to the contrary? One simple and previously unexamined possibility is selection effects. The idea that adversity can lead to growth can emerge if laypeople and scholars alike focus on people who happened to grow *despite* experiencing adversity. In many ways, this is a statistical necessity. In the case of adversity having no relation to growth, by definition an equal number of people will increase and decrease if there are meaningful changes in emotional stability and effortful control, which there were in our data. Even in the case of the negative association between increasing adversity and decreasing effortful control and emotional stability, the less than perfect relation means there are still a small minority of individuals who do not get worse and thus appear “resilient” to the effects of increasing adversity. Thus, theories that emphasize the importance of suffering for growth and maturation are grounded in a statistical reality, but one that ignores the overall null or negative relation between adversity and growth. People do not grow because of adversity, but despite it.

Popular science and lay discussions often frame the developmental importance of suffering and adversity for maturation as an essential precondition for success, but in the context of the entire study population, growth after initial adversity was a relatively rare sequence of events that does not describe the typical experience of development. Most (79–83 %) of the participants who increased in effortful control and emotional stability over the course of development did so in the absence of adversity. When scholars and laypeople speak of the benefits of suffering for the development of character, the language is often imprecise and vague. We suspect that the the forms of severe adversity and stress that were assessed in the present research are not typically what they have in mind. However, we believe that is it plausible that stressful and challenging life experiences may commonly lead to growth and maturation when they possess certain characteristics not shared by the experiences assessed in the present research, such as being able to self-select into and opt-out of by choice, being anticipated and desired, and being controllable and manageable. Scholars have referred to these challenging-yet-manageable life experiences as the “sweet spot” (Bloom, 2021) or as “desirable difficulties” (Bjork & Bjork, 2011). Advancing the assessment and understanding of these forms of desirable, self-imposed challenges may yield empirical evidence for processes and outcomes that better reflect lay intuitions about the connection between suffering and growth (Bonner & Roberts, 2023).

As a counterfactual test of the argument that growth necessitates adversity, we explored the relationship between experiences that had the potential to improve the lives of children and adolescents, such as secure attachment to parents, positive parenting styles, and peer support. Among these factors, only improving attachment security was correlated with increases in emotional stability and effortful control. We provided these ancillary tests to begin addressing the obvious follow up question: if adversity does not lead to growth, then what does? It appears that increasing attachment security to a parent might be one of those experiences.

### 3.1. Strengths, limitations, and future directions

The present findings are unique as they are based on multi-informant data from a diverse, non-clinical community sample of children and adolescents. Despite these strengths, a limitation was the relatively low base rate of adversity. The limited occurrence of adversity prohibited us from more fine-grained analyses among different age and demographic sub-groups in the sample. Future research should extend these findings with larger samples that permit investigating the developmental timing of growth following adversity.

The assessment of adversity also utilized a gold-standard clinical assessment based on objective raters of interviews (Rudolph & Flynn, 2007), combined with self- and parent-report assessments of effortful control and emotional stability. While this improves on prior work that relied upon mono-method self-reports of adversity and provides estimates that overcome common method biases (Podsakoff et al., 2003), it is possible that subjective perceptions of adversity may show a different pattern (Dugan et al., 2023; Schwaba et al., 2023), which future research should directly compare. Two of the predictors of growth despite adversity were not pre-registered and didn’t cover the full duration of the study; future research should test additional *a priori*, theoretically informed predictors. Finally, the apparent initial elevation bias revealed by the accelerated cohort data structure also means that the results for the youth/parent-reported emotional stability and youth-reported effortful control should be interpreted cautiously.

#### 3.1.1. Limitations on generality

These results would likely generalize to samples of children and adolescents that have similar demographic and cultural characteristics in the context United States, because the sample was broadly representative of the diversity of the United States (though Hispanic participants were under-represented; see Hankin et al., 2015). We would also expect that many of these results would generalize to other western, highly developed industrialized countries, but are substantially less confident about their generalizability to patterns of lifespan development in other socio-cultural contexts.

## 4. Conclusion

The results of the present research demonstrate that growth following adversity is an empirically observable phenomena, but it characterizes a relatively small group. It is misleading to claim, based on the accumulated evidence, that these individuals grew *because* of adversity — rather, it is more plausible that they grew *despite* it. Researchers should endeavor to use more precise and careful language when discussing the role of adversity in development. While the experience of those who grew despite adversity merits further study, we should keep in mind that most of the youth in this sample experienced growth in the absence of adversity. Researchers will need to look elsewhere to identify the life experiences that reliably lead to growth for most people.

## Supplementary Material

1

2

3

4

## Figures and Tables

**Table 1:** Fit indices for conditional univariate latent growth curve models of youth and parent-reported effortful control and emotional stability.

Model	*χ* ^2^	DF	RMSEA	TLI	CFI
Effortful control youth-report	31.164	2	0.146	0.858	0.953
Effortful control parent-report	25.842	2	0.132	0.934	0.978
Emotional stability youth-report	86.559	2	0.249	0.264	0.755
Emotional stability parent-report	41.186	2	0.169	0.872	0.957
Adversity	9.007	2	0.072	0.958	0.986

*Note*: *n* = 682 for all models.

**Table 2:** Fit indices for bivariate latent growth curve models of youth and parent-reported effortful control and emotional stability.

Model	*χ* ^2^	DF	RMSEA	TLI	CFI
Effortful Control Youth-Report	56.236	9	0.088	0.908	0.961
Effortful Control Parent-Report	40.641	9	0.072	0.955	0.981
Emotional Stability Youth-Report	101.622	9	0.123	0.761	0.897
Emotional Stability Parent-Report	71.125	9	0.101	0.901	0.958

Note: *n* = 682 for all models.

**Table 3:** Unstandardized slopes for conditional univariate latent growth curve models of youth and parent-reported effortful control and emotional stability.

Construct	Parameter	*b*	*SE*	*p*	*Standardized Mean Difference*
Effortful Control Youth-Report	Slope Intercept	0.12	0.04	0.002	0.19
	Slope Variance	0.15	0.04	0.000	
Effortful Control Parent-Report	Slope Intercept	0.14	0.03	0.000	0.22
	Slope Variance	0.15	0.03	0.000	
Emotional Stability Youth-Report	Slope Intercept	0.37	0.04	0.000	0.57
	Slope Variance	0.14	0.04	0.000	
Emotional Stability Parent-Report	Slope Intercept	0.23	0.03	0.000	0.53
	Slope Variance	0.15	0.03	0.000	

Note: *n* = 682 for all models.

**Table 4:** Proportion of participants who increased and decreased relative to the smallest effect size of interest within adversity exposure subgroups.

Model	Low Adversity (*n* = 488; 76 %)		High Baseline Adversity Group (*n* = 93; 10 %)		Increases in Adversity Group (*n* = 63; 15 %)	
	Decrease	Increase	Decrease	Increase	Decrease	Increase
EC Youth-Report	17 % (83/488)	48 % (236/488)	17 % (16/93)	40 % (37/93)	38 % (24/63)	19 % (12/63)
EC Parent-Report	19 % (95/488)	49 % (239/488)	20 % (19/93)	47 % (44/93)	29 % (18/63)	38 % (24/63)
ES Youth-Report	3 % (16/488)	81 % (397/488)	5 % (5/93)	76 % (71/93)	19 % (12/63)	49 % (31/63)
ES Parent-Report	11 % (54/488)	72 % (353/488)	13 % (12/93)	66 % (61/93)	16 % (10/63)	54 % (34/63)

*Note*: The percentages for the three groups add up to 101 % because 3 participants overlapped in the Baseline and Increasing Adversity groups. EC = effortful control; ES = emotional stability. *n* = 641 for all models.

**Table 5:** Regression of effortful control and emotional stability slopes on change in adversity and third variable.

Outcome	*Predictor*	*b*	*SE*	*p*	*β*
EC parent slope	Adversity slope	−0.77	0.49	0.113	−0.44
	Anxiety slope	−0.16	0.07	0.024	−0.36
ES parent slope	Adversity slope	−0.72	0.48	0.138	−0.44
	Anxiety slope	−0.01	0.06	0.920	−0.01
EC parent slope	Adversity slope	−0.77	0.48	0.108	−0.46
	Avoidance slope	−0.07	0.03	0.043	−0.23
ES parent slope	Adversity slope	−0.56	0.44	0.205	−0.33
	Avoidance slope	−0.07	0.03	0.025	−0.25
EC parent slope	Adversity slope	−0.93	0.52	0.074	−0.56
	Peer support slope	0.00	0.02	0.934	0.01
ES parent slope	Adversity slope	−0.76	0.49	0.119	−0.46
	Peer support slope	0.00	0.02	0.820	−0.02
EC parent slope	Adversity slope	−0.88	0.50	0.078	−0.53
	Parenting slope	0.09	0.13	0.503	0.05
ES parent slope	Adversity slope	−0.70	0.46	0.134	−0.40
	Parenting slope	0.13	0.12	0.271	0.08

*Note:* EC = effortful control; ES = emotional stability.

## Data Availability

We cannot publicly share the data because the IRBs did not approve — and the participants did not consent to — sharing the data on a publicly accessible repository. The code is available on OSF: osf.io/ugxa2/.

## Footnotes

1 We also re-ran the conditional univariate and bivariate models for emotional stability and effortful control while controlling for gender, in addition to cohort. The resulting fixed effects parameters, and the covariances between the latent variables, were not meaningfully different. Gender did not significantly predict the slope of any of the variables; see Tables S25a–d in the Supplement.

2 Though the slope factor scores from unconditional univariate models were strongly correlated with the raw Time 3 – Time 1 difference scores (*r*s = 0.97-0.99), they also provide values for missing data and yield more conservative and informative estimates of predicted change because they are based on information from three waves.

3 We initially planned to use bivariate LGC models for this analysis, but the estimation of the factor scores for adversity fluctuated between the models, which made the denominator inconsistent and therefore complicated comparison. Therefore, we saved the factor scores from the univariate adversity model as an exogenous variable, which yielded a sample of 641 cases.

4 One potential explanation for the especially poor fit of the emotional stability models is initial elevation bias. Inspection of the means for each cohort indicated that there were several instances of an initial elevation bias (Anvari et al., 2023; Long et al., 2020) where the 1st wave reports were consistently lower than the final wave from the previous cohort. We quantified the extent of the bias as the Cohen’s *d* difference between the the first and final waves for the successive cohorts (Table S4). Insofar as it manifested in severe model misfit, the bias was most extreme for youth-reported emotional stability. Parent-reported emotional stability and youth-reported effortful control also suffered from the bias, but it was relatively smaller, and less consequential for model fit. Parent-reported effortful control was unaffected by the initial elevation bias. Inspection of the mean residuals for the youth-reported emotional stability LGC model suggested that the mis-fit could have occurred because of a large discrepancy between the observed and predicted mean for the second wave. It appears that this discrepancy occurred because the non-linear pattern of the initial elevation bias does not conform to the predictions from the linear growth curve model’s fixed factor loadings matrix.

## Appendix A. Supplementary materials

Supplementary materials for this article can be found online at https://doi.org/10.1016/j.jrp.2025.104628.
