# Supplementary material for: Growth following adversity is rare: Evidence from a multi-informant longitudinal study of children and adolescents
Source: J Res Pers. Author manuscript; Available in PMC 2026 Jun 10. (PMC13249454; doi:10.1016/j.jrp.2025.104628)
Supplement: 1 [file NIHMS2177602-supplement-1.pdf]

Preregistration Deviations Table
For the manuscript "Growth Following Adversity is Rare"
(adapted from Willroth & Atherton, 2023)

Table S1
Preregistration Deviations

#	Details		Original wording	Deviation description	Extent of deviation	Judgement of impact
1	Study	#1	"The first research question is how self-regulation, emotionality, and exposure to adversity change over the course of adolescence. This will be tested with **unconditional, univariate latent growth curve models** to determine whether there are systematic mean-level changes in self-regulation, emotionality, and adversity exposure, and to quantify individual differences in these patterns of change."	In addition to these unconditional models, we realized that we should also report univariate models that were conditioned on the grade cohort variable so we could test for age differences in the intercept and slope, and make the results more comparable to the main bivariate models, which included cohort as a covariate. We report the conditional univariate models in the main results.	Minor	We report the results from both the unconditional and conditional models in the Supplemental Results (see Table S6). The results do not differ meaningfully between the two model specifications.
	Type	Analyses				
	Reason	Typo/Error				
	Timing	After results known				
2	Study	#1	"Assuming there is statistically significant variance in these slopes, we will report the proportion of participants with slopes **greater than** our small effect size of interest in either direction (SESOI; i.e., $r = .10$; $d = .20$)." […] "For each subset of participants, we will report the variance of the distribution, and the proportion of participants with random slopes **greater than** our smallest effect size of interest in either direction."	This wording was ambiguous, since we had also said "greater than or equal to" the SESOI at other points in the pre-registration. We intended to say "greater than or equal to" throughout the entire pre-registration.	Minor	We did not consistently communicate this specific aspect of our analysis plan. We have tried to clarify our intended plan here so that there is less ambiguity about what we did.
	Type	Analyses				
	Reason	Typo/Error				
	Timing	After data access				
3	Study	#1	"We will first estimate	We were able to follow this plan for the	Major	We have tried to make it clear to readers

	Type<br>Reason<br>Timing	Analyses<br>Miscommunication<br>After data access	unconditional univariate latent growth curve models for each of the predictors of interest to verify whether there is meaningful change in the variable over time. Then, we will estimate a series of associative, tri-variate latent growth curve models between a given the predictor, temperament variable, and adversity to derive correlations between the intercept and slope of the predictor and the intercepts and slopes of temperament and adversity (Duncan et al., 2000).”	analysis of the attachment anxiety and avoidance data. However, we were not able to follow this in the case of our parenting (PSS) and and prosociality (RPEQ) data, since only two waves had been collected. We did not know this at the time. Therefore, we decided to compute latent change scores instead, and correlated the change scores with the slopes from LGC models for adversity and temperament in order to provide a comparable result to what we had initially pre-registered.		that the analyses with the PSS and RPEQ data were not pre-registered, and only utilized two waves of data, which constrains how informative the analyses are. However, we still believe that these were worth including in the results because the latent change score model still captures individual differences in change, and communicates conceptually similar information.
4	Study<br>Type<br>Reason<br>Timing	#1<br>Analyses<br>New knowledge<br>After data access	“To verify whether the association between adversity and temperament is explained by a third variable, **we will re-estimate the association between adversity and temperament intercepts and slopes while statistically controlling for the uni-directional effect of the intercept and slope of the third variable on the intercept and slope of adversity and temperament** (see Mu et al., 2019). If the associations between temperament and adversity are severely attenuated (which was the case in Mu et al., 2019), this would indicate that the additional factors explain the developmental trajectories of temperament and adversity. “	In the process of translating this verbal model into R code, we realized that we needed to make one modification in order for it to fully align with our research question and intentions. While the original text calls for estimating the correlation between the latent variables for adversity and temperament after regressing them on the intercept and slope of a third variable, we decided that a more easily interpretable test of this idea would be to regress the slope of temperament on the intercepts and slopes of adversity and the third variable. That way, the relative size of the regression coefficients for adversity and the third variables could be directly compared to one another.	Minor	This revision to the model specification slightly changes the interpretation of the model parameters compared to the original pre-registered. However, we believe that it is a better model to address our research question (i.e., “What predicts trajectories of positive change despite adversity?”) and does not dramatically deviate from the pre-registered model.

5	Study	#1	“The variables of attachment anxiety, attachment avoidance, and social competence are especially relevant based on a priori theorizing. We may conduct additional, exploratory analyses.”	There was a miscommunication among the authors about the content of the social competence measure. This scale actually contained self-esteem items, which we were not interested in, since self-esteem is very similar to low negative emotionality. We decided to not highlight these results in the main manuscript, but because we had already run the models we still report their results in the supplement in the interest of transparency.	Major	Readers should keep in mind that we ultimately did not end up analysing a measure of social competence, since it was ultimately not available. The results from the analyses with the data that we thought measured competence (but actually measure self-esteem) are included in the supplement for transparency.
	Type	Analyses				
	Reason	Miscommunication				
	Timing	After results known				
6	Study	#1	“The variables of attachment anxiety, attachment avoidance, and social competence are especially relevant based on a priori theorizing. We may conduct additional, exploratory analyses.”	We took another critical look at potentially relevant variables for the fourth research question after obtaining access to the data and running the initial models to address this research question, and we decided to run analyses for two additional variables: receipt of prosocial help from peers and youth-perceived parenting style.	Major	Readers should keep in mind that our analyses for the RPEQ and PSS were not pre-registered.
	Type	Analyses				
	Reason	New knowledge				
	Timing	After results known				
7	Study	#1	“In particular, **we are interested in comparing those who experience relatively little stress (score less than or equal to 2) to those who experienced moderate-to-severe stress (greater than 2)**. For the composite score that averages the 13 domain-specific stress ratings, we will partition the participants into a group whose scores are less than or equal to 2 (little to average/normal stress) and those whose composite scores are greater	While we stated in the pre-registration that we intended to compare the proportions between groups, we did not specify how we would do this beyond describing the differences. In order to formalize these comparisons, we tested whether the proportions were different between groups with inferential *z*-tests.	Major	This should be interpreted as a supplementary analysis that was added after the initial results were known in order to make the presentation of the results clearer and easier for readers to interpret.
	Type	Analyses				
	Reason	Other (Please Explain): Unregistered analysis				
	Timing	After results known				

			than 2 (moderate to severe). Because we also have longitudinal data on the development of adversity over time, we will also examine the subset of participants who experienced a marked increase in adversity over the course of the study and had an adversity slope greater than or equal to our SESOI. **We will compare these individuals to those who did not experience increasing adversity.**”			
8	Study	#1	“Finally, we will examine the self-regulation and emotionality **development of participants who met our criteria for high initial adversity *and* experienced increases in adversity over the course of the study >/= SESOI**. For each subset of participants, we will report the variance of the distribution, and the proportion of participants with random slopes greater than our smallest effect size of interest in either direction.”	We initially stated in our pre-registration that we would also examine the sub-population of participants who started out with high baseline adversity scores *and* meaningfully increased, but only three cases met this criterion, so further analyses would not have been warranted or informative.	Minor	We believe that it would not have been informative, and potentially misleading, to report proportional results for just three people, so we decided to not conduct these analyses or report them in the manuscript. We believe that this does not meaningfully change the interpretation of our results.
	Type	Analyses				
	Reason	New knowledge				
	Timing	After results known				
9	Study	#1	“First, the **regression** of the slope of self-regulation and emotionality change on the intercept of Time 1 adversity addresses the question of	This was a typo; we meant to say *correlation* in both instances.	Minor	This change helps aid the consistent and clear presentation of the results; we believe it does not change how the results would be interpreted substantively.
	Type	Analyses				
	Reas	Typo/Error				

	on		how initial adversity relates to changes in self-regulation and emotionality. Second, the **<u>correlation</u>** between the slope of self-regulation/emotionality development with the slope of adversity provides an estimate of the relation between changes in adversity and changes in self-regulation/emotionality."			
	Timing	After data access				
