# Supplementary material for: Growth following adversity is rare: Evidence from a multi-informant longitudinal study of children and adolescents
Source: J Res Pers. Author manuscript; Available in PMC 2026 Jun 10. (PMC13249454; doi:10.1016/j.jrp.2025.104628)
Supplement: 2 [file NIHMS2177602-supplement-2.docx]

**Supplement B: Supplemental Results**

**For the manuscript “Growth Following Adversity is Rare”**

Table of Contents

[Note on abbreviations in the Supplemental Materials. 4](#_Toc198126376)

[Table S1a. Descriptive Statistics, Reliability, and Manifest Correlations for Main Study Variables 5](#_Toc198126377)

[Table S1b. Descriptive Statistics, Reliability, Test-Retest Stability and Correlation with Adversity for EATQ Facets 7](#_Toc198126378)

[Table S2. Attrition 8](#_Toc198126379)

[Table S3. Univariate Model Fit 11](#_Toc198126380)

[Table S4. Initial Elevation Bias 14](#_Toc198126381)

[Table S5. Standardized Mean Difference Between t1 and t3 for Effortful Control, Emotional Stability, and Facets 16](#_Toc198126382)

[Table S6. Full Output for Youth and Parent Report Models for Univariate Growth in Facets 17](#_Toc198126383)

[Table S7. Bivariate Latent Growth Curve Model Fit 28](#_Toc198126384)

[Table S8a. Supplemental Slope-Slope and Intercept-Slope Correlations 30](#_Toc198126385)

[Table S8b. Full Output for Youth and Parent Report Models for Bivariate Growth in Facets and Adversity 32](#_Toc198126386)

[Table S9. Fit Indices for Univariate Latent Growth Curve Models for Effortful Control and Emotional Stability Conditioned on Cohort and Adversity Factor Scores 41](#_Toc198126387)

[Table S10. Youth and Parent Report Estimates for Prevalence of Growth Despite Adversity (Estimates for Univariate Model Conditioned on Initial Adversity, Change in Adversity, and Cohort) 42](#_Toc198126388)

[Table S11. Fit indices for all Research Question 04 models 43](#_Toc198126389)

[Table S12. Univariate Latent Difference Score Models for Parenting and Prosocial 44](#_Toc198126390)

[Table S13. Associations between Personality, Adversity, and Third Variable 45](#_Toc198126391)

[Table S14. Regression of Personality Intercept and Slope on Adversity and Third Variable 50](#_Toc198126392)

[Domain Level Adversity Robustness Analyses 53](#_Toc198126393)

[Table S15. Adversity Domain Univariate Latent Growth Curve Model Fit 54](#_Toc198126394)

[Table S16. Adversity Domain Univariate Latent Growth Curve Model Parameters 55](#_Toc198126395)

[Table S17. Adversity Domain Bivariate Latent Growth Curve Model Fit 58](#_Toc198126396)

[Table S18. Adversity Domain Bivariate Latent Growth Curve Model Parameters 61](#_Toc198126397)

[Table S19a. uv.onPCR.adv.fits.indices.csv 85](#_Toc198126398)

[Table S19b. uv.onPPR.adv.fits.indices.csv 86](#_Toc198126399)

[Table S19c. uv.onDisc.adv.fits.indices.csv 87](#_Toc198126400)

[Table S20a. Youth and Parent Report Estimates for Prevalence of Growth Despite Parent-Child Conflict (Estimates for Univariate Model Conditioned on Initial Adversity, Change in Adversity, and Cohort) 88](#_Toc198126401)

[Table S20b. Youth and Parent Report Estimates for Prevalence of Growth Despite Parent-Parent Conflict (Estimates for Univariate Model Conditioned on Initial Adversity, Change in Adversity, and Cohort) 89](#_Toc198126402)

[Table S20c. Youth and Parent Report Estimates for Prevalence of Growth Despite Discrimination/Acculturation Stress(Estimates for Univariate Model Conditioned on Initial Adversity, Change in Adversity, and Cohort) 90](#_Toc198126403)

[Table S21. Fit indices for domain specific trivariate regression models 91](#_Toc198126404)

[Table S22. Parameters from adversity domain trivariate regression models 92](#_Toc198126405)

[Table S23a. Demographic differences between youth who grew or did not grow in youth-reported effortful control following adversity 97](#_Toc198126406)

[Table S23b. Demographic differences between youth who grew or did not grow in parent-reported effortful control following adversity 98](#_Toc198126407)

[Table S23c. Demographic differences between youth who grew or did not grow in youth-reported emotional stability following adversity 99](#_Toc198126408)

[Table S23d. Demographic differences between youth who grew or did not grow in parent-reported emotional stability following adversity 100](#_Toc198126409)

[Table S24a. Demographic differences between youth who grew in youth-reported effortful control despite adversity or without adversity 101](#_Toc198126410)

[Table S24b. Demographic differences between youth who grew in parent-reported effortful control despite adversity or without adversity 102](#_Toc198126411)

[Table S24c. Demographic differences between youth who grew in youth-reported emotional stability despite adversity or without adversity 103](#_Toc198126412)

[Table S24d. Demographic differences between youth who grew in parent-reported emotional stability despite adversity or without adversity 104](#_Toc198126413)

[Table S25a. Regression of Univariate LGC Effortful Control and Emotional Stability Intercepts and Slopes on Gender (0 = Male, 1 = Female) 105](#_Toc198126414)

[Table S25b. Regression of Bivariate LGC Effortful Control and Emotional Stability Intercepts and Slopes on Gender (0 = Male, 1 = Female) 105](#_Toc198126415)

[Table S25c. Unstandardized Slopes for Univariate Latent Growth Curve Models of Youth and Parent-Reported Effortful Control and Emotional Stability Conditioned on Cohort and Gender 106](#_Toc198126416)

[Table S25d. Correlated Change Estimates for Adversity, Effortful Control, and Emotional Stability Slopes for Bivariate Latent Growth Curve Conditioned on Cohort and Gender 106](#_Toc198126417)

[Attachment Security to Caregiver 107](#_Toc198126418)

[Selected Items from the Parenting Styles Scale 108](#_Toc198126419)

[Selected Items from the Revised Peer Experiences Questionnaire (RPEQ; De Los Reyes & Prinstein, 2004) 111](#_Toc198126420)

[Self-Esteem 112](#_Toc198126421)

# Note on abbreviations in the Supplemental Materials.

There are several consistent abbreviations in the output for the supplemental materials that should be kept in mind. All of the EATQ personality constructs have the same naming convention: a 2-4 letter abbreviation, followed by a “y” or “p” to denote whether the construct was youth or parent reported, followed by a number to denote the measurement occasion (1, 2, or 3). For example, “ec.y.3” would represent youth-reported effortful control at the third wave, while “ne.p.1” represents parent-reported emotional stability (i.e., reverse-scored negative emotionality) at the first wave.

Activation Control = ac

Attention = at

Inhibitory Control = ic

Effortful Control = ec

Aggression = ag

Fear = fear

Frustration = fr

Shy = shy

Negative Emotionality/Emotional Stability = ne

Avoidance = avoid

Anxiety = anx

Parenting Style = PSS

Prosocial Behavior = ProSoc

Self-Esteem = SEQ

# Table S1a. Descriptive Statistics, Reliability, and Manifest Correlations for Main Study Variables

| Construct | *M* | *SD* | *α* | 1. | 2. | 3. | 4. | 5. | 6. | 7. | 8. | 9. | 10. |
| --- | --- | --- | --- | --- | --- | --- | --- | --- | --- | --- | --- | --- | --- |
| 1. EC (t1.y) | 3.42 | 0.55 | 0.74 |  |  |  |  |  |  |  |  |  |  |
| 2. EC (t2.y) | 3.56 | 0.57 | 0.81 | 0.58 |  |  |  |  |  |  |  |  |  |
| 3. EC (t3.y) | 3.53 | 0.55 | 0.81 | 0.50 | 0.67 |  |  |  |  |  |  |  |  |
| 4. EC (t1.p) | 3.24 | 0.56 | 0.85 | 0.34 | 0.25 | 0.30 |  |  |  |  |  |  |  |
| 5. EC (t2.p) | 3.37 | 0.58 | 0.86 | 0.28 | 0.31 | 0.36 | 0.77 |  |  |  |  |  |  |
| 6. EC (t3.p) | 3.36 | 0.58 | 0.86 | 0.30 | 0.29 | 0.41 | 0.70 | 0.81 |  |  |  |  |  |
| 7. ES (t1.y) | 3.34 | 0.53 | 0.79 | 0.38 | 0.26 | 0.14 | 0.15 | 0.12 | 0.05 |  |  |  |  |
| 8. ES (t2.y) | 3.64 | 0.5 | 0.83 | 0.32 | 0.50 | 0.39 | 0.07 | 0.09 | 0.05 | 0.46 |  |  |  |
| 9. ES (t3.y) | 3.64 | 0.49 | 0.82 | 0.24 | 0.36 | 0.53 | 0.12 | 0.13 | 0.12 | 0.32 | 0.55 |  |  |
| 10. ES (t1.p) | 3.43 | 0.49 | 0.82 | 0.09 | 0.08 | 0.10 | 0.42 | 0.37 | 0.32 | 0.15 | 0.21 | 0.18 |  |
| 11. ES (t2.p) | 3.64 | 0.53 | 0.86 | 0.14 | 0.13 | 0.23 | 0.39 | 0.52 | 0.45 | 0.17 | 0.21 | 0.26 | 0.72 |
| 12. ES (t3.p) | 3.69 | 0.52 | 0.86 | 0.12 | 0.11 | 0.20 | 0.36 | 0.45 | 0.48 | 0.14 | 0.20 | 0.24 | 0.64 |
| 13. Adv. (t1) | 1.64 | 0.36 | 0.79 | -0.30 | -0.31 | -0.30 | -0.18 | -0.20 | -0.18 | -0.18 | -0.20 | -0.20 | -0.08 |
| 14. Adv. (t2) | 1.56 | 0.37 | 0.81 | -0.20 | -0.33 | -0.31 | -0.15 | -0.19 | -0.20 | -0.09 | -0.19 | -0.23 | -0.11 |
| 15. Adv. (t3) | 1.55 | 0.43 | 0.80 | -0.14 | -0.18 | -0.34 | -0.08 | -0.15 | -0.25 | -0.03 | -0.14 | -0.29 | -0.08 |
| 16. Anxiety (t1.y) | 1.67 | 1.10 | 0.82 | -0.22 | -0.11 | -0.09 | -0.16 | -0.13 | -0.09 | -0.25 | -0.16 | -0.07 | -0.17 |
| 17. Anxiety (t2.y) | 1.58 | 0.97 | 0.83 | -0.19 | -0.26 | -0.24 | -0.13 | -0.21 | -0.13 | -0.21 | -0.28 | -0.23 | -0.19 |
| 18. Anxiety (t3.y) | 1.60 | 1.06 | 0.82 | -0.16 | -0.16 | -0.26 | -0.19 | -0.26 | -0.21 | -0.07 | -0.15 | -0.25 | -0.18 |
| 19. Avoidance (t1.y) | 2.72 | 1.31 | 0.81 | -0.26 | -0.17 | -0.08 | -0.11 | -0.07 | -0.05 | -0.04 | -0.10 | -0.04 | -0.04 |
| 20. Avoidance (t2.y) | 2.83 | 1.41 | 0.80 | -0.23 | -0.32 | -0.24 | -0.07 | -0.11 | -0.10 | -0.06 | -0.14 | -0.13 | -0.07 |
| 21. Avoidance (t3.y) | 2.94 | 1.47 | 0.82 | -0.20 | -0.22 | -0.29 | -0.13 | -0.15 | -0.16 | -0.01 | -0.07 | -0.18 | -0.11 |
| 22. Parenting (t2.y) | 0.85 | 0.16 | 0.59 | 0.15 | 0.20 | 0.16 | 0.13 | 0.10 | 0.13 | 0.07 | 0.13 | 0.11 | 0.17 |
| 23. Parenting (t3.y) | 0.89 | 0.16 | 0.71 | 0.10 | 0.11 | 0.18 | 0.09 | 0.09 | 0.12 | -0.01 | 0.06 | 0.10 | 0.14 |
| 24. Peer support (t2.y) | 2.62 | 0.99 | 0.65 | 0.06 | 0.08 | 0.10 | 0.04 | 0.07 | 0.04 | 0.00 | 0.01 | 0.05 | 0.04 |
| 25. Peer support (t3.y) | 2.57 | 0.91 | 0.60 | 0.09 | 0.09 | 0.15 | 0.12 | 0.08 | 0.09 | -0.01 | 0.06 | 0.03 | 0.03 |

**Table S1a.** Descriptive Statistics, Reliability, and Manifest Correlations for Main Study Variables (continued)

| Construct | 11. | 12. | 13. | 14. | 15. | 16. | 17. | 18. | 19. | 20. | 21. | 22. | 23. | 24. |
| --- | --- | --- | --- | --- | --- | --- | --- | --- | --- | --- | --- | --- | --- | --- |
| 1. EC (t1y) |  |  |  |  |  |  |  |  |  |  |  |  |  |  |
| 2. EC (t2y) |  |  |  |  |  |  |  |  |  |  |  |  |  |  |
| 3. EC (t3y) |  |  |  |  |  |  |  |  |  |  |  |  |  |  |
| 4. EC (t1p) |  |  |  |  |  |  |  |  |  |  |  |  |  |  |
| 5. EC (t2p) |  |  |  |  |  |  |  |  |  |  |  |  |  |  |
| 6. EC (t3p) |  |  |  |  |  |  |  |  |  |  |  |  |  |  |
| 7. NE (t1y) |  |  |  |  |  |  |  |  |  |  |  |  |  |  |
| 8. NE t2y) |  |  |  |  |  |  |  |  |  |  |  |  |  |  |
| 9. NE t3y) |  |  |  |  |  |  |  |  |  |  |  |  |  |  |
| 10. NE (t1p) |  |  |  |  |  |  |  |  |  |  |  |  |  |  |
| 11. NE (t2p) |  |  |  |  |  |  |  |  |  |  |  |  |  |  |
| 12. NE (t3p) | 0.77 |  |  |  |  |  |  |  |  |  |  |  |  |  |
| 13. Adv. t1 | -0.14 | -0.14 |  |  |  |  |  |  |  |  |  |  |  |  |
| 14. Adv. t2 | -0.18 | -0.13 | 0.64 |  |  |  |  |  |  |  |  |  |  |  |
| 15. Adv. t3 | -0.15 | -0.22 | 0.42 | 0.53 |  |  |  |  |  |  |  |  |  |  |
| 16. Anxiety t1 | -0.19 | -0.20 | 0.20 | 0.06 | 0.02 |  |  |  |  |  |  |  |  |  |
| 17. Anxiety t2 | -0.24 | -0.21 | 0.13 | 0.19 | 0.14 | 0.27 |  |  |  |  |  |  |  |  |
| 18. Anxiety t3 | -0.22 | -0.17 | 0.11 | 0.11 | 0.20 | 0.22 | 0.47 |  |  |  |  |  |  |  |
| 19. Avoidance t3 | -0.09 | -0.04 | 0.22 | 0.09 | 0.08 | 0.41 | 0.26 | 0.21 |  |  |  |  |  |  |
| 20. Avoidance t2 | -0.13 | -0.09 | 0.11 | 0.19 | 0.11 | 0.17 | 0.41 | 0.34 | 0.49 |  |  |  |  |  |
| 21. Avoidance t3 | -0.13 | -0.14 | 0.12 | 0.19 | 0.22 | 0.13 | 0.39 | 0.47 | 0.39 | 0.63 |  |  |  |  |
| 22. Parenting t2 | 0.14 | 0.07 | -0.05 | -0.12 | -0.06 | -0.15 | -0.32 | -0.27 | -0.27 | -0.49 | -0.35 |  |  |  |
| 23. Parenting t3 | 0.13 | 0.11 | -0.13 | -0.16 | -0.23 | -0.13 | -0.35 | -0.37 | -0.26 | -0.38 | -0.55 | 0.45 |  |  |
| 24. Peer support t2 | 0.09 | 0.08 | -0.06 | -0.05 | 0.02 | -0.04 | -0.19 | -0.12 | -0.16 | -0.24 | -0.25 | 0.21 | 0.16 |  |
| 25. Peer support t3 | 0.00 | 0.01 | -0.04 | -0.02 | -0.06 | 0.05 | -0.06 | -0.03 | -0.09 | -0.14 | -0.18 | 0.13 | 0.21 | 0.33 |

# Table S1b. Descriptive Statistics, Reliability, Test-Retest Stability and Correlation with Adversity for EATQ Facets

| Construct | *M* | *SD* | Alpha | t2 Stability | t3 Stability | Adv. t1 | Adv. t2 | Adv. t3 |
| --- | --- | --- | --- | --- | --- | --- | --- | --- |
| ac.y.1 | 3.27 | 0.84 | 0.66 | 0.56 | 0.51 | -0.29 | -0.21 | -0.14 |
| ac.y.2 | 3.3 | 0.86 | 0.76 | — | 0.64 | -0.27 | -0.28 | -0.14 |
| ac.y.3 | 3.16 | 0.84 | 0.77 | — | — | -0.26 | -0.25 | -0.3 |
| ac.p.1 | 3.11 | 0.78 | 0.81 | 0.73 | 0.65 | -0.2 | -0.18 | -0.09 |
| ac.p.2 | 3.21 | 0.78 | 0.82 | — | 0.77 | -0.21 | -0.22 | -0.15 |
| ac.p.3 | 3.13 | 0.82 | 0.84 | — | — | -0.17 | -0.2 | -0.24 |
| at.y.1 | 3.38 | 0.63 | 0.5 | 0.42 | 0.3 | -0.25 | -0.16 | -0.1 |
| at.y.2 | 3.54 | 0.61 | 0.59 | — | 0.58 | -0.26 | -0.29 | -0.17 |
| at.y.3 | 3.53 | 0.59 | 0.58 | — | — | -0.24 | -0.29 | -0.3 |
| at.p.1 | 3.05 | 0.6 | 0.6 | 0.66 | 0.62 | -0.13 | -0.13 | -0.07 |
| at.p.2 | 3.16 | 0.61 | 0.64 | — | 0.68 | -0.15 | -0.12 | -0.12 |
| at.p.3 | 3.18 | 0.59 | 0.58 | — | — | -0.13 | -0.12 | -0.17 |
| ic.y.1 | 3.63 | 0.63 | 0.31 | 0.45 | 0.38 | -0.16 | -0.08 | -0.09 |
| ic.y.2 | 3.85 | 0.59 | 0.43 | — | 0.48 | -0.22 | -0.24 | -0.15 |
| ic.y.3 | 3.89 | 0.63 | 0.56 | — | — | -0.22 | -0.2 | -0.22 |
| ic.p.1 | 3.64 | 0.58 | 0.51 | 0.6 | 0.52 | -0.1 | -0.03 | 0 |
| ic.p.2 | 3.85 | 0.59 | 0.54 | — | 0.65 | -0.14 | -0.13 | -0.1 |
| ic.p.3 | 3.89 | 0.6 | 0.57 | — | — | -0.15 | -0.17 | -0.23 |
| ag.y.1 | 1.97 | 0.73 | 0.76 | 0.55 | 0.4 | -0.28 | -0.19 | -0.14 |
| ag.y.2 | 1.87 | 0.68 | 0.78 | — | 0.57 | -0.25 | -0.26 | -0.16 |
| ag.y.3 | 1.9 | 0.69 | 0.8 | — | — | -0.24 | -0.28 | -0.3 |
| ag.p.1 | 2.37 | 0.69 | 0.75 | 0.69 | 0.62 | -0.18 | -0.14 | -0.13 |
| ag.p.2 | 2.17 | 0.7 | 0.78 | — | 0.72 | -0.18 | -0.21 | -0.18 |
| ag.p.3 | 2.14 | 0.65 | 0.74 | — | — | -0.16 | -0.19 | -0.28 |
| fear.y.1 | 2.81 | 0.81 | 0.63 | 0.46 | 0.36 | -0.02 | 0 | 0.04 |
| fear.y.2 | 2.24 | 0.71 | 0.65 | — | 0.51 | -0.07 | -0.04 | 0.01 |
| fear.y.3 | 2.1 | 0.65 | 0.66 | — | — | -0.02 | -0.06 | -0.08 |
| fear.p.1 | 2.48 | 0.71 | 0.64 | 0.65 | 0.58 | -0.01 | -0.04 | -0.04 |
| fear.p.2 | 2.14 | 0.69 | 0.68 | — | 0.67 | -0.05 | -0.09 | -0.09 |
| fear.p.3 | 2.04 | 0.68 | 0.69 | — | — | -0.07 | -0.04 | -0.08 |
| fr.y.1 | 3.18 | 0.72 | 0.71 | 0.44 | 0.32 | -0.18 | -0.06 | -0.03 |
| fr.y.2 | 2.9 | 0.71 | 0.76 | — | 0.49 | -0.16 | -0.17 | -0.12 |
| fr.y.3 | 2.93 | 0.69 | 0.75 | — | — | -0.18 | -0.19 | -0.22 |
| fr.p.1 | 2.93 | 0.67 | 0.77 | 0.64 | 0.58 | -0.13 | -0.13 | -0.07 |
| fr.p.2 | 2.72 | 0.71 | 0.79 | — | 0.66 | -0.19 | -0.2 | -0.15 |
| fr.p.3 | 2.7 | 0.71 | 0.8 | — | — | -0.16 | -0.15 | -0.22 |
| shy.y.1 | 2.57 | 1.02 | 0.75 | 0.48 | 0.44 | 0 | 0.01 | 0.03 |
| shy.y.2 | 2.3 | 0.91 | 0.8 | — | 0.61 | -0.04 | -0.04 | -0.08 |
| shy.y.3 | 2.43 | 0.99 | 0.84 | — | — | -0.07 | -0.08 | -0.14 |
| shy.p.1 | 2.51 | 0.9 | 0.86 | 0.71 | 0.68 | 0.11 | 0.02 | 0.02 |
| shy.p.2 | 2.44 | 0.88 | 0.86 | — | 0.76 | 0.02 | -0.01 | 0 |
| shy.p.3 | 2.42 | 0.9 | 0.86 | — | — | 0 | 0.01 | -0.03 |

#

#

# Table S2. Attrition

| Dependent Variable | *Dropout*  *M* | *Stay M* | *t/z* | *df* | *p* | *d/h* |
| --- | --- | --- | --- | --- | --- | --- |
| Site | 0.44 | 0.47 | -0.63 | 1 | 0.526 | -0.06 |
| Child Gender | 0.54 | 0.56 | -0.55 | 1 | 0.580 | -0.05 |
| Child Ethnicity | 0.21 | 0.10 | 3.82 | 1 | 0.000 | 0.32 |
| Child Race | 0.44 | 0.28 | 3.78 | 1 | 0.000 | 0.33 |
| Child Age | 12.06 | 11.77 | 1.29 | 254.32 | .198 | 0.12 |
| Child Grade | 6.20 | 6.02 | 0.81 | 259.05 | .418 | 0.07 |
| Parent Gender | 0.94 | 0.92 | 0.67 | 1 | 0.501 | 0.06 |
| Parent Ethnicity | 0.15 | 0.09 | 1.93 | 1 | 0.054 | 0.17 |
| Parent Race | 0.35 | 0.25 | 2.58 | 1 | 0.010 | 0.23 |
| Parent Marital Status | 0.73 | 0.77 | -1.21 | 1 | 0.227 | -0.11 |
| Parent Education | 5.24 | 5.75 | -4.47 | 250.05 | < .001*** | -0.42 |
| Other Parent Education | 4.67 | 5.35 | -4.45 | 244.96 | < .001*** | -0.43 |
| School Lunch (PR) | 0.32 | 0.14 | 4.98 | 1 | 0.000 | 0.42 |
| Food Stamps (PR) | 0.12 | 0.05 | 3.26 | 1 | 0.001 | 0.27 |
| Total Income (PR) | 88,877.78 | 102,382.34 | -1.64 | 218.98 | .103 | -0.17 |
| People Home (PR) | 4.49 | 4.16 | 2.32 | 207.33 | .021* | 0.25 |
| adv.1 | 1.70 | 1.62 | 2.10 | 192.64 | .037* | 0.23 |
| ec.y.1 | 3.39 | 3.43 | -0.87 | 249.66 | .386 | -0.08 |
| ec.p.1 | 3.18 | 3.25 | -1.27 | 241.90 | .204 | -0.12 |
| ac.y.1 | 3.22 | 3.29 | -0.88 | 253.75 | .378 | -0.08 |
| ac.p.1 | 3.04 | 3.12 | -1.09 | 233.17 | .276 | -0.11 |
| at.y.1 | 3.36 | 3.39 | -0.40 | 248.78 | .688 | -0.04 |
| at.p.1 | 3.02 | 3.06 | -0.79 | 251.33 | .433 | -0.07 |
| ic.y.1 | 3.59 | 3.64 | -0.75 | 256.17 | .455 | -0.07 |
| ic.p.1 | 3.58 | 3.66 | -1.35 | 252.10 | .177 | -0.12 |
| ne.y.1 | 3.28 | 3.36 | -1.48 | 246.44 | .141 | -0.14 |
| ne.p.1 | 3.38 | 3.45 | -1.57 | 264.90 | .117 | -0.14 |
| ag.y.1 | 3.92 | 4.06 | -1.90 | 241.45 | .059 | -0.18 |
| ag.p.1 | 3.57 | 3.65 | -1.17 | 241.78 | .244 | -0.11 |
| fear.y.1 | 3.17 | 3.20 | -0.35 | 247.95 | .724 | -0.03 |
| fear.p.1 | 3.46 | 3.53 | -1.03 | 262.64 | .306 | -0.09 |
| fr.y.1 | 2.74 | 2.85 | -1.59 | 255.74 | .112 | -0.15 |
| fr.p.1 | 3.06 | 3.08 | -0.33 | 249.94 | .742 | -0.03 |
| shy.y.1 | 3.44 | 3.43 | 0.10 | 267.44 | .922 | 0.01 |
| shy.p.1 | 3.40 | 3.51 | -1.33 | 243.56 | .185 | -0.13 |
| anx.y.1 | 1.71 | 1.66 | 0.43 | 242.45 | .669 | 0.04 |
| avoid.y.1 | 2.72 | 2.72 | 0.00 | 233.45 | 1.00 | 0.00 |

*Note:* Parent Education was originally a 9-category nominal variable (1 = 8th grade or less; 2 = some high school; 3 = finished high school; 4 = completed GED; 5 = vocational/trade/business school; 6 = some college or 2 year degree; 7 = finished 4 year degree; 8 = masters or equivalent; 9 = other advanced degree). Levels 3 and 4, and 8 and 9, were collapsed together in order to make an ordered numeric variable so that inferential tests for attrition were interpretable. Child Race and Parent Race were also originally multi-category nominal variables (descriptives are in Hankin et al., 2015) that were re-coded into binary variables for the purposes of attrition tests (1 = non-Caucasian; Caucasian = 0). Parent Marital Status was re-coded so that 0 = Not Married and 1 = Married. For both of the Gender variables, 0 = Male and 1 = Female.

# Table S3. Univariate Model Fit

| chisq | df | rmsea | tli | cfi | aic | bic | ModID |
| --- | --- | --- | --- | --- | --- | --- | --- |
| 63.719 | 4 | 0.148 | 0.919 | 0.892 | 2465.248 | 2487.873 | ec.y.uv.Mod0 |
| 24.122 | 1 | 0.184 | 0.875 | 0.958 | 2431.652 | 2467.852 | ec.y.uv.Mod1 |
| 31.164 | 2 | 0.146 | 0.858 | 0.953 | 2379.031 | 2424.282 | ec.y.uv.Mod2 |
| 85.888 | 4 | 0.174 | 0.943 | 0.924 | 2064.142 | 2086.723 | ec.p.uv.Mod0 |
| 23.199 | 1 | 0.181 | 0.938 | 0.979 | 2007.453 | 2043.582 | ec.p.uv.Mod1 |
| 25.842 | 2 | 0.132 | 0.934 | 0.978 | 2002.635 | 2047.885 | ec.p.uv.Mod2 |
| 33.596 | 4 | 0.104 | 0.957 | 0.943 | 3963.11 | 3985.735 | ac.y.uv.Mod0 |
| 9.13 | 1 | 0.109 | 0.953 | 0.984 | 3944.644 | 3980.845 | ac.y.uv.Mod1 |
| 10.119 | 2 | 0.077 | 0.963 | 0.988 | 3810.239 | 3855.489 | ac.y.uv.Mod2 |
| 33.925 | 4 | 0.105 | 0.976 | 0.968 | 3310.256 | 3332.837 | ac.p.uv.Mod0 |
| 15.378 | 1 | 0.146 | 0.954 | 0.985 | 3297.708 | 3333.837 | ac.p.uv.Mod1 |
| 19.765 | 2 | 0.114 | 0.943 | 0.981 | 3301.262 | 3346.513 | ac.p.uv.Mod2 |
| 68.951 | 4 | 0.154 | 0.847 | 0.796 | 3045.127 | 3067.753 | at.y.uv.Mod0 |
| 14.047 | 1 | 0.138 | 0.877 | 0.959 | 2996.223 | 3032.423 | at.y.uv.Mod1 |
| 21.441 | 2 | 0.119 | 0.826 | 0.942 | 2987.608 | 3032.859 | at.y.uv.Mod2 |
| 41.934 | 4 | 0.118 | 0.96 | 0.946 | 2569.137 | 2591.718 | at.p.uv.Mod0 |
| 4.463 | 1 | 0.072 | 0.985 | 0.995 | 2537.666 | 2573.795 | at.p.uv.Mod1 |
| 7.386 | 2 | 0.063 | 0.978 | 0.993 | 2513.224 | 2558.474 | at.p.uv.Mod2 |
| 102.19 | 4 | 0.19 | 0.741 | 0.655 | 3124.009 | 3146.634 | ic.y.uv.Mod0 |
| 17.714 | 1 | 0.157 | 0.824 | 0.941 | 3045.533 | 3081.733 | ic.y.uv.Mod1 |
| 22.686 | 2 | 0.123 | 0.789 | 0.93 | 3042.34 | 3087.59 | ic.y.uv.Mod2 |
| 136.946 | 4 | 0.222 | 0.821 | 0.762 | 2758.768 | 2781.349 | ic.p.uv.Mod0 |
| 17.866 | 1 | 0.158 | 0.909 | 0.97 | 2645.688 | 2681.818 | ic.p.uv.Mod1 |
| 18.587 | 2 | 0.11 | 0.915 | 0.972 | 2621.63 | 2666.881 | ic.p.uv.Mod2 |
| 218.997 | 4 | 0.281 | 0.5 | 0.333 | 2520.634 | 2543.259 | ne.y.uv.Mod0 |
| 70.896 | 1 | 0.32 | 0.35 | 0.783 | 2378.533 | 2414.733 | ne.y.uv.Mod1 |
| 86.559 | 2 | 0.249 | 0.264 | 0.755 | 2372.982 | 2418.232 | ne.y.uv.Mod2 |
| 249.069 | 4 | 0.301 | 0.795 | 0.727 | 1999.098 | 2021.679 | ne.p.uv.Mod0 |
| 39.592 | 1 | 0.239 | 0.871 | 0.957 | 1795.621 | 1831.751 | ne.p.uv.Mod1 |
| 41.186 | 2 | 0.169 | 0.872 | 0.957 | 1777.776 | 1823.026 | ne.p.uv.Mod2 |
| 25.497 | 4 | 0.089 | 0.961 | 0.948 | 3402.268 | 3424.893 | ag.y.uv.Mod0 |
| 5.172 | 1 | 0.078 | 0.97 | 0.99 | 3387.943 | 3424.144 | ag.y.uv.Mod1 |
| 18.879 | 2 | 0.111 | 0.891 | 0.964 | 3349.737 | 3394.987 | ag.y.uv.Mod2 |
| 106.028 | 4 | 0.194 | 0.902 | 0.869 | 3019.182 | 3041.762 | ag.p.uv.Mod0 |
| 18.683 | 1 | 0.162 | 0.932 | 0.977 | 2937.836 | 2973.966 | ag.p.uv.Mod1 |
| 20.553 | 2 | 0.117 | 0.929 | 0.976 | 2936.289 | 2981.54 | ag.p.uv.Mod2 |
| 382.926 | 4 | 0.373 | 0.044 | 0 | 3983.429 | 4006.054 | fear.y.uv.Mod0 |
| 69.471 | 1 | 0.317 | 0.309 | 0.77 | 3675.974 | 3712.174 | fear.y.uv.Mod1 |
| 77.644 | 2 | 0.235 | 0.328 | 0.776 | 3644.793 | 3690.044 | fear.y.uv.Mod2 |
| 266.55 | 4 | 0.312 | 0.699 | 0.598 | 3369.168 | 3391.749 | fear.p.uv.Mod0 |
| 39.722 | 1 | 0.239 | 0.822 | 0.941 | 3148.34 | 3184.469 | fear.p.uv.Mod1 |
| 40.489 | 2 | 0.168 | 0.834 | 0.945 | 3107.221 | 3152.471 | fear.p.uv.Mod2 |
| 94.653 | 4 | 0.182 | 0.749 | 0.665 | 3625.148 | 3647.773 | fr.y.uv.Mod0 |
| 36.417 | 1 | 0.228 | 0.608 | 0.869 | 3572.912 | 3609.112 | fr.y.uv.Mod1 |
| 42.168 | 2 | 0.172 | 0.578 | 0.859 | 3564.752 | 3610.002 | fr.y.uv.Mod2 |
| 104.926 | 4 | 0.193 | 0.88 | 0.84 | 3217.917 | 3240.498 | fr.p.uv.Mod0 |
| 17.649 | 1 | 0.157 | 0.921 | 0.974 | 3136.64 | 3172.77 | fr.p.uv.Mod1 |
| 19.421 | 2 | 0.113 | 0.919 | 0.973 | 3129.517 | 3174.768 | fr.p.uv.Mod2 |
| 50.669 | 4 | 0.131 | 0.915 | 0.887 | 4572.885 | 4595.51 | shy.y.uv.Mod0 |
| 33.13 | 1 | 0.217 | 0.766 | 0.922 | 4561.346 | 4597.547 | shy.y.uv.Mod1 |
| 37.206 | 2 | 0.161 | 0.753 | 0.918 | 4550.075 | 4595.325 | shy.y.uv.Mod2 |
| 16.364 | 4 | 0.068 | 0.99 | 0.986 | 3737.72 | 3760.301 | shy.p.uv.Mod0 |
| 0.651 | 1 | 0 | 1.001 | 1 | 3728.007 | 3764.136 | shy.p.uv.Mod1 |
| 2.321 | 2 | 0.015 | 0.999 | 1 | 3728.919 | 3774.169 | shy.p.uv.Mod2 |
| 48.505 | 4 | 0.13 | 0.925 | 0.9 | 1131.982 | 1154.465 | adv.uv.Mod0 |
| 7.15 | 1 | 0.096 | 0.958 | 0.986 | 1096.627 | 1132.601 | adv.uv.Mod1 |
| 9.007 | 2 | 0.072 | 0.958 | 0.986 | 1042.476 | 1087.726 | adv.uv.Mod2 |
| 54.894 | 4 | 0.138 | 0.906 | 0.875 | 5712.707 | 5735.259 | avoid.uv.Mod0 |
| 0.014 | 1 | 0 | 1.007 | 1 | 5663.827 | 5699.909 | avoid.uv.Mod1 |
| 4.06 | 2 | 0.039 | 0.986 | 0.995 | 5644.037 | 5689.287 | avoid.uv.Mod2 |
| 21.735 | 4 | 0.081 | 0.92 | 0.893 | 4940.863 | 4963.414 | anx.uv.Mod0 |
| 1.984 | 1 | 0.038 | 0.982 | 0.994 | 4927.112 | 4963.194 | anx.uv.Mod1 |
| 3.596 | 2 | 0.034 | 0.972 | 0.991 | 4926.924 | 4972.174 | anx.uv.Mod2 |

*Note*: Mod0 represents the No Growth model, Mod1 represents the unconditional univariate model, and Mod2 represents the conditional (on cohort) univariate model.

# Table S4. Initial Elevation Bias

| Construct |  | C1 Mean | C2 Mean | C3 Mean | W1-W3 Cohen’s *d* difference | W1-W3  *p*-value |
| --- | --- | --- | --- | --- | --- | --- |
| Emotional Stability (y) | C1 v. C2 (6^th^ grade) | 3.72 | 3.38 |  | -0.68 | 0.000 |
|  | C2 v. C3 (9^th^ grade) |  | 3.61 | 3.35 | -0.55 | 0.000 |
| Emotional Stability (p) | C1 v. C2 (6^th^ grade) | 3.58 | 3.46 |  | -0.27 | 0.012 |
|  | C2 v. C3 (9^th^ grade) |  | 3.68 | 3.51 | -0.32 | 0.001 |
| Aggression (y) | C1 v. C2 (6^th^ grade) | 4.30 | 4.03 |  | -0.41 | 0.000 |
|  | C2 v. C3 (9^th^ grade) |  | 4.01 | 3.86 | -0.21 | 0.033 |
| Aggression (p) | C1 v. C2 (6^th^ grade) | 3.80 | 3.64 |  | -0.25 | 0.016 |
|  | C2 v. C3 (9^th^ grade) |  | 3.83 | 3.68 | -0.21 | 0.032 |
| Fear (y) | C1 v. C2 (6^th^ grade) | 3.76 | 3.22 |  | -0.73 | 0.000 |
|  | C2 v. C3 (9^th^ grade) |  | 3.97 | 3.41 | -0.85 | 0.000 |
| Fear (p) | C1 v. C2 (6^th^ grade) | 3.74 | 3.53 |  | -0.31 | 0.003 |
|  | C2 v. C3 (9^th^ grade) |  | 4.04 | 3.70 | -0.49 | 0.000 |
| Frustration (y) | C1 v. C2 (6^th^ grade) | 3.21 | 2.87 |  | -0.47 | 0.000 |
|  | C2 v. C3 (9^th^ grade) |  | 3.00 | 2.76 | -0.34 | 0.001 |
| Frustration (p) | C1 v. C2 (6^th^ grade) | 3.19 | 3.14 |  | -0.08 | 0.439 |
|  | C2 v. C3 (9^th^ grade) |  | 3.29 | 3.08 | -0.29 | 0.003 |
| Shy (y) | C1 v. C2 (6^th^ grade) | 3.67 | 3.51 |  | -0.16 | 0.112 |
|  | C2 v. C3 (9^th^ grade) |  | 3.56 | 3.52 | -0.05 | 0.634 |
| Shy (p) | C1 v. C2 (6^th^ grade) | 3.55 | 3.50 |  | -0.05 | 0.611 |
|  | C2 v. C3 (9^th^ grade) |  | 3.51 | 3.56 | 0.06 | 0.570 |
| Effortful Control (y) | C1 v. C2 (6^th^ grade) | 3.75 | 3.45 |  | -0.52 | 0.000 |
|  | C2 v. C3 (9^th^ grade) |  | 3.48 | 3.27 | -0.41 | 0.000 |
| Effortful Control (p) | C1 v. C2 (6^th^ grade) | 3.32 | 3.25 |  | -0.13 | 0.226 |
|  | C2 v. C3 (9^th^ grade) |  | 3.34 | 3.30 | -0.08 | 0.410 |
| Activation Control (y) | C1 v. C2 (6^th^ grade) | 3.66 | 3.33 |  | -0.43 | 0.000 |
|  | C2 v. C3 (9^th^ grade) |  | 3.04 | 2.89 | -0.19 | 0.049 |
| Activation Control (p) | C1 v. C2 (6^th^ grade) | 3.21 | 3.14 |  | -0.09 | 0.369 |
|  | C2 v. C3 (9^th^ grade) |  | 3.07 | 3.05 | -0.03 | 0.793 |
| Attention (y) | C1 v. C2 (6^th^ grade) | 3.64 | 3.39 |  | -0.38 | 0.000 |
|  | C2 v. C3 (9^th^ grade) |  | 3.52 | 3.33 | -0.34 | 0.001 |
| Attention (p) | C1 v. C2 (6^th^ grade) | 3.05 | 3.04 |  | -0.02 | 0.850 |
|  | C2 v. C3 (9^th^ grade) |  | 3.18 | 3.19 | 0.01 | 0.913 |
| Inhibitory Control (y) | C1 v. C2 (6^th^ grade) | 3.97 | 3.64 |  | -0.49 | 0.000 |
|  | C2 v. C3 (9^th^ grade) |  | 3.85 | 3.58 | -0.50 | 0.000 |
| Inhibitory Control (p) | C1 v. C2 (6^th^ grade) | 3.81 | 3.67 |  | -0.24 | 0.019 |
|  | C2 v. C3 (9^th^ grade) |  | 3.91 | 3.77 | -0.24 | 0.014 |

*Note:* Cohort 1 and 2 are compared when both cohorts are in the 6^th^ grade (similarly for Cohorts 2 and 3 for 9^th^ grade); therefore, differences at this assessment are likely due to initial elevation bias, as Cohorts 2 and 3 reliably self-report increased negative emotionality, aggression and fear.

# Table S5. Standardized Mean Difference Between t1 and t3 for Effortful Control, Emotional Stability, and Facets

| Construct | Std. Mean Diff. | 3^rd^ Grade Std. Mean Diff. | 6^th^ Grade Std. Mean Diff. | 9^th^ Grade Std. Mean Diff. |
| --- | --- | --- | --- | --- |
| Effortful Control (y) | 0.19 | 0.35 | 0.05 | 0.21 |
| Effortful Control (p) | 0.22 | 0.31 | 0.16 | 0.19 |
| Activation Control (y) | -0.13 | 0.07 | -0.36 | -0.08 |
| Activation Control (p) | 0.03 | 0.11 | -0.09 | 0.09 |
| Attention (y) | 0.23 | 0.31 | 0.21 | 0.18 |
| Attention (p) | 0.21 | 0.23 | 0.24 | 0.16 |
| Inhibitory Control (y) | 0.42 | 0.45 | 0.31 | 0.55 |
| Inhibitory Control (p) | 0.44 | 0.6 | 0.44 | 0.32 |
| Negative Emotionality (y) | 0.57 | 0.67 | 0.49 | 0.55 |
| Negative Emotionality (p) | 0.53 | 0.54 | 0.54 | 0.53 |
| Aggression (y) | 0.11 | 0.12 | -0.04 | 0.24 |
| Aggression (p) | 0.33 | 0.35 | 0.29 | 0.36 |
| Fear (y) | 0.86 | 0.9 | 0.98 | 0.79 |
| Fear (p) | 0.63 | 0.6 | 0.79 | 0.54 |
| Frustration (y) | 0.33 | 0.45 | 0.18 | 0.38 |
| Frustration (p) | 0.34 | 0.31 | 0.25 | 0.46 |
| Shy (y) | 0.14 | 0.39 | 0.05 | -0.03 |
| Shy (p) | 0.1 | 0.17 | 0.01 | 0.14 |

# Table S6. Full Output for Youth and Parent Report Models for Univariate Growth in Facets

| Parameter | Estimate | SE | PValue | StdAll | Construct | ModelName |
| --- | --- | --- | --- | --- | --- | --- |
| InterceptOfIntercept | 3.50 | 0.02 | 0.000 | 8.17 | ec.y | ec.y.uv.Mod0 |
| VarianceOfIntercept | 0.18 | 0.01 | 0.000 | 1.00 | ec.y | ec.y.uv.Mod0 |
| InterceptOfIntercept | 3.44 | 0.02 | 0.000 | 7.42 | ec.y | ec.y.uv.Mod1 |
| VarianceOfIntercept | 0.22 | 0.02 | 0.000 | 1.00 | ec.y | ec.y.uv.Mod1 |
| InterceptOfSlope | 0.09 | 0.02 | 0.000 | 0.22 | ec.y | ec.y.uv.Mod1 |
| VarianceOfSlope | 0.18 | 0.04 | 0.000 | 1.00 | ec.y | ec.y.uv.Mod1 |
| InterceptSlopeCorr | -0.06 | 0.02 | 0.006 | -0.33 | ec.y | ec.y.uv.Mod1 |
| InterceptOfIntercept | 3.61 | 0.03 | 0.000 | 7.95 | ec.y | ec.y.uv.Mod2 |
| VarianceOfIntercept | 0.19 | 0.02 | 0.000 | 0.92 | ec.y | ec.y.uv.Mod2 |
| InterceptOfSlope | 0.12 | 0.04 | 0.002 | 0.31 | ec.y | ec.y.uv.Mod2 |
| VarianceOfSlope | 0.15 | 0.04 | 0.000 | 1.00 | ec.y | ec.y.uv.Mod2 |
| InterceptSlopeCorr | -0.06 | 0.02 | 0.016 | -0.33 | ec.y | ec.y.uv.Mod2 |
| InterceptOnCohort | -0.16 | 0.03 | 0.000 | -0.28 | ec.y | ec.y.uv.Mod2 |
| SlopeOnCohort | -0.03 | 0.03 | 0.368 | -0.05 | ec.y | ec.y.uv.Mod2 |
| InterceptOfIntercept | 3.32 | 0.02 | 0.000 | 6.54 | ec.p | ec.p.uv.Mod0 |
| VarianceOfIntercept | 0.26 | 0.02 | 0.000 | 1.00 | ec.p | ec.p.uv.Mod0 |
| InterceptOfIntercept | 3.25 | 0.02 | 0.000 | 6.13 | ec.p | ec.p.uv.Mod1 |
| VarianceOfIntercept | 0.28 | 0.02 | 0.000 | 1.00 | ec.p | ec.p.uv.Mod1 |
| InterceptOfSlope | 0.11 | 0.02 | 0.000 | 0.28 | ec.p | ec.p.uv.Mod1 |
| VarianceOfSlope | 0.15 | 0.03 | 0.000 | 1.00 | ec.p | ec.p.uv.Mod1 |
| InterceptSlopeCorr | -0.05 | 0.02 | 0.004 | -0.26 | ec.p | ec.p.uv.Mod1 |
| InterceptOfIntercept | 3.17 | 0.03 | 0.000 | 6.01 | ec.p | ec.p.uv.Mod2 |
| VarianceOfIntercept | 0.27 | 0.02 | 0.000 | 0.99 | ec.p | ec.p.uv.Mod2 |
| InterceptOfSlope | 0.14 | 0.03 | 0.000 | 0.37 | ec.p | ec.p.uv.Mod2 |
| VarianceOfSlope | 0.15 | 0.03 | 0.000 | 1.00 | ec.p | ec.p.uv.Mod2 |
| InterceptSlopeCorr | -0.05 | 0.02 | 0.008 | -0.24 | ec.p | ec.p.uv.Mod2 |
| InterceptOnCohort | 0.08 | 0.03 | 0.003 | 0.12 | ec.p | ec.p.uv.Mod2 |
| SlopeOnCohort | -0.03 | 0.02 | 0.175 | -0.07 | ec.p | ec.p.uv.Mod2 |
| InterceptOfIntercept | 3.24 | 0.03 | 0.000 | 5.06 | ac.y | ac.y.uv.Mod0 |
| VarianceOfIntercept | 0.41 | 0.03 | 0.000 | 1.00 | ac.y | ac.y.uv.Mod0 |
| InterceptOfIntercept | 3.29 | 0.03 | 0.000 | 4.83 | ac.y | ac.y.uv.Mod1 |
| VarianceOfIntercept | 0.47 | 0.05 | 0.000 | 1.00 | ac.y | ac.y.uv.Mod1 |
| InterceptOfSlope | -0.13 | 0.04 | 0.000 | -0.23 | ac.y | ac.y.uv.Mod1 |
| VarianceOfSlope | 0.30 | 0.10 | 0.002 | 1.00 | ac.y | ac.y.uv.Mod1 |
| InterceptSlopeCorr | -0.10 | 0.05 | 0.060 | -0.28 | ac.y | ac.y.uv.Mod1 |
| InterceptOfIntercept | 3.68 | 0.05 | 0.000 | 5.47 | ac.y | ac.y.uv.Mod2 |
| VarianceOfIntercept | 0.36 | 0.05 | 0.000 | 0.80 | ac.y | ac.y.uv.Mod2 |
| InterceptOfSlope | -0.08 | 0.06 | 0.173 | -0.15 | ac.y | ac.y.uv.Mod2 |
| VarianceOfSlope | 0.27 | 0.09 | 0.003 | 0.99 | ac.y | ac.y.uv.Mod2 |
| InterceptSlopeCorr | -0.10 | 0.05 | 0.055 | -0.32 | ac.y | ac.y.uv.Mod2 |
| InterceptOnCohort | -0.37 | 0.04 | 0.000 | -0.44 | ac.y | ac.y.uv.Mod2 |
| SlopeOnCohort | -0.05 | 0.04 | 0.263 | -0.08 | ac.y | ac.y.uv.Mod2 |
| InterceptOfIntercept | 3.14 | 0.03 | 0.000 | 4.62 | ac.p | ac.p.uv.Mod0 |
| VarianceOfIntercept | 0.46 | 0.03 | 0.000 | 1.00 | ac.p | ac.p.uv.Mod0 |
| InterceptOfIntercept | 3.13 | 0.03 | 0.000 | 4.50 | ac.p | ac.p.uv.Mod1 |
| VarianceOfIntercept | 0.48 | 0.04 | 0.000 | 1.00 | ac.p | ac.p.uv.Mod1 |
| InterceptOfSlope | 0.01 | 0.03 | 0.677 | 0.02 | ac.p | ac.p.uv.Mod1 |
| VarianceOfSlope | 0.23 | 0.06 | 0.000 | 1.00 | ac.p | ac.p.uv.Mod1 |
| InterceptSlopeCorr | -0.06 | 0.04 | 0.127 | -0.18 | ac.p | ac.p.uv.Mod1 |
| InterceptOfIntercept | 3.15 | 0.05 | 0.000 | 4.53 | ac.p | ac.p.uv.Mod2 |
| VarianceOfIntercept | 0.48 | 0.04 | 0.000 | 1.00 | ac.p | ac.p.uv.Mod2 |
| InterceptOfSlope | 0.02 | 0.05 | 0.719 | 0.03 | ac.p | ac.p.uv.Mod2 |
| VarianceOfSlope | 0.23 | 0.07 | 0.000 | 1.00 | ac.p | ac.p.uv.Mod2 |
| InterceptSlopeCorr | -0.06 | 0.04 | 0.118 | -0.18 | ac.p | ac.p.uv.Mod2 |
| InterceptOnCohort | -0.02 | 0.04 | 0.563 | -0.02 | ac.p | ac.p.uv.Mod2 |
| SlopeOnCohort | 0.00 | 0.04 | 0.898 | -0.01 | ac.p | ac.p.uv.Mod2 |
| InterceptOfIntercept | 3.49 | 0.02 | 0.000 | 8.44 | at.y | at.y.uv.Mod0 |
| VarianceOfIntercept | 0.17 | 0.01 | 0.000 | 1.00 | at.y | at.y.uv.Mod0 |
| InterceptOfIntercept | 3.41 | 0.02 | 0.000 | 7.44 | at.y | at.y.uv.Mod1 |
| VarianceOfIntercept | 0.21 | 0.03 | 0.000 | 1.00 | at.y | at.y.uv.Mod1 |
| InterceptOfSlope | 0.13 | 0.03 | 0.000 | 0.25 | at.y | at.y.uv.Mod1 |
| VarianceOfSlope | 0.28 | 0.06 | 0.000 | 1.00 | at.y | at.y.uv.Mod1 |
| InterceptSlopeCorr | -0.10 | 0.03 | 0.003 | -0.39 | at.y | at.y.uv.Mod1 |
| InterceptOfIntercept | 3.48 | 0.04 | 0.000 | 7.68 | at.y | at.y.uv.Mod2 |
| VarianceOfIntercept | 0.20 | 0.03 | 0.000 | 0.98 | at.y | at.y.uv.Mod2 |
| InterceptOfSlope | 0.16 | 0.05 | 0.001 | 0.32 | at.y | at.y.uv.Mod2 |
| VarianceOfSlope | 0.27 | 0.06 | 0.000 | 1.00 | at.y | at.y.uv.Mod2 |
| InterceptSlopeCorr | -0.09 | 0.03 | 0.005 | -0.39 | at.y | at.y.uv.Mod2 |
| InterceptOnCohort | -0.07 | 0.03 | 0.014 | -0.13 | at.y | at.y.uv.Mod2 |
| SlopeOnCohort | -0.03 | 0.04 | 0.464 | -0.04 | at.y | at.y.uv.Mod2 |
| InterceptOfIntercept | 3.12 | 0.02 | 0.000 | 6.42 | at.p | at.p.uv.Mod0 |
| VarianceOfIntercept | 0.24 | 0.02 | 0.000 | 1.00 | at.p | at.p.uv.Mod0 |
| InterceptOfIntercept | 3.06 | 0.02 | 0.000 | 5.82 | at.p | at.p.uv.Mod1 |
| VarianceOfIntercept | 0.28 | 0.03 | 0.000 | 1.00 | at.p | at.p.uv.Mod1 |
| InterceptOfSlope | 0.12 | 0.02 | 0.000 | 0.35 | at.p | at.p.uv.Mod1 |
| VarianceOfSlope | 0.12 | 0.04 | 0.006 | 1.00 | at.p | at.p.uv.Mod1 |
| InterceptSlopeCorr | -0.06 | 0.03 | 0.019 | -0.33 | at.p | at.p.uv.Mod1 |
| InterceptOfIntercept | 2.91 | 0.04 | 0.000 | 5.59 | at.p | at.p.uv.Mod2 |
| VarianceOfIntercept | 0.26 | 0.03 | 0.000 | 0.95 | at.p | at.p.uv.Mod2 |
| InterceptOfSlope | 0.15 | 0.04 | 0.000 | 0.45 | at.p | at.p.uv.Mod2 |
| VarianceOfSlope | 0.10 | 0.04 | 0.014 | 1.00 | at.p | at.p.uv.Mod2 |
| InterceptSlopeCorr | -0.05 | 0.02 | 0.042 | -0.31 | at.p | at.p.uv.Mod2 |
| InterceptOnCohort | 0.15 | 0.03 | 0.000 | 0.22 | at.p | at.p.uv.Mod2 |
| SlopeOnCohort | -0.03 | 0.03 | 0.356 | -0.06 | at.p | at.p.uv.Mod2 |
| InterceptOfIntercept | 3.78 | 0.02 | 0.000 | 9.39 | ic.y | ic.y.uv.Mod0 |
| VarianceOfIntercept | 0.16 | 0.01 | 0.000 | 1.00 | ic.y | ic.y.uv.Mod0 |
| InterceptOfIntercept | 3.66 | 0.02 | 0.000 | 8.71 | ic.y | ic.y.uv.Mod1 |
| VarianceOfIntercept | 0.18 | 0.03 | 0.000 | 1.00 | ic.y | ic.y.uv.Mod1 |
| InterceptOfSlope | 0.27 | 0.03 | 0.000 | 1.01 | ic.y | ic.y.uv.Mod1 |
| VarianceOfSlope | 0.07 | 0.06 | 0.248 | 1.00 | ic.y | ic.y.uv.Mod1 |
| InterceptSlopeCorr | -0.02 | 0.03 | 0.497 | -0.21 | ic.y | ic.y.uv.Mod1 |
| InterceptOfIntercept | 3.72 | 0.04 | 0.000 | 8.94 | ic.y | ic.y.uv.Mod2 |
| VarianceOfIntercept | 0.17 | 0.03 | 0.000 | 0.99 | ic.y | ic.y.uv.Mod2 |
| InterceptOfSlope | 0.28 | 0.05 | 0.000 | 1.12 | ic.y | ic.y.uv.Mod2 |
| VarianceOfSlope | 0.06 | 0.06 | 0.321 | 1.00 | ic.y | ic.y.uv.Mod2 |
| InterceptSlopeCorr | -0.02 | 0.04 | 0.580 | -0.19 | ic.y | ic.y.uv.Mod2 |
| InterceptOnCohort | -0.06 | 0.03 | 0.035 | -0.12 | ic.y | ic.y.uv.Mod2 |
| SlopeOnCohort | -0.01 | 0.04 | 0.809 | -0.03 | ic.y | ic.y.uv.Mod2 |
| InterceptOfIntercept | 3.79 | 0.02 | 0.000 | 8.29 | ic.p | ic.p.uv.Mod0 |
| VarianceOfIntercept | 0.21 | 0.02 | 0.000 | 1.00 | ic.p | ic.p.uv.Mod0 |
| InterceptOfIntercept | 3.66 | 0.02 | 0.000 | 7.50 | ic.p | ic.p.uv.Mod1 |
| VarianceOfIntercept | 0.24 | 0.02 | 0.000 | 1.00 | ic.p | ic.p.uv.Mod1 |
| InterceptOfSlope | 0.25 | 0.03 | 0.000 | 0.60 | ic.p | ic.p.uv.Mod1 |
| VarianceOfSlope | 0.17 | 0.05 | 0.000 | 1.00 | ic.p | ic.p.uv.Mod1 |
| InterceptSlopeCorr | -0.06 | 0.03 | 0.022 | -0.30 | ic.p | ic.p.uv.Mod1 |
| InterceptOfIntercept | 3.51 | 0.04 | 0.000 | 7.15 | ic.p | ic.p.uv.Mod2 |
| VarianceOfIntercept | 0.23 | 0.02 | 0.000 | 0.94 | ic.p | ic.p.uv.Mod2 |
| InterceptOfSlope | 0.32 | 0.04 | 0.000 | 0.77 | ic.p | ic.p.uv.Mod2 |
| VarianceOfSlope | 0.17 | 0.04 | 0.000 | 0.98 | ic.p | ic.p.uv.Mod2 |
| InterceptSlopeCorr | -0.06 | 0.03 | 0.026 | -0.29 | ic.p | ic.p.uv.Mod2 |
| InterceptOnCohort | 0.14 | 0.03 | 0.000 | 0.24 | ic.p | ic.p.uv.Mod2 |
| SlopeOnCohort | -0.07 | 0.03 | 0.019 | -0.14 | ic.p | ic.p.uv.Mod2 |
| InterceptOfIntercept | 3.56 | 0.02 | 0.000 | 10.31 | ne.y | ne.y.uv.Mod0 |
| VarianceOfIntercept | 0.12 | 0.01 | 0.000 | 1.00 | ne.y | ne.y.uv.Mod0 |
| InterceptOfIntercept | 3.38 | 0.02 | 0.000 | 8.61 | ne.y | ne.y.uv.Mod1 |
| VarianceOfIntercept | 0.15 | 0.02 | 0.000 | 1.00 | ne.y | ne.y.uv.Mod1 |
| InterceptOfSlope | 0.28 | 0.03 | 0.000 | 0.71 | ne.y | ne.y.uv.Mod1 |
| VarianceOfSlope | 0.16 | 0.04 | 0.000 | 1.00 | ne.y | ne.y.uv.Mod1 |
| InterceptSlopeCorr | -0.07 | 0.02 | 0.007 | -0.42 | ne.y | ne.y.uv.Mod1 |
| InterceptOfIntercept | 3.38 | 0.03 | 0.000 | 8.59 | ne.y | ne.y.uv.Mod2 |
| VarianceOfIntercept | 0.15 | 0.02 | 0.000 | 1.00 | ne.y | ne.y.uv.Mod2 |
| InterceptOfSlope | 0.37 | 0.04 | 0.000 | 0.96 | ne.y | ne.y.uv.Mod2 |
| VarianceOfSlope | 0.14 | 0.04 | 0.000 | 0.97 | ne.y | ne.y.uv.Mod2 |
| InterceptSlopeCorr | -0.06 | 0.02 | 0.008 | -0.43 | ne.y | ne.y.uv.Mod2 |
| InterceptOnCohort | 0.01 | 0.03 | 0.824 | 0.01 | ne.y | ne.y.uv.Mod2 |
| SlopeOnCohort | -0.08 | 0.03 | 0.008 | -0.17 | ne.y | ne.y.uv.Mod2 |
| InterceptOfIntercept | 3.59 | 0.02 | 0.000 | 8.21 | ne.p | ne.p.uv.Mod0 |
| VarianceOfIntercept | 0.19 | 0.01 | 0.000 | 1.00 | ne.p | ne.p.uv.Mod0 |
| InterceptOfIntercept | 3.44 | 0.02 | 0.000 | 7.58 | ne.p | ne.p.uv.Mod1 |
| VarianceOfIntercept | 0.21 | 0.02 | 0.000 | 1.00 | ne.p | ne.p.uv.Mod1 |
| InterceptOfSlope | 0.24 | 0.02 | 0.000 | 0.64 | ne.p | ne.p.uv.Mod1 |
| VarianceOfSlope | 0.14 | 0.03 | 0.000 | 1.00 | ne.p | ne.p.uv.Mod1 |
| InterceptSlopeCorr | -0.04 | 0.02 | 0.005 | -0.26 | ne.p | ne.p.uv.Mod1 |
| InterceptOfIntercept | 3.34 | 0.03 | 0.000 | 7.32 | ne.p | ne.p.uv.Mod2 |
| VarianceOfIntercept | 0.20 | 0.02 | 0.000 | 0.97 | ne.p | ne.p.uv.Mod2 |
| InterceptOfSlope | 0.23 | 0.03 | 0.000 | 0.59 | ne.p | ne.p.uv.Mod2 |
| VarianceOfSlope | 0.15 | 0.03 | 0.000 | 1.00 | ne.p | ne.p.uv.Mod2 |
| InterceptSlopeCorr | -0.05 | 0.02 | 0.002 | -0.28 | ne.p | ne.p.uv.Mod2 |
| InterceptOnCohort | 0.10 | 0.02 | 0.000 | 0.17 | ne.p | ne.p.uv.Mod2 |
| SlopeOnCohort | 0.01 | 0.02 | 0.543 | 0.03 | ne.p | ne.p.uv.Mod2 |
| InterceptOfIntercept | 4.08 | 0.02 | 0.000 | 7.88 | ag.y | ag.y.uv.Mod0 |
| VarianceOfIntercept | 0.27 | 0.02 | 0.000 | 1.00 | ag.y | ag.y.uv.Mod0 |
| InterceptOfIntercept | 4.04 | 0.03 | 0.000 | 6.62 | ag.y | ag.y.uv.Mod1 |
| VarianceOfIntercept | 0.37 | 0.04 | 0.000 | 1.00 | ag.y | ag.y.uv.Mod1 |
| InterceptOfSlope | 0.07 | 0.03 | 0.034 | 0.13 | ag.y | ag.y.uv.Mod1 |
| VarianceOfSlope | 0.29 | 0.07 | 0.000 | 1.00 | ag.y | ag.y.uv.Mod1 |
| InterceptSlopeCorr | -0.16 | 0.04 | 0.000 | -0.50 | ag.y | ag.y.uv.Mod1 |
| InterceptOfIntercept | 4.25 | 0.04 | 0.000 | 7.19 | ag.y | ag.y.uv.Mod2 |
| VarianceOfIntercept | 0.32 | 0.04 | 0.000 | 0.92 | ag.y | ag.y.uv.Mod2 |
| InterceptOfSlope | 0.03 | 0.05 | 0.629 | 0.05 | ag.y | ag.y.uv.Mod2 |
| VarianceOfSlope | 0.24 | 0.07 | 0.001 | 1.00 | ag.y | ag.y.uv.Mod2 |
| InterceptSlopeCorr | -0.13 | 0.04 | 0.003 | -0.46 | ag.y | ag.y.uv.Mod2 |
| InterceptOnCohort | -0.20 | 0.03 | 0.000 | -0.28 | ag.y | ag.y.uv.Mod2 |
| SlopeOnCohort | 0.04 | 0.04 | 0.328 | 0.07 | ag.y | ag.y.uv.Mod2 |
| InterceptOfIntercept | 3.77 | 0.02 | 0.000 | 6.59 | ag.p | ag.p.uv.Mod0 |
| VarianceOfIntercept | 0.33 | 0.02 | 0.000 | 1.00 | ag.p | ag.p.uv.Mod0 |
| InterceptOfIntercept | 3.65 | 0.03 | 0.000 | 5.77 | ag.p | ag.p.uv.Mod1 |
| VarianceOfIntercept | 0.40 | 0.03 | 0.000 | 1.00 | ag.p | ag.p.uv.Mod1 |
| InterceptOfSlope | 0.21 | 0.02 | 0.000 | 0.46 | ag.p | ag.p.uv.Mod1 |
| VarianceOfSlope | 0.21 | 0.05 | 0.000 | 1.00 | ag.p | ag.p.uv.Mod1 |
| InterceptSlopeCorr | -0.11 | 0.03 | 0.001 | -0.38 | ag.p | ag.p.uv.Mod1 |
| InterceptOfIntercept | 3.59 | 0.04 | 0.000 | 5.67 | ag.p | ag.p.uv.Mod2 |
| VarianceOfIntercept | 0.40 | 0.03 | 0.000 | 0.99 | ag.p | ag.p.uv.Mod2 |
| InterceptOfSlope | 0.19 | 0.04 | 0.000 | 0.41 | ag.p | ag.p.uv.Mod2 |
| VarianceOfSlope | 0.21 | 0.05 | 0.000 | 1.00 | ag.p | ag.p.uv.Mod2 |
| InterceptSlopeCorr | -0.11 | 0.03 | 0.000 | -0.39 | ag.p | ag.p.uv.Mod2 |
| InterceptOnCohort | 0.06 | 0.03 | 0.091 | 0.07 | ag.p | ag.p.uv.Mod2 |
| SlopeOnCohort | 0.02 | 0.03 | 0.464 | 0.04 | ag.p | ag.p.uv.Mod2 |
| InterceptOfIntercept | 3.69 | 0.03 | 0.000 | 7.76 | fear.y | fear.y.uv.Mod0 |
| VarianceOfIntercept | 0.23 | 0.02 | 0.000 | 1.00 | fear.y | fear.y.uv.Mod0 |
| InterceptOfIntercept | 3.27 | 0.03 | 0.000 | 5.90 | fear.y | fear.y.uv.Mod1 |
| VarianceOfIntercept | 0.31 | 0.05 | 0.000 | 1.00 | fear.y | fear.y.uv.Mod1 |
| InterceptOfSlope | 0.67 | 0.04 | 0.000 | 1.68 | fear.y | fear.y.uv.Mod1 |
| VarianceOfSlope | 0.16 | 0.09 | 0.065 | 1.00 | fear.y | fear.y.uv.Mod1 |
| InterceptSlopeCorr | -0.10 | 0.05 | 0.052 | -0.46 | fear.y | fear.y.uv.Mod1 |
| InterceptOfIntercept | 3.04 | 0.05 | 0.000 | 5.29 | fear.y | fear.y.uv.Mod2 |
| VarianceOfIntercept | 0.30 | 0.05 | 0.000 | 0.91 | fear.y | fear.y.uv.Mod2 |
| InterceptOfSlope | 0.83 | 0.06 | 0.000 | 1.87 | fear.y | fear.y.uv.Mod2 |
| VarianceOfSlope | 0.18 | 0.08 | 0.031 | 0.93 | fear.y | fear.y.uv.Mod2 |
| InterceptSlopeCorr | -0.11 | 0.05 | 0.037 | -0.46 | fear.y | fear.y.uv.Mod2 |
| InterceptOnCohort | 0.22 | 0.04 | 0.000 | 0.31 | fear.y | fear.y.uv.Mod2 |
| SlopeOnCohort | -0.15 | 0.04 | 0.001 | -0.27 | fear.y | fear.y.uv.Mod2 |
| InterceptOfIntercept | 3.79 | 0.03 | 0.000 | 6.88 | fear.p | fear.p.uv.Mod0 |
| VarianceOfIntercept | 0.30 | 0.02 | 0.000 | 1.00 | fear.p | fear.p.uv.Mod0 |
| InterceptOfIntercept | 3.55 | 0.03 | 0.000 | 6.02 | fear.p | fear.p.uv.Mod1 |
| VarianceOfIntercept | 0.35 | 0.03 | 0.000 | 1.00 | fear.p | fear.p.uv.Mod1 |
| InterceptOfSlope | 0.43 | 0.03 | 0.000 | 1.19 | fear.p | fear.p.uv.Mod1 |
| VarianceOfSlope | 0.13 | 0.06 | 0.027 | 1.00 | fear.p | fear.p.uv.Mod1 |
| InterceptSlopeCorr | -0.07 | 0.04 | 0.048 | -0.33 | fear.p | fear.p.uv.Mod1 |
| InterceptOfIntercept | 3.34 | 0.04 | 0.000 | 5.70 | fear.p | fear.p.uv.Mod2 |
| VarianceOfIntercept | 0.32 | 0.03 | 0.000 | 0.92 | fear.p | fear.p.uv.Mod2 |
| InterceptOfSlope | 0.46 | 0.04 | 0.000 | 1.32 | fear.p | fear.p.uv.Mod2 |
| VarianceOfSlope | 0.12 | 0.06 | 0.040 | 1.00 | fear.p | fear.p.uv.Mod2 |
| InterceptSlopeCorr | -0.06 | 0.04 | 0.077 | -0.32 | fear.p | fear.p.uv.Mod2 |
| InterceptOnCohort | 0.21 | 0.03 | 0.000 | 0.28 | fear.p | fear.p.uv.Mod2 |
| SlopeOnCohort | -0.03 | 0.03 | 0.430 | -0.06 | fear.p | fear.p.uv.Mod2 |
| InterceptOfIntercept | 2.99 | 0.02 | 0.000 | 6.53 | fr.y | fr.y.uv.Mod0 |
| VarianceOfIntercept | 0.21 | 0.02 | 0.000 | 1.00 | fr.y | fr.y.uv.Mod0 |
| InterceptOfIntercept | 2.86 | 0.03 | 0.000 | 5.45 | fr.y | fr.y.uv.Mod1 |
| VarianceOfIntercept | 0.28 | 0.04 | 0.000 | 1.00 | fr.y | fr.y.uv.Mod1 |
| InterceptOfSlope | 0.23 | 0.04 | 0.000 | 0.46 | fr.y | fr.y.uv.Mod1 |
| VarianceOfSlope | 0.26 | 0.08 | 0.002 | 1.00 | fr.y | fr.y.uv.Mod1 |
| InterceptSlopeCorr | -0.11 | 0.05 | 0.019 | -0.42 | fr.y | fr.y.uv.Mod1 |
| InterceptOfIntercept | 2.92 | 0.04 | 0.000 | 5.60 | fr.y | fr.y.uv.Mod2 |
| VarianceOfIntercept | 0.27 | 0.04 | 0.000 | 0.99 | fr.y | fr.y.uv.Mod2 |
| InterceptOfSlope | 0.30 | 0.06 | 0.000 | 0.63 | fr.y | fr.y.uv.Mod2 |
| VarianceOfSlope | 0.23 | 0.08 | 0.004 | 0.99 | fr.y | fr.y.uv.Mod2 |
| InterceptSlopeCorr | -0.11 | 0.05 | 0.025 | -0.42 | fr.y | fr.y.uv.Mod2 |
| InterceptOnCohort | -0.05 | 0.03 | 0.108 | -0.08 | fr.y | fr.y.uv.Mod2 |
| SlopeOnCohort | -0.07 | 0.04 | 0.105 | -0.12 | fr.y | fr.y.uv.Mod2 |
| InterceptOfIntercept | 3.20 | 0.02 | 0.000 | 5.84 | fr.p | fr.p.uv.Mod0 |
| VarianceOfIntercept | 0.30 | 0.02 | 0.000 | 1.00 | fr.p | fr.p.uv.Mod0 |
| InterceptOfIntercept | 3.09 | 0.03 | 0.000 | 5.31 | fr.p | fr.p.uv.Mod1 |
| VarianceOfIntercept | 0.34 | 0.03 | 0.000 | 1.00 | fr.p | fr.p.uv.Mod1 |
| InterceptOfSlope | 0.23 | 0.03 | 0.000 | 0.54 | fr.p | fr.p.uv.Mod1 |
| VarianceOfSlope | 0.18 | 0.06 | 0.002 | 1.00 | fr.p | fr.p.uv.Mod1 |
| InterceptSlopeCorr | -0.06 | 0.04 | 0.067 | -0.26 | fr.p | fr.p.uv.Mod1 |
| InterceptOfIntercept | 3.04 | 0.04 | 0.000 | 5.22 | fr.p | fr.p.uv.Mod2 |
| VarianceOfIntercept | 0.34 | 0.03 | 0.000 | 1.00 | fr.p | fr.p.uv.Mod2 |
| InterceptOfSlope | 0.15 | 0.04 | 0.000 | 0.35 | fr.p | fr.p.uv.Mod2 |
| VarianceOfSlope | 0.19 | 0.06 | 0.002 | 0.98 | fr.p | fr.p.uv.Mod2 |
| InterceptSlopeCorr | -0.07 | 0.04 | 0.053 | -0.27 | fr.p | fr.p.uv.Mod2 |
| InterceptOnCohort | 0.05 | 0.03 | 0.155 | 0.06 | fr.p | fr.p.uv.Mod2 |
| SlopeOnCohort | 0.08 | 0.03 | 0.026 | 0.14 | fr.p | fr.p.uv.Mod2 |
| InterceptOfIntercept | 3.58 | 0.03 | 0.000 | 5.13 | shy.y | shy.y.uv.Mod0 |
| VarianceOfIntercept | 0.49 | 0.04 | 0.000 | 1.00 | shy.y | shy.y.uv.Mod0 |
| InterceptOfIntercept | 3.51 | 0.04 | 0.000 | 5.36 | shy.y | shy.y.uv.Mod1 |
| VarianceOfIntercept | 0.43 | 0.07 | 0.000 | 1.00 | shy.y | shy.y.uv.Mod1 |
| InterceptOfSlope | 0.12 | 0.05 | 0.006 | 0.30 | shy.y | shy.y.uv.Mod1 |
| VarianceOfSlope | 0.17 | 0.14 | 0.221 | 1.00 | shy.y | shy.y.uv.Mod1 |
| InterceptSlopeCorr | 0.02 | 0.08 | 0.812 | 0.07 | shy.y | shy.y.uv.Mod1 |
| InterceptOfIntercept | 3.41 | 0.06 | 0.000 | 5.17 | shy.y | shy.y.uv.Mod2 |
| VarianceOfIntercept | 0.43 | 0.07 | 0.000 | 0.99 | shy.y | shy.y.uv.Mod2 |
| InterceptOfSlope | 0.35 | 0.07 | 0.000 | 0.85 | shy.y | shy.y.uv.Mod2 |
| VarianceOfSlope | 0.14 | 0.14 | 0.333 | 0.82 | shy.y | shy.y.uv.Mod2 |
| InterceptSlopeCorr | 0.03 | 0.08 | 0.715 | 0.12 | shy.y | shy.y.uv.Mod2 |
| InterceptOnCohort | 0.10 | 0.05 | 0.031 | 0.12 | shy.y | shy.y.uv.Mod2 |
| SlopeOnCohort | -0.22 | 0.06 | 0.000 | -0.43 | shy.y | shy.y.uv.Mod2 |
| InterceptOfIntercept | 3.53 | 0.03 | 0.000 | 4.64 | shy.p | shy.p.uv.Mod0 |
| VarianceOfIntercept | 0.58 | 0.04 | 0.000 | 1.00 | shy.p | shy.p.uv.Mod0 |
| InterceptOfIntercept | 3.49 | 0.03 | 0.000 | 4.49 | shy.p | shy.p.uv.Mod1 |
| VarianceOfIntercept | 0.60 | 0.05 | 0.000 | 1.00 | shy.p | shy.p.uv.Mod1 |
| InterceptOfSlope | 0.08 | 0.03 | 0.007 | 0.18 | shy.p | shy.p.uv.Mod1 |
| VarianceOfSlope | 0.20 | 0.08 | 0.014 | 1.00 | shy.p | shy.p.uv.Mod1 |
| InterceptSlopeCorr | -0.06 | 0.05 | 0.201 | -0.18 | shy.p | shy.p.uv.Mod1 |
| InterceptOfIntercept | 3.42 | 0.05 | 0.000 | 4.39 | shy.p | shy.p.uv.Mod2 |
| VarianceOfIntercept | 0.60 | 0.05 | 0.000 | 0.99 | shy.p | shy.p.uv.Mod2 |
| InterceptOfSlope | 0.11 | 0.05 | 0.032 | 0.23 | shy.p | shy.p.uv.Mod2 |
| VarianceOfSlope | 0.21 | 0.08 | 0.012 | 1.00 | shy.p | shy.p.uv.Mod2 |
| InterceptSlopeCorr | -0.06 | 0.05 | 0.184 | -0.18 | shy.p | shy.p.uv.Mod2 |
| InterceptOnCohort | 0.07 | 0.04 | 0.079 | 0.08 | shy.p | shy.p.uv.Mod2 |
| SlopeOnCohort | -0.02 | 0.04 | 0.539 | -0.04 | shy.p | shy.p.uv.Mod2 |
| InterceptOfIntercept | 1.60 | 0.01 | 0.000 | 5.54 | adv | adv.uv.Mod0 |
| VarianceOfIntercept | 0.08 | 0.01 | 0.000 | 1.00 | adv | adv.uv.Mod0 |
| InterceptOfIntercept | 1.63 | 0.01 | 0.000 | 4.97 | adv | adv.uv.Mod1 |
| VarianceOfIntercept | 0.11 | 0.01 | 0.000 | 1.00 | adv | adv.uv.Mod1 |
| InterceptOfSlope | -0.10 | 0.02 | 0.000 | -0.35 | adv | adv.uv.Mod1 |
| VarianceOfSlope | 0.08 | 0.02 | 0.000 | 1.00 | adv | adv.uv.Mod1 |
| InterceptSlopeCorr | -0.04 | 0.01 | 0.002 | -0.46 | adv | adv.uv.Mod1 |
| InterceptOfIntercept | 1.50 | 0.02 | 0.000 | 4.64 | adv | adv.uv.Mod2 |
| VarianceOfIntercept | 0.09 | 0.01 | 0.000 | 0.89 | adv | adv.uv.Mod2 |
| InterceptOfSlope | -0.03 | 0.03 | 0.365 | -0.10 | adv | adv.uv.Mod2 |
| VarianceOfSlope | 0.06 | 0.02 | 0.002 | 0.96 | adv | adv.uv.Mod2 |
| InterceptSlopeCorr | -0.03 | 0.01 | 0.017 | -0.39 | adv | adv.uv.Mod2 |
| InterceptOnCohort | 0.13 | 0.02 | 0.000 | 0.32 | adv | adv.uv.Mod2 |
| SlopeOnCohort | -0.07 | 0.02 | 0.002 | -0.21 | adv | adv.uv.Mod2 |
| InterceptOfIntercept | 2.81 | 0.05 | 0.000 | 2.81 | avoid.y | avoid.uv.Mod0 |
| VarianceOfIntercept | 1.00 | 0.08 | 0.000 | 1.00 | avoid.y | avoid.uv.Mod0 |
| InterceptOfIntercept | 2.72 | 0.05 | 0.000 | 2.59 | avoid.y | avoid.uv.Mod1 |
| VarianceOfIntercept | 1.10 | 0.13 | 0.000 | 1.00 | avoid.y | avoid.uv.Mod1 |
| InterceptOfSlope | 0.21 | 0.06 | 0.001 | 0.18 | avoid.y | avoid.uv.Mod1 |
| VarianceOfSlope | 1.39 | 0.27 | 0.000 | 1.00 | avoid.y | avoid.uv.Mod1 |
| InterceptSlopeCorr | -0.32 | 0.15 | 0.031 | -0.26 | avoid.y | avoid.uv.Mod1 |
| InterceptOfIntercept | 2.45 | 0.08 | 0.000 | 2.37 | avoid.y | avoid.uv.Mod2 |
| VarianceOfIntercept | 1.02 | 0.13 | 0.000 | 0.96 | avoid.y | avoid.uv.Mod2 |
| InterceptOfSlope | 0.19 | 0.11 | 0.076 | 0.17 | avoid.y | avoid.uv.Mod2 |
| VarianceOfSlope | 1.31 | 0.27 | 0.000 | 1.00 | avoid.y | avoid.uv.Mod2 |
| InterceptSlopeCorr | -0.28 | 0.15 | 0.055 | -0.25 | avoid.y | avoid.uv.Mod2 |
| InterceptOnCohort | 0.26 | 0.06 | 0.000 | 0.20 | avoid.y | avoid.uv.Mod2 |
| SlopeOnCohort | 0.02 | 0.08 | 0.782 | 0.02 | avoid.y | avoid.uv.Mod2 |
| InterceptOfIntercept | 1.62 | 0.03 | 0.000 | 2.67 | anx.y | anx.uv.Mod0 |
| VarianceOfIntercept | 0.37 | 0.04 | 0.000 | 1.00 | anx.y | anx.uv.Mod0 |
| InterceptOfIntercept | 1.65 | 0.04 | 0.000 | 2.89 | anx.y | anx.uv.Mod1 |
| VarianceOfIntercept | 0.32 | 0.08 | 0.000 | 1.00 | anx.y | anx.uv.Mod1 |
| InterceptOfSlope | -0.06 | 0.06 | 0.291 | -0.08 | anx.y | anx.uv.Mod1 |
| VarianceOfSlope | 0.50 | 0.19 | 0.009 | 1.00 | anx.y | anx.uv.Mod1 |
| InterceptSlopeCorr | -0.06 | 0.10 | 0.576 | -0.14 | anx.y | anx.uv.Mod1 |
| InterceptOfIntercept | 1.71 | 0.06 | 0.000 | 2.99 | anx.y | anx.uv.Mod2 |
| VarianceOfIntercept | 0.32 | 0.08 | 0.000 | 0.99 | anx.y | anx.uv.Mod2 |
| InterceptOfSlope | -0.21 | 0.09 | 0.023 | -0.30 | anx.y | anx.uv.Mod2 |
| VarianceOfSlope | 0.48 | 0.19 | 0.013 | 0.97 | anx.y | anx.uv.Mod2 |
| InterceptSlopeCorr | -0.05 | 0.10 | 0.623 | -0.13 | anx.y | anx.uv.Mod2 |
| InterceptOnCohort | -0.06 | 0.05 | 0.232 | -0.08 | anx.y | anx.uv.Mod2 |
| SlopeOnCohort | 0.15 | 0.07 | 0.040 | 0.17 | anx.y | anx.uv.Mod2 |

*Note:* Mod0 = no growth model; Mod1 = unconditional, univariate latent growth curve model; Mod2 = univariate latent growth curve model conditioned on cohort.

# Table S7. Bivariate Latent Growth Curve Model Fit

| chisq | df | rmsea | tli | cfi | aic | bic | ModID |
| --- | --- | --- | --- | --- | --- | --- | --- |
| 48.251 | 7 | 0.093 | 0.92 | 0.963 | 3429.858 | 3520.359 | ec.y_bv |
| 56.236 | 9 | 0.088 | 0.908 | 0.961 | 3348.667 | 3457.267 | ec.y_bv_onC |
| 34.702 | 7 | 0.076 | 0.962 | 0.982 | 3068.737 | 3159.179 | ec.p_bv |
| 40.641 | 9 | 0.072 | 0.955 | 0.981 | 2994.378 | 3102.979 | ec.p_bv_onC |
| 27.401 | 7 | 0.065 | 0.958 | 0.98 | 4965.204 | 5055.704 | ac.y_bv |
| 30.066 | 9 | 0.059 | 0.959 | 0.982 | 4813.032 | 4921.632 | ac.y_bv_onC |
| 25.986 | 7 | 0.063 | 0.971 | 0.987 | 4357.153 | 4447.595 | ac.p_bv |
| 33.779 | 9 | 0.064 | 0.961 | 0.983 | 4304.429 | 4413.03 | ac.p_bv_onC |
| 30.363 | 7 | 0.07 | 0.941 | 0.972 | 4015.75 | 4106.251 | at.y_bv |
| 38.968 | 9 | 0.07 | 0.923 | 0.967 | 3962.11 | 4070.71 | at.y_bv_onC |
| 14.071 | 7 | 0.039 | 0.987 | 0.994 | 3622.215 | 3712.656 | at.p_bv |
| 19.586 | 9 | 0.042 | 0.981 | 0.992 | 3522.198 | 3630.799 | at.p_bv_onC |
| 34.407 | 7 | 0.076 | 0.925 | 0.965 | 4099.787 | 4190.287 | ic.y_bv |
| 40.892 | 9 | 0.072 | 0.911 | 0.962 | 4047.646 | 4156.247 | ic.y_bv_onC |
| 28.012 | 7 | 0.066 | 0.956 | 0.98 | 3718.582 | 3809.024 | ic.p_bv |
| 30.386 | 9 | 0.059 | 0.956 | 0.981 | 3624.413 | 3733.014 | ic.p_bv_onC |
| 81.704 | 7 | 0.125 | 0.806 | 0.909 | 3419.016 | 3509.517 | ne.y_bv |
| 101.622 | 9 | 0.123 | 0.761 | 0.897 | 3362.295 | 3470.895 | ne.y_bv_onC |
| 67.611 | 7 | 0.113 | 0.905 | 0.956 | 2881.304 | 2971.746 | ne.p_bv |
| 71.125 | 9 | 0.101 | 0.901 | 0.958 | 2795.069 | 2903.67 | ne.p_bv_onC |
| 20.514 | 7 | 0.053 | 0.969 | 0.986 | 4402.816 | 4493.317 | ag.y_bv |
| 39.045 | 9 | 0.07 | 0.932 | 0.971 | 4333.294 | 4441.895 | ag.y_bv_onC |
| 36.384 | 7 | 0.079 | 0.95 | 0.977 | 3999.846 | 4090.288 | ag.p_bv |
| 39.935 | 9 | 0.071 | 0.946 | 0.977 | 3931.668 | 4040.269 | ag.p_bv_onC |
| 79.42 | 7 | 0.123 | 0.792 | 0.903 | 4769.56 | 4860.06 | fear.y_bv |
| 89.572 | 9 | 0.115 | 0.779 | 0.905 | 4677.793 | 4786.394 | fear.y_bv_onC |
| 56.23 | 7 | 0.102 | 0.904 | 0.955 | 4247.935 | 4338.377 | fear.p_bv |
| 59.737 | 9 | 0.091 | 0.902 | 0.958 | 4144.291 | 4252.892 | fear.p_bv_onC |
| 49.171 | 7 | 0.094 | 0.882 | 0.945 | 4625.723 | 4716.224 | fr.y_bv |
| 58.542 | 9 | 0.09 | 0.861 | 0.94 | 4570.282 | 4678.883 | fr.y_bv_onC |
| 38.459 | 7 | 0.081 | 0.939 | 0.972 | 4211.236 | 4301.677 | fr.p_bv |
| 41.407 | 9 | 0.073 | 0.936 | 0.973 | 4137.742 | 4246.342 | fr.p_bv_onC |
| 43.083 | 7 | 0.087 | 0.91 | 0.958 | 5657.713 | 5748.214 | shy.y_bv |
| 50.466 | 9 | 0.082 | 0.896 | 0.955 | 5594.838 | 5703.438 | shy.y_bv_onC |
| 15.623 | 7 | 0.043 | 0.986 | 0.994 | 4821.939 | 4912.381 | shy.p_bv |
| 19.045 | 9 | 0.04 | 0.984 | 0.993 | 4769.894 | 4878.495 | shy.p_bv_onC |

*Note:* The models with the “_bv” identifier attached at the end are unconditional, while the “_bv_onC” models are conditioned on cohort.

# Table S8a. Supplemental Slope-Slope and Intercept-Slope Correlations

| Parameter | Estimate | SE | PValue | StdAll | Construct | ModelName |
| --- | --- | --- | --- | --- | --- | --- |
| Slope_Corr_Adv_Intercept | 0.01 | 0.01 | 0.542 | 0.04 | ec.y | ec.y_bv_onC |
| Slope_Corr_Adv_Slope | -0.05 | 0.01 | 0.000 | -0.50 | ec.y | ec.y_bv_onC |
| Slope_Corr_Adv_Intercept | 0.00 | 0.01 | 0.834 | 0.01 | ec.p | ec.p_bv_onC |
| Slope_Corr_Adv_Slope | -0.03 | 0.01 | 0.003 | -0.31 | ec.p | ec.p_bv_onC |
| Slope_Corr_Adv_Intercept | 0.01 | 0.01 | 0.326 | 0.08 | ac.y | ac.y_bv_onC |
| Slope_Corr_Adv_Slope | -0.05 | 0.02 | 0.009 | -0.35 | ac.y | ac.y_bv_onC |
| Slope_Corr_Adv_Intercept | 0.01 | 0.01 | 0.581 | 0.04 | ac.p | ac.p_bv_onC |
| Slope_Corr_Adv_Slope | -0.04 | 0.01 | 0.008 | -0.33 | ac.p | ac.p_bv_onC |
| Slope_Corr_Adv_Intercept | 0.01 | 0.01 | 0.258 | 0.08 | at.y | at.y_bv_onC |
| Slope_Corr_Adv_Slope | -0.06 | 0.01 | 0.000 | -0.47 | at.y | at.y_bv_onC |
| Slope_Corr_Adv_Intercept | 0.00 | 0.01 | 0.609 | 0.04 | at.p | at.p_bv_onC |
| Slope_Corr_Adv_Slope | -0.02 | 0.01 | 0.177 | -0.19 | at.p | at.p_bv_onC |
| Slope_Corr_Adv_Intercept | -0.01 | 0.01 | 0.233 | -0.18 | ic.y | ic.y_bv_onC |
| Slope_Corr_Adv_Slope | -0.04 | 0.02 | 0.020 | -0.59 | ic.y | ic.y_bv_onC |
| Slope_Corr_Adv_Intercept | -0.01 | 0.01 | 0.372 | -0.06 | ic.p | ic.p_bv_onC |
| Slope_Corr_Adv_Slope | -0.04 | 0.01 | 0.005 | -0.34 | ic.p | ic.p_bv_onC |
| Slope_Corr_Adv_Intercept | 0.00 | 0.01 | 0.570 | 0.04 | ne.y | ne.y_bv_onC |
| Slope_Corr_Adv_Slope | -0.05 | 0.01 | 0.000 | -0.54 | ne.y | ne.y_bv_onC |
| Slope_Corr_Adv_Intercept | -0.01 | 0.01 | 0.194 | -0.07 | ne.p | ne.p_bv_onC |
| Slope_Corr_Adv_Slope | -0.02 | 0.01 | 0.064 | -0.18 | ne.p | ne.p_bv_onC |
| Slope_Corr_Adv_Intercept | 0.00 | 0.01 | 0.740 | 0.03 | ag.y | ag.y_bv_onC |
| Slope_Corr_Adv_Slope | -0.05 | 0.02 | 0.003 | -0.38 | ag.y | ag.y_bv_onC |
| Slope_Corr_Adv_Intercept | 0.00 | 0.01 | 0.737 | 0.02 | ag.p | ag.p_bv_onC |
| Slope_Corr_Adv_Slope | -0.04 | 0.01 | 0.002 | -0.32 | ag.p | ag.p_bv_onC |
| Slope_Corr_Adv_Intercept | 0.02 | 0.01 | 0.204 | 0.12 | fear.y | fear.y_bv_onC |
| Slope_Corr_Adv_Slope | -0.06 | 0.02 | 0.001 | -0.53 | fear.y | fear.y_bv_onC |
| Slope_Corr_Adv_Intercept | 0.00 | 0.01 | 0.701 | -0.04 | fear.p | fear.p_bv_onC |
| Slope_Corr_Adv_Slope | -0.02 | 0.01 | 0.254 | -0.18 | fear.p | fear.p_bv_onC |
| Slope_Corr_Adv_Intercept | 0.01 | 0.01 | 0.527 | 0.05 | fr.y | fr.y_bv_onC |
| Slope_Corr_Adv_Slope | -0.07 | 0.02 | 0.000 | -0.54 | fr.y | fr.y_bv_onC |
| Slope_Corr_Adv_Intercept | -0.01 | 0.01 | 0.417 | -0.06 | fr.p | fr.p_bv_onC |
| Slope_Corr_Adv_Slope | -0.04 | 0.01 | 0.007 | -0.35 | fr.p | fr.p_bv_onC |
| Slope_Corr_Adv_Intercept | -0.01 | 0.02 | 0.721 | -0.05 | shy.y | shy.y_bv_onC |
| Slope_Corr_Adv_Slope | -0.03 | 0.02 | 0.114 | -0.35 | shy.y | shy.y_bv_onC |
| Slope_Corr_Adv_Intercept | -0.03 | 0.01 | 0.005 | -0.22 | shy.p | shy.p_bv_onC |
| Slope_Corr_Adv_Slope | 0.03 | 0.02 | 0.037 | 0.27 | shy.p | shy.p_bv_onC |

# Table S8b. Full Output for Youth and Parent Report Models for Bivariate Growth in Facets and Adversity

| Parameter | Estimate | SE | PValue | StdAll | Construct | ModelName |
| --- | --- | --- | --- | --- | --- | --- |
| ConstructInterceptOfIntercept | 3.61 | 0.03 | 0.000 | 8.01 | ec.y | ec.y_bv_onC |
| ConstructVarianceOfIntercept | 0.19 | 0.02 | 0.000 | 0.92 | ec.y | ec.y_bv_onC |
| ConstructInterceptOfSlope | 0.11 | 0.04 | 0.006 | 0.28 | ec.y | ec.y_bv_onC |
| ConstructVarianceOfSlope | 0.14 | 0.04 | 0.000 | 1.00 | ec.y | ec.y_bv_onC |
| AdvInterceptOfIntercept | 1.50 | 0.02 | 0.000 | 4.62 | ec.y | ec.y_bv_onC |
| AdvVarianceOfIntercept | 0.09 | 0.01 | 0.000 | 0.89 | ec.y | ec.y_bv_onC |
| AdvInterceptOfSlope | -0.03 | 0.03 | 0.337 | -0.10 | ec.y | ec.y_bv_onC |
| AdvVarianceOfSlope | 0.07 | 0.02 | 0.001 | 0.96 | ec.y | ec.y_bv_onC |
| ConstructInterceptOnCohort | -0.16 | 0.03 | 0.000 | -0.29 | ec.y | ec.y_bv_onC |
| ConstructSlopeOnCohort | -0.02 | 0.03 | 0.505 | -0.04 | ec.y | ec.y_bv_onC |
| AdvInterceptOnCohort | 0.13 | 0.02 | 0.000 | 0.33 | ec.y | ec.y_bv_onC |
| AdvSlopeOnCohort | -0.07 | 0.02 | 0.002 | -0.21 | ec.y | ec.y_bv_onC |
| Cnsrct.Intercept_Corr_Adv_Intercept | -0.05 | 0.01 | 0.000 | -0.36 | ec.y | ec.y_bv_onC |
| Cnsrct.Intercept_Corr_Adv_Slope | 0.03 | 0.01 | 0.002 | 0.27 | ec.y | ec.y_bv_onC |
| Cnsrct.Slope_Corr_Adv_Intercept | 0.01 | 0.01 | 0.542 | 0.04 | ec.y | ec.y_bv_onC |
| Cnsrct.Slope_Corr_Adv_Slope | -0.05 | 0.01 | 0.000 | -0.50 | ec.y | ec.y_bv_onC |
| ConstructInterceptOfIntercept | 3.17 | 0.03 | 0.000 | 6.05 | ec.p | ec.p_bv_onC |
| ConstructVarianceOfIntercept | 0.27 | 0.02 | 0.000 | 0.99 | ec.p | ec.p_bv_onC |
| ConstructInterceptOfSlope | 0.13 | 0.03 | 0.000 | 0.36 | ec.p | ec.p_bv_onC |
| ConstructVarianceOfSlope | 0.14 | 0.03 | 0.000 | 1.00 | ec.p | ec.p_bv_onC |
| AdvInterceptOfIntercept | 1.50 | 0.02 | 0.000 | 4.65 | ec.p | ec.p_bv_onC |
| AdvVarianceOfIntercept | 0.09 | 0.01 | 0.000 | 0.89 | ec.p | ec.p_bv_onC |
| AdvInterceptOfSlope | -0.02 | 0.03 | 0.391 | -0.10 | ec.p | ec.p_bv_onC |
| AdvVarianceOfSlope | 0.06 | 0.02 | 0.002 | 0.95 | ec.p | ec.p_bv_onC |
| ConstructInterceptOnCohort | 0.08 | 0.03 | 0.003 | 0.12 | ec.p | ec.p_bv_onC |
| ConstructSlopeOnCohort | -0.03 | 0.02 | 0.255 | -0.06 | ec.p | ec.p_bv_onC |
| AdvInterceptOnCohort | 0.13 | 0.02 | 0.000 | 0.33 | ec.p | ec.p_bv_onC |
| AdvSlopeOnCohort | -0.07 | 0.02 | 0.002 | -0.22 | ec.p | ec.p_bv_onC |
| Cnsrct.Intercept_Corr_Adv_Intercept | -0.05 | 0.01 | 0.000 | -0.30 | ec.p | ec.p_bv_onC |
| Cnsrct.Intercept_Corr_Adv_Slope | 0.02 | 0.01 | 0.012 | 0.19 | ec.p | ec.p_bv_onC |
| Cnsrct.Slope_Corr_Adv_Intercept | 0.00 | 0.01 | 0.834 | 0.01 | ec.p | ec.p_bv_onC |
| Cnsrct.Slope_Corr_Adv_Slope | -0.03 | 0.01 | 0.003 | -0.31 | ec.p | ec.p_bv_onC |
| ConstructInterceptOfIntercept | 3.68 | 0.05 | 0.000 | 5.49 | ac.y | ac.y_bv_onC |
| ConstructVarianceOfIntercept | 0.36 | 0.05 | 0.000 | 0.80 | ac.y | ac.y_bv_onC |
| ConstructInterceptOfSlope | -0.09 | 0.06 | 0.118 | -0.18 | ac.y | ac.y_bv_onC |
| ConstructVarianceOfSlope | 0.26 | 0.09 | 0.003 | 1.00 | ac.y | ac.y_bv_onC |
| AdvInterceptOfIntercept | 1.50 | 0.02 | 0.000 | 4.62 | ac.y | ac.y_bv_onC |
| AdvVarianceOfIntercept | 0.09 | 0.01 | 0.000 | 0.89 | ac.y | ac.y_bv_onC |
| AdvInterceptOfSlope | -0.02 | 0.03 | 0.388 | -0.09 | ac.y | ac.y_bv_onC |
| AdvVarianceOfSlope | 0.07 | 0.02 | 0.001 | 0.95 | ac.y | ac.y_bv_onC |
| ConstructInterceptOnCohort | -0.38 | 0.04 | 0.000 | -0.45 | ac.y | ac.y_bv_onC |
| ConstructSlopeOnCohort | -0.04 | 0.04 | 0.331 | -0.07 | ac.y | ac.y_bv_onC |
| AdvInterceptOnCohort | 0.13 | 0.02 | 0.000 | 0.33 | ac.y | ac.y_bv_onC |
| AdvSlopeOnCohort | -0.07 | 0.02 | 0.002 | -0.21 | ac.y | ac.y_bv_onC |
| Cnsrct.Intercept_Corr_Adv_Intercept | -0.06 | 0.01 | 0.000 | -0.32 | ac.y | ac.y_bv_onC |
| Cnsrct.Intercept_Corr_Adv_Slope | 0.03 | 0.01 | 0.023 | 0.20 | ac.y | ac.y_bv_onC |
| Cnsrct.Slope_Corr_Adv_Intercept | 0.01 | 0.01 | 0.326 | 0.08 | ac.y | ac.y_bv_onC |
| Cnsrct.Slope_Corr_Adv_Slope | -0.05 | 0.02 | 0.009 | -0.35 | ac.y | ac.y_bv_onC |
| ConstructInterceptOfIntercept | 3.15 | 0.05 | 0.000 | 4.55 | ac.p | ac.p_bv_onC |
| ConstructVarianceOfIntercept | 0.48 | 0.04 | 0.000 | 1.00 | ac.p | ac.p_bv_onC |
| ConstructInterceptOfSlope | 0.01 | 0.05 | 0.875 | 0.02 | ac.p | ac.p_bv_onC |
| ConstructVarianceOfSlope | 0.21 | 0.06 | 0.001 | 1.00 | ac.p | ac.p_bv_onC |
| AdvInterceptOfIntercept | 1.50 | 0.02 | 0.000 | 4.66 | ac.p | ac.p_bv_onC |
| AdvVarianceOfIntercept | 0.09 | 0.01 | 0.000 | 0.89 | ac.p | ac.p_bv_onC |
| AdvInterceptOfSlope | -0.03 | 0.03 | 0.377 | -0.10 | ac.p | ac.p_bv_onC |
| AdvVarianceOfSlope | 0.06 | 0.02 | 0.003 | 0.95 | ac.p | ac.p_bv_onC |
| ConstructInterceptOnCohort | -0.02 | 0.04 | 0.578 | -0.02 | ac.p | ac.p_bv_onC |
| ConstructSlopeOnCohort | 0.00 | 0.04 | 0.961 | 0.00 | ac.p | ac.p_bv_onC |
| AdvInterceptOnCohort | 0.13 | 0.02 | 0.000 | 0.33 | ac.p | ac.p_bv_onC |
| AdvSlopeOnCohort | -0.07 | 0.02 | 0.002 | -0.22 | ac.p | ac.p_bv_onC |
| Cnsrct.Intercept_Corr_Adv_Intercept | -0.06 | 0.01 | 0.000 | -0.29 | ac.p | ac.p_bv_onC |
| Cnsrct.Intercept_Corr_Adv_Slope | 0.03 | 0.01 | 0.040 | 0.16 | ac.p | ac.p_bv_onC |
| Cnsrct.Slope_Corr_Adv_Intercept | 0.01 | 0.01 | 0.581 | 0.04 | ac.p | ac.p_bv_onC |
| Cnsrct.Slope_Corr_Adv_Slope | -0.04 | 0.01 | 0.008 | -0.33 | ac.p | ac.p_bv_onC |
| ConstructInterceptOfIntercept | 3.48 | 0.04 | 0.000 | 7.71 | at.y | at.y_bv_onC |
| ConstructVarianceOfIntercept | 0.20 | 0.03 | 0.000 | 0.98 | at.y | at.y_bv_onC |
| ConstructInterceptOfSlope | 0.15 | 0.05 | 0.003 | 0.29 | at.y | at.y_bv_onC |
| ConstructVarianceOfSlope | 0.27 | 0.06 | 0.000 | 1.00 | at.y | at.y_bv_onC |
| AdvInterceptOfIntercept | 1.50 | 0.02 | 0.000 | 4.63 | at.y | at.y_bv_onC |
| AdvVarianceOfIntercept | 0.09 | 0.01 | 0.000 | 0.89 | at.y | at.y_bv_onC |
| AdvInterceptOfSlope | -0.03 | 0.03 | 0.322 | -0.11 | at.y | at.y_bv_onC |
| AdvVarianceOfSlope | 0.06 | 0.02 | 0.002 | 0.96 | at.y | at.y_bv_onC |
| ConstructInterceptOnCohort | -0.07 | 0.03 | 0.013 | -0.13 | at.y | at.y_bv_onC |
| ConstructSlopeOnCohort | -0.02 | 0.04 | 0.598 | -0.03 | at.y | at.y_bv_onC |
| AdvInterceptOnCohort | 0.13 | 0.02 | 0.000 | 0.33 | at.y | at.y_bv_onC |
| AdvSlopeOnCohort | -0.07 | 0.02 | 0.002 | -0.21 | at.y | at.y_bv_onC |
| Cnsrct.Intercept_Corr_Adv_Intercept | -0.05 | 0.01 | 0.000 | -0.39 | at.y | at.y_bv_onC |
| Cnsrct.Intercept_Corr_Adv_Slope | 0.04 | 0.01 | 0.001 | 0.31 | at.y | at.y_bv_onC |
| Cnsrct.Slope_Corr_Adv_Intercept | 0.01 | 0.01 | 0.258 | 0.08 | at.y | at.y_bv_onC |
| Cnsrct.Slope_Corr_Adv_Slope | -0.06 | 0.01 | 0.000 | -0.47 | at.y | at.y_bv_onC |
| ConstructInterceptOfIntercept | 2.91 | 0.04 | 0.000 | 5.62 | at.p | at.p_bv_onC |
| ConstructVarianceOfIntercept | 0.25 | 0.02 | 0.000 | 0.95 | at.p | at.p_bv_onC |
| ConstructInterceptOfSlope | 0.14 | 0.04 | 0.000 | 0.45 | at.p | at.p_bv_onC |
| ConstructVarianceOfSlope | 0.10 | 0.04 | 0.021 | 1.00 | at.p | at.p_bv_onC |
| AdvInterceptOfIntercept | 1.50 | 0.02 | 0.000 | 4.65 | at.p | at.p_bv_onC |
| AdvVarianceOfIntercept | 0.09 | 0.01 | 0.000 | 0.89 | at.p | at.p_bv_onC |
| AdvInterceptOfSlope | -0.02 | 0.03 | 0.386 | -0.10 | at.p | at.p_bv_onC |
| AdvVarianceOfSlope | 0.06 | 0.02 | 0.002 | 0.95 | at.p | at.p_bv_onC |
| ConstructInterceptOnCohort | 0.15 | 0.03 | 0.000 | 0.23 | at.p | at.p_bv_onC |
| ConstructSlopeOnCohort | -0.02 | 0.03 | 0.409 | -0.06 | at.p | at.p_bv_onC |
| AdvInterceptOnCohort | 0.13 | 0.02 | 0.000 | 0.33 | at.p | at.p_bv_onC |
| AdvSlopeOnCohort | -0.07 | 0.02 | 0.002 | -0.22 | at.p | at.p_bv_onC |
| Cnsrct.Intercept_Corr_Adv_Intercept | -0.04 | 0.01 | 0.000 | -0.29 | at.p | at.p_bv_onC |
| Cnsrct.Intercept_Corr_Adv_Slope | 0.02 | 0.01 | 0.084 | 0.14 | at.p | at.p_bv_onC |
| Cnsrct.Slope_Corr_Adv_Intercept | 0.00 | 0.01 | 0.609 | 0.04 | at.p | at.p_bv_onC |
| Cnsrct.Slope_Corr_Adv_Slope | -0.02 | 0.01 | 0.177 | -0.19 | at.p | at.p_bv_onC |
| ConstructInterceptOfIntercept | 3.72 | 0.04 | 0.000 | 9.02 | ic.y | ic.y_bv_onC |
| ConstructVarianceOfIntercept | 0.17 | 0.03 | 0.000 | 0.99 | ic.y | ic.y_bv_onC |
| ConstructInterceptOfSlope | 0.26 | 0.05 | 0.000 | 1.12 | ic.y | ic.y_bv_onC |
| ConstructVarianceOfSlope | 0.06 | 0.06 | 0.365 | 1.00 | ic.y | ic.y_bv_onC |
| AdvInterceptOfIntercept | 1.50 | 0.02 | 0.000 | 4.64 | ic.y | ic.y_bv_onC |
| AdvVarianceOfIntercept | 0.09 | 0.01 | 0.000 | 0.89 | ic.y | ic.y_bv_onC |
| AdvInterceptOfSlope | -0.03 | 0.03 | 0.337 | -0.11 | ic.y | ic.y_bv_onC |
| AdvVarianceOfSlope | 0.06 | 0.02 | 0.002 | 0.96 | ic.y | ic.y_bv_onC |
| ConstructInterceptOnCohort | -0.06 | 0.03 | 0.030 | -0.12 | ic.y | ic.y_bv_onC |
| ConstructSlopeOnCohort | 0.00 | 0.04 | 0.935 | -0.01 | ic.y | ic.y_bv_onC |
| AdvInterceptOnCohort | 0.13 | 0.02 | 0.000 | 0.33 | ic.y | ic.y_bv_onC |
| AdvSlopeOnCohort | -0.07 | 0.02 | 0.002 | -0.21 | ic.y | ic.y_bv_onC |
| Cnsrct.Intercept_Corr_Adv_Intercept | -0.03 | 0.01 | 0.000 | -0.24 | ic.y | ic.y_bv_onC |
| Cnsrct.Intercept_Corr_Adv_Slope | 0.02 | 0.01 | 0.063 | 0.19 | ic.y | ic.y_bv_onC |
| Cnsrct.Slope_Corr_Adv_Intercept | -0.01 | 0.01 | 0.233 | -0.18 | ic.y | ic.y_bv_onC |
| Cnsrct.Slope_Corr_Adv_Slope | -0.04 | 0.02 | 0.020 | -0.59 | ic.y | ic.y_bv_onC |
| ConstructInterceptOfIntercept | 3.51 | 0.04 | 0.000 | 7.15 | ic.p | ic.p_bv_onC |
| ConstructVarianceOfIntercept | 0.23 | 0.02 | 0.000 | 0.95 | ic.p | ic.p_bv_onC |
| ConstructInterceptOfSlope | 0.31 | 0.04 | 0.000 | 0.75 | ic.p | ic.p_bv_onC |
| ConstructVarianceOfSlope | 0.17 | 0.04 | 0.000 | 0.98 | ic.p | ic.p_bv_onC |
| AdvInterceptOfIntercept | 1.50 | 0.02 | 0.000 | 4.63 | ic.p | ic.p_bv_onC |
| AdvVarianceOfIntercept | 0.09 | 0.01 | 0.000 | 0.89 | ic.p | ic.p_bv_onC |
| AdvInterceptOfSlope | -0.03 | 0.03 | 0.367 | -0.10 | ic.p | ic.p_bv_onC |
| AdvVarianceOfSlope | 0.06 | 0.02 | 0.001 | 0.95 | ic.p | ic.p_bv_onC |
| ConstructInterceptOnCohort | 0.14 | 0.03 | 0.000 | 0.23 | ic.p | ic.p_bv_onC |
| ConstructSlopeOnCohort | -0.07 | 0.03 | 0.032 | -0.13 | ic.p | ic.p_bv_onC |
| AdvInterceptOnCohort | 0.13 | 0.02 | 0.000 | 0.33 | ic.p | ic.p_bv_onC |
| AdvSlopeOnCohort | -0.07 | 0.02 | 0.002 | -0.21 | ic.p | ic.p_bv_onC |
| Cnsrct.Intercept_Corr_Adv_Intercept | -0.04 | 0.01 | 0.000 | -0.24 | ic.p | ic.p_bv_onC |
| Cnsrct.Intercept_Corr_Adv_Slope | 0.03 | 0.01 | 0.003 | 0.24 | ic.p | ic.p_bv_onC |
| Cnsrct.Slope_Corr_Adv_Intercept | -0.01 | 0.01 | 0.372 | -0.06 | ic.p | ic.p_bv_onC |
| Cnsrct.Slope_Corr_Adv_Slope | -0.04 | 0.01 | 0.005 | -0.34 | ic.p | ic.p_bv_onC |
| ConstructInterceptOfIntercept | 3.38 | 0.03 | 0.000 | 8.55 | ne.y | ne.y_bv_onC |
| ConstructVarianceOfIntercept | 0.16 | 0.02 | 0.000 | 1.00 | ne.y | ne.y_bv_onC |
| ConstructInterceptOfSlope | 0.35 | 0.04 | 0.000 | 0.91 | ne.y | ne.y_bv_onC |
| ConstructVarianceOfSlope | 0.15 | 0.04 | 0.000 | 0.98 | ne.y | ne.y_bv_onC |
| AdvInterceptOfIntercept | 1.50 | 0.02 | 0.000 | 4.64 | ne.y | ne.y_bv_onC |
| AdvVarianceOfIntercept | 0.09 | 0.01 | 0.000 | 0.89 | ne.y | ne.y_bv_onC |
| AdvInterceptOfSlope | -0.03 | 0.03 | 0.360 | -0.10 | ne.y | ne.y_bv_onC |
| AdvVarianceOfSlope | 0.06 | 0.02 | 0.001 | 0.96 | ne.y | ne.y_bv_onC |
| ConstructInterceptOnCohort | 0.01 | 0.03 | 0.841 | 0.01 | ne.y | ne.y_bv_onC |
| ConstructSlopeOnCohort | -0.07 | 0.03 | 0.018 | -0.15 | ne.y | ne.y_bv_onC |
| AdvInterceptOnCohort | 0.13 | 0.02 | 0.000 | 0.33 | ne.y | ne.y_bv_onC |
| AdvSlopeOnCohort | -0.07 | 0.02 | 0.002 | -0.21 | ne.y | ne.y_bv_onC |
| Cnsrct.Intercept_Corr_Adv_Intercept | -0.03 | 0.01 | 0.000 | -0.29 | ne.y | ne.y_bv_onC |
| Cnsrct.Intercept_Corr_Adv_Slope | 0.03 | 0.01 | 0.001 | 0.29 | ne.y | ne.y_bv_onC |
| Cnsrct.Slope_Corr_Adv_Intercept | 0.00 | 0.01 | 0.570 | 0.04 | ne.y | ne.y_bv_onC |
| Cnsrct.Slope_Corr_Adv_Slope | -0.05 | 0.01 | 0.000 | -0.54 | ne.y | ne.y_bv_onC |
| ConstructInterceptOfIntercept | 3.34 | 0.03 | 0.000 | 7.36 | ne.p | ne.p_bv_onC |
| ConstructVarianceOfIntercept | 0.20 | 0.02 | 0.000 | 0.97 | ne.p | ne.p_bv_onC |
| ConstructInterceptOfSlope | 0.22 | 0.03 | 0.000 | 0.58 | ne.p | ne.p_bv_onC |
| ConstructVarianceOfSlope | 0.14 | 0.03 | 0.000 | 1.00 | ne.p | ne.p_bv_onC |
| AdvInterceptOfIntercept | 1.50 | 0.02 | 0.000 | 4.64 | ne.p | ne.p_bv_onC |
| AdvVarianceOfIntercept | 0.09 | 0.01 | 0.000 | 0.89 | ne.p | ne.p_bv_onC |
| AdvInterceptOfSlope | -0.02 | 0.03 | 0.392 | -0.09 | ne.p | ne.p_bv_onC |
| AdvVarianceOfSlope | 0.07 | 0.02 | 0.001 | 0.96 | ne.p | ne.p_bv_onC |
| ConstructInterceptOnCohort | 0.10 | 0.02 | 0.000 | 0.17 | ne.p | ne.p_bv_onC |
| ConstructSlopeOnCohort | 0.02 | 0.02 | 0.438 | 0.04 | ne.p | ne.p_bv_onC |
| AdvInterceptOnCohort | 0.13 | 0.02 | 0.000 | 0.33 | ne.p | ne.p_bv_onC |
| AdvSlopeOnCohort | -0.07 | 0.02 | 0.002 | -0.21 | ne.p | ne.p_bv_onC |
| Cnsrct.Intercept_Corr_Adv_Intercept | -0.02 | 0.01 | 0.000 | -0.18 | ne.p | ne.p_bv_onC |
| Cnsrct.Intercept_Corr_Adv_Slope | 0.01 | 0.01 | 0.340 | 0.07 | ne.p | ne.p_bv_onC |
| Cnsrct.Slope_Corr_Adv_Intercept | -0.01 | 0.01 | 0.194 | -0.07 | ne.p | ne.p_bv_onC |
| Cnsrct.Slope_Corr_Adv_Slope | -0.02 | 0.01 | 0.064 | -0.18 | ne.p | ne.p_bv_onC |
| ConstructInterceptOfIntercept | 4.25 | 0.04 | 0.000 | 7.15 | ag.y | ag.y_bv_onC |
| ConstructVarianceOfIntercept | 0.33 | 0.04 | 0.000 | 0.93 | ag.y | ag.y_bv_onC |
| ConstructInterceptOfSlope | 0.01 | 0.05 | 0.858 | 0.02 | ag.y | ag.y_bv_onC |
| ConstructVarianceOfSlope | 0.26 | 0.07 | 0.000 | 0.99 | ag.y | ag.y_bv_onC |
| AdvInterceptOfIntercept | 1.50 | 0.02 | 0.000 | 4.64 | ag.y | ag.y_bv_onC |
| AdvVarianceOfIntercept | 0.09 | 0.01 | 0.000 | 0.90 | ag.y | ag.y_bv_onC |
| AdvInterceptOfSlope | -0.03 | 0.03 | 0.362 | -0.10 | ag.y | ag.y_bv_onC |
| AdvVarianceOfSlope | 0.07 | 0.02 | 0.001 | 0.96 | ag.y | ag.y_bv_onC |
| ConstructInterceptOnCohort | -0.20 | 0.03 | 0.000 | -0.27 | ag.y | ag.y_bv_onC |
| ConstructSlopeOnCohort | 0.05 | 0.04 | 0.231 | 0.08 | ag.y | ag.y_bv_onC |
| AdvInterceptOnCohort | 0.13 | 0.02 | 0.000 | 0.32 | ag.y | ag.y_bv_onC |
| AdvSlopeOnCohort | -0.07 | 0.02 | 0.002 | -0.21 | ag.y | ag.y_bv_onC |
| Cnsrct.Intercept_Corr_Adv_Intercept | -0.05 | 0.01 | 0.000 | -0.31 | ag.y | ag.y_bv_onC |
| Cnsrct.Intercept_Corr_Adv_Slope | 0.02 | 0.01 | 0.076 | 0.16 | ag.y | ag.y_bv_onC |
| Cnsrct.Slope_Corr_Adv_Intercept | 0.00 | 0.01 | 0.740 | 0.03 | ag.y | ag.y_bv_onC |
| Cnsrct.Slope_Corr_Adv_Slope | -0.05 | 0.02 | 0.003 | -0.38 | ag.y | ag.y_bv_onC |
| ConstructInterceptOfIntercept | 3.59 | 0.04 | 0.000 | 5.68 | ag.p | ag.p_bv_onC |
| ConstructVarianceOfIntercept | 0.40 | 0.03 | 0.000 | 1.00 | ag.p | ag.p_bv_onC |
| ConstructInterceptOfSlope | 0.18 | 0.04 | 0.000 | 0.39 | ag.p | ag.p_bv_onC |
| ConstructVarianceOfSlope | 0.21 | 0.05 | 0.000 | 1.00 | ag.p | ag.p_bv_onC |
| AdvInterceptOfIntercept | 1.50 | 0.02 | 0.000 | 4.63 | ag.p | ag.p_bv_onC |
| AdvVarianceOfIntercept | 0.09 | 0.01 | 0.000 | 0.89 | ag.p | ag.p_bv_onC |
| AdvInterceptOfSlope | -0.02 | 0.03 | 0.418 | -0.09 | ag.p | ag.p_bv_onC |
| AdvVarianceOfSlope | 0.07 | 0.02 | 0.001 | 0.95 | ag.p | ag.p_bv_onC |
| ConstructInterceptOnCohort | 0.06 | 0.03 | 0.092 | 0.07 | ag.p | ag.p_bv_onC |
| ConstructSlopeOnCohort | 0.03 | 0.03 | 0.341 | 0.05 | ag.p | ag.p_bv_onC |
| AdvInterceptOnCohort | 0.13 | 0.02 | 0.000 | 0.33 | ag.p | ag.p_bv_onC |
| AdvSlopeOnCohort | -0.07 | 0.02 | 0.002 | -0.21 | ag.p | ag.p_bv_onC |
| Cnsrct.Intercept_Corr_Adv_Intercept | -0.05 | 0.01 | 0.000 | -0.27 | ag.p | ag.p_bv_onC |
| Cnsrct.Intercept_Corr_Adv_Slope | 0.02 | 0.01 | 0.209 | 0.10 | ag.p | ag.p_bv_onC |
| Cnsrct.Slope_Corr_Adv_Intercept | 0.00 | 0.01 | 0.737 | 0.02 | ag.p | ag.p_bv_onC |
| Cnsrct.Slope_Corr_Adv_Slope | -0.04 | 0.01 | 0.002 | -0.32 | ag.p | ag.p_bv_onC |
| ConstructInterceptOfIntercept | 3.04 | 0.05 | 0.000 | 5.33 | fear.y | fear.y_bv_onC |
| ConstructVarianceOfIntercept | 0.29 | 0.05 | 0.000 | 0.91 | fear.y | fear.y_bv_onC |
| ConstructInterceptOfSlope | 0.82 | 0.06 | 0.000 | 1.87 | fear.y | fear.y_bv_onC |
| ConstructVarianceOfSlope | 0.18 | 0.09 | 0.036 | 0.93 | fear.y | fear.y_bv_onC |
| AdvInterceptOfIntercept | 1.50 | 0.02 | 0.000 | 4.66 | fear.y | fear.y_bv_onC |
| AdvVarianceOfIntercept | 0.09 | 0.01 | 0.000 | 0.89 | fear.y | fear.y_bv_onC |
| AdvInterceptOfSlope | -0.03 | 0.03 | 0.334 | -0.11 | fear.y | fear.y_bv_onC |
| AdvVarianceOfSlope | 0.06 | 0.02 | 0.003 | 0.96 | fear.y | fear.y_bv_onC |
| ConstructInterceptOnCohort | 0.22 | 0.04 | 0.000 | 0.31 | fear.y | fear.y_bv_onC |
| ConstructSlopeOnCohort | -0.14 | 0.04 | 0.001 | -0.26 | fear.y | fear.y_bv_onC |
| AdvInterceptOnCohort | 0.13 | 0.02 | 0.000 | 0.33 | fear.y | fear.y_bv_onC |
| AdvSlopeOnCohort | -0.07 | 0.02 | 0.002 | -0.21 | fear.y | fear.y_bv_onC |
| Cnsrct.Intercept_Corr_Adv_Intercept | -0.03 | 0.01 | 0.008 | -0.17 | fear.y | fear.y_bv_onC |
| Cnsrct.Intercept_Corr_Adv_Slope | 0.04 | 0.01 | 0.005 | 0.28 | fear.y | fear.y_bv_onC |
| Cnsrct.Slope_Corr_Adv_Intercept | 0.02 | 0.01 | 0.204 | 0.12 | fear.y | fear.y_bv_onC |
| Cnsrct.Slope_Corr_Adv_Slope | -0.06 | 0.02 | 0.001 | -0.53 | fear.y | fear.y_bv_onC |
| ConstructInterceptOfIntercept | 3.34 | 0.04 | 0.000 | 5.73 | fear.p | fear.p_bv_onC |
| ConstructVarianceOfIntercept | 0.31 | 0.03 | 0.000 | 0.92 | fear.p | fear.p_bv_onC |
| ConstructInterceptOfSlope | 0.46 | 0.04 | 0.000 | 1.34 | fear.p | fear.p_bv_onC |
| ConstructVarianceOfSlope | 0.12 | 0.06 | 0.052 | 1.00 | fear.p | fear.p_bv_onC |
| AdvInterceptOfIntercept | 1.50 | 0.02 | 0.000 | 4.64 | fear.p | fear.p_bv_onC |
| AdvVarianceOfIntercept | 0.09 | 0.01 | 0.000 | 0.89 | fear.p | fear.p_bv_onC |
| AdvInterceptOfSlope | -0.03 | 0.03 | 0.355 | -0.10 | fear.p | fear.p_bv_onC |
| AdvVarianceOfSlope | 0.06 | 0.02 | 0.002 | 0.96 | fear.p | fear.p_bv_onC |
| ConstructInterceptOnCohort | 0.21 | 0.03 | 0.000 | 0.28 | fear.p | fear.p_bv_onC |
| ConstructSlopeOnCohort | -0.02 | 0.03 | 0.502 | -0.05 | fear.p | fear.p_bv_onC |
| AdvInterceptOnCohort | 0.13 | 0.02 | 0.000 | 0.33 | fear.p | fear.p_bv_onC |
| AdvSlopeOnCohort | -0.07 | 0.02 | 0.002 | -0.21 | fear.p | fear.p_bv_onC |
| Cnsrct.Intercept_Corr_Adv_Intercept | -0.02 | 0.01 | 0.020 | -0.13 | fear.p | fear.p_bv_onC |
| Cnsrct.Intercept_Corr_Adv_Slope | 0.00 | 0.01 | 0.705 | 0.03 | fear.p | fear.p_bv_onC |
| Cnsrct.Slope_Corr_Adv_Intercept | 0.00 | 0.01 | 0.701 | -0.04 | fear.p | fear.p_bv_onC |
| Cnsrct.Slope_Corr_Adv_Slope | -0.02 | 0.01 | 0.254 | -0.18 | fear.p | fear.p_bv_onC |
| ConstructInterceptOfIntercept | 2.92 | 0.04 | 0.000 | 5.56 | fr.y | fr.y_bv_onC |
| ConstructVarianceOfIntercept | 0.27 | 0.04 | 0.000 | 0.99 | fr.y | fr.y_bv_onC |
| ConstructInterceptOfSlope | 0.29 | 0.06 | 0.000 | 0.59 | fr.y | fr.y_bv_onC |
| ConstructVarianceOfSlope | 0.24 | 0.08 | 0.003 | 0.99 | fr.y | fr.y_bv_onC |
| AdvInterceptOfIntercept | 1.50 | 0.02 | 0.000 | 4.60 | fr.y | fr.y_bv_onC |
| AdvVarianceOfIntercept | 0.09 | 0.01 | 0.000 | 0.89 | fr.y | fr.y_bv_onC |
| AdvInterceptOfSlope | -0.03 | 0.03 | 0.360 | -0.10 | fr.y | fr.y_bv_onC |
| AdvVarianceOfSlope | 0.07 | 0.02 | 0.001 | 0.96 | fr.y | fr.y_bv_onC |
| ConstructInterceptOnCohort | -0.05 | 0.03 | 0.105 | -0.08 | fr.y | fr.y_bv_onC |
| ConstructSlopeOnCohort | -0.06 | 0.04 | 0.165 | -0.10 | fr.y | fr.y_bv_onC |
| AdvInterceptOnCohort | 0.13 | 0.02 | 0.000 | 0.32 | fr.y | fr.y_bv_onC |
| AdvSlopeOnCohort | -0.07 | 0.02 | 0.002 | -0.21 | fr.y | fr.y_bv_onC |
| Cnsrct.Intercept_Corr_Adv_Intercept | -0.04 | 0.01 | 0.000 | -0.25 | fr.y | fr.y_bv_onC |
| Cnsrct.Intercept_Corr_Adv_Slope | 0.04 | 0.01 | 0.003 | 0.27 | fr.y | fr.y_bv_onC |
| Cnsrct.Slope_Corr_Adv_Intercept | 0.01 | 0.01 | 0.527 | 0.05 | fr.y | fr.y_bv_onC |
| Cnsrct.Slope_Corr_Adv_Slope | -0.07 | 0.02 | 0.000 | -0.54 | fr.y | fr.y_bv_onC |
| ConstructInterceptOfIntercept | 3.05 | 0.04 | 0.000 | 5.27 | fr.p | fr.p_bv_onC |
| ConstructVarianceOfIntercept | 0.33 | 0.03 | 0.000 | 1.00 | fr.p | fr.p_bv_onC |
| ConstructInterceptOfSlope | 0.14 | 0.04 | 0.001 | 0.34 | fr.p | fr.p_bv_onC |
| ConstructVarianceOfSlope | 0.17 | 0.06 | 0.004 | 0.98 | fr.p | fr.p_bv_onC |
| AdvInterceptOfIntercept | 1.50 | 0.02 | 0.000 | 4.65 | fr.p | fr.p_bv_onC |
| AdvVarianceOfIntercept | 0.09 | 0.01 | 0.000 | 0.89 | fr.p | fr.p_bv_onC |
| AdvInterceptOfSlope | -0.03 | 0.03 | 0.376 | -0.10 | fr.p | fr.p_bv_onC |
| AdvVarianceOfSlope | 0.06 | 0.02 | 0.001 | 0.96 | fr.p | fr.p_bv_onC |
| ConstructInterceptOnCohort | 0.04 | 0.03 | 0.162 | 0.06 | fr.p | fr.p_bv_onC |
| ConstructSlopeOnCohort | 0.08 | 0.03 | 0.016 | 0.16 | fr.p | fr.p_bv_onC |
| AdvInterceptOnCohort | 0.13 | 0.02 | 0.000 | 0.33 | fr.p | fr.p_bv_onC |
| AdvSlopeOnCohort | -0.07 | 0.02 | 0.002 | -0.21 | fr.p | fr.p_bv_onC |
| Cnsrct.Intercept_Corr_Adv_Intercept | -0.04 | 0.01 | 0.000 | -0.22 | fr.p | fr.p_bv_onC |
| Cnsrct.Intercept_Corr_Adv_Slope | 0.02 | 0.01 | 0.076 | 0.14 | fr.p | fr.p_bv_onC |
| Cnsrct.Slope_Corr_Adv_Intercept | -0.01 | 0.01 | 0.417 | -0.06 | fr.p | fr.p_bv_onC |
| Cnsrct.Slope_Corr_Adv_Slope | -0.04 | 0.01 | 0.007 | -0.35 | fr.p | fr.p_bv_onC |
| ConstructInterceptOfIntercept | 3.41 | 0.06 | 0.000 | 5.15 | shy.y | shy.y_bv_onC |
| ConstructVarianceOfIntercept | 0.43 | 0.07 | 0.000 | 0.99 | shy.y | shy.y_bv_onC |
| ConstructInterceptOfSlope | 0.34 | 0.07 | 0.000 | 0.81 | shy.y | shy.y_bv_onC |
| ConstructVarianceOfSlope | 0.14 | 0.14 | 0.304 | 0.84 | shy.y | shy.y_bv_onC |
| AdvInterceptOfIntercept | 1.50 | 0.02 | 0.000 | 4.64 | shy.y | shy.y_bv_onC |
| AdvVarianceOfIntercept | 0.09 | 0.01 | 0.000 | 0.89 | shy.y | shy.y_bv_onC |
| AdvInterceptOfSlope | -0.03 | 0.03 | 0.373 | -0.10 | shy.y | shy.y_bv_onC |
| AdvVarianceOfSlope | 0.06 | 0.02 | 0.002 | 0.95 | shy.y | shy.y_bv_onC |
| ConstructInterceptOnCohort | 0.10 | 0.05 | 0.033 | 0.12 | shy.y | shy.y_bv_onC |
| ConstructSlopeOnCohort | -0.21 | 0.06 | 0.000 | -0.41 | shy.y | shy.y_bv_onC |
| AdvInterceptOnCohort | 0.13 | 0.02 | 0.000 | 0.33 | shy.y | shy.y_bv_onC |
| AdvSlopeOnCohort | -0.07 | 0.02 | 0.002 | -0.21 | shy.y | shy.y_bv_onC |
| Cnsrct.Intercept_Corr_Adv_Intercept | -0.01 | 0.01 | 0.363 | -0.06 | shy.y | shy.y_bv_onC |
| Cnsrct.Intercept_Corr_Adv_Slope | 0.02 | 0.02 | 0.342 | 0.10 | shy.y | shy.y_bv_onC |
| Cnsrct.Slope_Corr_Adv_Intercept | -0.01 | 0.02 | 0.721 | -0.05 | shy.y | shy.y_bv_onC |
| Cnsrct.Slope_Corr_Adv_Slope | -0.03 | 0.02 | 0.114 | -0.35 | shy.y | shy.y_bv_onC |
| ConstructInterceptOfIntercept | 3.42 | 0.05 | 0.000 | 4.38 | shy.p | shy.p_bv_onC |
| ConstructVarianceOfIntercept | 0.60 | 0.05 | 0.000 | 0.99 | shy.p | shy.p_bv_onC |
| ConstructInterceptOfSlope | 0.11 | 0.05 | 0.031 | 0.23 | shy.p | shy.p_bv_onC |
| ConstructVarianceOfSlope | 0.21 | 0.08 | 0.010 | 1.00 | shy.p | shy.p_bv_onC |
| AdvInterceptOfIntercept | 1.50 | 0.02 | 0.000 | 4.60 | shy.p | shy.p_bv_onC |
| AdvVarianceOfIntercept | 0.10 | 0.01 | 0.000 | 0.90 | shy.p | shy.p_bv_onC |
| AdvInterceptOfSlope | -0.03 | 0.03 | 0.343 | -0.10 | shy.p | shy.p_bv_onC |
| AdvVarianceOfSlope | 0.07 | 0.02 | 0.001 | 0.96 | shy.p | shy.p_bv_onC |
| ConstructInterceptOnCohort | 0.07 | 0.04 | 0.078 | 0.08 | shy.p | shy.p_bv_onC |
| ConstructSlopeOnCohort | -0.03 | 0.04 | 0.489 | -0.05 | shy.p | shy.p_bv_onC |
| AdvInterceptOnCohort | 0.13 | 0.02 | 0.000 | 0.32 | shy.p | shy.p_bv_onC |
| AdvSlopeOnCohort | -0.07 | 0.02 | 0.002 | -0.20 | shy.p | shy.p_bv_onC |
| Cnsrct.Intercept_Corr_Adv_Intercept | 0.02 | 0.01 | 0.088 | 0.09 | shy.p | shy.p_bv_onC |
| Cnsrct.Intercept_Corr_Adv_Slope | -0.02 | 0.02 | 0.310 | -0.08 | shy.p | shy.p_bv_onC |
| Cnsrct.Slope_Corr_Adv_Intercept | -0.03 | 0.01 | 0.005 | -0.22 | shy.p | shy.p_bv_onC |
| Cnsrct.Slope_Corr_Adv_Slope | 0.03 | 0.02 | 0.037 | 0.27 | shy.p | shy.p_bv_onC |

# Table S9. Fit Indices for Univariate Latent Growth Curve Models for Effortful Control and Emotional Stability Conditioned on Cohort and Adversity Factor Scores

| chisq | df | rmsea | tli | cfi | ModID |
| --- | --- | --- | --- | --- | --- |
| 34.955 | 5 | 0.094 | 0.896 | 0.957 | ec.y_uv.onS_ADVandC |
| 30.533 | 5 | 0.087 | 0.947 | 0.978 | ec.p_uv.onS_ADVandC |
| 11.436 | 5 | 0.043 | 0.978 | 0.991 | ac.y_uv.onS_ADVandC |
| 23.721 | 5 | 0.074 | 0.954 | 0.981 | ac.p_uv.onS_ADVandC |
| 25.659 | 5 | 0.078 | 0.878 | 0.949 | at.y_uv.onS_ADVandC |
| 8.809 | 5 | 0.033 | 0.988 | 0.995 | at.p_uv.onS_ADVandC |
| 24.643 | 5 | 0.076 | 0.859 | 0.941 | ic.y_uv.onS_ADVandC |
| 23.191 | 5 | 0.073 | 0.93 | 0.971 | ic.p_uv.onS_ADVandC |
| 94.998 | 5 | 0.162 | 0.466 | 0.777 | ne.y_uv.onS_ADVandC |
| 45.353 | 5 | 0.109 | 0.898 | 0.957 | ne.p_uv.onS_ADVandC |
| 24.371 | 5 | 0.075 | 0.912 | 0.963 | ag.y_uv.onS_ADVandC |
| 22.466 | 5 | 0.072 | 0.95 | 0.979 | ag.p_uv.onS_ADVandC |
| 84.967 | 5 | 0.153 | 0.454 | 0.772 | fear.y_uv.onS_ADVandC |
| 42.525 | 5 | 0.105 | 0.872 | 0.947 | fear.p_uv.onS_ADVandC |
| 49.4 | 5 | 0.114 | 0.675 | 0.865 | fr.y_uv.onS_ADVandC |
| 23.399 | 5 | 0.073 | 0.935 | 0.973 | fr.p_uv.onS_ADVandC |
| 40.66 | 5 | 0.102 | 0.801 | 0.917 | shy.y_uv.onS_ADVandC |
| 3.655 | 5 | 0 | 1.004 | 1 | shy.p_uv.onS_ADVandC |

# Table S10. Youth and Parent Report Estimates for Prevalence of Growth Despite Adversity (Estimates for Univariate Model Conditioned on Initial Adversity, Change in Adversity, and Cohort)

|  | Low Adversity | | % Change >/= SESOI despite baseline Adversity | | % Change >/= SESOI despite increases in Adversity | |
| --- | --- | --- | --- | --- | --- | --- |
| Model | Decrease | Increase | Decrease | Increase | Decrease | Increase |
| ec.y_bv | 17% (83/488) | 48% (236/488) | 17% (16/93) | 40% (37/93) | 38% (24/63) | 19% (12/63) |
| ec.p_bv | 19% (95/488) | 49% (239/488) | 20% (19/93) | 47% (44/93) | 29% (18/63) | 38% (24/63) |
| ac.y_bv | 45% (222/488) | 16% (76/488) | 38% (35/93) | 15% (14/93) | 65% (41/63) | 5% (3/63) |
| ac.p_bv | 25% (121/488) | 30% (146/488) | 25% (23/93) | 25% (23/93) | 43% (27/63) | 19% (12/63) |
| at.y_bv | 21% (101/488) | 51% (251/488) | 11% (10/93) | 55% (51/93) | 40% (25/63) | 32% (20/63) |
| at.p_bv | 8% (40/488) | 49% (241/488) | 4% (4/93) | 57% (53/93) | 10% (6/63) | 43% (27/63) |
| ic.y_bv | 0% (0/488) | 96% (468/488) | 0% (0/93) | 72% (67/93) | 5% (3/63) | 73% (46/63) |
| ic.p_bv | 8% (40/488) | 74% (360/488) | 9% (8/93) | 57% (53/93) | 16% (10/63) | 44% (28/63) |
| ne.y_bv | 3% (16/488) | 81% (397/488) | 5% (5/93) | 76% (71/93) | 19% (12/63) | 49% (31/63) |
| ne.p_bv | 11% (54/488) | 72% (353/488) | 13% (12/93) | 66% (61/93) | 16% (10/63) | 54% (34/63) |
| ag.y_bv | 18% (88/488) | 40% (194/488) | 23% (21/93) | 44% (41/93) | 46% (29/63) | 16% (10/63) |
| ag.p_bv | 14% (66/488) | 61% (299/488) | 12% (11/93) | 67% (62/93) | 30% (19/63) | 35% (22/63) |
| fear.y_bv | 0% (0/488) | 99% (484/488) | 0% (0/93) | 100% (93/93) | 0% (0/63) | 95% (60/63) |
| fear.p_bv | 0% (1/488) | 96% (467/488) | 0% (0/93) | 95% (88/93) | 0% (0/63) | 89% (56/63) |
| fr.y_bv | 5% (25/488) | 67% (327/488) | 10% (9/93) | 65% (60/93) | 25% (16/63) | 37% (23/63) |
| fr.p_bv | 5% (24/488) | 69% (339/488) | 5% (5/93) | 58% (54/93) | 21% (13/63) | 40% (25/63) |
| shy.y_bv | 7% (34/488) | 39% (192/488) | 16% (15/93) | 17% (16/93) | 13% (8/63) | 33% (21/63) |
| shy.p_bv | 14% (69/488) | 35% (171/488) | 31% (29/93) | 12% (11/93) | 8% (5/63) | 46% (29/63) |

# Table S11. Fit indices for all Research Question 04 models

| chisq | df | rmsea | tli | cfi | ModID |
| --- | --- | --- | --- | --- | --- |
| 0 | 0 | 0 | 1 | 1 | Univariate PSS |
| 0 | 0 | 0 | 1 | 1 | Univariate ProSoc |
| 0 | 0 | 0 | 1 | 1 | Univariate Self Esteem |
| 75.555 | 21 | 0.062 | 0.939 | 0.971 | associative_ec.p_anx.y |
| 97.385 | 21 | 0.073 | 0.905 | 0.956 | associative_ne.p_anx.y |
| 63.028 | 21 | 0.054 | 0.958 | 0.98 | associative_ec.p_avoid.y |
| 91.301 | 21 | 0.07 | 0.923 | 0.964 | associative_ne.p_avoid.y |
| 54.66 | 13 | 0.069 | 0.936 | 0.977 | associative_ec.p_PSS.y |
| 84.802 | 13 | 0.09 | 0.878 | 0.956 | associative_ne.p_PSS.y |
| 46.414 | 13 | 0.061 | 0.946 | 0.98 | associative_ec.p_ProSoc.y |
| 76.193 | 13 | 0.084 | 0.885 | 0.958 | associative_ne.p_ProSoc.y |
| 42.827 | 13 | 0.058 | 0.955 | 0.984 | associative_ec.p_SEQ.y |
| 76.038 | 13 | 0.084 | 0.894 | 0.962 | associative_ne.p_SEQ.y |
| 140.886 | 30 | 0.074 | 0.913 | 0.942 | regression_ec.p_anx.y |
| 161.443 | 30 | 0.08 | 0.886 | 0.924 | regression_ne.p_anx.y |
| 124.015 | 30 | 0.068 | 0.934 | 0.956 | regression_ec.p_avoid.y |
| 150.334 | 30 | 0.077 | 0.907 | 0.938 | regression_ne.p_avoid.y |
| 241.594 | 22 | 0.121 | 0.8 | 0.878 | regression_ec.p_PSS.y |
| 268.517 | 22 | 0.128 | 0.752 | 0.848 | regression_ne.p_PSS.y |
| 329.392 | 22 | 0.143 | 0.705 | 0.82 | regression_ec.p_ProSoc.y |
| 353.156 | 22 | 0.149 | 0.643 | 0.782 | regression_ne.p_ProSoc.y |
| 283.227 | 22 | 0.132 | 0.766 | 0.857 | regression_ec.p_SEQ.y |
| 315.679 | 22 | 0.14 | 0.709 | 0.822 | regression_ne.p_SEQ.y |

# Table S12. Univariate Latent Difference Score Models for Parenting and Prosocial

| Construct | Parameter | Estimate | SE | PValue |
| --- | --- | --- | --- | --- |
| Parenting Style | Level | 0.85 | 0.01 | 0.000 |
|  | Level Variance | 0.02 | 0.00 | 0.000 |
|  | Slope | 0.03 | 0.01 | 0.000 |
|  | Slope Variance | 0.03 | 0.00 | 0.000 |
| Prosocial | Level | 2.62 | 0.04 | 0.000 |
|  | Level Variance | 0.99 | 0.06 | 0.000 |
|  | Slope | -0.04 | 0.05 | 0.434 |
|  | Slope Variance | 1.21 | 0.08 | 0.000 |
| Self Esteem | Level | 1.70 | 0.02 | 0.000 |
|  | Level Variance | 0.23 | 0.01 | 0.000 |
|  | Slope | -0.11 | 0.02 | 0.000 |
|  | Slope Variance | 0.29 | 0.02 | 0.000 |

# Table S13. Associations between Personality, Adversity, and Third Variable

| Parameter | *r* | *p*-value | Personality | Factor |
| --- | --- | --- | --- | --- |
| I_Temperment_Corr_S_Temperment | -0.21 | 0.041 | ec.p | anx.y |
| I_Temperment_Corr_I_Adv | -0.31 | 0.000 | ec.p | anx.y |
| I_Temperment_Corr_S_Adv | 0.19 | 0.013 | ec.p | anx.y |
| I_Temperment_Corr_I_Factor | -0.30 | 0.000 | ec.p | anx.y |
| I_Temperment_Corr_S_Factor | -0.07 | 0.433 | ec.p | anx.y |
| S_Temperment_Corr_I_Adv | 0.01 | 0.840 | ec.p | anx.y |
| S_Temperment_Corr_S_Adv | -0.33 | 0.003 | ec.p | anx.y |
| S_Temperment_Corr_I_Factor | 0.15 | 0.116 | ec.p | anx.y |
| **S_Temperment_Corr_S_Factor** | **-0.24** | **0.028** | **ec.p** | **anx.y** |
| I_Adv_Corr_S_Adv | -0.38 | 0.018 | ec.p | anx.y |
| I_Adv_Corr_I_Factor | 0.44 | 0.000 | ec.p | anx.y |
| I_Adv_Corr_S_Factor | -0.20 | 0.032 | ec.p | anx.y |
| S_Adv_Corr_I_Factor | -0.32 | 0.009 | ec.p | anx.y |
| S_Adv_Corr_S_Factor | 0.51 | 0.001 | ec.p | anx.y |
| I_Factor_Corr_S_Factor | -0.21 | 0.380 | ec.p | anx.y |
| I_Temperment_Corr_S_Temperment | -0.26 | 0.008 | ne.p | anx.y |
| I_Temperment_Corr_I_Adv | -0.18 | 0.000 | ne.p | anx.y |
| I_Temperment_Corr_S_Adv | 0.07 | 0.368 | ne.p | anx.y |
| I_Temperment_Corr_I_Factor | -0.34 | 0.000 | ne.p | anx.y |
| I_Temperment_Corr_S_Factor | -0.03 | 0.762 | ne.p | anx.y |
| S_Temperment_Corr_I_Adv | -0.08 | 0.157 | ne.p | anx.y |
| S_Temperment_Corr_S_Adv | -0.18 | 0.075 | ne.p | anx.y |
| S_Temperment_Corr_I_Factor | -0.08 | 0.342 | ne.p | anx.y |
| **S_Temperment_Corr_S_Factor** | **0.04** | **0.676** | **ne.p** | **anx.y** |
| I_Adv_Corr_S_Adv | -0.38 | 0.014 | ne.p | anx.y |
| I_Adv_Corr_I_Factor | 0.45 | 0.000 | ne.p | anx.y |
| I_Adv_Corr_S_Factor | -0.22 | 0.019 | ne.p | anx.y |
| S_Adv_Corr_I_Factor | -0.33 | 0.006 | ne.p | anx.y |
| S_Adv_Corr_S_Factor | 0.54 | 0.000 | ne.p | anx.y |
| I_Factor_Corr_S_Factor | -0.20 | 0.417 | ne.p | anx.y |
| I_Temperment_Corr_S_Temperment | -0.23 | 0.013 | ec.p | avoid.y |
| I_Temperment_Corr_I_Adv | -0.30 | 0.000 | ec.p | avoid.y |
| I_Temperment_Corr_S_Adv | 0.19 | 0.012 | ec.p | avoid.y |
| I_Temperment_Corr_I_Factor | -0.16 | 0.002 | ec.p | avoid.y |
| I_Temperment_Corr_S_Factor | -0.03 | 0.616 | ec.p | avoid.y |
| S_Temperment_Corr_I_Adv | 0.01 | 0.826 | ec.p | avoid.y |
| S_Temperment_Corr_S_Adv | -0.30 | 0.003 | ec.p | avoid.y |
| S_Temperment_Corr_I_Factor | 0.09 | 0.175 | ec.p | avoid.y |
| **S_Temperment_Corr_S_Factor** | **-0.16** | **0.023** | **ec.p** | **avoid.y** |
| I_Adv_Corr_S_Adv | -0.39 | 0.013 | ec.p | avoid.y |
| I_Adv_Corr_I_Factor | 0.24 | 0.000 | ec.p | avoid.y |
| I_Adv_Corr_S_Factor | -0.10 | 0.131 | ec.p | avoid.y |
| S_Adv_Corr_I_Factor | -0.17 | 0.053 | ec.p | avoid.y |
| S_Adv_Corr_S_Factor | 0.46 | 0.000 | ec.p | avoid.y |
| I_Factor_Corr_S_Factor | -0.27 | 0.031 | ec.p | avoid.y |
| I_Temperment_Corr_S_Temperment | -0.27 | 0.004 | ne.p | avoid.y |
| I_Temperment_Corr_I_Adv | -0.18 | 0.000 | ne.p | avoid.y |
| I_Temperment_Corr_S_Adv | 0.07 | 0.342 | ne.p | avoid.y |
| I_Temperment_Corr_I_Factor | -0.10 | 0.052 | ne.p | avoid.y |
| I_Temperment_Corr_S_Factor | -0.04 | 0.493 | ne.p | avoid.y |
| S_Temperment_Corr_I_Adv | -0.07 | 0.199 | ne.p | avoid.y |
| S_Temperment_Corr_S_Adv | -0.17 | 0.071 | ne.p | avoid.y |
| S_Temperment_Corr_I_Factor | 0.03 | 0.654 | ne.p | avoid.y |
| **S_Temperment_Corr_S_Factor** | **-0.15** | **0.030** | **ne.p** | **avoid.y** |
| I_Adv_Corr_S_Adv | -0.40 | 0.011 | ne.p | avoid.y |
| I_Adv_Corr_I_Factor | 0.24 | 0.000 | ne.p | avoid.y |
| I_Adv_Corr_S_Factor | -0.11 | 0.112 | ne.p | avoid.y |
| S_Adv_Corr_I_Factor | -0.17 | 0.055 | ne.p | avoid.y |
| S_Adv_Corr_S_Factor | 0.46 | 0.000 | ne.p | avoid.y |
| I_Factor_Corr_S_Factor | -0.26 | 0.035 | ne.p | avoid.y |
| I_Temperment_Corr_S_Temperment | -0.24 | 0.008 | ec.p | PSS.y |
| I_Temperment_Corr_I_Adv | -0.30 | 0.000 | ec.p | PSS.y |
| I_Temperment_Corr_S_Adv | 0.18 | 0.013 | ec.p | PSS.y |
| I_Temperment_Corr_I_Factor | 0.15 | 0.001 | ec.p | PSS.y |
| I_Temperment_Corr_S_Factor | -0.05 | 0.274 | ec.p | PSS.y |
| S_Temperment_Corr_I_Adv | 0.01 | 0.837 | ec.p | PSS.y |
| S_Temperment_Corr_S_Adv | -0.30 | 0.003 | ec.p | PSS.y |
| S_Temperment_Corr_I_Factor | -0.02 | 0.748 | ec.p | PSS.y |
| **S_Temperment_Corr_S_Factor** | **0.07** | **0.190** | **ec.p** | **PSS.y** |
| I_Adv_Corr_S_Adv | -0.39 | 0.013 | ec.p | PSS.y |
| I_Adv_Corr_I_Factor | -0.07 | 0.171 | ec.p | PSS.y |
| I_Adv_Corr_S_Factor | -0.05 | 0.318 | ec.p | PSS.y |
| S_Adv_Corr_I_Factor | -0.05 | 0.447 | ec.p | PSS.y |
| S_Adv_Corr_S_Factor | -0.18 | 0.043 | ec.p | PSS.y |
| I_Factor_Corr_S_Factor | -0.49 | 0.000 | ec.p | PSS.y |
| I_Temperment_Corr_S_Temperment | -0.26 | 0.005 | ne.p | PSS.y |
| I_Temperment_Corr_I_Adv | -0.18 | 0.000 | ne.p | PSS.y |
| I_Temperment_Corr_S_Adv | 0.07 | 0.317 | ne.p | PSS.y |
| I_Temperment_Corr_I_Factor | 0.20 | 0.000 | ne.p | PSS.y |
| I_Temperment_Corr_S_Factor | -0.04 | 0.360 | ne.p | PSS.y |
| S_Temperment_Corr_I_Adv | -0.07 | 0.204 | ne.p | PSS.y |
| S_Temperment_Corr_S_Adv | -0.19 | 0.048 | ne.p | PSS.y |
| S_Temperment_Corr_I_Factor | -0.13 | 0.009 | ne.p | PSS.y |
| **S_Temperment_Corr_S_Factor** | **0.12** | **0.017** | **ne.p** | **PSS.y** |
| I_Adv_Corr_S_Adv | -0.39 | 0.012 | ne.p | PSS.y |
| I_Adv_Corr_I_Factor | -0.05 | 0.298 | ne.p | PSS.y |
| I_Adv_Corr_S_Factor | -0.06 | 0.249 | ne.p | PSS.y |
| S_Adv_Corr_I_Factor | -0.06 | 0.352 | ne.p | PSS.y |
| S_Adv_Corr_S_Factor | -0.17 | 0.047 | ne.p | PSS.y |
| I_Factor_Corr_S_Factor | -0.49 | 0.000 | ne.p | PSS.y |
| I_Temperment_Corr_S_Temperment | -0.24 | 0.009 | ec.p | ProSoc.y |
| I_Temperment_Corr_I_Adv | -0.30 | 0.000 | ec.p | ProSoc.y |
| I_Temperment_Corr_S_Adv | 0.19 | 0.012 | ec.p | ProSoc.y |
| I_Temperment_Corr_I_Factor | 0.06 | 0.200 | ec.p | ProSoc.y |
| I_Temperment_Corr_S_Factor | 0.05 | 0.288 | ec.p | ProSoc.y |
| S_Temperment_Corr_I_Adv | 0.01 | 0.837 | ec.p | ProSoc.y |
| S_Temperment_Corr_S_Adv | -0.31 | 0.003 | ec.p | ProSoc.y |
| S_Temperment_Corr_I_Factor | 0.00 | 0.974 | ec.p | ProSoc.y |
| **S_Temperment_Corr_S_Factor** | **-0.03** | **0.583** | **ec.p** | **ProSoc.y** |
| I_Adv_Corr_S_Adv | -0.38 | 0.021 | ec.p | ProSoc.y |
| I_Adv_Corr_I_Factor | -0.08 | 0.100 | ec.p | ProSoc.y |
| I_Adv_Corr_S_Factor | 0.04 | 0.439 | ec.p | ProSoc.y |
| S_Adv_Corr_I_Factor | 0.08 | 0.289 | ec.p | ProSoc.y |
| S_Adv_Corr_S_Factor | -0.04 | 0.628 | ec.p | ProSoc.y |
| I_Factor_Corr_S_Factor | -0.63 | 0.000 | ec.p | ProSoc.y |
| I_Temperment_Corr_S_Temperment | -0.27 | 0.004 | ne.p | ProSoc.y |
| I_Temperment_Corr_I_Adv | -0.18 | 0.000 | ne.p | ProSoc.y |
| I_Temperment_Corr_S_Adv | 0.07 | 0.357 | ne.p | ProSoc.y |
| I_Temperment_Corr_I_Factor | 0.05 | 0.260 | ne.p | ProSoc.y |
| I_Temperment_Corr_S_Factor | -0.02 | 0.630 | ne.p | ProSoc.y |
| S_Temperment_Corr_I_Adv | -0.08 | 0.188 | ne.p | ProSoc.y |
| S_Temperment_Corr_S_Adv | -0.18 | 0.067 | ne.p | ProSoc.y |
| S_Temperment_Corr_I_Factor | 0.06 | 0.274 | ne.p | ProSoc.y |
| **S_Temperment_Corr_S_Factor** | **-0.06** | **0.274** | **ne.p** | **ProSoc.y** |
| I_Adv_Corr_S_Adv | -0.39 | 0.017 | ne.p | ProSoc.y |
| I_Adv_Corr_I_Factor | -0.08 | 0.108 | ne.p | ProSoc.y |
| I_Adv_Corr_S_Factor | 0.05 | 0.384 | ne.p | ProSoc.y |
| S_Adv_Corr_I_Factor | 0.08 | 0.279 | ne.p | ProSoc.y |
| S_Adv_Corr_S_Factor | -0.07 | 0.441 | ne.p | ProSoc.y |
| I_Factor_Corr_S_Factor | -0.63 | 0.000 | ne.p | ProSoc.y |
| I_Temperment_Corr_S_Temperment | -0.24 | 0.011 | ec.p | SEQ.y |
| I_Temperment_Corr_I_Adv | -0.30 | 0.000 | ec.p | SEQ.y |
| I_Temperment_Corr_S_Adv | 0.18 | 0.013 | ec.p | SEQ.y |
| I_Temperment_Corr_I_Factor | -0.15 | 0.000 | ec.p | SEQ.y |
| I_Temperment_Corr_S_Factor | 0.03 | 0.519 | ec.p | SEQ.y |
| S_Temperment_Corr_I_Adv | 0.02 | 0.783 | ec.p | SEQ.y |
| S_Temperment_Corr_S_Adv | -0.31 | 0.002 | ec.p | SEQ.y |
| S_Temperment_Corr_I_Factor | -0.05 | 0.294 | ec.p | SEQ.y |
| S_Temperment_Corr_S_Factor | -0.09 | 0.108 | ec.p | SEQ.y |
| I_Adv_Corr_S_Adv | -0.39 | 0.012 | ec.p | SEQ.y |
| I_Adv_Corr_I_Factor | 0.29 | 0.000 | ec.p | SEQ.y |
| I_Adv_Corr_S_Factor | -0.09 | 0.073 | ec.p | SEQ.y |
| S_Adv_Corr_I_Factor | -0.17 | 0.012 | ec.p | SEQ.y |
| S_Adv_Corr_S_Factor | 0.37 | 0.000 | ec.p | SEQ.y |
| I_Factor_Corr_S_Factor | -0.51 | 0.000 | ec.p | SEQ.y |
| I_Temperment_Corr_S_Temperment | -0.27 | 0.005 | ne.p | SEQ.y |
| I_Temperment_Corr_I_Adv | -0.18 | 0.000 | ne.p | SEQ.y |
| I_Temperment_Corr_S_Adv | 0.07 | 0.347 | ne.p | SEQ.y |
| I_Temperment_Corr_I_Factor | -0.13 | 0.002 | ne.p | SEQ.y |
| I_Temperment_Corr_S_Factor | 0.01 | 0.904 | ne.p | SEQ.y |
| S_Temperment_Corr_I_Adv | -0.07 | 0.203 | ne.p | SEQ.y |
| S_Temperment_Corr_S_Adv | -0.18 | 0.054 | ne.p | SEQ.y |
| S_Temperment_Corr_I_Factor | -0.05 | 0.299 | ne.p | SEQ.y |
| S_Temperment_Corr_S_Factor | -0.08 | 0.135 | ne.p | SEQ.y |
| I_Adv_Corr_S_Adv | -0.39 | 0.011 | ne.p | SEQ.y |
| I_Adv_Corr_I_Factor | 0.29 | 0.000 | ne.p | SEQ.y |
| I_Adv_Corr_S_Factor | -0.09 | 0.071 | ne.p | SEQ.y |
| S_Adv_Corr_I_Factor | -0.18 | 0.009 | ne.p | SEQ.y |
| S_Adv_Corr_S_Factor | 0.36 | 0.000 | ne.p | SEQ.y |
| I_Factor_Corr_S_Factor | -0.51 | 0.000 | ne.p | SEQ.y |

*Note*: “Factor” refers to the exploratory third variable.

# Table S14. Regression of Personality Intercept and Slope on Adversity and Third Variable

| Parameter | Estimate | SE | PValue | StdAll | Personality Construct | Factor | ModelName |
| --- | --- | --- | --- | --- | --- | --- | --- |
| S_Temperment_ON_I_Adv | -0.04 | 0.09 | 0.673 | -0.04 | ec.p | anx.y | tri_adv_ec.p_anx.y |
| S_Temperment_ON_S_Adv | -0.77 | 0.49 | 0.113 | -0.44 | ec.p | anx.y | tri_adv_ec.p_anx.y |
| S_Temperment_ON_I_Factor | 0.14 | 0.07 | 0.037 | 0.27 | ec.p | anx.y | tri_adv_ec.p_anx.y |
| S_Temperment_ON_S_Factor | -0.16 | 0.07 | 0.024 | -0.36 | ec.p | anx.y | tri_adv_ec.p_anx.y |
| S_Temperment_ON_Cohort | -0.05 | 0.05 | 0.369 | -0.13 | ec.p | anx.y | tri_adv_ec.p_anx.y |
| I_Temperment_ON_I_Adv | -0.47 | 0.09 | 0.000 | -0.28 | ec.p | anx.y | tri_adv_ec.p_anx.y |
| I_Temperment_ON_I_Factor | -0.30 | 0.07 | 0.000 | -0.32 | ec.p | anx.y | tri_adv_ec.p_anx.y |
| I_Temperment_ON_Cohort | 0.13 | 0.03 | 0.000 | 0.20 | ec.p | anx.y | tri_adv_ec.p_anx.y |
| S_Temperment_ON_I_Adv | -0.10 | 0.09 | 0.226 | -0.12 | ne.p | anx.y | tri_adv_ne.p_anx.y |
| S_Temperment_ON_S_Adv | -0.72 | 0.48 | 0.138 | -0.44 | ne.p | anx.y | tri_adv_ne.p_anx.y |
| S_Temperment_ON_I_Factor | 0.01 | 0.06 | 0.866 | 0.02 | ne.p | anx.y | tri_adv_ne.p_anx.y |
| S_Temperment_ON_S_Factor | -0.01 | 0.06 | 0.920 | -0.01 | ne.p | anx.y | tri_adv_ne.p_anx.y |
| S_Temperment_ON_Cohort | -0.02 | 0.05 | 0.733 | -0.05 | ne.p | anx.y | tri_adv_ne.p_anx.y |
| I_Temperment_ON_I_Adv | -0.23 | 0.08 | 0.004 | -0.16 | ne.p | anx.y | tri_adv_ne.p_anx.y |
| I_Temperment_ON_I_Factor | -0.31 | 0.06 | 0.000 | -0.40 | ne.p | anx.y | tri_adv_ne.p_anx.y |
| I_Temperment_ON_Cohort | 0.11 | 0.03 | 0.000 | 0.20 | ne.p | anx.y | tri_adv_ne.p_anx.y |
| S_Temperment_ON_I_Adv | -0.02 | 0.09 | 0.847 | -0.02 | ec.p | avoid.y | tri_adv_ec.p_avoid.y |
| S_Temperment_ON_S_Adv | -0.77 | 0.48 | 0.108 | -0.46 | ec.p | avoid.y | tri_adv_ec.p_avoid.y |
| S_Temperment_ON_I_Factor | 0.04 | 0.03 | 0.147 | 0.14 | ec.p | avoid.y | tri_adv_ec.p_avoid.y |
| S_Temperment_ON_S_Factor | -0.07 | 0.03 | 0.043 | -0.23 | ec.p | avoid.y | tri_adv_ec.p_avoid.y |
| S_Temperment_ON_Cohort | -0.09 | 0.05 | 0.051 | -0.27 | ec.p | avoid.y | tri_adv_ec.p_avoid.y |
| I_Temperment_ON_I_Adv | -0.53 | 0.09 | 0.000 | -0.32 | ec.p | avoid.y | tri_adv_ec.p_avoid.y |
| I_Temperment_ON_I_Factor | -0.08 | 0.03 | 0.005 | -0.16 | ec.p | avoid.y | tri_adv_ec.p_avoid.y |
| I_Temperment_ON_Cohort | 0.18 | 0.03 | 0.000 | 0.28 | ec.p | avoid.y | tri_adv_ec.p_avoid.y |
| S_Temperment_ON_I_Adv | -0.10 | 0.08 | 0.221 | -0.11 | ne.p | avoid.y | tri_adv_ne.p_avoid.y |
| S_Temperment_ON_S_Adv | -0.56 | 0.44 | 0.205 | -0.33 | ne.p | avoid.y | tri_adv_ne.p_avoid.y |
| S_Temperment_ON_I_Factor | 0.03 | 0.03 | 0.359 | 0.09 | ne.p | avoid.y | tri_adv_ne.p_avoid.y |
| S_Temperment_ON_S_Factor | -0.07 | 0.03 | 0.025 | -0.25 | ne.p | avoid.y | tri_adv_ne.p_avoid.y |
| S_Temperment_ON_Cohort | -0.01 | 0.04 | 0.769 | -0.04 | ne.p | avoid.y | tri_adv_ne.p_avoid.y |
| I_Temperment_ON_I_Adv | -0.30 | 0.08 | 0.000 | -0.21 | ne.p | avoid.y | tri_adv_ne.p_avoid.y |
| I_Temperment_ON_I_Factor | -0.06 | 0.03 | 0.027 | -0.13 | ne.p | avoid.y | tri_adv_ne.p_avoid.y |
| I_Temperment_ON_Cohort | 0.15 | 0.03 | 0.000 | 0.29 | ne.p | avoid.y | tri_adv_ne.p_avoid.y |
| S_Temperment_ON_I_Adv | 0.00 | 0.09 | 0.971 | 0.00 | ec.p | PSS.y | tri_adv_ec.p_PSS.y |
| S_Temperment_ON_S_Adv | -0.88 | 0.50 | 0.078 | -0.53 | ec.p | PSS.y | tri_adv_ec.p_PSS.y |
| S_Temperment_ON_I_Factor | -0.02 | 0.14 | 0.884 | -0.01 | ec.p | PSS.y | tri_adv_ec.p_PSS.y |
| S_Temperment_ON_S_Factor | 0.09 | 0.13 | 0.503 | 0.05 | ec.p | PSS.y | tri_adv_ec.p_PSS.y |
| S_Temperment_ON_Cohort | -0.09 | 0.05 | 0.057 | -0.26 | ec.p | PSS.y | tri_adv_ec.p_PSS.y |
| I_Temperment_ON_I_Adv | -0.56 | 0.09 | 0.000 | -0.33 | ec.p | PSS.y | tri_adv_ec.p_PSS.y |
| I_Temperment_ON_I_Factor | 0.41 | 0.15 | 0.005 | 0.13 | ec.p | PSS.y | tri_adv_ec.p_PSS.y |
| I_Temperment_ON_Cohort | 0.16 | 0.03 | 0.000 | 0.26 | ec.p | PSS.y | tri_adv_ec.p_PSS.y |
| S_Temperment_ON_I_Adv | -0.10 | 0.08 | 0.216 | -0.11 | ne.p | PSS.y | tri_adv_ne.p_PSS.y |
| S_Temperment_ON_S_Adv | -0.70 | 0.46 | 0.134 | -0.40 | ne.p | PSS.y | tri_adv_ne.p_PSS.y |
| S_Temperment_ON_I_Factor | -0.29 | 0.13 | 0.028 | -0.16 | ne.p | PSS.y | tri_adv_ne.p_PSS.y |
| S_Temperment_ON_S_Factor | 0.13 | 0.12 | 0.271 | 0.08 | ne.p | PSS.y | tri_adv_ne.p_PSS.y |
| S_Temperment_ON_Cohort | -0.02 | 0.04 | 0.662 | -0.06 | ne.p | PSS.y | tri_adv_ne.p_PSS.y |
| I_Temperment_ON_I_Adv | -0.31 | 0.08 | 0.000 | -0.21 | ne.p | PSS.y | tri_adv_ne.p_PSS.y |
| I_Temperment_ON_I_Factor | 0.56 | 0.12 | 0.000 | 0.21 | ne.p | PSS.y | tri_adv_ne.p_PSS.y |
| I_Temperment_ON_Cohort | 0.14 | 0.02 | 0.000 | 0.27 | ne.p | PSS.y | tri_adv_ne.p_PSS.y |
| S_Temperment_ON_I_Adv | 0.00 | 0.09 | 0.978 | 0.00 | ec.p | ProSoc.y | tri_adv_ec.p_ProSoc.y |
| S_Temperment_ON_S_Adv | -0.93 | 0.52 | 0.074 | -0.56 | ec.p | ProSoc.y | tri_adv_ec.p_ProSoc.y |
| S_Temperment_ON_I_Factor | 0.00 | 0.02 | 0.916 | 0.01 | ec.p | ProSoc.y | tri_adv_ec.p_ProSoc.y |
| S_Temperment_ON_S_Factor | 0.00 | 0.02 | 0.934 | 0.01 | ec.p | ProSoc.y | tri_adv_ec.p_ProSoc.y |
| S_Temperment_ON_Cohort | -0.09 | 0.05 | 0.056 | -0.28 | ec.p | ProSoc.y | tri_adv_ec.p_ProSoc.y |
| I_Temperment_ON_I_Adv | -0.57 | 0.09 | 0.000 | -0.34 | ec.p | ProSoc.y | tri_adv_ec.p_ProSoc.y |
| I_Temperment_ON_I_Factor | 0.02 | 0.02 | 0.300 | 0.05 | ec.p | ProSoc.y | tri_adv_ec.p_ProSoc.y |
| I_Temperment_ON_Cohort | 0.16 | 0.03 | 0.000 | 0.25 | ec.p | ProSoc.y | tri_adv_ec.p_ProSoc.y |
| S_Temperment_ON_I_Adv | -0.09 | 0.08 | 0.299 | -0.10 | ne.p | ProSoc.y | tri_adv_ne.p_ProSoc.y |
| S_Temperment_ON_S_Adv | -0.76 | 0.49 | 0.119 | -0.46 | ne.p | ProSoc.y | tri_adv_ne.p_ProSoc.y |
| S_Temperment_ON_I_Factor | 0.01 | 0.02 | 0.519 | 0.06 | ne.p | ProSoc.y | tri_adv_ne.p_ProSoc.y |
| S_Temperment_ON_S_Factor | 0.00 | 0.02 | 0.820 | -0.02 | ne.p | ProSoc.y | tri_adv_ne.p_ProSoc.y |
| S_Temperment_ON_Cohort | -0.02 | 0.05 | 0.612 | -0.07 | ne.p | ProSoc.y | tri_adv_ne.p_ProSoc.y |
| I_Temperment_ON_I_Adv | -0.33 | 0.08 | 0.000 | -0.23 | ne.p | ProSoc.y | tri_adv_ne.p_ProSoc.y |
| I_Temperment_ON_I_Factor | 0.02 | 0.02 | 0.247 | 0.05 | ne.p | ProSoc.y | tri_adv_ne.p_ProSoc.y |
| I_Temperment_ON_Cohort | 0.14 | 0.03 | 0.000 | 0.26 | ne.p | ProSoc.y | tri_adv_ne.p_ProSoc.y |
| S_Temperment_ON_I_Adv | 0.05 | 0.09 | 0.596 | 0.05 | ec.p | SEQ.y | tri_adv_ec.p_SEQ.y |
| S_Temperment_ON_S_Adv | -0.89 | 0.53 | 0.094 | -0.53 | ec.p | SEQ.y | tri_adv_ec.p_SEQ.y |
| S_Temperment_ON_I_Factor | -0.10 | 0.04 | 0.029 | -0.17 | ec.p | SEQ.y | tri_adv_ec.p_SEQ.y |
| S_Temperment_ON_S_Factor | -0.10 | 0.04 | 0.017 | -0.19 | ec.p | SEQ.y | tri_adv_ec.p_SEQ.y |
| S_Temperment_ON_Cohort | -0.09 | 0.05 | 0.085 | -0.25 | ec.p | SEQ.y | tri_adv_ec.p_SEQ.y |
| I_Temperment_ON_I_Adv | -0.53 | 0.09 | 0.000 | -0.32 | ec.p | SEQ.y | tri_adv_ec.p_SEQ.y |
| I_Temperment_ON_I_Factor | -0.10 | 0.04 | 0.021 | -0.10 | ec.p | SEQ.y | tri_adv_ec.p_SEQ.y |
| I_Temperment_ON_Cohort | 0.16 | 0.03 | 0.000 | 0.25 | ec.p | SEQ.y | tri_adv_ec.p_SEQ.y |
| S_Temperment_ON_I_Adv | -0.06 | 0.09 | 0.516 | -0.06 | ne.p | SEQ.y | tri_adv_ne.p_SEQ.y |
| S_Temperment_ON_S_Adv | -0.70 | 0.49 | 0.153 | -0.42 | ne.p | SEQ.y | tri_adv_ne.p_SEQ.y |
| S_Temperment_ON_I_Factor | -0.08 | 0.04 | 0.050 | -0.16 | ne.p | SEQ.y | tri_adv_ne.p_SEQ.y |
| S_Temperment_ON_S_Factor | -0.10 | 0.04 | 0.012 | -0.20 | ne.p | SEQ.y | tri_adv_ne.p_SEQ.y |
| S_Temperment_ON_Cohort | -0.01 | 0.05 | 0.799 | -0.04 | ne.p | SEQ.y | tri_adv_ne.p_SEQ.y |
| I_Temperment_ON_I_Adv | -0.29 | 0.08 | 0.000 | -0.21 | ne.p | SEQ.y | tri_adv_ne.p_SEQ.y |
| I_Temperment_ON_I_Factor | -0.09 | 0.04 | 0.017 | -0.11 | ne.p | SEQ.y | tri_adv_ne.p_SEQ.y |
| I_Temperment_ON_Cohort | 0.13 | 0.02 | 0.000 | 0.26 | ne.p | SEQ.y | tri_adv_ne.p_SEQ.y |

*Note*: “Factor” refers to the exploratory third variable.

# Domain Level Adversity Robustness Analyses

As a robustness check, we examined how our main results differed when we examined each of the individual 16 adversity domains in isolation. First, we ran univariate unconditional latent growth curve models for each of the adversity domains. Model fit indices are reported in Table S15 and model parameters of interest are reported in Table S16. The variance of the slope parameter was only significant for the domains of parent-child relationship stress (“parentchild.relationship.adv”), stress related to the relationship between the youth’s parents (“parentparent.relationship.adv”) and discrimination/acculturation stress (“discrimination.adv”), which should be kept in mind when interpreting additional output.

Though the slope variance was mostly non-significant, indicating that reliable individual differences in change were not present at the level of individual adversity domains, we still computed bivariate latent growth curve models for all adversity domains and parent and youth-reported effortful control and emotional stability to get a comprehensive picture of how the size of the slope-slope correlations compared to what we found in the main analyses. Model fit indices are reported in Table S17 and model parameters of interest are reported in Table S18. All of the bivariate models that used body image (referred to as “body” in the output) have negative variances for the body image slope and should be dis-regarded.

There was negative correlated change between parent-child relationship stress and youth-reported effortful control (*r* = -0.39, *p* = .003) and emotional stability (*r* = -0.39, *p* = .002). The variance of the parent-child relationship stress slopes was consistently significant in all models. There was no correlated change for the other two domains where the slope variance was initially significant. There were also consistent patterns of correlated change for academic stress, peer stress, and violence. Though these estimates should be interpreted cautiously, we highlight them here as potentially interesting direction for future research. Change in academic stress was negatively associated with growth in youth and parent reported effortful control (*r* = **-0.79**, *p* < .**000**; *r* = **-0.50**, *p* < .**000**) and emotional stability (*r* = **-0.57**, *p* < .**000**; *r* = **-0.29**, *p* = .**008**). Change in peer stress was negatively associated with growth in youth and parent-reported effortful control (*r* = **-0.29**, *p* = **.022**; *r* = **-0.43**, *p* < .**000**) and emotional stability (*r* = **-0.51**, *p* = .**001**; *r* = **-0.25**, *p* = **.026**). Change in stress from exposure to violence was negatively associated with growth in youth and parent reported effortful control (*r* = **-0.37**, *p* = .**025**; *r* = **-0.31**, *p* = **.026**) and parent-reported emotional stability (*r* = **-0.31**, *p* = **.029**).

Because of the significant slope variance was limited to just three domains, we decided to only run further analyses for RQs 3 and 4 with those domains. Fit indices for the supplemental RQ 3 models are reported in Tables S19a-c.The proportions on participants who grew in low-adversity, initial adversity, and increasing adversity conditions are reported for parent-child relationship stress (Table S20a), parent-parent relationship stress (Table S20b), and discrimination/acculturation stress (Table S20c). We also re-ran the trivariate regression models for each of these three adversity domains. The fit indices for the models are reported in Table S21, and the model parameters are reported in Table S22.

# Table S15. Adversity Domain Univariate Latent Growth Curve Model Fit

| chisq | df | rmsea | tli | cfi | ModID |
| --- | --- | --- | --- | --- | --- |
| 7.838 | 1 | 0.102 | 0.904 | 0.968 | academic.adv |
| 7.764 | 1 | 0.101 | 0.836 | 0.945 | behavioral.adv |
| 0.815 | 1 | 0 | 1.002 | 1 | peer.adv |
| 2.284 | 1 | 0.044 | 0.986 | 0.995 | parentchild.relationship.adv |
| 0.718 | 1 | 0 | 1.004 | 1 | parentparent.relationship.adv |
| 0.53 | 1 | 0 | 1.008 | 1 | household.relationship.adv |
| 1.061 | 1 | 0.01 | 0.999 | 1 | body.adv |
| 2.54 | 1 | 0.048 | 0.98 | 0.993 | romantic.adv |
| 1.711 | 1 | 0.033 | 0.986 | 0.995 | neighborhood.adv |
| 4.779 | 1 | 0.075 | 0.907 | 0.969 | health.self.adv |
| 0.022 | 1 | 0 | 1.031 | 1 | health.other.adv |
| 9.591 | 1 | 0.114 | 0.889 | 0.963 | finance.adv |
| 12.068 | 1 | 0.129 | 0.494 | 0.831 | legal.adv |
| 4.81 | 1 | 0.076 | 0.879 | 0.96 | violence.adv |
| 0.377 | 1 | 0 | 1.013 | 1 | discrimination.adv |
| 0.132 | 1 | 0 | 1.056 | 1 | activities.adv |

# Table S16. Adversity Domain Univariate Latent Growth Curve Model Parameters

| Parameter | Estimate | SE | PValue | StdAll | Model |
| --- | --- | --- | --- | --- | --- |
| AdvInterceptOfIntercept | 1.79 | 0.03 | 0.000 | 3.46 | academic.adv |
| AdvVarianceOfIntercept | 0.27 | 0.05 | 0.000 | 1.00 | academic.adv |
| AdvInterceptOfSlope | 0.05 | 0.04 | 0.147 | 0.14 | academic.adv |
| AdvVarianceOfSlope | 0.14 | 0.10 | 0.160 | 1.00 | academic.adv |
| AdvInterceptOfIntercept | 1.42 | 0.03 | 0.000 | 3.19 | behavioral.adv |
| AdvVarianceOfIntercept | 0.20 | 0.04 | 0.000 | 1.00 | behavioral.adv |
| AdvInterceptOfSlope | -0.25 | 0.03 | 0.000 | -1.21 | behavioral.adv |
| AdvVarianceOfSlope | 0.04 | 0.07 | 0.561 | 1.00 | behavioral.adv |
| AdvInterceptOfIntercept | 1.90 | 0.03 | 0.000 | 3.54 | peer.adv |
| AdvVarianceOfIntercept | 0.29 | 0.05 | 0.000 | 1.00 | peer.adv |
| AdvInterceptOfSlope | -0.22 | 0.04 | 0.000 | -0.57 | peer.adv |
| AdvVarianceOfSlope | 0.14 | 0.09 | 0.097 | 1.00 | peer.adv |
| AdvInterceptOfIntercept | 1.95 | 0.03 | 0.000 | 4.08 | parentchild.relationship.adv |
| AdvVarianceOfIntercept | 0.23 | 0.04 | 0.000 | 1.00 | parentchild.relationship.adv |
| AdvInterceptOfSlope | -0.05 | 0.04 | 0.159 | -0.11 | parentchild.relationship.adv |
| **AdvVarianceOfSlope** | **0.21** | **0.09** | **0.025** | **1.00** | **parentchild.relationship.adv** |
| AdvInterceptOfIntercept | 1.84 | 0.03 | 0.000 | 3.05 | parentparent.relationship.adv |
| AdvVarianceOfIntercept | 0.36 | 0.06 | 0.000 | 1.00 | parentparent.relationship.adv |
| AdvInterceptOfSlope | -0.11 | 0.04 | 0.009 | -0.16 | parentparent.relationship.adv |
| **AdvVarianceOfSlope** | **0.43** | **0.11** | **0.000** | **1.00** | **parentparent.relationship.adv** |
| AdvInterceptOfIntercept | 2.06 | 0.03 | 0.000 | 4.43 | household.relationship.adv |
| AdvVarianceOfIntercept | 0.22 | 0.04 | 0.000 | 1.00 | household.relationship.adv |
| AdvInterceptOfSlope | -0.14 | 0.04 | 0.000 | -0.46 | household.relationship.adv |
| AdvVarianceOfSlope | 0.09 | 0.09 | 0.327 | 1.00 | household.relationship.adv |
| AdvInterceptOfIntercept | 1.59 | 0.03 | 0.000 | 3.66 | body.adv |
| AdvVarianceOfIntercept | 0.19 | 0.04 | 0.000 | 1.00 | body.adv |
| AdvInterceptOfSlope | 0.00 | 0.03 | 0.998 | NA | body.adv |
| AdvVarianceOfSlope | -0.02 | 0.09 | 0.784 | NA | body.adv |
| AdvInterceptOfIntercept | 1.38 | 0.02 | 0.000 | 3.40 | romantic.adv |
| AdvVarianceOfIntercept | 0.16 | 0.03 | 0.000 | 1.00 | romantic.adv |
| AdvInterceptOfSlope | 0.03 | 0.03 | 0.292 | 0.12 | romantic.adv |
| AdvVarianceOfSlope | 0.06 | 0.06 | 0.324 | 1.00 | romantic.adv |
| AdvInterceptOfIntercept | 1.52 | 0.03 | 0.000 | 3.50 | neighborhood.adv |
| AdvVarianceOfIntercept | 0.19 | 0.04 | 0.000 | 1.00 | neighborhood.adv |
| AdvInterceptOfSlope | 0.02 | 0.03 | 0.551 | 0.07 | neighborhood.adv |
| AdvVarianceOfSlope | 0.08 | 0.08 | 0.307 | 1.00 | neighborhood.adv |
| AdvInterceptOfIntercept | 1.49 | 0.03 | 0.000 | 3.81 | health.self.adv |
| AdvVarianceOfIntercept | 0.15 | 0.04 | 0.000 | 1.00 | health.self.adv |
| AdvInterceptOfSlope | -0.10 | 0.03 | 0.006 | -0.63 | health.self.adv |
| AdvVarianceOfSlope | 0.02 | 0.08 | 0.776 | 1.00 | health.self.adv |
| AdvInterceptOfIntercept | 2.08 | 0.04 | 0.000 | 3.41 | health.other.adv |
| AdvVarianceOfIntercept | 0.37 | 0.09 | 0.000 | 1.00 | health.other.adv |
| AdvInterceptOfSlope | -0.26 | 0.05 | 0.000 | -0.55 | health.other.adv |
| AdvVarianceOfSlope | 0.23 | 0.18 | 0.205 | 1.00 | health.other.adv |
| AdvInterceptOfIntercept | 1.53 | 0.03 | 0.000 | 3.66 | finance.adv |
| AdvVarianceOfIntercept | 0.17 | 0.04 | 0.000 | 1.00 | finance.adv |
| AdvInterceptOfSlope | -0.07 | 0.04 | 0.076 | -0.16 | finance.adv |
| AdvVarianceOfSlope | 0.18 | 0.10 | 0.060 | 1.00 | finance.adv |
| AdvInterceptOfIntercept | 1.54 | 0.03 | 0.000 | 4.04 | legal.adv |
| AdvVarianceOfIntercept | 0.14 | 0.06 | 0.012 | 1.00 | legal.adv |
| AdvInterceptOfSlope | -0.19 | 0.04 | 0.000 | -0.64 | legal.adv |
| AdvVarianceOfSlope | 0.09 | 0.12 | 0.482 | 1.00 | legal.adv |
| AdvInterceptOfIntercept | 1.43 | 0.03 | 0.000 | 3.72 | violence.adv |
| AdvVarianceOfIntercept | 0.15 | 0.04 | 0.000 | 1.00 | violence.adv |
| AdvInterceptOfSlope | -0.23 | 0.03 | 0.000 | -0.79 | violence.adv |
| AdvVarianceOfSlope | 0.08 | 0.08 | 0.313 | 1.00 | violence.adv |
| AdvInterceptOfIntercept | 1.22 | 0.02 | 0.000 | 5.05 | discrimination.adv |
| AdvVarianceOfIntercept | 0.06 | 0.02 | 0.002 | 1.00 | discrimination.adv |
| AdvInterceptOfSlope | -0.01 | 0.03 | 0.833 | -0.01 | discrimination.adv |
| **AdvVarianceOfSlope** | **0.14** | **0.04** | **0.001** | **1.00** | **discrimination.adv** |
| AdvInterceptOfIntercept | 1.35 | 0.02 | 0.000 | 5.91 | activities.adv |
| AdvVarianceOfIntercept | 0.05 | 0.02 | 0.010 | 1.00 | activities.adv |
| AdvInterceptOfSlope | -0.10 | 0.02 | 0.000 | -1.45 | activities.adv |
| AdvVarianceOfSlope | 0.00 | 0.04 | 0.909 | 1.00 | activities.adv |

# Table S17. Adversity Domain Bivariate Latent Growth Curve Model Fit

| chisq | df | rmsea | tli | cfi | ModID |
| --- | --- | --- | --- | --- | --- |
| 39.678 | 7 | 0.083 | 0.927 | 0.966 | academic.adv_ec.y |
| 41.195 | 7 | 0.085 | 0.898 | 0.952 | behavioral.adv_ec.y |
| 34.55 | 7 | 0.076 | 0.928 | 0.966 | peer.adv_ec.y |
| 50.21 | 7 | 0.095 | 0.901 | 0.954 | parentchild.relationship.adv_ec.y |
| 33.672 | 7 | 0.075 | 0.93 | 0.967 | parentparent.relationship.adv_ec.y |
| 40.118 | 7 | 0.083 | 0.908 | 0.957 | household.relationship.adv_ec.y |
| 27.244 | 7 | 0.065 | 0.949 | 0.976 | body.adv_ec.y |
| 44.915 | 7 | 0.089 | 0.903 | 0.955 | romantic.adv_ec.y |
| 30.124 | 7 | 0.07 | 0.932 | 0.968 | neighborhood.adv_ec.y |
| 31.709 | 7 | 0.072 | 0.922 | 0.963 | health.self.adv_ec.y |
| 29.581 | 7 | 0.069 | 0.926 | 0.965 | health.other.adv_ec.y |
| 40.516 | 7 | 0.084 | 0.913 | 0.959 | finance.adv_ec.y |
| 36.487 | 7 | 0.079 | 0.9 | 0.953 | legal.adv_ec.y |
| 39.232 | 7 | 0.082 | 0.896 | 0.952 | violence.adv_ec.y |
| 32.419 | 7 | 0.073 | 0.924 | 0.964 | discrimination.adv_ec.y |
| 25.663 | 7 | 0.063 | 0.935 | 0.97 | activities.adv_ec.y |
| 42.416 | 7 | 0.086 | 0.948 | 0.976 | academic.adv_ec.p |
| 40.8 | 7 | 0.084 | 0.943 | 0.973 | behavioral.adv_ec.p |
| 30.474 | 7 | 0.07 | 0.963 | 0.983 | peer.adv_ec.p |
| 29.86 | 7 | 0.069 | 0.965 | 0.984 | parentchild.relationship.adv_ec.p |
| 33.207 | 7 | 0.074 | 0.958 | 0.98 | parentparent.relationship.adv_ec.p |
| 25.034 | 7 | 0.062 | 0.97 | 0.986 | household.relationship.adv_ec.p |
| 30.887 | 7 | 0.071 | 0.962 | 0.982 | body.adv_ec.p |
| 29.322 | 7 | 0.068 | 0.964 | 0.983 | romantic.adv_ec.p |
| 30.17 | 7 | 0.07 | 0.96 | 0.981 | neighborhood.adv_ec.p |
| 31.089 | 7 | 0.071 | 0.957 | 0.98 | health.self.adv_ec.p |
| 25.353 | 7 | 0.062 | 0.967 | 0.984 | health.other.adv_ec.p |
| 38.655 | 7 | 0.082 | 0.949 | 0.976 | finance.adv_ec.p |
| 38.576 | 7 | 0.081 | 0.941 | 0.973 | legal.adv_ec.p |
| 30.452 | 7 | 0.07 | 0.958 | 0.98 | violence.adv_ec.p |
| 25.362 | 7 | 0.062 | 0.968 | 0.985 | discrimination.adv_ec.p |
| 29.701 | 7 | 0.069 | 0.957 | 0.98 | activities.adv_ec.p |
| 84.274 | 7 | 0.127 | 0.709 | 0.864 | academic.adv_ne.y |
| 80.949 | 7 | 0.124 | 0.647 | 0.835 | behavioral.adv_ne.y |
| 80.03 | 7 | 0.124 | 0.747 | 0.882 | peer.adv_ne.y |
| 85.877 | 7 | 0.129 | 0.744 | 0.881 | parentchild.relationship.adv_ne.y |
| 74.539 | 7 | 0.119 | 0.742 | 0.879 | parentparent.relationship.adv_ne.y |
| 80.512 | 7 | 0.124 | 0.704 | 0.862 | household.relationship.adv_ne.y |
| 79.196 | 7 | 0.123 | 0.756 | 0.886 | body.adv_ne.y |
| 86.497 | 7 | 0.129 | 0.701 | 0.86 | romantic.adv_ne.y |
| 87.191 | 7 | 0.13 | 0.654 | 0.838 | neighborhood.adv_ne.y |
| 82.068 | 7 | 0.125 | 0.638 | 0.831 | health.self.adv_ne.y |
| 73.485 | 7 | 0.118 | 0.656 | 0.839 | health.other.adv_ne.y |
| 84.818 | 7 | 0.128 | 0.709 | 0.864 | finance.adv_ne.y |
| 85.272 | 7 | 0.128 | 0.58 | 0.804 | legal.adv_ne.y |
| 86.084 | 7 | 0.129 | 0.61 | 0.818 | violence.adv_ne.y |
| 74.453 | 7 | 0.119 | 0.696 | 0.858 | discrimination.adv_ne.y |
| 73.298 | 7 | 0.118 | 0.637 | 0.83 | activities.adv_ne.y |
| 58.544 | 7 | 0.104 | 0.904 | 0.955 | academic.adv_ne.p |
| 68.35 | 7 | 0.114 | 0.876 | 0.942 | behavioral.adv_ne.p |
| 45.931 | 7 | 0.09 | 0.928 | 0.966 | peer.adv_ne.p |
| 50.508 | 7 | 0.096 | 0.923 | 0.964 | parentchild.relationship.adv_ne.p |
| 44.06 | 7 | 0.088 | 0.93 | 0.967 | parentparent.relationship.adv_ne.p |
| 48.272 | 7 | 0.093 | 0.921 | 0.963 | household.relationship.adv_ne.p |
| 45.715 | 7 | 0.09 | 0.928 | 0.966 | body.adv_ne.p |
| 47.446 | 7 | 0.092 | 0.923 | 0.964 | romantic.adv_ne.p |
| 42.33 | 7 | 0.086 | 0.928 | 0.966 | neighborhood.adv_ne.p |
| 55.755 | 7 | 0.101 | 0.898 | 0.953 | health.self.adv_ne.p |
| 49.012 | 7 | 0.094 | 0.91 | 0.958 | health.other.adv_ne.p |
| 60.347 | 7 | 0.106 | 0.9 | 0.953 | finance.adv_ne.p |
| 57.84 | 7 | 0.103 | 0.887 | 0.947 | legal.adv_ne.p |
| 50.17 | 7 | 0.095 | 0.908 | 0.957 | violence.adv_ne.p |
| 41.192 | 7 | 0.085 | 0.93 | 0.967 | discrimination.adv_ne.p |
| 51.942 | 7 | 0.097 | 0.899 | 0.953 | activities.adv_ne.p |

# Table S18. Adversity Domain Bivariate Latent Growth Curve Model Parameters

| Parameter | Estimate | SE | PValue | StdAll | Construct | Adv |
| --- | --- | --- | --- | --- | --- | --- |
| ConstructInterceptOfIntercept | 3.44 | 0.02 | 0.000 | 7.59 | ec.y | academic.adv |
| ConstructVarianceOfIntercept | 0.21 | 0.02 | 0.000 | 1.00 | ec.y | academic.adv |
| ConstructInterceptOfSlope | 0.09 | 0.02 | 0.000 | 0.23 | ec.y | academic.adv |
| ConstructVarianceOfSlope | 0.15 | 0.04 | 0.000 | 1.00 | ec.y | academic.adv |
| AdvInterceptOfIntercept | 1.79 | 0.03 | 0.000 | 3.52 | ec.y | academic.adv |
| AdvVarianceOfIntercept | 0.26 | 0.05 | 0.000 | 1.00 | ec.y | academic.adv |
| AdvInterceptOfSlope | 0.05 | 0.04 | 0.158 | 0.14 | ec.y | academic.adv |
| AdvVarianceOfSlope | 0.13 | 0.09 | 0.161 | 1.00 | ec.y | academic.adv |
| Cnsrct.Intercept_Corr_Adv_Intercept | -0.15 | 0.02 | 0.000 | -0.65 | ec.y | academic.adv |
| Cnsrct.Intercept_Corr_Adv_Slope | 0.03 | 0.02 | 0.082 | 0.21 | ec.y | academic.adv |
| **Cnsrct.Slope_Corr_Adv_Intercept** | **0.05** | **0.02** | **0.003** | **0.26** | **ec.y** | **academic.adv** |
| **Cnsrct.Slope_Corr_Adv_Slope** | **-0.11** | **0.02** | **0.000** | **-0.79** | **ec.y** | **academic.adv** |
| ConstructInterceptOfIntercept | 3.44 | 0.02 | 0.000 | 7.53 | ec.y | behavioral.adv |
| ConstructVarianceOfIntercept | 0.21 | 0.02 | 0.000 | 1.00 | ec.y | behavioral.adv |
| ConstructInterceptOfSlope | 0.09 | 0.02 | 0.000 | 0.23 | ec.y | behavioral.adv |
| ConstructVarianceOfSlope | 0.16 | 0.04 | 0.000 | 1.00 | ec.y | behavioral.adv |
| AdvInterceptOfIntercept | 1.42 | 0.03 | 0.000 | 3.14 | ec.y | behavioral.adv |
| AdvVarianceOfIntercept | 0.20 | 0.04 | 0.000 | 1.00 | ec.y | behavioral.adv |
| AdvInterceptOfSlope | -0.25 | 0.03 | 0.000 | -1.03 | ec.y | behavioral.adv |
| AdvVarianceOfSlope | 0.06 | 0.07 | 0.409 | 1.00 | ec.y | behavioral.adv |
| Cnsrct.Intercept_Corr_Adv_Intercept | -0.08 | 0.01 | 0.000 | -0.36 | ec.y | behavioral.adv |
| Cnsrct.Intercept_Corr_Adv_Slope | 0.04 | 0.02 | 0.013 | 0.39 | ec.y | behavioral.adv |
| Cnsrct.Slope_Corr_Adv_Intercept | 0.02 | 0.02 | 0.246 | 0.10 | ec.y | behavioral.adv |
| Cnsrct.Slope_Corr_Adv_Slope | -0.03 | 0.02 | 0.170 | -0.27 | ec.y | behavioral.adv |
| ConstructInterceptOfIntercept | 3.44 | 0.02 | 0.000 | 7.38 | ec.y | peer.adv |
| ConstructVarianceOfIntercept | 0.22 | 0.02 | 0.000 | 1.00 | ec.y | peer.adv |
| ConstructInterceptOfSlope | 0.09 | 0.02 | 0.000 | 0.21 | ec.y | peer.adv |
| ConstructVarianceOfSlope | 0.18 | 0.04 | 0.000 | 1.00 | ec.y | peer.adv |
| AdvInterceptOfIntercept | 1.90 | 0.03 | 0.000 | 3.52 | ec.y | peer.adv |
| AdvVarianceOfIntercept | 0.29 | 0.05 | 0.000 | 1.00 | ec.y | peer.adv |
| AdvInterceptOfSlope | -0.22 | 0.04 | 0.000 | -0.56 | ec.y | peer.adv |
| AdvVarianceOfSlope | 0.15 | 0.09 | 0.078 | 1.00 | ec.y | peer.adv |
| Cnsrct.Intercept_Corr_Adv_Intercept | -0.08 | 0.02 | 0.000 | -0.32 | ec.y | peer.adv |
| Cnsrct.Intercept_Corr_Adv_Slope | 0.05 | 0.02 | 0.009 | 0.28 | ec.y | peer.adv |
| Cnsrct.Slope_Corr_Adv_Intercept | 0.02 | 0.02 | 0.207 | 0.10 | ec.y | peer.adv |
| **Cnsrct.Slope_Corr_Adv_Slope** | **-0.05** | **0.02** | **0.022** | **-0.29** | **ec.y** | **peer.adv** |
| ConstructInterceptOfIntercept | 3.44 | 0.02 | 0.000 | 7.43 | ec.y | parentchild.relationship.adv |
| ConstructVarianceOfIntercept | 0.21 | 0.02 | 0.000 | 1.00 | ec.y | parentchild.relationship.adv |
| ConstructInterceptOfSlope | 0.09 | 0.02 | 0.000 | 0.22 | ec.y | parentchild.relationship.adv |
| ConstructVarianceOfSlope | 0.17 | 0.04 | 0.000 | 1.00 | ec.y | parentchild.relationship.adv |
| AdvInterceptOfIntercept | 1.95 | 0.03 | 0.000 | 4.24 | ec.y | parentchild.relationship.adv |
| AdvVarianceOfIntercept | 0.21 | 0.04 | 0.000 | 1.00 | ec.y | parentchild.relationship.adv |
| AdvInterceptOfSlope | -0.05 | 0.04 | 0.152 | -0.13 | ec.y | parentchild.relationship.adv |
| AdvVarianceOfSlope | 0.16 | 0.09 | 0.073 | 1.00 | ec.y | parentchild.relationship.adv |
| Cnsrct.Intercept_Corr_Adv_Intercept | -0.09 | 0.02 | 0.000 | -0.41 | ec.y | parentchild.relationship.adv |
| Cnsrct.Intercept_Corr_Adv_Slope | 0.02 | 0.02 | 0.240 | 0.13 | ec.y | parentchild.relationship.adv |
| Cnsrct.Slope_Corr_Adv_Intercept | -0.01 | 0.02 | 0.448 | -0.07 | ec.y | parentchild.relationship.adv |
| **Cnsrct.Slope_Corr_Adv_Slope** | **-0.06** | **0.02** | **0.003** | **-0.39** | **ec.y** | **parentchild.relationship.adv** |
| ConstructInterceptOfIntercept | 3.44 | 0.02 | 0.000 | 7.47 | ec.y | parentparent.relationship.adv |
| ConstructVarianceOfIntercept | 0.21 | 0.02 | 0.000 | 1.00 | ec.y | parentparent.relationship.adv |
| ConstructInterceptOfSlope | 0.09 | 0.02 | 0.000 | 0.22 | ec.y | parentparent.relationship.adv |
| ConstructVarianceOfSlope | 0.17 | 0.04 | 0.000 | 1.00 | ec.y | parentparent.relationship.adv |
| AdvInterceptOfIntercept | 1.85 | 0.03 | 0.000 | 3.04 | ec.y | parentparent.relationship.adv |
| AdvVarianceOfIntercept | 0.37 | 0.06 | 0.000 | 1.00 | ec.y | parentparent.relationship.adv |
| AdvInterceptOfSlope | -0.11 | 0.04 | 0.009 | -0.16 | ec.y | parentparent.relationship.adv |
| **AdvVarianceOfSlope** | **0.44** | **0.11** | **0.000** | **1.00** | **ec.y** | **parentparent.relationship.adv** |
| Cnsrct.Intercept_Corr_Adv_Intercept | -0.07 | 0.02 | 0.000 | -0.25 | ec.y | parentparent.relationship.adv |
| Cnsrct.Intercept_Corr_Adv_Slope | 0.02 | 0.02 | 0.478 | 0.05 | ec.y | parentparent.relationship.adv |
| Cnsrct.Slope_Corr_Adv_Intercept | 0.00 | 0.02 | 0.868 | -0.01 | ec.y | parentparent.relationship.adv |
| Cnsrct.Slope_Corr_Adv_Slope | -0.03 | 0.02 | 0.237 | -0.11 | ec.y | parentparent.relationship.adv |
| ConstructInterceptOfIntercept | 3.44 | 0.02 | 0.000 | 7.45 | ec.y | household.relationship.adv |
| ConstructVarianceOfIntercept | 0.21 | 0.02 | 0.000 | 1.00 | ec.y | household.relationship.adv |
| ConstructInterceptOfSlope | 0.09 | 0.02 | 0.000 | 0.22 | ec.y | household.relationship.adv |
| ConstructVarianceOfSlope | 0.17 | 0.04 | 0.000 | 1.00 | ec.y | household.relationship.adv |
| AdvInterceptOfIntercept | 2.06 | 0.03 | 0.000 | 4.51 | ec.y | household.relationship.adv |
| AdvVarianceOfIntercept | 0.21 | 0.04 | 0.000 | 1.00 | ec.y | household.relationship.adv |
| AdvInterceptOfSlope | -0.14 | 0.04 | 0.000 | -0.51 | ec.y | household.relationship.adv |
| AdvVarianceOfSlope | 0.07 | 0.09 | 0.419 | 1.00 | ec.y | household.relationship.adv |
| Cnsrct.Intercept_Corr_Adv_Intercept | -0.07 | 0.02 | 0.000 | -0.32 | ec.y | household.relationship.adv |
| Cnsrct.Intercept_Corr_Adv_Slope | 0.01 | 0.02 | 0.544 | 0.10 | ec.y | household.relationship.adv |
| Cnsrct.Slope_Corr_Adv_Intercept | 0.01 | 0.02 | 0.575 | 0.05 | ec.y | household.relationship.adv |
| Cnsrct.Slope_Corr_Adv_Slope | -0.03 | 0.02 | 0.152 | -0.27 | ec.y | household.relationship.adv |
| ConstructInterceptOfIntercept | 3.44 | 0.02 | 0.000 | 7.44 | ec.y | body.adv |
| ConstructVarianceOfIntercept | 0.21 | 0.02 | 0.000 | 1.00 | ec.y | body.adv |
| ConstructInterceptOfSlope | 0.09 | 0.02 | 0.000 | 0.22 | ec.y | body.adv |
| ConstructVarianceOfSlope | 0.17 | 0.04 | 0.000 | 1.00 | ec.y | body.adv |
| AdvInterceptOfIntercept | 1.60 | 0.03 | 0.000 | 3.67 | ec.y | body.adv |
| AdvVarianceOfIntercept | 0.19 | 0.04 | 0.000 | 1.00 | ec.y | body.adv |
| AdvInterceptOfSlope | 0.00 | 0.03 | 0.998 | NA | ec.y | body.adv |
| AdvVarianceOfSlope | -0.03 | 0.09 | 0.762 | NA | ec.y | body.adv |
| Cnsrct.Intercept_Corr_Adv_Intercept | -0.06 | 0.02 | 0.000 | -0.31 | ec.y | body.adv |
| Cnsrct.Intercept_Corr_Adv_Slope | 0.02 | 0.02 | 0.431 | 0.20 | ec.y | body.adv |
| Cnsrct.Slope_Corr_Adv_Intercept | -0.02 | 0.02 | 0.361 | -0.08 | ec.y | body.adv |
| Cnsrct.Slope_Corr_Adv_Slope | -0.03 | 0.02 | 0.174 | -0.40 | ec.y | body.adv |
| ConstructInterceptOfIntercept | 3.44 | 0.02 | 0.000 | 7.63 | ec.y | romantic.adv |
| ConstructVarianceOfIntercept | 0.20 | 0.02 | 0.000 | 1.00 | ec.y | romantic.adv |
| ConstructInterceptOfSlope | 0.09 | 0.02 | 0.000 | 0.23 | ec.y | romantic.adv |
| ConstructVarianceOfSlope | 0.15 | 0.04 | 0.000 | 1.00 | ec.y | romantic.adv |
| AdvInterceptOfIntercept | 1.38 | 0.02 | 0.000 | 3.37 | ec.y | romantic.adv |
| AdvVarianceOfIntercept | 0.17 | 0.03 | 0.000 | 1.00 | ec.y | romantic.adv |
| AdvInterceptOfSlope | 0.03 | 0.03 | 0.299 | 0.11 | ec.y | romantic.adv |
| AdvVarianceOfSlope | 0.08 | 0.06 | 0.205 | 1.00 | ec.y | romantic.adv |
| Cnsrct.Intercept_Corr_Adv_Intercept | -0.07 | 0.01 | 0.000 | -0.35 | ec.y | romantic.adv |
| Cnsrct.Intercept_Corr_Adv_Slope | 0.01 | 0.02 | 0.453 | 0.09 | ec.y | romantic.adv |
| Cnsrct.Slope_Corr_Adv_Intercept | 0.01 | 0.01 | 0.430 | 0.07 | ec.y | romantic.adv |
| Cnsrct.Slope_Corr_Adv_Slope | -0.03 | 0.02 | 0.086 | -0.27 | ec.y | romantic.adv |
| ConstructInterceptOfIntercept | 3.44 | 0.02 | 0.000 | 7.50 | ec.y | neighborhood.adv |
| ConstructVarianceOfIntercept | 0.21 | 0.02 | 0.000 | 1.00 | ec.y | neighborhood.adv |
| ConstructInterceptOfSlope | 0.09 | 0.02 | 0.000 | 0.23 | ec.y | neighborhood.adv |
| ConstructVarianceOfSlope | 0.16 | 0.04 | 0.000 | 1.00 | ec.y | neighborhood.adv |
| AdvInterceptOfIntercept | 1.52 | 0.03 | 0.000 | 3.51 | ec.y | neighborhood.adv |
| AdvVarianceOfIntercept | 0.19 | 0.04 | 0.000 | 1.00 | ec.y | neighborhood.adv |
| AdvInterceptOfSlope | 0.02 | 0.03 | 0.543 | 0.08 | ec.y | neighborhood.adv |
| AdvVarianceOfSlope | 0.08 | 0.08 | 0.335 | 1.00 | ec.y | neighborhood.adv |
| Cnsrct.Intercept_Corr_Adv_Intercept | -0.03 | 0.01 | 0.018 | -0.17 | ec.y | neighborhood.adv |
| Cnsrct.Intercept_Corr_Adv_Slope | 0.00 | 0.02 | 0.851 | 0.03 | ec.y | neighborhood.adv |
| Cnsrct.Slope_Corr_Adv_Intercept | -0.01 | 0.02 | 0.372 | -0.08 | ec.y | neighborhood.adv |
| Cnsrct.Slope_Corr_Adv_Slope | -0.01 | 0.02 | 0.578 | -0.10 | ec.y | neighborhood.adv |
| ConstructInterceptOfIntercept | 3.44 | 0.02 | 0.000 | 7.42 | ec.y | health.self.adv |
| ConstructVarianceOfIntercept | 0.21 | 0.02 | 0.000 | 1.00 | ec.y | health.self.adv |
| ConstructInterceptOfSlope | 0.09 | 0.02 | 0.000 | 0.22 | ec.y | health.self.adv |
| ConstructVarianceOfSlope | 0.18 | 0.04 | 0.000 | 1.00 | ec.y | health.self.adv |
| AdvInterceptOfIntercept | 1.49 | 0.03 | 0.000 | 3.84 | ec.y | health.self.adv |
| AdvVarianceOfIntercept | 0.15 | 0.04 | 0.000 | 1.00 | ec.y | health.self.adv |
| AdvInterceptOfSlope | -0.09 | 0.03 | 0.006 | -0.66 | ec.y | health.self.adv |
| AdvVarianceOfSlope | 0.02 | 0.08 | 0.795 | 1.00 | ec.y | health.self.adv |
| Cnsrct.Intercept_Corr_Adv_Intercept | -0.02 | 0.01 | 0.268 | -0.09 | ec.y | health.self.adv |
| Cnsrct.Intercept_Corr_Adv_Slope | 0.00 | 0.02 | 0.812 | 0.07 | ec.y | health.self.adv |
| Cnsrct.Slope_Corr_Adv_Intercept | -0.03 | 0.02 | 0.100 | -0.16 | ec.y | health.self.adv |
| Cnsrct.Slope_Corr_Adv_Slope | 0.03 | 0.02 | 0.125 | 0.51 | ec.y | health.self.adv |
| ConstructInterceptOfIntercept | 3.44 | 0.02 | 0.000 | 7.43 | ec.y | health.other.adv |
| ConstructVarianceOfIntercept | 0.21 | 0.02 | 0.000 | 1.00 | ec.y | health.other.adv |
| ConstructInterceptOfSlope | 0.09 | 0.02 | 0.000 | 0.22 | ec.y | health.other.adv |
| ConstructVarianceOfSlope | 0.17 | 0.04 | 0.000 | 1.00 | ec.y | health.other.adv |
| AdvInterceptOfIntercept | 2.08 | 0.04 | 0.000 | 3.47 | ec.y | health.other.adv |
| AdvVarianceOfIntercept | 0.36 | 0.09 | 0.000 | 1.00 | ec.y | health.other.adv |
| AdvInterceptOfSlope | -0.27 | 0.05 | 0.000 | -0.59 | ec.y | health.other.adv |
| AdvVarianceOfSlope | 0.21 | 0.18 | 0.267 | 1.00 | ec.y | health.other.adv |
| Cnsrct.Intercept_Corr_Adv_Intercept | -0.04 | 0.02 | 0.051 | -0.15 | ec.y | health.other.adv |
| Cnsrct.Intercept_Corr_Adv_Slope | 0.07 | 0.03 | 0.023 | 0.31 | ec.y | health.other.adv |
| Cnsrct.Slope_Corr_Adv_Intercept | 0.00 | 0.02 | 0.871 | 0.01 | ec.y | health.other.adv |
| Cnsrct.Slope_Corr_Adv_Slope | -0.06 | 0.03 | 0.063 | -0.31 | ec.y | health.other.adv |
| ConstructInterceptOfIntercept | 3.44 | 0.02 | 0.000 | 7.42 | ec.y | finance.adv |
| ConstructVarianceOfIntercept | 0.22 | 0.02 | 0.000 | 1.00 | ec.y | finance.adv |
| ConstructInterceptOfSlope | 0.09 | 0.02 | 0.000 | 0.22 | ec.y | finance.adv |
| ConstructVarianceOfSlope | 0.18 | 0.04 | 0.000 | 1.00 | ec.y | finance.adv |
| AdvInterceptOfIntercept | 1.53 | 0.03 | 0.000 | 3.74 | ec.y | finance.adv |
| AdvVarianceOfIntercept | 0.17 | 0.04 | 0.000 | 1.00 | ec.y | finance.adv |
| AdvInterceptOfSlope | -0.07 | 0.04 | 0.079 | -0.17 | ec.y | finance.adv |
| AdvVarianceOfSlope | 0.16 | 0.10 | 0.092 | 1.00 | ec.y | finance.adv |
| Cnsrct.Intercept_Corr_Adv_Intercept | -0.06 | 0.02 | 0.000 | -0.33 | ec.y | finance.adv |
| Cnsrct.Intercept_Corr_Adv_Slope | 0.02 | 0.02 | 0.300 | 0.12 | ec.y | finance.adv |
| Cnsrct.Slope_Corr_Adv_Intercept | -0.03 | 0.02 | 0.055 | -0.18 | ec.y | finance.adv |
| Cnsrct.Slope_Corr_Adv_Slope | 0.01 | 0.02 | 0.585 | 0.07 | ec.y | finance.adv |
| ConstructInterceptOfIntercept | 3.44 | 0.02 | 0.000 | 7.44 | ec.y | legal.adv |
| ConstructVarianceOfIntercept | 0.21 | 0.02 | 0.000 | 1.00 | ec.y | legal.adv |
| ConstructInterceptOfSlope | 0.09 | 0.02 | 0.000 | 0.22 | ec.y | legal.adv |
| ConstructVarianceOfSlope | 0.17 | 0.04 | 0.000 | 1.00 | ec.y | legal.adv |
| AdvInterceptOfIntercept | 1.54 | 0.03 | 0.000 | 4.13 | ec.y | legal.adv |
| AdvVarianceOfIntercept | 0.14 | 0.06 | 0.017 | 1.00 | ec.y | legal.adv |
| AdvInterceptOfSlope | -0.19 | 0.04 | 0.000 | -0.67 | ec.y | legal.adv |
| AdvVarianceOfSlope | 0.08 | 0.12 | 0.518 | 1.00 | ec.y | legal.adv |
| Cnsrct.Intercept_Corr_Adv_Intercept | -0.06 | 0.02 | 0.002 | -0.32 | ec.y | legal.adv |
| Cnsrct.Intercept_Corr_Adv_Slope | 0.02 | 0.02 | 0.339 | 0.17 | ec.y | legal.adv |
| Cnsrct.Slope_Corr_Adv_Intercept | 0.01 | 0.02 | 0.577 | 0.07 | ec.y | legal.adv |
| Cnsrct.Slope_Corr_Adv_Slope | -0.02 | 0.03 | 0.351 | -0.21 | ec.y | legal.adv |
| ConstructInterceptOfIntercept | 3.44 | 0.02 | 0.000 | 7.46 | ec.y | violence.adv |
| ConstructVarianceOfIntercept | 0.21 | 0.02 | 0.000 | 1.00 | ec.y | violence.adv |
| ConstructInterceptOfSlope | 0.09 | 0.02 | 0.000 | 0.21 | ec.y | violence.adv |
| ConstructVarianceOfSlope | 0.18 | 0.04 | 0.000 | 1.00 | ec.y | violence.adv |
| AdvInterceptOfIntercept | 1.44 | 0.03 | 0.000 | 3.67 | ec.y | violence.adv |
| AdvVarianceOfIntercept | 0.15 | 0.04 | 0.000 | 1.00 | ec.y | violence.adv |
| AdvInterceptOfSlope | -0.23 | 0.03 | 0.000 | -0.75 | ec.y | violence.adv |
| AdvVarianceOfSlope | 0.09 | 0.08 | 0.250 | 1.00 | ec.y | violence.adv |
| Cnsrct.Intercept_Corr_Adv_Intercept | -0.04 | 0.02 | 0.004 | -0.24 | ec.y | violence.adv |
| Cnsrct.Intercept_Corr_Adv_Slope | 0.05 | 0.02 | 0.014 | 0.33 | ec.y | violence.adv |
| Cnsrct.Slope_Corr_Adv_Intercept | 0.01 | 0.02 | 0.473 | 0.08 | ec.y | violence.adv |
| **Cnsrct.Slope_Corr_Adv_Slope** | **-0.05** | **0.02** | **0.025** | **-0.37** | **ec.y** | **violence.adv** |
| ConstructInterceptOfIntercept | 3.44 | 0.02 | 0.000 | 7.49 | ec.y | discrimination.adv |
| ConstructVarianceOfIntercept | 0.21 | 0.02 | 0.000 | 1.00 | ec.y | discrimination.adv |
| ConstructInterceptOfSlope | 0.09 | 0.02 | 0.000 | 0.22 | ec.y | discrimination.adv |
| ConstructVarianceOfSlope | 0.17 | 0.04 | 0.000 | 1.00 | ec.y | discrimination.adv |
| AdvInterceptOfIntercept | 1.22 | 0.02 | 0.000 | 5.13 | ec.y | discrimination.adv |
| AdvVarianceOfIntercept | 0.06 | 0.02 | 0.003 | 1.00 | ec.y | discrimination.adv |
| AdvInterceptOfSlope | -0.01 | 0.03 | 0.815 | -0.02 | ec.y | discrimination.adv |
| **AdvVarianceOfSlope** | **0.13** | **0.04** | **0.002** | **1.00** | **ec.y** | **discrimination.adv** |
| Cnsrct.Intercept_Corr_Adv_Intercept | -0.03 | 0.01 | 0.002 | -0.29 | ec.y | discrimination.adv |
| Cnsrct.Intercept_Corr_Adv_Slope | 0.03 | 0.01 | 0.047 | 0.17 | ec.y | discrimination.adv |
| Cnsrct.Slope_Corr_Adv_Intercept | 0.00 | 0.01 | 0.740 | 0.04 | ec.y | discrimination.adv |
| Cnsrct.Slope_Corr_Adv_Slope | -0.02 | 0.02 | 0.144 | -0.15 | ec.y | discrimination.adv |
| ConstructInterceptOfIntercept | 3.44 | 0.02 | 0.000 | 7.42 | ec.y | activities.adv |
| ConstructVarianceOfIntercept | 0.21 | 0.02 | 0.000 | 1.00 | ec.y | activities.adv |
| ConstructInterceptOfSlope | 0.09 | 0.02 | 0.000 | 0.22 | ec.y | activities.adv |
| ConstructVarianceOfSlope | 0.17 | 0.04 | 0.000 | 1.00 | ec.y | activities.adv |
| AdvInterceptOfIntercept | 1.35 | 0.02 | 0.000 | 5.95 | ec.y | activities.adv |
| AdvVarianceOfIntercept | 0.05 | 0.02 | 0.010 | 1.00 | ec.y | activities.adv |
| AdvInterceptOfSlope | -0.10 | 0.02 | 0.000 | -2.11 | ec.y | activities.adv |
| AdvVarianceOfSlope | 0.00 | 0.04 | 0.957 | 1.00 | ec.y | activities.adv |
| Cnsrct.Intercept_Corr_Adv_Intercept | -0.03 | 0.01 | 0.002 | -0.28 | ec.y | activities.adv |
| Cnsrct.Intercept_Corr_Adv_Slope | 0.02 | 0.01 | 0.068 | 1.09 | ec.y | activities.adv |
| Cnsrct.Slope_Corr_Adv_Intercept | -0.01 | 0.01 | 0.421 | -0.09 | ec.y | activities.adv |
| Cnsrct.Slope_Corr_Adv_Slope | -0.02 | 0.01 | 0.260 | -0.78 | ec.y | activities.adv |
| ConstructInterceptOfIntercept | 3.25 | 0.02 | 0.000 | 6.26 | ec.p | academic.adv |
| ConstructVarianceOfIntercept | 0.27 | 0.02 | 0.000 | 1.00 | ec.p | academic.adv |
| ConstructInterceptOfSlope | 0.11 | 0.02 | 0.000 | 0.30 | ec.p | academic.adv |
| ConstructVarianceOfSlope | 0.12 | 0.03 | 0.000 | 1.00 | ec.p | academic.adv |
| AdvInterceptOfIntercept | 1.79 | 0.03 | 0.000 | 3.38 | ec.p | academic.adv |
| AdvVarianceOfIntercept | 0.28 | 0.05 | 0.000 | 1.00 | ec.p | academic.adv |
| AdvInterceptOfSlope | 0.05 | 0.04 | 0.164 | 0.12 | ec.p | academic.adv |
| AdvVarianceOfSlope | 0.17 | 0.09 | 0.070 | 1.00 | ec.p | academic.adv |
| Cnsrct.Intercept_Corr_Adv_Intercept | -0.14 | 0.02 | 0.000 | -0.50 | ec.p | academic.adv |
| Cnsrct.Intercept_Corr_Adv_Slope | 0.02 | 0.02 | 0.290 | 0.10 | ec.p | academic.adv |
| Cnsrct.Slope_Corr_Adv_Intercept | 0.01 | 0.01 | 0.661 | 0.03 | ec.p | academic.adv |
| **Cnsrct.Slope_Corr_Adv_Slope** | **-0.07** | **0.02** | **0.000** | **-0.50** | **ec.p** | **academic.adv** |
| ConstructInterceptOfIntercept | 3.25 | 0.02 | 0.000 | 6.24 | ec.p | behavioral.adv |
| ConstructVarianceOfIntercept | 0.27 | 0.02 | 0.000 | 1.00 | ec.p | behavioral.adv |
| ConstructInterceptOfSlope | 0.11 | 0.02 | 0.000 | 0.30 | ec.p | behavioral.adv |
| ConstructVarianceOfSlope | 0.13 | 0.03 | 0.000 | 1.00 | ec.p | behavioral.adv |
| AdvInterceptOfIntercept | 1.42 | 0.03 | 0.000 | 3.14 | ec.p | behavioral.adv |
| AdvVarianceOfIntercept | 0.20 | 0.03 | 0.000 | 1.00 | ec.p | behavioral.adv |
| AdvInterceptOfSlope | -0.25 | 0.03 | 0.000 | -1.01 | ec.p | behavioral.adv |
| AdvVarianceOfSlope | 0.06 | 0.07 | 0.390 | 1.00 | ec.p | behavioral.adv |
| Cnsrct.Intercept_Corr_Adv_Intercept | -0.07 | 0.01 | 0.000 | -0.31 | ec.p | behavioral.adv |
| Cnsrct.Intercept_Corr_Adv_Slope | 0.01 | 0.02 | 0.720 | 0.05 | ec.p | behavioral.adv |
| Cnsrct.Slope_Corr_Adv_Intercept | 0.00 | 0.01 | 0.995 | 0.00 | ec.p | behavioral.adv |
| Cnsrct.Slope_Corr_Adv_Slope | -0.01 | 0.01 | 0.318 | -0.17 | ec.p | behavioral.adv |
| ConstructInterceptOfIntercept | 3.25 | 0.02 | 0.000 | 6.18 | ec.p | peer.adv |
| ConstructVarianceOfIntercept | 0.28 | 0.02 | 0.000 | 1.00 | ec.p | peer.adv |
| ConstructInterceptOfSlope | 0.11 | 0.02 | 0.000 | 0.28 | ec.p | peer.adv |
| ConstructVarianceOfSlope | 0.14 | 0.03 | 0.000 | 1.00 | ec.p | peer.adv |
| AdvInterceptOfIntercept | 1.91 | 0.03 | 0.000 | 3.50 | ec.p | peer.adv |
| AdvVarianceOfIntercept | 0.30 | 0.04 | 0.000 | 1.00 | ec.p | peer.adv |
| AdvInterceptOfSlope | -0.22 | 0.04 | 0.000 | -0.54 | ec.p | peer.adv |
| AdvVarianceOfSlope | 0.16 | 0.08 | 0.055 | 1.00 | ec.p | peer.adv |
| Cnsrct.Intercept_Corr_Adv_Intercept | -0.10 | 0.02 | 0.000 | -0.35 | ec.p | peer.adv |
| Cnsrct.Intercept_Corr_Adv_Slope | 0.06 | 0.02 | 0.003 | 0.27 | ec.p | peer.adv |
| Cnsrct.Slope_Corr_Adv_Intercept | 0.03 | 0.01 | 0.019 | 0.16 | ec.p | peer.adv |
| **Cnsrct.Slope_Corr_Adv_Slope** | **-0.07** | **0.02** | **0.000** | **-0.43** | **ec.p** | **peer.adv** |
| ConstructInterceptOfIntercept | 3.25 | 0.02 | 0.000 | 6.18 | ec.p | parentchild.relationship.adv |
| ConstructVarianceOfIntercept | 0.28 | 0.02 | 0.000 | 1.00 | ec.p | parentchild.relationship.adv |
| ConstructInterceptOfSlope | 0.11 | 0.02 | 0.000 | 0.29 | ec.p | parentchild.relationship.adv |
| ConstructVarianceOfSlope | 0.14 | 0.03 | 0.000 | 1.00 | ec.p | parentchild.relationship.adv |
| AdvInterceptOfIntercept | 1.95 | 0.03 | 0.000 | 4.04 | ec.p | parentchild.relationship.adv |
| AdvVarianceOfIntercept | 0.23 | 0.04 | 0.000 | 1.00 | ec.p | parentchild.relationship.adv |
| AdvInterceptOfSlope | -0.05 | 0.04 | 0.162 | -0.11 | ec.p | parentchild.relationship.adv |
| **AdvVarianceOfSlope** | **0.22** | **0.09** | **0.015** | **1.00** | **ec.p** | **parentchild.relationship.adv** |
| Cnsrct.Intercept_Corr_Adv_Intercept | -0.07 | 0.02 | 0.000 | -0.28 | ec.p | parentchild.relationship.adv |
| Cnsrct.Intercept_Corr_Adv_Slope | 0.00 | 0.02 | 0.837 | -0.02 | ec.p | parentchild.relationship.adv |
| Cnsrct.Slope_Corr_Adv_Intercept | 0.00 | 0.01 | 0.909 | 0.01 | ec.p | parentchild.relationship.adv |
| Cnsrct.Slope_Corr_Adv_Slope | -0.02 | 0.02 | 0.181 | -0.13 | ec.p | parentchild.relationship.adv |
| ConstructInterceptOfIntercept | 3.25 | 0.02 | 0.000 | 6.13 | ec.p | parentparent.relationship.adv |
| ConstructVarianceOfIntercept | 0.28 | 0.02 | 0.000 | 1.00 | ec.p | parentparent.relationship.adv |
| ConstructInterceptOfSlope | 0.11 | 0.02 | 0.000 | 0.28 | ec.p | parentparent.relationship.adv |
| ConstructVarianceOfSlope | 0.15 | 0.03 | 0.000 | 1.00 | ec.p | parentparent.relationship.adv |
| AdvInterceptOfIntercept | 1.85 | 0.03 | 0.000 | 3.06 | ec.p | parentparent.relationship.adv |
| AdvVarianceOfIntercept | 0.36 | 0.06 | 0.000 | 1.00 | ec.p | parentparent.relationship.adv |
| AdvInterceptOfSlope | -0.11 | 0.04 | 0.008 | -0.17 | ec.p | parentparent.relationship.adv |
| **AdvVarianceOfSlope** | **0.44** | **0.11** | **0.000** | **1.00** | **ec.p** | **parentparent.relationship.adv** |
| Cnsrct.Intercept_Corr_Adv_Intercept | -0.04 | 0.02 | 0.025 | -0.12 | ec.p | parentparent.relationship.adv |
| Cnsrct.Intercept_Corr_Adv_Slope | 0.04 | 0.02 | 0.102 | 0.11 | ec.p | parentparent.relationship.adv |
| **Cnsrct.Slope_Corr_Adv_Intercept** | **-0.03** | **0.01** | **0.042** | **-0.13** | **ec.p** | **parentparent.relationship.adv** |
| Cnsrct.Slope_Corr_Adv_Slope | -0.02 | 0.02 | 0.295 | -0.08 | ec.p | parentparent.relationship.adv |
| ConstructInterceptOfIntercept | 3.25 | 0.02 | 0.000 | 6.14 | ec.p | household.relationship.adv |
| ConstructVarianceOfIntercept | 0.28 | 0.02 | 0.000 | 1.00 | ec.p | household.relationship.adv |
| ConstructInterceptOfSlope | 0.11 | 0.02 | 0.000 | 0.28 | ec.p | household.relationship.adv |
| ConstructVarianceOfSlope | 0.15 | 0.03 | 0.000 | 1.00 | ec.p | household.relationship.adv |
| AdvInterceptOfIntercept | 2.06 | 0.03 | 0.000 | 4.42 | ec.p | household.relationship.adv |
| AdvVarianceOfIntercept | 0.22 | 0.04 | 0.000 | 1.00 | ec.p | household.relationship.adv |
| AdvInterceptOfSlope | -0.14 | 0.04 | 0.000 | -0.45 | ec.p | household.relationship.adv |
| AdvVarianceOfSlope | 0.09 | 0.09 | 0.330 | 1.00 | ec.p | household.relationship.adv |
| Cnsrct.Intercept_Corr_Adv_Intercept | -0.03 | 0.02 | 0.043 | -0.13 | ec.p | household.relationship.adv |
| Cnsrct.Intercept_Corr_Adv_Slope | -0.04 | 0.02 | 0.088 | -0.23 | ec.p | household.relationship.adv |
| Cnsrct.Slope_Corr_Adv_Intercept | 0.00 | 0.01 | 0.825 | 0.02 | ec.p | household.relationship.adv |
| Cnsrct.Slope_Corr_Adv_Slope | -0.01 | 0.02 | 0.676 | -0.06 | ec.p | household.relationship.adv |
| ConstructInterceptOfIntercept | 3.25 | 0.02 | 0.000 | 6.14 | ec.p | body.adv |
| ConstructVarianceOfIntercept | 0.28 | 0.02 | 0.000 | 1.00 | ec.p | body.adv |
| ConstructInterceptOfSlope | 0.11 | 0.02 | 0.000 | 0.28 | ec.p | body.adv |
| ConstructVarianceOfSlope | 0.15 | 0.03 | 0.000 | 1.00 | ec.p | body.adv |
| AdvInterceptOfIntercept | 1.59 | 0.03 | 0.000 | 3.63 | ec.p | body.adv |
| AdvVarianceOfIntercept | 0.19 | 0.04 | 0.000 | 1.00 | ec.p | body.adv |
| AdvInterceptOfSlope | 0.00 | 0.03 | 0.985 | NA | ec.p | body.adv |
| AdvVarianceOfSlope | -0.03 | 0.09 | 0.751 | NA | ec.p | body.adv |
| Cnsrct.Intercept_Corr_Adv_Intercept | 0.01 | 0.02 | 0.409 | 0.05 | ec.p | body.adv |
| Cnsrct.Intercept_Corr_Adv_Slope | -0.02 | 0.02 | 0.375 | -0.19 | ec.p | body.adv |
| Cnsrct.Slope_Corr_Adv_Intercept | 0.00 | 0.01 | 0.816 | -0.02 | ec.p | body.adv |
| **Cnsrct.Slope_Corr_Adv_Slope** | **-0.03** | **0.02** | **0.046** | **-0.50** | **ec.p** | **body.adv** |
| ConstructInterceptOfIntercept | 3.25 | 0.02 | 0.000 | 6.15 | ec.p | romantic.adv |
| ConstructVarianceOfIntercept | 0.28 | 0.02 | 0.000 | 1.00 | ec.p | romantic.adv |
| ConstructInterceptOfSlope | 0.11 | 0.02 | 0.000 | 0.28 | ec.p | romantic.adv |
| ConstructVarianceOfSlope | 0.15 | 0.03 | 0.000 | 1.00 | ec.p | romantic.adv |
| AdvInterceptOfIntercept | 1.38 | 0.02 | 0.000 | 3.41 | ec.p | romantic.adv |
| AdvVarianceOfIntercept | 0.16 | 0.03 | 0.000 | 1.00 | ec.p | romantic.adv |
| AdvInterceptOfSlope | 0.03 | 0.03 | 0.288 | 0.13 | ec.p | romantic.adv |
| AdvVarianceOfSlope | 0.06 | 0.06 | 0.352 | 1.00 | ec.p | romantic.adv |
| Cnsrct.Intercept_Corr_Adv_Intercept | -0.02 | 0.01 | 0.109 | -0.10 | ec.p | romantic.adv |
| Cnsrct.Intercept_Corr_Adv_Slope | 0.00 | 0.02 | 0.921 | 0.01 | ec.p | romantic.adv |
| Cnsrct.Slope_Corr_Adv_Intercept | 0.00 | 0.01 | 0.719 | -0.03 | ec.p | romantic.adv |
| Cnsrct.Slope_Corr_Adv_Slope | 0.01 | 0.01 | 0.623 | 0.07 | ec.p | romantic.adv |
| ConstructInterceptOfIntercept | 3.25 | 0.02 | 0.000 | 6.15 | ec.p | neighborhood.adv |
| ConstructVarianceOfIntercept | 0.28 | 0.02 | 0.000 | 1.00 | ec.p | neighborhood.adv |
| ConstructInterceptOfSlope | 0.11 | 0.02 | 0.000 | 0.28 | ec.p | neighborhood.adv |
| ConstructVarianceOfSlope | 0.15 | 0.03 | 0.000 | 1.00 | ec.p | neighborhood.adv |
| AdvInterceptOfIntercept | 1.52 | 0.03 | 0.000 | 3.52 | ec.p | neighborhood.adv |
| AdvVarianceOfIntercept | 0.19 | 0.04 | 0.000 | 1.00 | ec.p | neighborhood.adv |
| AdvInterceptOfSlope | 0.02 | 0.03 | 0.533 | 0.08 | ec.p | neighborhood.adv |
| AdvVarianceOfSlope | 0.07 | 0.08 | 0.360 | 1.00 | ec.p | neighborhood.adv |
| Cnsrct.Intercept_Corr_Adv_Intercept | -0.01 | 0.01 | 0.397 | -0.06 | ec.p | neighborhood.adv |
| Cnsrct.Intercept_Corr_Adv_Slope | -0.02 | 0.02 | 0.253 | -0.15 | ec.p | neighborhood.adv |
| Cnsrct.Slope_Corr_Adv_Intercept | -0.01 | 0.01 | 0.386 | -0.07 | ec.p | neighborhood.adv |
| Cnsrct.Slope_Corr_Adv_Slope | 0.01 | 0.02 | 0.566 | 0.09 | ec.p | neighborhood.adv |
| ConstructInterceptOfIntercept | 3.25 | 0.02 | 0.000 | 6.14 | ec.p | health.self.adv |
| ConstructVarianceOfIntercept | 0.28 | 0.02 | 0.000 | 1.00 | ec.p | health.self.adv |
| ConstructInterceptOfSlope | 0.11 | 0.02 | 0.000 | 0.28 | ec.p | health.self.adv |
| ConstructVarianceOfSlope | 0.15 | 0.03 | 0.000 | 1.00 | ec.p | health.self.adv |
| AdvInterceptOfIntercept | 1.49 | 0.03 | 0.000 | 3.80 | ec.p | health.self.adv |
| AdvVarianceOfIntercept | 0.15 | 0.04 | 0.000 | 1.00 | ec.p | health.self.adv |
| AdvInterceptOfSlope | -0.10 | 0.03 | 0.006 | -0.61 | ec.p | health.self.adv |
| AdvVarianceOfSlope | 0.02 | 0.08 | 0.758 | 1.00 | ec.p | health.self.adv |
| Cnsrct.Intercept_Corr_Adv_Intercept | 0.00 | 0.01 | 0.749 | -0.02 | ec.p | health.self.adv |
| Cnsrct.Intercept_Corr_Adv_Slope | 0.01 | 0.02 | 0.769 | 0.07 | ec.p | health.self.adv |
| Cnsrct.Slope_Corr_Adv_Intercept | 0.00 | 0.01 | 0.719 | -0.03 | ec.p | health.self.adv |
| Cnsrct.Slope_Corr_Adv_Slope | -0.01 | 0.02 | 0.632 | -0.13 | ec.p | health.self.adv |
| ConstructInterceptOfIntercept | 3.25 | 0.02 | 0.000 | 6.14 | ec.p | health.other.adv |
| ConstructVarianceOfIntercept | 0.28 | 0.02 | 0.000 | 1.00 | ec.p | health.other.adv |
| ConstructInterceptOfSlope | 0.11 | 0.02 | 0.000 | 0.28 | ec.p | health.other.adv |
| ConstructVarianceOfSlope | 0.15 | 0.03 | 0.000 | 1.00 | ec.p | health.other.adv |
| AdvInterceptOfIntercept | 2.08 | 0.04 | 0.000 | 3.44 | ec.p | health.other.adv |
| AdvVarianceOfIntercept | 0.37 | 0.09 | 0.000 | 1.00 | ec.p | health.other.adv |
| AdvInterceptOfSlope | -0.27 | 0.05 | 0.000 | -0.56 | ec.p | health.other.adv |
| AdvVarianceOfSlope | 0.22 | 0.18 | 0.224 | 1.00 | ec.p | health.other.adv |
| Cnsrct.Intercept_Corr_Adv_Intercept | 0.01 | 0.02 | 0.661 | 0.03 | ec.p | health.other.adv |
| Cnsrct.Intercept_Corr_Adv_Slope | 0.01 | 0.03 | 0.803 | 0.03 | ec.p | health.other.adv |
| **Cnsrct.Slope_Corr_Adv_Intercept** | **-0.05** | **0.02** | **0.008** | **-0.21** | **ec.p** | **health.other.adv** |
| Cnsrct.Slope_Corr_Adv_Slope | 0.04 | 0.03 | 0.157 | 0.19 | ec.p | health.other.adv |
| ConstructInterceptOfIntercept | 3.25 | 0.02 | 0.000 | 6.14 | ec.p | finance.adv |
| ConstructVarianceOfIntercept | 0.28 | 0.02 | 0.000 | 1.00 | ec.p | finance.adv |
| ConstructInterceptOfSlope | 0.11 | 0.02 | 0.000 | 0.28 | ec.p | finance.adv |
| ConstructVarianceOfSlope | 0.15 | 0.03 | 0.000 | 1.00 | ec.p | finance.adv |
| AdvInterceptOfIntercept | 1.53 | 0.03 | 0.000 | 3.66 | ec.p | finance.adv |
| AdvVarianceOfIntercept | 0.17 | 0.04 | 0.000 | 1.00 | ec.p | finance.adv |
| AdvInterceptOfSlope | -0.07 | 0.04 | 0.085 | -0.16 | ec.p | finance.adv |
| AdvVarianceOfSlope | 0.19 | 0.10 | 0.053 | 1.00 | ec.p | finance.adv |
| Cnsrct.Intercept_Corr_Adv_Intercept | -0.03 | 0.02 | 0.049 | -0.14 | ec.p | finance.adv |
| Cnsrct.Intercept_Corr_Adv_Slope | -0.02 | 0.02 | 0.452 | -0.07 | ec.p | finance.adv |
| Cnsrct.Slope_Corr_Adv_Intercept | 0.00 | 0.01 | 0.752 | -0.03 | ec.p | finance.adv |
| Cnsrct.Slope_Corr_Adv_Slope | 0.02 | 0.02 | 0.264 | 0.12 | ec.p | finance.adv |
| ConstructInterceptOfIntercept | 3.25 | 0.02 | 0.000 | 6.11 | ec.p | legal.adv |
| ConstructVarianceOfIntercept | 0.28 | 0.02 | 0.000 | 1.00 | ec.p | legal.adv |
| ConstructInterceptOfSlope | 0.11 | 0.02 | 0.000 | 0.27 | ec.p | legal.adv |
| ConstructVarianceOfSlope | 0.15 | 0.03 | 0.000 | 1.00 | ec.p | legal.adv |
| AdvInterceptOfIntercept | 1.54 | 0.03 | 0.000 | 4.03 | ec.p | legal.adv |
| AdvVarianceOfIntercept | 0.15 | 0.06 | 0.012 | 1.00 | ec.p | legal.adv |
| AdvInterceptOfSlope | -0.19 | 0.04 | 0.000 | -0.63 | ec.p | legal.adv |
| AdvVarianceOfSlope | 0.09 | 0.12 | 0.466 | 1.00 | ec.p | legal.adv |
| Cnsrct.Intercept_Corr_Adv_Intercept | -0.06 | 0.02 | 0.001 | -0.28 | ec.p | legal.adv |
| Cnsrct.Intercept_Corr_Adv_Slope | 0.05 | 0.02 | 0.037 | 0.31 | ec.p | legal.adv |
| Cnsrct.Slope_Corr_Adv_Intercept | 0.01 | 0.02 | 0.551 | 0.06 | ec.p | legal.adv |
| Cnsrct.Slope_Corr_Adv_Slope | -0.01 | 0.02 | 0.571 | -0.10 | ec.p | legal.adv |
| ConstructInterceptOfIntercept | 3.25 | 0.02 | 0.000 | 6.13 | ec.p | violence.adv |
| ConstructVarianceOfIntercept | 0.28 | 0.02 | 0.000 | 1.00 | ec.p | violence.adv |
| ConstructInterceptOfSlope | 0.11 | 0.02 | 0.000 | 0.28 | ec.p | violence.adv |
| ConstructVarianceOfSlope | 0.15 | 0.03 | 0.000 | 1.00 | ec.p | violence.adv |
| AdvInterceptOfIntercept | 1.44 | 0.03 | 0.000 | 3.62 | ec.p | violence.adv |
| AdvVarianceOfIntercept | 0.16 | 0.04 | 0.000 | 1.00 | ec.p | violence.adv |
| AdvInterceptOfSlope | -0.23 | 0.03 | 0.000 | -0.75 | ec.p | violence.adv |
| AdvVarianceOfSlope | 0.09 | 0.08 | 0.242 | 1.00 | ec.p | violence.adv |
| Cnsrct.Intercept_Corr_Adv_Intercept | -0.06 | 0.02 | 0.000 | -0.30 | ec.p | violence.adv |
| Cnsrct.Intercept_Corr_Adv_Slope | 0.04 | 0.02 | 0.056 | 0.23 | ec.p | violence.adv |
| Cnsrct.Slope_Corr_Adv_Intercept | 0.02 | 0.01 | 0.259 | 0.10 | ec.p | violence.adv |
| **Cnsrct.Slope_Corr_Adv_Slope** | **-0.04** | **0.02** | **0.026** | **-0.31** | **ec.p** | **violence.adv** |
| ConstructInterceptOfIntercept | 3.25 | 0.02 | 0.000 | 6.13 | ec.p | discrimination.adv |
| ConstructVarianceOfIntercept | 0.28 | 0.02 | 0.000 | 1.00 | ec.p | discrimination.adv |
| ConstructInterceptOfSlope | 0.11 | 0.02 | 0.000 | 0.28 | ec.p | discrimination.adv |
| ConstructVarianceOfSlope | 0.15 | 0.03 | 0.000 | 1.00 | ec.p | discrimination.adv |
| AdvInterceptOfIntercept | 1.22 | 0.02 | 0.000 | 5.03 | ec.p | discrimination.adv |
| AdvVarianceOfIntercept | 0.06 | 0.02 | 0.002 | 1.00 | ec.p | discrimination.adv |
| AdvInterceptOfSlope | -0.01 | 0.03 | 0.829 | -0.02 | ec.p | discrimination.adv |
| **AdvVarianceOfSlope** | **0.13** | **0.04** | **0.002** | **1.00** | **ec.p** | **discrimination.adv** |
| Cnsrct.Intercept_Corr_Adv_Intercept | 0.01 | 0.01 | 0.466 | 0.06 | ec.p | discrimination.adv |
| Cnsrct.Intercept_Corr_Adv_Slope | -0.01 | 0.01 | 0.647 | -0.03 | ec.p | discrimination.adv |
| Cnsrct.Slope_Corr_Adv_Intercept | 0.00 | 0.01 | 0.767 | -0.03 | ec.p | discrimination.adv |
| Cnsrct.Slope_Corr_Adv_Slope | -0.01 | 0.01 | 0.338 | -0.08 | ec.p | discrimination.adv |
| ConstructInterceptOfIntercept | 3.25 | 0.02 | 0.000 | 6.14 | ec.p | activities.adv |
| ConstructVarianceOfIntercept | 0.28 | 0.02 | 0.000 | 1.00 | ec.p | activities.adv |
| ConstructInterceptOfSlope | 0.11 | 0.02 | 0.000 | 0.28 | ec.p | activities.adv |
| ConstructVarianceOfSlope | 0.15 | 0.03 | 0.000 | 1.00 | ec.p | activities.adv |
| AdvInterceptOfIntercept | 1.35 | 0.02 | 0.000 | 5.86 | ec.p | activities.adv |
| AdvVarianceOfIntercept | 0.05 | 0.02 | 0.009 | 1.00 | ec.p | activities.adv |
| AdvInterceptOfSlope | -0.10 | 0.02 | 0.000 | -1.21 | ec.p | activities.adv |
| AdvVarianceOfSlope | 0.01 | 0.04 | 0.869 | 1.00 | ec.p | activities.adv |
| Cnsrct.Intercept_Corr_Adv_Intercept | 0.00 | 0.01 | 0.889 | 0.01 | ec.p | activities.adv |
| Cnsrct.Intercept_Corr_Adv_Slope | 0.01 | 0.01 | 0.612 | 0.15 | ec.p | activities.adv |
| Cnsrct.Slope_Corr_Adv_Intercept | -0.01 | 0.01 | 0.533 | -0.06 | ec.p | activities.adv |
| Cnsrct.Slope_Corr_Adv_Slope | 0.00 | 0.01 | 0.779 | -0.10 | ec.p | activities.adv |
| ConstructInterceptOfIntercept | 3.38 | 0.02 | 0.000 | 8.66 | ne.y | academic.adv |
| ConstructVarianceOfIntercept | 0.15 | 0.02 | 0.000 | 1.00 | ne.y | academic.adv |
| ConstructInterceptOfSlope | 0.28 | 0.03 | 0.000 | 0.72 | ne.y | academic.adv |
| ConstructVarianceOfSlope | 0.15 | 0.04 | 0.000 | 1.00 | ne.y | academic.adv |
| AdvInterceptOfIntercept | 1.79 | 0.03 | 0.000 | 3.50 | ne.y | academic.adv |
| AdvVarianceOfIntercept | 0.26 | 0.05 | 0.000 | 1.00 | ne.y | academic.adv |
| AdvInterceptOfSlope | 0.05 | 0.04 | 0.133 | 0.15 | ne.y | academic.adv |
| AdvVarianceOfSlope | 0.13 | 0.10 | 0.185 | 1.00 | ne.y | academic.adv |
| Cnsrct.Intercept_Corr_Adv_Intercept | -0.05 | 0.01 | 0.000 | -0.25 | ne.y | academic.adv |
| Cnsrct.Intercept_Corr_Adv_Slope | 0.02 | 0.02 | 0.324 | 0.13 | ne.y | academic.adv |
| Cnsrct.Slope_Corr_Adv_Intercept | 0.03 | 0.02 | 0.048 | 0.17 | ne.y | academic.adv |
| **Cnsrct.Slope_Corr_Adv_Slope** | **-0.08** | **0.02** | **0.000** | **-0.57** | **ne.y** | **academic.adv** |
| ConstructInterceptOfIntercept | 3.38 | 0.02 | 0.000 | 8.61 | ne.y | behavioral.adv |
| ConstructVarianceOfIntercept | 0.15 | 0.02 | 0.000 | 1.00 | ne.y | behavioral.adv |
| ConstructInterceptOfSlope | 0.28 | 0.03 | 0.000 | 0.72 | ne.y | behavioral.adv |
| ConstructVarianceOfSlope | 0.16 | 0.04 | 0.000 | 1.00 | ne.y | behavioral.adv |
| AdvInterceptOfIntercept | 1.42 | 0.03 | 0.000 | 3.19 | ne.y | behavioral.adv |
| AdvVarianceOfIntercept | 0.20 | 0.04 | 0.000 | 1.00 | ne.y | behavioral.adv |
| AdvInterceptOfSlope | -0.25 | 0.03 | 0.000 | -1.23 | ne.y | behavioral.adv |
| AdvVarianceOfSlope | 0.04 | 0.07 | 0.576 | 1.00 | ne.y | behavioral.adv |
| Cnsrct.Intercept_Corr_Adv_Intercept | -0.04 | 0.01 | 0.005 | -0.21 | ne.y | behavioral.adv |
| Cnsrct.Intercept_Corr_Adv_Slope | 0.03 | 0.02 | 0.050 | 0.40 | ne.y | behavioral.adv |
| Cnsrct.Slope_Corr_Adv_Intercept | 0.02 | 0.02 | 0.352 | 0.09 | ne.y | behavioral.adv |
| Cnsrct.Slope_Corr_Adv_Slope | -0.02 | 0.02 | 0.230 | -0.30 | ne.y | behavioral.adv |
| ConstructInterceptOfIntercept | 3.38 | 0.02 | 0.000 | 8.53 | ne.y | peer.adv |
| ConstructVarianceOfIntercept | 0.16 | 0.02 | 0.000 | 1.00 | ne.y | peer.adv |
| ConstructInterceptOfSlope | 0.28 | 0.03 | 0.000 | 0.71 | ne.y | peer.adv |
| ConstructVarianceOfSlope | 0.16 | 0.04 | 0.000 | 1.00 | ne.y | peer.adv |
| AdvInterceptOfIntercept | 1.90 | 0.03 | 0.000 | 3.55 | ne.y | peer.adv |
| AdvVarianceOfIntercept | 0.29 | 0.04 | 0.000 | 1.00 | ne.y | peer.adv |
| AdvInterceptOfSlope | -0.22 | 0.04 | 0.000 | -0.59 | ne.y | peer.adv |
| AdvVarianceOfSlope | 0.13 | 0.09 | 0.116 | 1.00 | ne.y | peer.adv |
| Cnsrct.Intercept_Corr_Adv_Intercept | -0.08 | 0.01 | 0.000 | -0.39 | ne.y | peer.adv |
| Cnsrct.Intercept_Corr_Adv_Slope | 0.06 | 0.02 | 0.002 | 0.40 | ne.y | peer.adv |
| Cnsrct.Slope_Corr_Adv_Intercept | 0.02 | 0.02 | 0.361 | 0.08 | ne.y | peer.adv |
| **Cnsrct.Slope_Corr_Adv_Slope** | **-0.07** | **0.02** | **0.001** | **-0.51** | **ne.y** | **peer.adv** |
| ConstructInterceptOfIntercept | 3.38 | 0.02 | 0.000 | 8.58 | ne.y | parentchild.relationship.adv |
| ConstructVarianceOfIntercept | 0.16 | 0.02 | 0.000 | 1.00 | ne.y | parentchild.relationship.adv |
| ConstructInterceptOfSlope | 0.28 | 0.03 | 0.000 | 0.71 | ne.y | parentchild.relationship.adv |
| ConstructVarianceOfSlope | 0.15 | 0.04 | 0.000 | 1.00 | ne.y | parentchild.relationship.adv |
| AdvInterceptOfIntercept | 1.95 | 0.03 | 0.000 | 4.13 | ne.y | parentchild.relationship.adv |
| AdvVarianceOfIntercept | 0.22 | 0.04 | 0.000 | 1.00 | ne.y | parentchild.relationship.adv |
| AdvInterceptOfSlope | -0.05 | 0.04 | 0.180 | -0.11 | ne.y | parentchild.relationship.adv |
| **AdvVarianceOfSlope** | **0.20** | **0.09** | **0.027** | **1.00** | **ne.y** | **parentchild.relationship.adv** |
| Cnsrct.Intercept_Corr_Adv_Intercept | -0.05 | 0.01 | 0.000 | -0.28 | ne.y | parentchild.relationship.adv |
| Cnsrct.Intercept_Corr_Adv_Slope | 0.02 | 0.02 | 0.284 | 0.11 | ne.y | parentchild.relationship.adv |
| Cnsrct.Slope_Corr_Adv_Intercept | -0.01 | 0.02 | 0.460 | -0.07 | ne.y | parentchild.relationship.adv |
| **Cnsrct.Slope_Corr_Adv_Slope** | **-0.07** | **0.02** | **0.002** | **-0.39** | **ne.y** | **parentchild.relationship.adv** |
| ConstructInterceptOfIntercept | 3.38 | 0.02 | 0.000 | 8.62 | ne.y | parentparent.relationship.adv |
| ConstructVarianceOfIntercept | 0.15 | 0.02 | 0.000 | 1.00 | ne.y | parentparent.relationship.adv |
| ConstructInterceptOfSlope | 0.28 | 0.03 | 0.000 | 0.72 | ne.y | parentparent.relationship.adv |
| ConstructVarianceOfSlope | 0.15 | 0.04 | 0.000 | 1.00 | ne.y | parentparent.relationship.adv |
| AdvInterceptOfIntercept | 1.84 | 0.03 | 0.000 | 3.04 | ne.y | parentparent.relationship.adv |
| AdvVarianceOfIntercept | 0.37 | 0.06 | 0.000 | 1.00 | ne.y | parentparent.relationship.adv |
| AdvInterceptOfSlope | -0.11 | 0.04 | 0.010 | -0.16 | ne.y | parentparent.relationship.adv |
| **AdvVarianceOfSlope** | **0.44** | **0.11** | **0.000** | **1.00** | **ne.y** | **parentparent.relationship.adv** |
| Cnsrct.Intercept_Corr_Adv_Intercept | -0.04 | 0.02 | 0.007 | -0.18 | ne.y | parentparent.relationship.adv |
| Cnsrct.Intercept_Corr_Adv_Slope | 0.02 | 0.02 | 0.470 | 0.06 | ne.y | parentparent.relationship.adv |
| Cnsrct.Slope_Corr_Adv_Intercept | 0.01 | 0.02 | 0.647 | 0.04 | ne.y | parentparent.relationship.adv |
| Cnsrct.Slope_Corr_Adv_Slope | -0.03 | 0.03 | 0.199 | -0.13 | ne.y | parentparent.relationship.adv |
| ConstructInterceptOfIntercept | 3.38 | 0.02 | 0.000 | 8.61 | ne.y | household.relationship.adv |
| ConstructVarianceOfIntercept | 0.15 | 0.02 | 0.000 | 1.00 | ne.y | household.relationship.adv |
| ConstructInterceptOfSlope | 0.28 | 0.03 | 0.000 | 0.72 | ne.y | household.relationship.adv |
| ConstructVarianceOfSlope | 0.16 | 0.04 | 0.000 | 1.00 | ne.y | household.relationship.adv |
| AdvInterceptOfIntercept | 2.06 | 0.03 | 0.000 | 4.47 | ne.y | household.relationship.adv |
| AdvVarianceOfIntercept | 0.21 | 0.04 | 0.000 | 1.00 | ne.y | household.relationship.adv |
| AdvInterceptOfSlope | -0.13 | 0.04 | 0.000 | -0.47 | ne.y | household.relationship.adv |
| AdvVarianceOfSlope | 0.08 | 0.09 | 0.378 | 1.00 | ne.y | household.relationship.adv |
| Cnsrct.Intercept_Corr_Adv_Intercept | -0.05 | 0.01 | 0.001 | -0.28 | ne.y | household.relationship.adv |
| Cnsrct.Intercept_Corr_Adv_Slope | 0.01 | 0.02 | 0.653 | 0.08 | ne.y | household.relationship.adv |
| Cnsrct.Slope_Corr_Adv_Intercept | 0.02 | 0.02 | 0.381 | 0.08 | ne.y | household.relationship.adv |
| **Cnsrct.Slope_Corr_Adv_Slope** | **-0.05** | **0.02** | **0.041** | **-0.41** | **ne.y** | **household.relationship.adv** |
| ConstructInterceptOfIntercept | 3.38 | 0.02 | 0.000 | 8.77 | ne.y | body.adv |
| ConstructVarianceOfIntercept | 0.15 | 0.02 | 0.000 | 1.00 | ne.y | body.adv |
| ConstructInterceptOfSlope | 0.28 | 0.03 | 0.000 | 0.75 | ne.y | body.adv |
| ConstructVarianceOfSlope | 0.14 | 0.04 | 0.000 | 1.00 | ne.y | body.adv |
| AdvInterceptOfIntercept | 1.59 | 0.03 | 0.000 | 3.60 | ne.y | body.adv |
| AdvVarianceOfIntercept | 0.20 | 0.04 | 0.000 | 1.00 | ne.y | body.adv |
| AdvInterceptOfSlope | 0.00 | 0.03 | 0.962 | NA | ne.y | body.adv |
| AdvVarianceOfSlope | -0.01 | 0.09 | 0.938 | NA | ne.y | body.adv |
| Cnsrct.Intercept_Corr_Adv_Intercept | -0.05 | 0.01 | 0.000 | -0.30 | ne.y | body.adv |
| Cnsrct.Intercept_Corr_Adv_Slope | 0.02 | 0.02 | 0.204 | 0.71 | ne.y | body.adv |
| Cnsrct.Slope_Corr_Adv_Intercept | -0.03 | 0.02 | 0.102 | -0.17 | ne.y | body.adv |
| Cnsrct.Slope_Corr_Adv_Slope | -0.04 | 0.02 | 0.068 | -1.23 | ne.y | body.adv |
| ConstructInterceptOfIntercept | 3.38 | 0.02 | 0.000 | 8.69 | ne.y | romantic.adv |
| ConstructVarianceOfIntercept | 0.15 | 0.02 | 0.000 | 1.00 | ne.y | romantic.adv |
| ConstructInterceptOfSlope | 0.28 | 0.03 | 0.000 | 0.74 | ne.y | romantic.adv |
| ConstructVarianceOfSlope | 0.15 | 0.04 | 0.000 | 1.00 | ne.y | romantic.adv |
| AdvInterceptOfIntercept | 1.38 | 0.02 | 0.000 | 3.38 | ne.y | romantic.adv |
| AdvVarianceOfIntercept | 0.17 | 0.03 | 0.000 | 1.00 | ne.y | romantic.adv |
| AdvInterceptOfSlope | 0.03 | 0.03 | 0.284 | 0.12 | ne.y | romantic.adv |
| AdvVarianceOfSlope | 0.07 | 0.06 | 0.290 | 1.00 | ne.y | romantic.adv |
| Cnsrct.Intercept_Corr_Adv_Intercept | -0.01 | 0.01 | 0.385 | -0.06 | ne.y | romantic.adv |
| Cnsrct.Intercept_Corr_Adv_Slope | 0.00 | 0.02 | 0.882 | -0.02 | ne.y | romantic.adv |
| **Cnsrct.Slope_Corr_Adv_Intercept** | **-0.03** | **0.01** | **0.047** | **-0.19** | **ne.y** | **romantic.adv** |
| Cnsrct.Slope_Corr_Adv_Slope | 0.01 | 0.02 | 0.455 | 0.14 | ne.y | romantic.adv |
| ConstructInterceptOfIntercept | 3.38 | 0.02 | 0.000 | 8.62 | ne.y | neighborhood.adv |
| ConstructVarianceOfIntercept | 0.15 | 0.02 | 0.000 | 1.00 | ne.y | neighborhood.adv |
| ConstructInterceptOfSlope | 0.28 | 0.03 | 0.000 | 0.71 | ne.y | neighborhood.adv |
| ConstructVarianceOfSlope | 0.16 | 0.04 | 0.000 | 1.00 | ne.y | neighborhood.adv |
| AdvInterceptOfIntercept | 1.52 | 0.03 | 0.000 | 3.50 | ne.y | neighborhood.adv |
| AdvVarianceOfIntercept | 0.19 | 0.04 | 0.000 | 1.00 | ne.y | neighborhood.adv |
| AdvInterceptOfSlope | 0.02 | 0.03 | 0.532 | 0.08 | ne.y | neighborhood.adv |
| AdvVarianceOfSlope | 0.08 | 0.08 | 0.329 | 1.00 | ne.y | neighborhood.adv |
| Cnsrct.Intercept_Corr_Adv_Intercept | -0.01 | 0.01 | 0.444 | -0.06 | ne.y | neighborhood.adv |
| Cnsrct.Intercept_Corr_Adv_Slope | 0.00 | 0.02 | 0.884 | 0.02 | ne.y | neighborhood.adv |
| Cnsrct.Slope_Corr_Adv_Intercept | -0.02 | 0.02 | 0.293 | -0.10 | ne.y | neighborhood.adv |
| Cnsrct.Slope_Corr_Adv_Slope | -0.02 | 0.02 | 0.463 | -0.14 | ne.y | neighborhood.adv |
| ConstructInterceptOfIntercept | 3.38 | 0.02 | 0.000 | 8.63 | ne.y | health.self.adv |
| ConstructVarianceOfIntercept | 0.15 | 0.02 | 0.000 | 1.00 | ne.y | health.self.adv |
| ConstructInterceptOfSlope | 0.28 | 0.03 | 0.000 | 0.72 | ne.y | health.self.adv |
| ConstructVarianceOfSlope | 0.15 | 0.04 | 0.000 | 1.00 | ne.y | health.self.adv |
| AdvInterceptOfIntercept | 1.49 | 0.03 | 0.000 | 3.83 | ne.y | health.self.adv |
| AdvVarianceOfIntercept | 0.15 | 0.04 | 0.000 | 1.00 | ne.y | health.self.adv |
| AdvInterceptOfSlope | -0.09 | 0.03 | 0.006 | -0.71 | ne.y | health.self.adv |
| AdvVarianceOfSlope | 0.02 | 0.08 | 0.825 | 1.00 | ne.y | health.self.adv |
| Cnsrct.Intercept_Corr_Adv_Intercept | 0.00 | 0.01 | 0.886 | 0.01 | ne.y | health.self.adv |
| Cnsrct.Intercept_Corr_Adv_Slope | -0.01 | 0.02 | 0.475 | -0.24 | ne.y | health.self.adv |
| Cnsrct.Slope_Corr_Adv_Intercept | -0.02 | 0.02 | 0.355 | -0.10 | ne.y | health.self.adv |
| Cnsrct.Slope_Corr_Adv_Slope | 0.01 | 0.02 | 0.583 | 0.22 | ne.y | health.self.adv |
| ConstructInterceptOfIntercept | 3.38 | 0.02 | 0.000 | 8.60 | ne.y | health.other.adv |
| ConstructVarianceOfIntercept | 0.15 | 0.02 | 0.000 | 1.00 | ne.y | health.other.adv |
| ConstructInterceptOfSlope | 0.28 | 0.03 | 0.000 | 0.72 | ne.y | health.other.adv |
| ConstructVarianceOfSlope | 0.15 | 0.04 | 0.000 | 1.00 | ne.y | health.other.adv |
| AdvInterceptOfIntercept | 2.08 | 0.04 | 0.000 | 3.42 | ne.y | health.other.adv |
| AdvVarianceOfIntercept | 0.37 | 0.09 | 0.000 | 1.00 | ne.y | health.other.adv |
| AdvInterceptOfSlope | -0.27 | 0.05 | 0.000 | -0.56 | ne.y | health.other.adv |
| AdvVarianceOfSlope | 0.23 | 0.18 | 0.218 | 1.00 | ne.y | health.other.adv |
| Cnsrct.Intercept_Corr_Adv_Intercept | -0.02 | 0.02 | 0.340 | -0.08 | ne.y | health.other.adv |
| Cnsrct.Intercept_Corr_Adv_Slope | 0.03 | 0.03 | 0.204 | 0.18 | ne.y | health.other.adv |
| Cnsrct.Slope_Corr_Adv_Intercept | 0.00 | 0.02 | 0.857 | 0.02 | ne.y | health.other.adv |
| Cnsrct.Slope_Corr_Adv_Slope | -0.05 | 0.03 | 0.139 | -0.26 | ne.y | health.other.adv |
| ConstructInterceptOfIntercept | 3.38 | 0.02 | 0.000 | 8.60 | ne.y | finance.adv |
| ConstructVarianceOfIntercept | 0.15 | 0.02 | 0.000 | 1.00 | ne.y | finance.adv |
| ConstructInterceptOfSlope | 0.28 | 0.03 | 0.000 | 0.70 | ne.y | finance.adv |
| ConstructVarianceOfSlope | 0.16 | 0.04 | 0.000 | 1.00 | ne.y | finance.adv |
| AdvInterceptOfIntercept | 1.53 | 0.03 | 0.000 | 3.70 | ne.y | finance.adv |
| AdvVarianceOfIntercept | 0.17 | 0.04 | 0.000 | 1.00 | ne.y | finance.adv |
| AdvInterceptOfSlope | -0.07 | 0.04 | 0.084 | -0.17 | ne.y | finance.adv |
| AdvVarianceOfSlope | 0.17 | 0.10 | 0.082 | 1.00 | ne.y | finance.adv |
| Cnsrct.Intercept_Corr_Adv_Intercept | -0.03 | 0.01 | 0.045 | -0.17 | ne.y | finance.adv |
| Cnsrct.Intercept_Corr_Adv_Slope | 0.00 | 0.02 | 0.991 | 0.00 | ne.y | finance.adv |
| Cnsrct.Slope_Corr_Adv_Intercept | -0.03 | 0.02 | 0.124 | -0.16 | ne.y | finance.adv |
| Cnsrct.Slope_Corr_Adv_Slope | 0.00 | 0.02 | 0.880 | -0.02 | ne.y | finance.adv |
| ConstructInterceptOfIntercept | 3.38 | 0.02 | 0.000 | 8.63 | ne.y | legal.adv |
| ConstructVarianceOfIntercept | 0.15 | 0.02 | 0.000 | 1.00 | ne.y | legal.adv |
| ConstructInterceptOfSlope | 0.28 | 0.03 | 0.000 | 0.71 | ne.y | legal.adv |
| ConstructVarianceOfSlope | 0.15 | 0.04 | 0.000 | 1.00 | ne.y | legal.adv |
| AdvInterceptOfIntercept | 1.54 | 0.03 | 0.000 | 4.14 | ne.y | legal.adv |
| AdvVarianceOfIntercept | 0.14 | 0.06 | 0.017 | 1.00 | ne.y | legal.adv |
| AdvInterceptOfSlope | -0.19 | 0.04 | 0.000 | -0.69 | ne.y | legal.adv |
| AdvVarianceOfSlope | 0.07 | 0.12 | 0.547 | 1.00 | ne.y | legal.adv |
| Cnsrct.Intercept_Corr_Adv_Intercept | -0.04 | 0.02 | 0.009 | -0.29 | ne.y | legal.adv |
| Cnsrct.Intercept_Corr_Adv_Slope | 0.05 | 0.02 | 0.020 | 0.48 | ne.y | legal.adv |
| Cnsrct.Slope_Corr_Adv_Intercept | -0.01 | 0.02 | 0.746 | -0.04 | ne.y | legal.adv |
| Cnsrct.Slope_Corr_Adv_Slope | -0.04 | 0.03 | 0.187 | -0.33 | ne.y | legal.adv |
| ConstructInterceptOfIntercept | 3.38 | 0.02 | 0.000 | 8.69 | ne.y | violence.adv |
| ConstructVarianceOfIntercept | 0.15 | 0.02 | 0.000 | 1.00 | ne.y | violence.adv |
| ConstructInterceptOfSlope | 0.28 | 0.03 | 0.000 | 0.72 | ne.y | violence.adv |
| ConstructVarianceOfSlope | 0.15 | 0.04 | 0.000 | 1.00 | ne.y | violence.adv |
| AdvInterceptOfIntercept | 1.43 | 0.03 | 0.000 | 3.65 | ne.y | violence.adv |
| AdvVarianceOfIntercept | 0.15 | 0.04 | 0.000 | 1.00 | ne.y | violence.adv |
| AdvInterceptOfSlope | -0.23 | 0.03 | 0.000 | -0.74 | ne.y | violence.adv |
| AdvVarianceOfSlope | 0.09 | 0.08 | 0.248 | 1.00 | ne.y | violence.adv |
| Cnsrct.Intercept_Corr_Adv_Intercept | -0.02 | 0.01 | 0.159 | -0.13 | ne.y | violence.adv |
| Cnsrct.Intercept_Corr_Adv_Slope | 0.03 | 0.02 | 0.116 | 0.23 | ne.y | violence.adv |
| Cnsrct.Slope_Corr_Adv_Intercept | -0.01 | 0.02 | 0.478 | -0.08 | ne.y | violence.adv |
| Cnsrct.Slope_Corr_Adv_Slope | -0.03 | 0.02 | 0.160 | -0.26 | ne.y | violence.adv |
| ConstructInterceptOfIntercept | 3.38 | 0.02 | 0.000 | 8.67 | ne.y | discrimination.adv |
| ConstructVarianceOfIntercept | 0.15 | 0.02 | 0.000 | 1.00 | ne.y | discrimination.adv |
| ConstructInterceptOfSlope | 0.28 | 0.03 | 0.000 | 0.72 | ne.y | discrimination.adv |
| ConstructVarianceOfSlope | 0.15 | 0.04 | 0.000 | 1.00 | ne.y | discrimination.adv |
| AdvInterceptOfIntercept | 1.22 | 0.02 | 0.000 | 5.12 | ne.y | discrimination.adv |
| AdvVarianceOfIntercept | 0.06 | 0.02 | 0.003 | 1.00 | ne.y | discrimination.adv |
| AdvInterceptOfSlope | -0.01 | 0.03 | 0.822 | -0.02 | ne.y | discrimination.adv |
| **AdvVarianceOfSlope** | **0.13** | **0.04** | **0.002** | **1.00** | **ne.y** | **discrimination.adv** |
| Cnsrct.Intercept_Corr_Adv_Intercept | -0.02 | 0.01 | 0.015 | -0.24 | ne.y | discrimination.adv |
| Cnsrct.Intercept_Corr_Adv_Slope | 0.02 | 0.01 | 0.150 | 0.13 | ne.y | discrimination.adv |
| Cnsrct.Slope_Corr_Adv_Intercept | -0.01 | 0.01 | 0.411 | -0.10 | ne.y | discrimination.adv |
| Cnsrct.Slope_Corr_Adv_Slope | 0.01 | 0.02 | 0.615 | 0.06 | ne.y | discrimination.adv |
| ConstructInterceptOfIntercept | 3.38 | 0.02 | 0.000 | 8.64 | ne.y | activities.adv |
| ConstructVarianceOfIntercept | 0.15 | 0.02 | 0.000 | 1.00 | ne.y | activities.adv |
| ConstructInterceptOfSlope | 0.28 | 0.03 | 0.000 | 0.74 | ne.y | activities.adv |
| ConstructVarianceOfSlope | 0.15 | 0.04 | 0.000 | 1.00 | ne.y | activities.adv |
| AdvInterceptOfIntercept | 1.35 | 0.02 | 0.000 | 5.89 | ne.y | activities.adv |
| AdvVarianceOfIntercept | 0.05 | 0.02 | 0.009 | 1.00 | ne.y | activities.adv |
| AdvInterceptOfSlope | -0.10 | 0.02 | 0.000 | -1.67 | ne.y | activities.adv |
| AdvVarianceOfSlope | 0.00 | 0.04 | 0.932 | 1.00 | ne.y | activities.adv |
| Cnsrct.Intercept_Corr_Adv_Intercept | -0.01 | 0.01 | 0.269 | -0.11 | ne.y | activities.adv |
| Cnsrct.Intercept_Corr_Adv_Slope | 0.01 | 0.01 | 0.607 | 0.27 | ne.y | activities.adv |
| Cnsrct.Slope_Corr_Adv_Intercept | -0.02 | 0.01 | 0.125 | -0.19 | ne.y | activities.adv |
| Cnsrct.Slope_Corr_Adv_Slope | -0.02 | 0.01 | 0.216 | -0.77 | ne.y | activities.adv |
| ConstructInterceptOfIntercept | 3.45 | 0.02 | 0.000 | 7.65 | ne.p | academic.adv |
| ConstructVarianceOfIntercept | 0.20 | 0.02 | 0.000 | 1.00 | ne.p | academic.adv |
| ConstructInterceptOfSlope | 0.24 | 0.02 | 0.000 | 0.66 | ne.p | academic.adv |
| ConstructVarianceOfSlope | 0.13 | 0.03 | 0.000 | 1.00 | ne.p | academic.adv |
| AdvInterceptOfIntercept | 1.79 | 0.03 | 0.000 | 3.37 | ne.p | academic.adv |
| AdvVarianceOfIntercept | 0.28 | 0.05 | 0.000 | 1.00 | ne.p | academic.adv |
| AdvInterceptOfSlope | 0.05 | 0.04 | 0.137 | 0.13 | ne.p | academic.adv |
| AdvVarianceOfSlope | 0.17 | 0.10 | 0.073 | 1.00 | ne.p | academic.adv |
| Cnsrct.Intercept_Corr_Adv_Intercept | -0.05 | 0.01 | 0.000 | -0.22 | ne.p | academic.adv |
| Cnsrct.Intercept_Corr_Adv_Slope | 0.02 | 0.02 | 0.254 | 0.10 | ne.p | academic.adv |
| Cnsrct.Slope_Corr_Adv_Intercept | 0.00 | 0.01 | 0.874 | -0.01 | ne.p | academic.adv |
| **Cnsrct.Slope_Corr_Adv_Slope** | **-0.04** | **0.02** | **0.008** | **-0.29** | **ne.p** | **academic.adv** |
| ConstructInterceptOfIntercept | 3.45 | 0.02 | 0.000 | 7.74 | ne.p | behavioral.adv |
| ConstructVarianceOfIntercept | 0.20 | 0.02 | 0.000 | 1.00 | ne.p | behavioral.adv |
| ConstructInterceptOfSlope | 0.24 | 0.02 | 0.000 | 0.69 | ne.p | behavioral.adv |
| ConstructVarianceOfSlope | 0.12 | 0.03 | 0.000 | 1.00 | ne.p | behavioral.adv |
| AdvInterceptOfIntercept | 1.42 | 0.03 | 0.000 | 3.21 | ne.p | behavioral.adv |
| AdvVarianceOfIntercept | 0.20 | 0.03 | 0.000 | 1.00 | ne.p | behavioral.adv |
| AdvInterceptOfSlope | -0.24 | 0.03 | 0.000 | -1.25 | ne.p | behavioral.adv |
| AdvVarianceOfSlope | 0.04 | 0.07 | 0.595 | 1.00 | ne.p | behavioral.adv |
| Cnsrct.Intercept_Corr_Adv_Intercept | -0.03 | 0.01 | 0.008 | -0.17 | ne.p | behavioral.adv |
| Cnsrct.Intercept_Corr_Adv_Slope | -0.01 | 0.02 | 0.636 | -0.08 | ne.p | behavioral.adv |
| Cnsrct.Slope_Corr_Adv_Intercept | 0.01 | 0.01 | 0.297 | 0.08 | ne.p | behavioral.adv |
| Cnsrct.Slope_Corr_Adv_Slope | -0.02 | 0.01 | 0.095 | -0.36 | ne.p | behavioral.adv |
| ConstructInterceptOfIntercept | 3.44 | 0.02 | 0.000 | 7.64 | ne.p | peer.adv |
| ConstructVarianceOfIntercept | 0.20 | 0.02 | 0.000 | 1.00 | ne.p | peer.adv |
| ConstructInterceptOfSlope | 0.24 | 0.02 | 0.000 | 0.65 | ne.p | peer.adv |
| ConstructVarianceOfSlope | 0.14 | 0.03 | 0.000 | 1.00 | ne.p | peer.adv |
| AdvInterceptOfIntercept | 1.90 | 0.03 | 0.000 | 3.52 | ne.p | peer.adv |
| AdvVarianceOfIntercept | 0.29 | 0.05 | 0.000 | 1.00 | ne.p | peer.adv |
| AdvInterceptOfSlope | -0.22 | 0.04 | 0.000 | -0.56 | ne.p | peer.adv |
| AdvVarianceOfSlope | 0.15 | 0.09 | 0.076 | 1.00 | ne.p | peer.adv |
| Cnsrct.Intercept_Corr_Adv_Intercept | -0.06 | 0.01 | 0.000 | -0.26 | ne.p | peer.adv |
| Cnsrct.Intercept_Corr_Adv_Slope | 0.05 | 0.02 | 0.004 | 0.27 | ne.p | peer.adv |
| Cnsrct.Slope_Corr_Adv_Intercept | 0.01 | 0.01 | 0.457 | 0.05 | ne.p | peer.adv |
| **Cnsrct.Slope_Corr_Adv_Slope** | **-0.04** | **0.02** | **0.026** | **-0.25** | **ne.p** | **peer.adv** |
| ConstructInterceptOfIntercept | 3.44 | 0.02 | 0.000 | 7.62 | ne.p | parentchild.relationship.adv |
| ConstructVarianceOfIntercept | 0.20 | 0.02 | 0.000 | 1.00 | ne.p | parentchild.relationship.adv |
| ConstructInterceptOfSlope | 0.24 | 0.02 | 0.000 | 0.64 | ne.p | parentchild.relationship.adv |
| ConstructVarianceOfSlope | 0.14 | 0.03 | 0.000 | 1.00 | ne.p | parentchild.relationship.adv |
| AdvInterceptOfIntercept | 1.95 | 0.03 | 0.000 | 4.13 | ne.p | parentchild.relationship.adv |
| AdvVarianceOfIntercept | 0.22 | 0.04 | 0.000 | 1.00 | ne.p | parentchild.relationship.adv |
| AdvInterceptOfSlope | -0.05 | 0.04 | 0.173 | -0.11 | ne.p | parentchild.relationship.adv |
| **AdvVarianceOfSlope** | **0.20** | **0.09** | **0.028** | **1.00** | **ne.p** | **parentchild.relationship.adv** |
| Cnsrct.Intercept_Corr_Adv_Intercept | -0.05 | 0.01 | 0.000 | -0.25 | ne.p | parentchild.relationship.adv |
| Cnsrct.Intercept_Corr_Adv_Slope | -0.01 | 0.02 | 0.709 | -0.03 | ne.p | parentchild.relationship.adv |
| Cnsrct.Slope_Corr_Adv_Intercept | -0.02 | 0.01 | 0.219 | -0.09 | ne.p | parentchild.relationship.adv |
| Cnsrct.Slope_Corr_Adv_Slope | 0.00 | 0.02 | 0.838 | -0.02 | ne.p | parentchild.relationship.adv |
| ConstructInterceptOfIntercept | 3.44 | 0.02 | 0.000 | 7.59 | ne.p | parentparent.relationship.adv |
| ConstructVarianceOfIntercept | 0.21 | 0.02 | 0.000 | 1.00 | ne.p | parentparent.relationship.adv |
| ConstructInterceptOfSlope | 0.24 | 0.02 | 0.000 | 0.64 | ne.p | parentparent.relationship.adv |
| ConstructVarianceOfSlope | 0.14 | 0.03 | 0.000 | 1.00 | ne.p | parentparent.relationship.adv |
| AdvInterceptOfIntercept | 1.85 | 0.03 | 0.000 | 3.06 | ne.p | parentparent.relationship.adv |
| AdvVarianceOfIntercept | 0.36 | 0.06 | 0.000 | 1.00 | ne.p | parentparent.relationship.adv |
| AdvInterceptOfSlope | -0.11 | 0.04 | 0.009 | -0.16 | ne.p | parentparent.relationship.adv |
| **AdvVarianceOfSlope** | **0.43** | **0.11** | **0.000** | **1.00** | **ne.p** | **parentparent.relationship.adv** |
| Cnsrct.Intercept_Corr_Adv_Intercept | -0.02 | 0.02 | 0.245 | -0.06 | ne.p | parentparent.relationship.adv |
| Cnsrct.Intercept_Corr_Adv_Slope | 0.00 | 0.02 | 0.940 | -0.01 | ne.p | parentparent.relationship.adv |
| Cnsrct.Slope_Corr_Adv_Intercept | -0.02 | 0.01 | 0.194 | -0.08 | ne.p | parentparent.relationship.adv |
| Cnsrct.Slope_Corr_Adv_Slope | 0.01 | 0.02 | 0.682 | 0.03 | ne.p | parentparent.relationship.adv |
| ConstructInterceptOfIntercept | 3.45 | 0.02 | 0.000 | 7.67 | ne.p | household.relationship.adv |
| ConstructVarianceOfIntercept | 0.20 | 0.02 | 0.000 | 1.00 | ne.p | household.relationship.adv |
| ConstructInterceptOfSlope | 0.24 | 0.02 | 0.000 | 0.67 | ne.p | household.relationship.adv |
| ConstructVarianceOfSlope | 0.13 | 0.03 | 0.000 | 1.00 | ne.p | household.relationship.adv |
| AdvInterceptOfIntercept | 2.06 | 0.03 | 0.000 | 4.45 | ne.p | household.relationship.adv |
| AdvVarianceOfIntercept | 0.21 | 0.04 | 0.000 | 1.00 | ne.p | household.relationship.adv |
| AdvInterceptOfSlope | -0.13 | 0.04 | 0.000 | -0.47 | ne.p | household.relationship.adv |
| AdvVarianceOfSlope | 0.08 | 0.09 | 0.372 | 1.00 | ne.p | household.relationship.adv |
| Cnsrct.Intercept_Corr_Adv_Intercept | -0.03 | 0.01 | 0.037 | -0.14 | ne.p | household.relationship.adv |
| Cnsrct.Intercept_Corr_Adv_Slope | -0.04 | 0.02 | 0.044 | -0.28 | ne.p | household.relationship.adv |
| Cnsrct.Slope_Corr_Adv_Intercept | -0.01 | 0.01 | 0.260 | -0.09 | ne.p | household.relationship.adv |
| Cnsrct.Slope_Corr_Adv_Slope | -0.02 | 0.02 | 0.246 | -0.19 | ne.p | household.relationship.adv |
| ConstructInterceptOfIntercept | 3.44 | 0.02 | 0.000 | 7.58 | ne.p | body.adv |
| ConstructVarianceOfIntercept | 0.21 | 0.02 | 0.000 | 1.00 | ne.p | body.adv |
| ConstructInterceptOfSlope | 0.24 | 0.02 | 0.000 | 0.64 | ne.p | body.adv |
| ConstructVarianceOfSlope | 0.14 | 0.03 | 0.000 | 1.00 | ne.p | body.adv |
| AdvInterceptOfIntercept | 1.59 | 0.03 | 0.000 | 3.66 | ne.p | body.adv |
| AdvVarianceOfIntercept | 0.19 | 0.04 | 0.000 | 1.00 | ne.p | body.adv |
| AdvInterceptOfSlope | 0.00 | 0.03 | 0.964 | NA | ne.p | body.adv |
| AdvVarianceOfSlope | -0.02 | 0.09 | 0.795 | NA | ne.p | body.adv |
| Cnsrct.Intercept_Corr_Adv_Intercept | -0.01 | 0.01 | 0.693 | -0.03 | ne.p | body.adv |
| Cnsrct.Intercept_Corr_Adv_Slope | 0.00 | 0.02 | 0.945 | 0.02 | ne.p | body.adv |
| Cnsrct.Slope_Corr_Adv_Intercept | 0.00 | 0.01 | 0.885 | 0.01 | ne.p | body.adv |
| **Cnsrct.Slope_Corr_Adv_Slope** | **-0.03** | **0.02** | **0.049** | **-0.54** | **ne.p** | **body.adv** |
| ConstructInterceptOfIntercept | 3.44 | 0.02 | 0.000 | 7.58 | ne.p | romantic.adv |
| ConstructVarianceOfIntercept | 0.21 | 0.02 | 0.000 | 1.00 | ne.p | romantic.adv |
| ConstructInterceptOfSlope | 0.24 | 0.02 | 0.000 | 0.64 | ne.p | romantic.adv |
| ConstructVarianceOfSlope | 0.14 | 0.03 | 0.000 | 1.00 | ne.p | romantic.adv |
| AdvInterceptOfIntercept | 1.38 | 0.02 | 0.000 | 3.39 | ne.p | romantic.adv |
| AdvVarianceOfIntercept | 0.16 | 0.03 | 0.000 | 1.00 | ne.p | romantic.adv |
| AdvInterceptOfSlope | 0.03 | 0.03 | 0.283 | 0.12 | ne.p | romantic.adv |
| AdvVarianceOfSlope | 0.06 | 0.06 | 0.318 | 1.00 | ne.p | romantic.adv |
| Cnsrct.Intercept_Corr_Adv_Intercept | 0.01 | 0.01 | 0.502 | 0.04 | ne.p | romantic.adv |
| Cnsrct.Intercept_Corr_Adv_Slope | -0.01 | 0.01 | 0.441 | -0.09 | ne.p | romantic.adv |
| Cnsrct.Slope_Corr_Adv_Intercept | -0.01 | 0.01 | 0.361 | -0.07 | ne.p | romantic.adv |
| Cnsrct.Slope_Corr_Adv_Slope | 0.01 | 0.01 | 0.666 | 0.06 | ne.p | romantic.adv |
| ConstructInterceptOfIntercept | 3.44 | 0.02 | 0.000 | 7.57 | ne.p | neighborhood.adv |
| ConstructVarianceOfIntercept | 0.21 | 0.02 | 0.000 | 1.00 | ne.p | neighborhood.adv |
| ConstructInterceptOfSlope | 0.24 | 0.02 | 0.000 | 0.63 | ne.p | neighborhood.adv |
| ConstructVarianceOfSlope | 0.14 | 0.03 | 0.000 | 1.00 | ne.p | neighborhood.adv |
| AdvInterceptOfIntercept | 1.52 | 0.03 | 0.000 | 3.51 | ne.p | neighborhood.adv |
| AdvVarianceOfIntercept | 0.19 | 0.04 | 0.000 | 1.00 | ne.p | neighborhood.adv |
| AdvInterceptOfSlope | 0.02 | 0.03 | 0.537 | 0.08 | ne.p | neighborhood.adv |
| AdvVarianceOfSlope | 0.08 | 0.08 | 0.322 | 1.00 | ne.p | neighborhood.adv |
| Cnsrct.Intercept_Corr_Adv_Intercept | 0.01 | 0.01 | 0.266 | 0.07 | ne.p | neighborhood.adv |
| Cnsrct.Intercept_Corr_Adv_Slope | -0.01 | 0.02 | 0.679 | -0.05 | ne.p | neighborhood.adv |
| Cnsrct.Slope_Corr_Adv_Intercept | -0.01 | 0.01 | 0.482 | -0.05 | ne.p | neighborhood.adv |
| Cnsrct.Slope_Corr_Adv_Slope | -0.01 | 0.02 | 0.364 | -0.13 | ne.p | neighborhood.adv |
| ConstructInterceptOfIntercept | 3.44 | 0.02 | 0.000 | 7.54 | ne.p | health.self.adv |
| ConstructVarianceOfIntercept | 0.21 | 0.02 | 0.000 | 1.00 | ne.p | health.self.adv |
| ConstructInterceptOfSlope | 0.24 | 0.02 | 0.000 | 0.63 | ne.p | health.self.adv |
| ConstructVarianceOfSlope | 0.15 | 0.03 | 0.000 | 1.00 | ne.p | health.self.adv |
| AdvInterceptOfIntercept | 1.49 | 0.03 | 0.000 | 3.77 | ne.p | health.self.adv |
| AdvVarianceOfIntercept | 0.16 | 0.04 | 0.000 | 1.00 | ne.p | health.self.adv |
| AdvInterceptOfSlope | -0.10 | 0.03 | 0.005 | -0.57 | ne.p | health.self.adv |
| AdvVarianceOfSlope | 0.03 | 0.08 | 0.717 | 1.00 | ne.p | health.self.adv |
| Cnsrct.Intercept_Corr_Adv_Intercept | 0.02 | 0.01 | 0.080 | 0.12 | ne.p | health.self.adv |
| Cnsrct.Intercept_Corr_Adv_Slope | -0.01 | 0.02 | 0.710 | -0.08 | ne.p | health.self.adv |
| Cnsrct.Slope_Corr_Adv_Intercept | -0.01 | 0.01 | 0.525 | -0.05 | ne.p | health.self.adv |
| Cnsrct.Slope_Corr_Adv_Slope | 0.00 | 0.02 | 0.786 | 0.06 | ne.p | health.self.adv |
| ConstructInterceptOfIntercept | 3.44 | 0.02 | 0.000 | 7.55 | ne.p | health.other.adv |
| ConstructVarianceOfIntercept | 0.21 | 0.02 | 0.000 | 1.00 | ne.p | health.other.adv |
| ConstructInterceptOfSlope | 0.24 | 0.02 | 0.000 | 0.63 | ne.p | health.other.adv |
| ConstructVarianceOfSlope | 0.14 | 0.03 | 0.000 | 1.00 | ne.p | health.other.adv |
| AdvInterceptOfIntercept | 2.08 | 0.04 | 0.000 | 3.46 | ne.p | health.other.adv |
| AdvVarianceOfIntercept | 0.36 | 0.09 | 0.000 | 1.00 | ne.p | health.other.adv |
| AdvInterceptOfSlope | -0.27 | 0.05 | 0.000 | -0.58 | ne.p | health.other.adv |
| AdvVarianceOfSlope | 0.21 | 0.18 | 0.263 | 1.00 | ne.p | health.other.adv |
| Cnsrct.Intercept_Corr_Adv_Intercept | 0.02 | 0.02 | 0.258 | 0.08 | ne.p | health.other.adv |
| Cnsrct.Intercept_Corr_Adv_Slope | 0.01 | 0.02 | 0.746 | 0.04 | ne.p | health.other.adv |
| Cnsrct.Slope_Corr_Adv_Intercept | -0.03 | 0.02 | 0.059 | -0.15 | ne.p | health.other.adv |
| Cnsrct.Slope_Corr_Adv_Slope | 0.00 | 0.02 | 0.979 | 0.00 | ne.p | health.other.adv |
| ConstructInterceptOfIntercept | 3.44 | 0.02 | 0.000 | 7.61 | ne.p | finance.adv |
| ConstructVarianceOfIntercept | 0.20 | 0.02 | 0.000 | 1.00 | ne.p | finance.adv |
| ConstructInterceptOfSlope | 0.24 | 0.02 | 0.000 | 0.64 | ne.p | finance.adv |
| ConstructVarianceOfSlope | 0.14 | 0.03 | 0.000 | 1.00 | ne.p | finance.adv |
| AdvInterceptOfIntercept | 1.53 | 0.03 | 0.000 | 3.70 | ne.p | finance.adv |
| AdvVarianceOfIntercept | 0.17 | 0.04 | 0.000 | 1.00 | ne.p | finance.adv |
| AdvInterceptOfSlope | -0.07 | 0.04 | 0.080 | -0.16 | ne.p | finance.adv |
| AdvVarianceOfSlope | 0.18 | 0.10 | 0.067 | 1.00 | ne.p | finance.adv |
| Cnsrct.Intercept_Corr_Adv_Intercept | -0.03 | 0.01 | 0.047 | -0.14 | ne.p | finance.adv |
| Cnsrct.Intercept_Corr_Adv_Slope | 0.02 | 0.02 | 0.320 | 0.10 | ne.p | finance.adv |
| Cnsrct.Slope_Corr_Adv_Intercept | -0.01 | 0.01 | 0.495 | -0.06 | ne.p | finance.adv |
| Cnsrct.Slope_Corr_Adv_Slope | -0.03 | 0.02 | 0.146 | -0.17 | ne.p | finance.adv |
| ConstructInterceptOfIntercept | 3.44 | 0.02 | 0.000 | 7.57 | ne.p | legal.adv |
| ConstructVarianceOfIntercept | 0.21 | 0.02 | 0.000 | 1.00 | ne.p | legal.adv |
| ConstructInterceptOfSlope | 0.24 | 0.02 | 0.000 | 0.63 | ne.p | legal.adv |
| ConstructVarianceOfSlope | 0.14 | 0.03 | 0.000 | 1.00 | ne.p | legal.adv |
| AdvInterceptOfIntercept | 1.54 | 0.03 | 0.000 | 4.08 | ne.p | legal.adv |
| AdvVarianceOfIntercept | 0.14 | 0.06 | 0.014 | 1.00 | ne.p | legal.adv |
| AdvInterceptOfSlope | -0.19 | 0.04 | 0.000 | -0.65 | ne.p | legal.adv |
| AdvVarianceOfSlope | 0.08 | 0.12 | 0.499 | 1.00 | ne.p | legal.adv |
| Cnsrct.Intercept_Corr_Adv_Intercept | -0.02 | 0.02 | 0.108 | -0.14 | ne.p | legal.adv |
| Cnsrct.Intercept_Corr_Adv_Slope | 0.01 | 0.02 | 0.560 | 0.09 | ne.p | legal.adv |
| Cnsrct.Slope_Corr_Adv_Intercept | 0.00 | 0.02 | 0.868 | -0.02 | ne.p | legal.adv |
| Cnsrct.Slope_Corr_Adv_Slope | -0.01 | 0.02 | 0.709 | -0.07 | ne.p | legal.adv |
| ConstructInterceptOfIntercept | 3.44 | 0.02 | 0.000 | 7.60 | ne.p | violence.adv |
| ConstructVarianceOfIntercept | 0.21 | 0.02 | 0.000 | 1.00 | ne.p | violence.adv |
| ConstructInterceptOfSlope | 0.24 | 0.02 | 0.000 | 0.65 | ne.p | violence.adv |
| ConstructVarianceOfSlope | 0.14 | 0.03 | 0.000 | 1.00 | ne.p | violence.adv |
| AdvInterceptOfIntercept | 1.43 | 0.03 | 0.000 | 3.74 | ne.p | violence.adv |
| AdvVarianceOfIntercept | 0.15 | 0.04 | 0.000 | 1.00 | ne.p | violence.adv |
| AdvInterceptOfSlope | -0.23 | 0.03 | 0.000 | -0.75 | ne.p | violence.adv |
| AdvVarianceOfSlope | 0.09 | 0.08 | 0.271 | 1.00 | ne.p | violence.adv |
| Cnsrct.Intercept_Corr_Adv_Intercept | -0.05 | 0.01 | 0.001 | -0.26 | ne.p | violence.adv |
| Cnsrct.Intercept_Corr_Adv_Slope | 0.03 | 0.02 | 0.053 | 0.23 | ne.p | violence.adv |
| Cnsrct.Slope_Corr_Adv_Intercept | 0.01 | 0.01 | 0.335 | 0.09 | ne.p | violence.adv |
| **Cnsrct.Slope_Corr_Adv_Slope** | **-0.03** | **0.02** | **0.029** | **-0.31** | **ne.p** | **violence.adv** |
| ConstructInterceptOfIntercept | 3.44 | 0.02 | 0.000 | 7.59 | ne.p | discrimination.adv |
| ConstructVarianceOfIntercept | 0.21 | 0.02 | 0.000 | 1.00 | ne.p | discrimination.adv |
| ConstructInterceptOfSlope | 0.24 | 0.02 | 0.000 | 0.64 | ne.p | discrimination.adv |
| ConstructVarianceOfSlope | 0.14 | 0.03 | 0.000 | 1.00 | ne.p | discrimination.adv |
| AdvInterceptOfIntercept | 1.22 | 0.02 | 0.000 | 5.01 | ne.p | discrimination.adv |
| AdvVarianceOfIntercept | 0.06 | 0.02 | 0.002 | 1.00 | ne.p | discrimination.adv |
| AdvInterceptOfSlope | 0.00 | 0.03 | 0.889 | -0.01 | ne.p | discrimination.adv |
| **AdvVarianceOfSlope** | **0.13** | **0.04** | **0.001** | **1.00** | **ne.p** | **discrimination.adv** |
| Cnsrct.Intercept_Corr_Adv_Intercept | 0.01 | 0.01 | 0.304 | 0.08 | ne.p | discrimination.adv |
| Cnsrct.Intercept_Corr_Adv_Slope | -0.02 | 0.01 | 0.126 | -0.11 | ne.p | discrimination.adv |
| Cnsrct.Slope_Corr_Adv_Intercept | 0.01 | 0.01 | 0.336 | 0.09 | ne.p | discrimination.adv |
| Cnsrct.Slope_Corr_Adv_Slope | -0.02 | 0.01 | 0.156 | -0.12 | ne.p | discrimination.adv |
| ConstructInterceptOfIntercept | 3.44 | 0.02 | 0.000 | 7.58 | ne.p | activities.adv |
| ConstructVarianceOfIntercept | 0.21 | 0.02 | 0.000 | 1.00 | ne.p | activities.adv |
| ConstructInterceptOfSlope | 0.24 | 0.02 | 0.000 | 0.64 | ne.p | activities.adv |
| ConstructVarianceOfSlope | 0.14 | 0.03 | 0.000 | 1.00 | ne.p | activities.adv |
| AdvInterceptOfIntercept | 1.35 | 0.02 | 0.000 | 5.86 | ne.p | activities.adv |
| AdvVarianceOfIntercept | 0.05 | 0.02 | 0.009 | 1.00 | ne.p | activities.adv |
| AdvInterceptOfSlope | -0.10 | 0.02 | 0.000 | -1.00 | ne.p | activities.adv |
| AdvVarianceOfSlope | 0.01 | 0.04 | 0.817 | 1.00 | ne.p | activities.adv |
| Cnsrct.Intercept_Corr_Adv_Intercept | 0.00 | 0.01 | 0.861 | 0.01 | ne.p | activities.adv |
| Cnsrct.Intercept_Corr_Adv_Slope | -0.01 | 0.01 | 0.407 | -0.21 | ne.p | activities.adv |
| Cnsrct.Slope_Corr_Adv_Intercept | 0.00 | 0.01 | 0.590 | -0.05 | ne.p | activities.adv |
| Cnsrct.Slope_Corr_Adv_Slope | -0.01 | 0.01 | 0.384 | -0.25 | ne.p | activities.adv |

# Table S19a. uv.onPCR.adv.fits.indices.csv

| chisq | df | rmsea | tli | cfi | ModID |
| --- | --- | --- | --- | --- | --- |
| 34.606 | 5 | 0.093 | 0.897 | 0.957 | ec.y_uv.onPCR.adv |
| 30.664 | 5 | 0.087 | 0.946 | 0.977 | ec.p_uv.onPCR.adv |
| 12.817 | 5 | 0.048 | 0.973 | 0.989 | ac.y_uv.onPCR.adv |
| 21.978 | 5 | 0.071 | 0.958 | 0.982 | ac.p_uv.onPCR.adv |
| 22.824 | 5 | 0.072 | 0.89 | 0.954 | at.y_uv.onPCR.adv |
| 13.037 | 5 | 0.049 | 0.975 | 0.989 | at.p_uv.onPCR.adv |
| 24.468 | 5 | 0.076 | 0.865 | 0.944 | ic.y_uv.onPCR.adv |
| 19.619 | 5 | 0.065 | 0.943 | 0.976 | ic.p_uv.onPCR.adv |
| 93.248 | 5 | 0.161 | 0.471 | 0.779 | ne.y_uv.onPCR.adv |
| 43.814 | 5 | 0.107 | 0.903 | 0.96 | ne.p_uv.onPCR.adv |
| 26.657 | 5 | 0.08 | 0.901 | 0.959 | ag.y_uv.onPCR.adv |
| 22.441 | 5 | 0.072 | 0.95 | 0.979 | ag.p_uv.onPCR.adv |
| 78.811 | 5 | 0.147 | 0.478 | 0.783 | fear.y_uv.onPCR.adv |
| 44.953 | 5 | 0.108 | 0.865 | 0.944 | fear.p_uv.onPCR.adv |
| 44.585 | 5 | 0.108 | 0.704 | 0.877 | fr.y_uv.onPCR.adv |
| 20.113 | 5 | 0.067 | 0.947 | 0.978 | fr.p_uv.onPCR.adv |
| 41.638 | 5 | 0.104 | 0.798 | 0.916 | shy.y_uv.onPCR.adv |
| 4.137 | 5 | 0 | 1.002 | 1 | shy.p_uv.onPCR.adv |

# Table S19b. uv.onPPR.adv.fits.indices.csv

| chisq | df | rmsea | tli | cfi | ModID |
| --- | --- | --- | --- | --- | --- |
| 37.183 | 5 | 0.097 | 0.881 | 0.95 | ec.y_uv.onPPR.adv |
| 30.328 | 5 | 0.086 | 0.945 | 0.977 | ec.p_uv.onPPR.adv |
| 12.016 | 5 | 0.045 | 0.975 | 0.99 | ac.y_uv.onPPR.adv |
| 23.179 | 5 | 0.073 | 0.954 | 0.981 | ac.p_uv.onPPR.adv |
| 22.799 | 5 | 0.072 | 0.879 | 0.95 | at.y_uv.onPPR.adv |
| 7.616 | 5 | 0.028 | 0.992 | 0.996 | at.p_uv.onPPR.adv |
| 32.986 | 5 | 0.091 | 0.793 | 0.914 | ic.y_uv.onPPR.adv |
| 25.019 | 5 | 0.077 | 0.92 | 0.966 | ic.p_uv.onPPR.adv |
| 88.322 | 5 | 0.156 | 0.436 | 0.765 | ne.y_uv.onPPR.adv |
| 42.42 | 5 | 0.105 | 0.902 | 0.959 | ne.p_uv.onPPR.adv |
| 20.122 | 5 | 0.067 | 0.924 | 0.968 | ag.y_uv.onPPR.adv |
| 22.187 | 5 | 0.071 | 0.948 | 0.978 | ag.p_uv.onPPR.adv |
| 77.77 | 5 | 0.146 | 0.479 | 0.783 | fear.y_uv.onPPR.adv |
| 42.975 | 5 | 0.106 | 0.869 | 0.945 | fear.p_uv.onPPR.adv |
| 44.211 | 5 | 0.107 | 0.678 | 0.866 | fr.y_uv.onPPR.adv |
| 20.72 | 5 | 0.068 | 0.941 | 0.975 | fr.p_uv.onPPR.adv |
| 39.174 | 5 | 0.1 | 0.808 | 0.92 | shy.y_uv.onPPR.adv |
| 2.36 | 5 | 0 | 1.007 | 1 | shy.p_uv.onPPR.adv |

# Table S19c. uv.onDisc.adv.fits.indices.csv

| chisq | df | rmsea | tli | cfi | ModID |
| --- | --- | --- | --- | --- | --- |
| 38.465 | 5 | 0.099 | 0.87 | 0.946 | ec.y_uv.onDisc.adv |
| 25.929 | 5 | 0.078 | 0.954 | 0.981 | ec.p_uv.onDisc.adv |
| 13.71 | 5 | 0.051 | 0.968 | 0.987 | ac.y_uv.onDisc.adv |
| 20.417 | 5 | 0.067 | 0.96 | 0.983 | ac.p_uv.onDisc.adv |
| 28.892 | 5 | 0.084 | 0.833 | 0.93 | at.y_uv.onDisc.adv |
| 8.159 | 5 | 0.03 | 0.99 | 0.996 | at.p_uv.onDisc.adv |
| 30.003 | 5 | 0.086 | 0.8 | 0.917 | ic.y_uv.onDisc.adv |
| 19.01 | 5 | 0.064 | 0.942 | 0.976 | ic.p_uv.onDisc.adv |
| 88.555 | 5 | 0.157 | 0.428 | 0.762 | ne.y_uv.onDisc.adv |
| 43.751 | 5 | 0.107 | 0.899 | 0.958 | ne.p_uv.onDisc.adv |
| 21.743 | 5 | 0.07 | 0.914 | 0.964 | ag.y_uv.onDisc.adv |
| 21.065 | 5 | 0.069 | 0.951 | 0.979 | ag.p_uv.onDisc.adv |
| 81.231 | 5 | 0.15 | 0.465 | 0.777 | fear.y_uv.onDisc.adv |
| 43.125 | 5 | 0.106 | 0.87 | 0.946 | fear.p_uv.onDisc.adv |
| 46.682 | 5 | 0.111 | 0.66 | 0.858 | fr.y_uv.onDisc.adv |
| 21.91 | 5 | 0.07 | 0.937 | 0.974 | fr.p_uv.onDisc.adv |
| 44.088 | 5 | 0.107 | 0.782 | 0.909 | shy.y_uv.onDisc.adv |
| 17.126 | 5 | 0.06 | 0.969 | 0.987 | shy.p_uv.onDisc.adv |

# Table S20a. Youth and Parent Report Estimates for Prevalence of Growth Despite Parent-Child Conflict (Estimates for Univariate Model Conditioned on Initial Adversity, Change in Adversity, and Cohort)

|  | Low Adversity | | % Change >/= SESOI despite baseline Adversity | | % Change >/= SESOI despite increases in Adversity | |
| --- | --- | --- | --- | --- | --- | --- |
| Model | Decrease | Increase | Decrease | Increase | Decrease | Increase |
| ec.y_bv | 16% (63/404) | 50% (203/404) | 22% (34/155) | 41% (63/155) | 38% (43/114) | 22% (25/114) |
| ec.p_bv | 20% (82/404) | 49% (196/404) | 17% (26/155) | 49% (76/155) | 23% (26/114) | 47% (54/114) |
| ac.y_bv | 41% (167/404) | 17% (67/404) | 46% (72/155) | 14% (21/155) | 68% (77/114) | 7% (8/114) |
| ac.p_bv | 27% (111/404) | 28% (115/404) | 17% (26/155) | 32% (49/155) | 32% (36/114) | 23% (26/114) |
| at.y_bv | 17% (70/404) | 54% (218/404) | 22% (34/155) | 45% (69/155) | 34% (39/114) | 37% (42/114) |
| at.p_bv | 6% (24/404) | 48% (195/404) | 6% (9/155) | 53% (82/155) | 11% (12/114) | 50% (57/114) |
| ic.y_bv | 0% (0/404) | 97% (393/404) | 0% (0/155) | 85% (131/155) | 0% (0/114) | 71% (81/114) |
| ic.p_bv | 7% (27/404) | 71% (286/404) | 11% (17/155) | 60% (93/155) | 11% (13/114) | 59% (67/114) |
| ne.y_bv | 3% (11/404) | 83% (336/404) | 5% (7/155) | 75% (117/155) | 12% (14/114) | 59% (67/114) |
| ne.p_bv | 11% (44/404) | 72% (291/404) | 13% (20/155) | 64% (99/155) | 14% (16/114) | 63% (72/114) |
| ag.y_bv | 14% (56/404) | 45% (182/404) | 28% (44/155) | 34% (52/155) | 51% (58/114) | 21% (24/114) |
| ag.p_bv | 13% (53/404) | 62% (252/404) | 16% (25/155) | 52% (81/155) | 20% (23/114) | 50% (57/114) |
| fear.y_bv | 0% (0/404) | 100% (402/404) | 0% (0/155) | 100% (155/155) | 0% (0/114) | 99% (113/114) |
| fear.p_bv | 0% (1/404) | 96% (387/404) | 0% (0/155) | 95% (147/155) | 1% (1/114) | 92% (105/114) |
| fr.y_bv | 5% (20/404) | 69% (279/404) | 10% (16/155) | 58% (90/155) | 25% (29/114) | 32% (37/114) |
| fr.p_bv | 6% (25/404) | 69% (278/404) | 7% (11/155) | 61% (95/155) | 12% (14/114) | 49% (56/114) |
| shy.y_bv | 5% (19/404) | 41% (167/404) | 14% (21/155) | 28% (44/155) | 16% (18/114) | 28% (32/114) |
| shy.p_bv | 14% (57/404) | 33% (135/404) | 18% (28/155) | 19% (29/155) | 9% (10/114) | 35% (40/114) |

# Table S20b. Youth and Parent Report Estimates for Prevalence of Growth Despite Parent-Parent Conflict (Estimates for Univariate Model Conditioned on Initial Adversity, Change in Adversity, and Cohort)

|  | Low Adversity | | % Change >/= SESOI despite baseline Adversity | | % Change >/= SESOI despite increases in Adversity | |
| --- | --- | --- | --- | --- | --- | --- |
| Model | Decrease | Increase | Decrease | Increase | Decrease | Increase |
| ec.y_bv | 15% (55/379) | 49% (185/379) | 21% (24/117) | 40% (47/117) | 29% (34/118) | 35% (41/118) |
| ec.p_bv | 20% (76/379) | 51% (195/379) | 26% (30/117) | 32% (38/117) | 31% (36/118) | 40% (47/118) |
| ac.y_bv | 45% (169/379) | 16% (60/379) | 41% (48/117) | 17% (20/117) | 59% (70/118) | 8% (10/118) |
| ac.p_bv | 23% (88/379) | 32% (122/379) | 36% (42/117) | 21% (24/117) | 42% (49/118) | 19% (23/118) |
| at.y_bv | 18% (70/379) | 55% (208/379) | 20% (23/117) | 40% (47/117) | 29% (34/118) | 44% (52/118) |
| at.p_bv | 8% (29/379) | 50% (188/379) | 9% (11/117) | 41% (48/117) | 13% (15/118) | 51% (60/118) |
| ic.y_bv | 0% (0/379) | 98% (372/379) | 0% (0/117) | 97% (113/117) | 0% (0/118) | 92% (109/118) |
| ic.p_bv | 6% (23/379) | 73% (275/379) | 14% (16/117) | 60% (70/117) | 11% (13/118) | 58% (69/118) |
| ne.y_bv | 2% (6/379) | 82% (309/379) | 4% (5/117) | 83% (97/117) | 9% (11/118) | 68% (80/118) |
| ne.p_bv | 12% (44/379) | 72% (272/379) | 12% (14/117) | 65% (76/117) | 17% (20/118) | 65% (77/118) |
| ag.y_bv | 15% (58/379) | 40% (152/379) | 22% (26/117) | 41% (48/117) | 35% (41/118) | 19% (23/118) |
| ag.p_bv | 14% (53/379) | 62% (234/379) | 12% (14/117) | 59% (69/117) | 19% (23/118) | 55% (65/118) |
| fear.y_bv | 0% (0/379) | 99% (377/379) | 0% (0/117) | 100% (117/117) | 0% (0/118) | 98% (116/118) |
| fear.p_bv | 0% (1/379) | 95% (361/379) | 0% (0/117) | 90% (105/117) | 1% (1/118) | 94% (111/118) |
| fr.y_bv | 5% (20/379) | 67% (254/379) | 8% (9/117) | 59% (69/117) | 10% (12/118) | 53% (63/118) |
| fr.p_bv | 7% (25/379) | 68% (259/379) | 5% (6/117) | 65% (76/117) | 13% (15/118) | 59% (70/118) |
| shy.y_bv | 7% (25/379) | 37% (140/379) | 7% (8/117) | 47% (55/117) | 13% (15/118) | 35% (41/118) |
| shy.p_bv | 15% (58/379) | 33% (125/379) | 17% (20/117) | 22% (26/117) | 14% (16/118) | 34% (40/118) |

# Table S20c. Youth and Parent Report Estimates for Prevalence of Growth Despite Discrimination/Acculturation Stress(Estimates for Univariate Model Conditioned on Initial Adversity, Change in Adversity, and Cohort)

|  | Low Adversity | | % Change >/= SESOI despite baseline Adversity | | % Change >/= SESOI despite increases in Adversity | |
| --- | --- | --- | --- | --- | --- | --- |
| Model | Decrease | Increase | Decrease | Increase | Decrease | Increase |
| ec.y_bv | 18% (92/517) | 47% (243/517) | 17% (5/30) | 37% (11/30) | 28% (31/109) | 37% (40/109) |
| ec.p_bv | 20% (104/517) | 50% (257/517) | 20% (6/30) | 47% (14/30) | 28% (30/109) | 42% (46/109) |
| ac.y_bv | 44% (229/517) | 16% (84/517) | 40% (12/30) | 20% (6/30) | 60% (65/109) | 15% (16/109) |
| ac.p_bv | 25% (130/517) | 30% (154/517) | 27% (8/30) | 27% (8/30) | 37% (40/109) | 24% (26/109) |
| at.y_bv | 19% (99/517) | 53% (272/517) | 23% (7/30) | 40% (12/30) | 28% (31/109) | 44% (48/109) |
| at.p_bv | 8% (42/517) | 52% (270/517) | 13% (4/30) | 23% (7/30) | 13% (14/109) | 43% (47/109) |
| ic.y_bv | 0% (0/517) | 95% (492/517) | 0% (0/30) | 93% (28/30) | 0% (0/109) | 98% (107/109) |
| ic.p_bv | 8% (42/517) | 71% (365/517) | 7% (2/30) | 70% (21/30) | 14% (15/109) | 58% (63/109) |
| ne.y_bv | 4% (21/517) | 77% (398/517) | 3% (1/30) | 80% (24/30) | 2% (2/109) | 82% (89/109) |
| ne.p_bv | 13% (66/517) | 70% (364/517) | 10% (3/30) | 70% (21/30) | 13% (14/109) | 65% (71/109) |
| ag.y_bv | 20% (102/517) | 37% (191/517) | 20% (6/30) | 33% (10/30) | 19% (21/109) | 42% (46/109) |
| ag.p_bv | 14% (70/517) | 60% (309/517) | 10% (3/30) | 63% (19/30) | 17% (19/109) | 57% (62/109) |
| fear.y_bv | 0% (0/517) | 99% (510/517) | 0% (0/30) | 97% (29/30) | 0% (0/109) | 100% (109/109) |
| fear.p_bv | 0% (2/517) | 95% (492/517) | 0% (0/30) | 100% (30/30) | 0% (0/109) | 94% (103/109) |
| fr.y_bv | 9% (44/517) | 64% (330/517) | 13% (4/30) | 43% (13/30) | 6% (6/109) | 66% (72/109) |
| fr.p_bv | 7% (35/517) | 68% (352/517) | 13% (4/30) | 67% (20/30) | 10% (11/109) | 53% (58/109) |
| shy.y_bv | 7% (35/517) | 38% (196/517) | 7% (2/30) | 40% (12/30) | 17% (18/109) | 33% (36/109) |
| shy.p_bv | 15% (77/517) | 32% (166/517) | 13% (4/30) | 20% (6/30) | 19% (21/109) | 26% (28/109) |

# Table S21. Fit indices for domain specific trivariate regression models

| chisq | df | rmsea | tli | cfi | ModID |
| --- | --- | --- | --- | --- | --- |
| 150.618 | 30 | 0.077 | 0.894 | 0.929 | tri_PCR.adv_ec.p_anx.y |
| 164.676 | 30 | 0.081 | 0.868 | 0.912 | tri_PCR.adv_ne.p_anx.y |
| 135.903 | 30 | 0.072 | 0.918 | 0.945 | tri_PCR.adv_ec.p_avoid.y |
| 157.795 | 30 | 0.079 | 0.891 | 0.927 | tri_PCR.adv_ne.p_avoid.y |
| 296.753 | 22 | 0.135 | 0.725 | 0.832 | tri_PCR.adv_ec.p_PSS.y |
| 313.256 | 22 | 0.139 | 0.677 | 0.803 | tri_PCR.adv_ne.p_PSS.y |
| 312.097 | 22 | 0.139 | 0.679 | 0.804 | tri_PCR.adv_ec.p_ProSoc.y |
| 332.52 | 22 | 0.144 | 0.612 | 0.763 | tri_PCR.adv_ne.p_ProSoc.y |
| 268.382 | 22 | 0.128 | 0.749 | 0.847 | tri_PCR.adv_ec.p_SEQ.y |
| 296.704 | 22 | 0.135 | 0.688 | 0.809 | tri_PCR.adv_ne.p_SEQ.y |
| 106.023 | 30 | 0.061 | 0.928 | 0.952 | tri_PPR.adv_ec.p_anx.y |
| 108.097 | 30 | 0.062 | 0.915 | 0.944 | tri_PPR.adv_ne.p_anx.y |
| 111.135 | 30 | 0.063 | 0.933 | 0.955 | tri_PPR.adv_ec.p_avoid.y |
| 116.898 | 30 | 0.065 | 0.92 | 0.946 | tri_PPR.adv_ne.p_avoid.y |
| 229.885 | 22 | 0.118 | 0.77 | 0.859 | tri_PPR.adv_ec.p_PSS.y |
| 239.033 | 22 | 0.12 | 0.728 | 0.834 | tri_PPR.adv_ne.p_PSS.y |
| 323.007 | 22 | 0.142 | 0.647 | 0.784 | tri_PPR.adv_ec.p_ProSoc.y |
| 325.878 | 22 | 0.142 | 0.586 | 0.747 | tri_PPR.adv_ne.p_ProSoc.y |
| 241.008 | 22 | 0.121 | 0.76 | 0.853 | tri_PPR.adv_ec.p_SEQ.y |
| 255.232 | 22 | 0.125 | 0.708 | 0.821 | tri_PPR.adv_ne.p_SEQ.y |
| 61.977 | 30 | 0.04 | 0.968 | 0.979 | tri_Disc.adv_ec.p_anx.y |
| 75.054 | 30 | 0.047 | 0.949 | 0.966 | tri_Disc.adv_ne.p_anx.y |
| 64.753 | 30 | 0.041 | 0.97 | 0.98 | tri_Disc.adv_ec.p_avoid.y |
| 80.094 | 30 | 0.049 | 0.951 | 0.968 | tri_Disc.adv_ne.p_avoid.y |
| 194.334 | 22 | 0.107 | 0.798 | 0.876 | tri_Disc.adv_ec.p_PSS.y |
| 211.856 | 22 | 0.112 | 0.749 | 0.847 | tri_Disc.adv_ne.p_PSS.y |
| 316.613 | 22 | 0.14 | 0.639 | 0.78 | tri_Disc.adv_ec.p_ProSoc.y |
| 328.31 | 22 | 0.143 | 0.569 | 0.737 | tri_Disc.adv_ne.p_ProSoc.y |
| 209.54 | 22 | 0.112 | 0.782 | 0.867 | tri_Disc.adv_ec.p_SEQ.y |
| 232.309 | 22 | 0.118 | 0.723 | 0.831 | tri_Disc.adv_ne.p_SEQ.y |

# Table S22. Parameters from adversity domain trivariate regression models

| Parameter | Estimate | SE | PValue | StdAll | ModelName |
| --- | --- | --- | --- | --- | --- |
| S_Temperment_ON_I_Factor | 0.14 | 0.07 | 0.048 | 0.26 | tri_PCR.adv_ec.p_anx.y |
| S_Temperment_ON_S_Factor | -0.16 | 0.07 | 0.027 | -0.35 | tri_PCR.adv_ec.p_anx.y |
| S_Temperment_ON_Cohort | 0.00 | 0.03 | 0.959 | 0.00 | tri_PCR.adv_ec.p_anx.y |
| I_Temperment_ON_I_Factor | -0.29 | 0.07 | 0.000 | -0.31 | tri_PCR.adv_ec.p_anx.y |
| I_Temperment_ON_Cohort | 0.10 | 0.03 | 0.001 | 0.16 | tri_PCR.adv_ec.p_anx.y |
| S_Temperment_ON_I_Factor | 0.01 | 0.07 | 0.834 | 0.03 | tri_PCR.adv_ne.p_anx.y |
| S_Temperment_ON_S_Factor | -0.01 | 0.06 | 0.913 | -0.02 | tri_PCR.adv_ne.p_anx.y |
| S_Temperment_ON_Cohort | 0.02 | 0.03 | 0.349 | 0.08 | tri_PCR.adv_ne.p_anx.y |
| I_Temperment_ON_I_Factor | -0.28 | 0.06 | 0.000 | -0.37 | tri_PCR.adv_ne.p_anx.y |
| I_Temperment_ON_Cohort | 0.10 | 0.03 | 0.000 | 0.20 | tri_PCR.adv_ne.p_anx.y |
| S_Temperment_ON_I_Factor | 0.04 | 0.03 | 0.202 | 0.12 | tri_PCR.adv_ec.p_avoid.y |
| S_Temperment_ON_S_Factor | -0.07 | 0.03 | 0.046 | -0.22 | tri_PCR.adv_ec.p_avoid.y |
| S_Temperment_ON_Cohort | -0.04 | 0.03 | 0.085 | -0.13 | tri_PCR.adv_ec.p_avoid.y |
| I_Temperment_ON_I_Factor | -0.07 | 0.03 | 0.019 | -0.13 | tri_PCR.adv_ec.p_avoid.y |
| I_Temperment_ON_Cohort | 0.14 | 0.03 | 0.000 | 0.23 | tri_PCR.adv_ec.p_avoid.y |
| S_Temperment_ON_I_Factor | 0.02 | 0.03 | 0.426 | 0.08 | tri_PCR.adv_ne.p_avoid.y |
| S_Temperment_ON_S_Factor | -0.07 | 0.03 | 0.034 | -0.24 | tri_PCR.adv_ne.p_avoid.y |
| S_Temperment_ON_Cohort | 0.02 | 0.02 | 0.503 | 0.05 | tri_PCR.adv_ne.p_avoid.y |
| I_Temperment_ON_I_Factor | -0.04 | 0.03 | 0.133 | -0.09 | tri_PCR.adv_ne.p_avoid.y |
| I_Temperment_ON_Cohort | 0.14 | 0.02 | 0.000 | 0.26 | tri_PCR.adv_ne.p_avoid.y |
| S_Temperment_ON_I_Factor | -0.02 | 0.15 | 0.893 | -0.01 | tri_PCR.adv_ec.p_PSS.y |
| S_Temperment_ON_S_Factor | 0.08 | 0.13 | 0.536 | 0.05 | tri_PCR.adv_ec.p_PSS.y |
| S_Temperment_ON_Cohort | -0.04 | 0.03 | 0.147 | -0.10 | tri_PCR.adv_ec.p_PSS.y |
| I_Temperment_ON_I_Factor | 0.33 | 0.15 | 0.029 | 0.10 | tri_PCR.adv_ec.p_PSS.y |
| I_Temperment_ON_Cohort | 0.13 | 0.03 | 0.000 | 0.21 | tri_PCR.adv_ec.p_PSS.y |
| S_Temperment_ON_I_Factor | -0.34 | 0.14 | 0.014 | -0.19 | tri_PCR.adv_ne.p_PSS.y |
| S_Temperment_ON_S_Factor | 0.09 | 0.13 | 0.481 | 0.05 | tri_PCR.adv_ne.p_PSS.y |
| S_Temperment_ON_Cohort | 0.02 | 0.02 | 0.376 | 0.06 | tri_PCR.adv_ne.p_PSS.y |
| I_Temperment_ON_I_Factor | 0.49 | 0.13 | 0.000 | 0.18 | tri_PCR.adv_ne.p_PSS.y |
| I_Temperment_ON_Cohort | 0.13 | 0.02 | 0.000 | 0.25 | tri_PCR.adv_ne.p_PSS.y |
| S_Temperment_ON_I_Factor | 0.00 | 0.02 | 0.981 | 0.00 | tri_PCR.adv_ec.p_ProSoc.y |
| S_Temperment_ON_S_Factor | 0.00 | 0.02 | 0.973 | 0.00 | tri_PCR.adv_ec.p_ProSoc.y |
| S_Temperment_ON_Cohort | -0.04 | 0.03 | 0.152 | -0.10 | tri_PCR.adv_ec.p_ProSoc.y |
| I_Temperment_ON_I_Factor | 0.03 | 0.02 | 0.273 | 0.05 | tri_PCR.adv_ec.p_ProSoc.y |
| I_Temperment_ON_Cohort | 0.13 | 0.03 | 0.000 | 0.20 | tri_PCR.adv_ec.p_ProSoc.y |
| S_Temperment_ON_I_Factor | 0.01 | 0.02 | 0.595 | 0.05 | tri_PCR.adv_ne.p_ProSoc.y |
| S_Temperment_ON_S_Factor | -0.01 | 0.02 | 0.685 | -0.04 | tri_PCR.adv_ne.p_ProSoc.y |
| S_Temperment_ON_Cohort | 0.02 | 0.02 | 0.370 | 0.06 | tri_PCR.adv_ne.p_ProSoc.y |
| I_Temperment_ON_I_Factor | 0.02 | 0.02 | 0.254 | 0.05 | tri_PCR.adv_ne.p_ProSoc.y |
| I_Temperment_ON_Cohort | 0.13 | 0.02 | 0.000 | 0.25 | tri_PCR.adv_ne.p_ProSoc.y |
| S_Temperment_ON_I_Factor | -0.11 | 0.05 | 0.017 | -0.18 | tri_PCR.adv_ec.p_SEQ.y |
| S_Temperment_ON_S_Factor | -0.10 | 0.04 | 0.010 | -0.20 | tri_PCR.adv_ec.p_SEQ.y |
| S_Temperment_ON_Cohort | -0.03 | 0.03 | 0.259 | -0.08 | tri_PCR.adv_ec.p_SEQ.y |
| I_Temperment_ON_I_Factor | -0.10 | 0.04 | 0.018 | -0.10 | tri_PCR.adv_ec.p_SEQ.y |
| I_Temperment_ON_Cohort | 0.12 | 0.03 | 0.000 | 0.20 | tri_PCR.adv_ec.p_SEQ.y |
| S_Temperment_ON_I_Factor | -0.09 | 0.04 | 0.048 | -0.16 | tri_PCR.adv_ne.p_SEQ.y |
| S_Temperment_ON_S_Factor | -0.10 | 0.04 | 0.010 | -0.20 | tri_PCR.adv_ne.p_SEQ.y |
| S_Temperment_ON_Cohort | 0.03 | 0.02 | 0.208 | 0.09 | tri_PCR.adv_ne.p_SEQ.y |
| I_Temperment_ON_I_Factor | -0.08 | 0.04 | 0.038 | -0.09 | tri_PCR.adv_ne.p_SEQ.y |
| I_Temperment_ON_Cohort | 0.13 | 0.02 | 0.000 | 0.25 | tri_PCR.adv_ne.p_SEQ.y |
| S_Temperment_ON_I_Factor | 0.17 | 0.07 | 0.013 | 0.31 | tri_PPR.adv_ec.p_anx.y |
| S_Temperment_ON_S_Factor | -0.18 | 0.07 | 0.012 | -0.39 | tri_PPR.adv_ec.p_anx.y |
| S_Temperment_ON_Cohort | 0.01 | 0.03 | 0.769 | 0.02 | tri_PPR.adv_ec.p_anx.y |
| I_Temperment_ON_I_Factor | -0.37 | 0.07 | 0.000 | -0.39 | tri_PPR.adv_ec.p_anx.y |
| I_Temperment_ON_Cohort | 0.06 | 0.03 | 0.039 | 0.10 | tri_PPR.adv_ec.p_anx.y |
| S_Temperment_ON_I_Factor | 0.01 | 0.06 | 0.818 | 0.03 | tri_PPR.adv_ne.p_anx.y |
| S_Temperment_ON_S_Factor | -0.03 | 0.06 | 0.656 | -0.06 | tri_PPR.adv_ne.p_anx.y |
| S_Temperment_ON_Cohort | 0.02 | 0.03 | 0.413 | 0.06 | tri_PPR.adv_ne.p_anx.y |
| I_Temperment_ON_I_Factor | -0.34 | 0.07 | 0.000 | -0.43 | tri_PPR.adv_ne.p_anx.y |
| I_Temperment_ON_Cohort | 0.07 | 0.03 | 0.004 | 0.14 | tri_PPR.adv_ne.p_anx.y |
| S_Temperment_ON_I_Factor | 0.05 | 0.03 | 0.082 | 0.17 | tri_PPR.adv_ec.p_avoid.y |
| S_Temperment_ON_S_Factor | -0.08 | 0.03 | 0.025 | -0.26 | tri_PPR.adv_ec.p_avoid.y |
| S_Temperment_ON_Cohort | -0.04 | 0.02 | 0.113 | -0.12 | tri_PPR.adv_ec.p_avoid.y |
| I_Temperment_ON_I_Factor | -0.11 | 0.03 | 0.000 | -0.20 | tri_PPR.adv_ec.p_avoid.y |
| I_Temperment_ON_Cohort | 0.11 | 0.03 | 0.000 | 0.18 | tri_PPR.adv_ec.p_avoid.y |
| S_Temperment_ON_I_Factor | 0.03 | 0.03 | 0.366 | 0.08 | tri_PPR.adv_ne.p_avoid.y |
| S_Temperment_ON_S_Factor | -0.08 | 0.03 | 0.012 | -0.27 | tri_PPR.adv_ne.p_avoid.y |
| S_Temperment_ON_Cohort | 0.01 | 0.02 | 0.651 | 0.03 | tri_PPR.adv_ne.p_avoid.y |
| I_Temperment_ON_I_Factor | -0.07 | 0.03 | 0.010 | -0.15 | tri_PPR.adv_ne.p_avoid.y |
| I_Temperment_ON_Cohort | 0.11 | 0.02 | 0.000 | 0.22 | tri_PPR.adv_ne.p_avoid.y |
| S_Temperment_ON_I_Factor | -0.03 | 0.14 | 0.826 | -0.02 | tri_PPR.adv_ec.p_PSS.y |
| S_Temperment_ON_S_Factor | 0.10 | 0.13 | 0.441 | 0.06 | tri_PPR.adv_ec.p_PSS.y |
| S_Temperment_ON_Cohort | -0.03 | 0.02 | 0.234 | -0.08 | tri_PPR.adv_ec.p_PSS.y |
| I_Temperment_ON_I_Factor | 0.46 | 0.15 | 0.003 | 0.14 | tri_PPR.adv_ec.p_PSS.y |
| I_Temperment_ON_Cohort | 0.09 | 0.03 | 0.001 | 0.15 | tri_PPR.adv_ec.p_PSS.y |
| S_Temperment_ON_I_Factor | -0.26 | 0.13 | 0.055 | -0.14 | tri_PPR.adv_ne.p_PSS.y |
| S_Temperment_ON_S_Factor | 0.19 | 0.12 | 0.126 | 0.11 | tri_PPR.adv_ne.p_PSS.y |
| S_Temperment_ON_Cohort | 0.01 | 0.02 | 0.559 | 0.04 | tri_PPR.adv_ne.p_PSS.y |
| I_Temperment_ON_I_Factor | 0.57 | 0.13 | 0.000 | 0.21 | tri_PPR.adv_ne.p_PSS.y |
| I_Temperment_ON_Cohort | 0.10 | 0.02 | 0.000 | 0.20 | tri_PPR.adv_ne.p_PSS.y |
| S_Temperment_ON_I_Factor | 0.00 | 0.02 | 0.924 | 0.01 | tri_PPR.adv_ec.p_ProSoc.y |
| S_Temperment_ON_S_Factor | 0.00 | 0.02 | 0.941 | 0.01 | tri_PPR.adv_ec.p_ProSoc.y |
| S_Temperment_ON_Cohort | -0.03 | 0.02 | 0.239 | -0.08 | tri_PPR.adv_ec.p_ProSoc.y |
| I_Temperment_ON_I_Factor | 0.03 | 0.02 | 0.164 | 0.07 | tri_PPR.adv_ec.p_ProSoc.y |
| I_Temperment_ON_Cohort | 0.08 | 0.03 | 0.001 | 0.14 | tri_PPR.adv_ec.p_ProSoc.y |
| S_Temperment_ON_I_Factor | 0.02 | 0.02 | 0.487 | 0.06 | tri_PPR.adv_ne.p_ProSoc.y |
| S_Temperment_ON_S_Factor | 0.00 | 0.02 | 0.852 | -0.02 | tri_PPR.adv_ne.p_ProSoc.y |
| S_Temperment_ON_Cohort | 0.02 | 0.02 | 0.486 | 0.05 | tri_PPR.adv_ne.p_ProSoc.y |
| I_Temperment_ON_I_Factor | 0.03 | 0.02 | 0.167 | 0.07 | tri_PPR.adv_ne.p_ProSoc.y |
| I_Temperment_ON_Cohort | 0.10 | 0.02 | 0.000 | 0.18 | tri_PPR.adv_ne.p_ProSoc.y |
| S_Temperment_ON_I_Factor | -0.09 | 0.04 | 0.048 | -0.16 | tri_PPR.adv_ec.p_SEQ.y |
| S_Temperment_ON_S_Factor | -0.10 | 0.04 | 0.009 | -0.21 | tri_PPR.adv_ec.p_SEQ.y |
| S_Temperment_ON_Cohort | -0.02 | 0.02 | 0.485 | -0.05 | tri_PPR.adv_ec.p_SEQ.y |
| I_Temperment_ON_I_Factor | -0.15 | 0.04 | 0.000 | -0.15 | tri_PPR.adv_ec.p_SEQ.y |
| I_Temperment_ON_Cohort | 0.09 | 0.03 | 0.001 | 0.14 | tri_PPR.adv_ec.p_SEQ.y |
| S_Temperment_ON_I_Factor | -0.10 | 0.04 | 0.024 | -0.17 | tri_PPR.adv_ne.p_SEQ.y |
| S_Temperment_ON_S_Factor | -0.11 | 0.04 | 0.004 | -0.22 | tri_PPR.adv_ne.p_SEQ.y |
| S_Temperment_ON_Cohort | 0.03 | 0.02 | 0.211 | 0.09 | tri_PPR.adv_ne.p_SEQ.y |
| I_Temperment_ON_I_Factor | -0.12 | 0.04 | 0.002 | -0.14 | tri_PPR.adv_ne.p_SEQ.y |
| I_Temperment_ON_Cohort | 0.10 | 0.02 | 0.000 | 0.19 | tri_PPR.adv_ne.p_SEQ.y |
| S_Temperment_ON_I_Factor | 0.14 | 0.07 | 0.039 | 0.25 | tri_Disc.adv_ec.p_anx.y |
| S_Temperment_ON_S_Factor | -0.17 | 0.07 | 0.017 | -0.37 | tri_Disc.adv_ec.p_anx.y |
| S_Temperment_ON_Cohort | 0.00 | 0.04 | 0.951 | -0.01 | tri_Disc.adv_ec.p_anx.y |
| I_Temperment_ON_I_Factor | -0.37 | 0.07 | 0.000 | -0.40 | tri_Disc.adv_ec.p_anx.y |
| I_Temperment_ON_Cohort | 0.06 | 0.04 | 0.120 | 0.09 | tri_Disc.adv_ec.p_anx.y |
| S_Temperment_ON_I_Factor | -0.01 | 0.06 | 0.906 | -0.01 | tri_Disc.adv_ne.p_anx.y |
| S_Temperment_ON_S_Factor | -0.01 | 0.06 | 0.798 | -0.03 | tri_Disc.adv_ne.p_anx.y |
| S_Temperment_ON_Cohort | -0.03 | 0.04 | 0.519 | -0.08 | tri_Disc.adv_ne.p_anx.y |
| I_Temperment_ON_I_Factor | -0.34 | 0.06 | 0.000 | -0.44 | tri_Disc.adv_ne.p_anx.y |
| I_Temperment_ON_Cohort | 0.10 | 0.03 | 0.005 | 0.18 | tri_Disc.adv_ne.p_anx.y |
| S_Temperment_ON_I_Factor | 0.04 | 0.03 | 0.126 | 0.14 | tri_Disc.adv_ec.p_avoid.y |
| S_Temperment_ON_S_Factor | -0.08 | 0.03 | 0.018 | -0.26 | tri_Disc.adv_ec.p_avoid.y |
| S_Temperment_ON_Cohort | -0.05 | 0.04 | 0.229 | -0.13 | tri_Disc.adv_ec.p_avoid.y |
| I_Temperment_ON_I_Factor | -0.11 | 0.03 | 0.000 | -0.21 | tri_Disc.adv_ec.p_avoid.y |
| I_Temperment_ON_Cohort | 0.12 | 0.04 | 0.001 | 0.19 | tri_Disc.adv_ec.p_avoid.y |
| S_Temperment_ON_I_Factor | 0.02 | 0.03 | 0.371 | 0.08 | tri_Disc.adv_ne.p_avoid.y |
| S_Temperment_ON_S_Factor | -0.08 | 0.03 | 0.013 | -0.26 | tri_Disc.adv_ne.p_avoid.y |
| S_Temperment_ON_Cohort | -0.03 | 0.04 | 0.467 | -0.08 | tri_Disc.adv_ne.p_avoid.y |
| I_Temperment_ON_I_Factor | -0.07 | 0.03 | 0.005 | -0.16 | tri_Disc.adv_ne.p_avoid.y |
| I_Temperment_ON_Cohort | 0.14 | 0.03 | 0.000 | 0.27 | tri_Disc.adv_ne.p_avoid.y |
| S_Temperment_ON_I_Factor | 0.02 | 0.14 | 0.873 | 0.01 | tri_Disc.adv_ec.p_PSS.y |
| S_Temperment_ON_S_Factor | 0.15 | 0.13 | 0.242 | 0.09 | tri_Disc.adv_ec.p_PSS.y |
| S_Temperment_ON_Cohort | -0.04 | 0.04 | 0.325 | -0.11 | tri_Disc.adv_ec.p_PSS.y |
| I_Temperment_ON_I_Factor | 0.49 | 0.15 | 0.001 | 0.15 | tri_Disc.adv_ec.p_PSS.y |
| I_Temperment_ON_Cohort | 0.10 | 0.04 | 0.008 | 0.15 | tri_Disc.adv_ec.p_PSS.y |
| S_Temperment_ON_I_Factor | -0.25 | 0.13 | 0.062 | -0.13 | tri_Disc.adv_ne.p_PSS.y |
| S_Temperment_ON_S_Factor | 0.19 | 0.12 | 0.113 | 0.11 | tri_Disc.adv_ne.p_PSS.y |
| S_Temperment_ON_Cohort | -0.03 | 0.04 | 0.442 | -0.08 | tri_Disc.adv_ne.p_PSS.y |
| I_Temperment_ON_I_Factor | 0.59 | 0.13 | 0.000 | 0.22 | tri_Disc.adv_ne.p_PSS.y |
| I_Temperment_ON_Cohort | 0.13 | 0.03 | 0.000 | 0.24 | tri_Disc.adv_ne.p_PSS.y |
| S_Temperment_ON_I_Factor | 0.00 | 0.02 | 0.937 | -0.01 | tri_Disc.adv_ec.p_ProSoc.y |
| S_Temperment_ON_S_Factor | 0.00 | 0.02 | 0.921 | -0.01 | tri_Disc.adv_ec.p_ProSoc.y |
| S_Temperment_ON_Cohort | -0.04 | 0.04 | 0.302 | -0.11 | tri_Disc.adv_ec.p_ProSoc.y |
| I_Temperment_ON_I_Factor | 0.03 | 0.02 | 0.171 | 0.07 | tri_Disc.adv_ec.p_ProSoc.y |
| I_Temperment_ON_Cohort | 0.09 | 0.04 | 0.016 | 0.14 | tri_Disc.adv_ec.p_ProSoc.y |
| S_Temperment_ON_I_Factor | 0.01 | 0.02 | 0.556 | 0.05 | tri_Disc.adv_ne.p_ProSoc.y |
| S_Temperment_ON_S_Factor | -0.01 | 0.02 | 0.762 | -0.02 | tri_Disc.adv_ne.p_ProSoc.y |
| S_Temperment_ON_Cohort | -0.03 | 0.04 | 0.459 | -0.08 | tri_Disc.adv_ne.p_ProSoc.y |
| I_Temperment_ON_I_Factor | 0.03 | 0.02 | 0.188 | 0.06 | tri_Disc.adv_ne.p_ProSoc.y |
| I_Temperment_ON_Cohort | 0.12 | 0.03 | 0.000 | 0.23 | tri_Disc.adv_ne.p_ProSoc.y |
| S_Temperment_ON_I_Factor | -0.10 | 0.04 | 0.020 | -0.18 | tri_Disc.adv_ec.p_SEQ.y |
| S_Temperment_ON_S_Factor | -0.11 | 0.04 | 0.004 | -0.22 | tri_Disc.adv_ec.p_SEQ.y |
| S_Temperment_ON_Cohort | -0.03 | 0.04 | 0.492 | -0.08 | tri_Disc.adv_ec.p_SEQ.y |
| I_Temperment_ON_I_Factor | -0.16 | 0.04 | 0.000 | -0.16 | tri_Disc.adv_ec.p_SEQ.y |
| I_Temperment_ON_Cohort | 0.08 | 0.04 | 0.018 | 0.14 | tri_Disc.adv_ec.p_SEQ.y |
| S_Temperment_ON_I_Factor | -0.10 | 0.04 | 0.016 | -0.18 | tri_Disc.adv_ne.p_SEQ.y |
| S_Temperment_ON_S_Factor | -0.11 | 0.04 | 0.003 | -0.22 | tri_Disc.adv_ne.p_SEQ.y |
| S_Temperment_ON_Cohort | -0.02 | 0.04 | 0.691 | -0.05 | tri_Disc.adv_ne.p_SEQ.y |
| I_Temperment_ON_I_Factor | -0.12 | 0.04 | 0.001 | -0.14 | tri_Disc.adv_ne.p_SEQ.y |
| I_Temperment_ON_Cohort | 0.12 | 0.03 | 0.000 | 0.23 | tri_Disc.adv_ne.p_SEQ.y |

# Table S23a. Demographic differences between youth who grew or did not grow in youth-reported effortful control following adversity

| Dependent Variable | *No Growth*  *M*/% | *Growth*  *M*/% | *t/z* | *df* | *p* | *d/h* |
| --- | --- | --- | --- | --- | --- | --- |
| Child Age | 12.21 | 12.55 | -0.83 | 92.87 | .411 | -0.14 |
| Child Grade | 6.23 | 6.61 | -0.87 | 86.32 | .385 | -0.16 |
| Child Gender | 0.59 | 0.51 | 0.89 | 1 | 0.375 | 0.15 |
| Child Ethnicity | 0.17 | 0.14 | 0.47 | 1 | 0.637 | 0.08 |
| Child Race | 0.32 | 0.22 | 1.18 | 1 | 0.237 | 0.21 |
| Parent Marital Status | 0.67 | 0.65 | 0.25 | 1 | 0.806 | 0.04 |
| Parent Education | 5.58 | 5.12 | **2.08** | **93.01** | **.041** | **0.36** |
| Other Parent Education | 4.81 | 5.04 | -0.85 | 96.96 | .397 | -0.15 |
| School Lunch (PR) | 0.29 | 0.22 | 0.83 | 1 | 0.405 | 0.15 |
| Food Stamps (PR) | 0.18 | 0.10 | 1.30 | 1 | 0.193 | 0.24 |
| Total Income (PR) | 85,324.05 | 70,835.62 | 1.65 | 127.59 | .102 | 0.26 |
| Parent Gender | 0.89 | 0.86 | 0.64 | 1 | 0.520 | 0.11 |
| Parent Ethnicity | 0.14 | 0.14 | 0.02 | 1 | 0.982 | 0.00 |
| Parent Race | 0.26 | 0.22 | 0.47 | 1 | 0.639 | 0.08 |

# Table S23b. Demographic differences between youth who grew or did not grow in parent-reported effortful control following adversity

| Dependent Variable | *No Growth*  *M*/% | *Growth*  *M*/% | *t/z* | *df* | *p* | *d/h* |
| --- | --- | --- | --- | --- | --- | --- |
| Child Age | 12.43 | 12.19 | 0.62 | 141.35 | .538 | 0.10 |
| Child Grade | 6.39 | 6.31 | 0.20 | 138.24 | .843 | 0.03 |
| Child Gender | 0.54 | 0.59 | -0.58 | 1 | 0.560 | -0.09 |
| Child Ethnicity | 0.18 | 0.15 | 0.49 | 1 | 0.625 | 0.08 |
| Child Race | 0.28 | 0.29 | -0.16 | 1 | 0.873 | -0.03 |
| Parent Marital Status | 0.72 | 0.60 | 1.50 | 1 | 0.135 | 0.24 |
| Parent Education | 5.40 | 5.47 | -0.35 | 150.79 | .729 | -0.06 |
| Other Parent Education | 4.94 | 4.81 | 0.51 | 138.89 | .611 | 0.08 |
| School Lunch (PR) | 0.29 | 0.24 | 0.82 | 1 | 0.414 | 0.13 |
| Food Stamps (PR) | 0.19 | 0.12 | 1.16 | 1 | 0.248 | 0.19 |
| Total Income (PR) | 88,288.80 | 71,198.88 | 1.85 | 142.99 | .066 | 0.30 |
| Parent Gender | 0.87 | 0.90 | -0.53 | 1 | 0.595 | -0.09 |
| Parent Ethnicity | 0.15 | 0.13 | 0.36 | 1 | 0.718 | 0.06 |
| Parent Race | 0.25 | 0.25 | -0.04 | 1 | 0.967 | -0.01 |

# Table S23c. Demographic differences between youth who grew or did not grow in youth-reported emotional stability following adversity

| Dependent Variable | *No Growth*  *M*/% | *Growth*  *M*/% | *t/z* | *df* | *p* | *d/h* |
| --- | --- | --- | --- | --- | --- | --- |
| Child Age | 12.01 | 12.48 | -1.12 | 96.48 | .265 | -0.20 |
| Child Grade | 6.06 | 6.50 | -1.07 | 100.10 | .289 | -0.18 |
| Child Gender | 0.58 | 0.55 | 0.27 | 1 | 0.791 | 0.05 |
| Child Ethnicity | 0.10 | 0.20 | -1.61 | 1 | 0.106 | -0.29 |
| Child Race | 0.25 | 0.31 | -0.74 | 1 | 0.461 | -0.13 |
| Parent Marital Status | 0.63 | 0.68 | -0.60 | 1 | 0.546 | -0.10 |
| Parent Education | 5.67 | 5.31 | 1.70 | 103.04 | .093 | 0.29 |
| Other Parent Education | 5.06 | 4.79 | 0.96 | 101.17 | .337 | 0.17 |
| School Lunch (PR) | 0.33 | 0.24 | 1.18 | 1 | 0.237 | 0.20 |
| Food Stamps (PR) | 0.16 | 0.16 | -0.02 | 1 | 0.980 | 0.00 |
| Total Income (PR) | 85,214.29 | 78,286.78 | 0.65 | 82.22 | .517 | 0.12 |
| Parent Gender | 0.87 | 0.89 | -0.45 | 1 | 0.656 | -0.08 |
| Parent Ethnicity | 0.10 | 0.17 | -1.20 | 1 | 0.228 | -0.21 |
| Parent Race | 0.21 | 0.27 | -0.76 | 1 | 0.449 | -0.13 |

# Table S23d. Demographic differences between youth who grew or did not grow in parent-reported emotional stability following adversity

| Dependent Variable | *No Growth*  *M*/% | *Growth*  *M*/% | *t/z* | *df* | *p* | *d/h* |
| --- | --- | --- | --- | --- | --- | --- |
| Child Age | 11.70 | 12.73 | **-2.69** | **129.80** | **.008** | **-0.44** |
| Child Grade | 5.60 | 6.84 | **-3.22** | **133.53** | **.002** | **-0.52** |
| Child Gender | 0.60 | 0.54 | 0.76 | 1 | 0.448 | 0.13 |
| Child Ethnicity | 0.17 | 0.16 | 0.09 | 1 | 0.930 | 0.01 |
| Child Race | 0.30 | 0.28 | 0.27 | 1 | 0.785 | 0.05 |
| Parent Marital Status | 0.62 | 0.70 | -1.05 | 1 | 0.292 | -0.17 |
| Parent Education | 5.45 | 5.42 | 0.14 | 117.62 | .887 | 0.02 |
| Other Parent Education | 4.68 | 5.01 | -1.25 | 125.13 | .214 | -0.21 |
| School Lunch (PR) | 0.32 | 0.24 | 1.09 | 1 | 0.275 | 0.18 |
| Food Stamps (PR) | 0.20 | 0.13 | 1.15 | 1 | 0.250 | 0.19 |
| Total Income (PR) | 78,544.51 | 81,977.20 | -0.35 | 114.92 | .728 | -0.06 |
| Parent Gender | 0.86 | 0.89 | -0.52 | 1 | 0.602 | -0.09 |
| Parent Ethnicity | 0.15 | 0.14 | 0.18 | 1 | 0.860 | 0.03 |
| Parent Race | 0.27 | 0.24 | 0.42 | 1 | 0.674 | 0.07 |

# Table S24a. Demographic differences between youth who grew in youth-reported effortful control despite adversity or without adversity

| Dependent Variable | *Growth*  *Without*  *Adversity*  *M*/% | *Growth*  *Despite*  *Adversity*  *M*/% | *t/z* | *df* | *p* | *d/h* |
| --- | --- | --- | --- | --- | --- | --- |
| Child Age | 11.37 | 12.55 | **-3.11** | **71.34** | **.003** | **-0.48** |
| Child Grade | 5.63 | 6.61 | **-2.43** | **67.01** | **.018** | **-0.40** |
| Child Gender | 0.56 | 0.51 | 0.68 | 1 | 0.494 | 0.11 |
| Child Ethnicity | 0.13 | 0.14 | -0.30 | 1 | 0.765 | -0.05 |
| Child Race | 0.32 | 0.22 | 1.29 | 1 | 0.195 | 0.21 |
| Parent Marital Status | 0.83 | 0.65 | **2.90** | **1** | **0.004** | **0.42** |
| Parent Education | 5.60 | 5.12 | **2.42** | **68.64** | **.018** | **0.39** |
| Other Parent Education | 5.31 | 5.04 | 1.11 | 68.59 | .270 | 0.17 |
| School Lunch (PR) | 0.15 | 0.22 | -1.31 | 1 | 0.192 | -0.19 |
| Food Stamps (PR) | 0.04 | 0.10 | -1.68 | 1 | 0.092 | -0.23 |
| Total Income (PR) | 103,135.51 | 70,835.62 | **3.85** | **139.68** | **< .001** | **0.41** |
| Parent Gender | 0.92 | 0.86 | 1.47 | 1 | 0.142 | 0.21 |
| Parent Ethnicity | 0.10 | 0.14 | -0.93 | 1 | 0.351 | -0.14 |
| Parent Race | 0.26 | 0.22 | 0.57 | 1 | 0.566 | 0.09 |

# Table S24b. Demographic differences between youth who grew in parent-reported effortful control despite adversity or without adversity

| Dependent Variable | *Growth*  *Without*  *Adversity*  *M*/% | *Growth*  *Despite*  *Adversity*  *M*/% | *t/z* | *df* | *p* | *d/h* |
| --- | --- | --- | --- | --- | --- | --- |
| Child Age | 11.39 | 12.19 | **-2.40** | **106.98** | **.018** | **-0.33** |
| Child Grade | 5.70 | 6.31 | -1.76 | 104.10 | .081 | -0.25 |
| Child Gender | 0.59 | 0.59 | 0.03 | 1 | 0.980 | 0.00 |
| Child Ethnicity | 0.15 | 0.15 | 0.07 | 1 | 0.942 | 0.01 |
| Child Race | 0.30 | 0.29 | 0.05 | 1 | 0.962 | 0.01 |
| Parent Marital Status | 0.79 | 0.60 | **3.22** | **1** | **0.001** | **0.42** |
| Parent Education | 5.68 | 5.47 | 1.27 | 123.20 | .207 | 0.16 |
| Other Parent Education | 5.27 | 4.81 | **2.03** | **106.12** | **.044** | **0.28** |
| School Lunch (PR) | 0.17 | 0.24 | -1.26 | 1 | 0.206 | -0.17 |
| Food Stamps (PR) | 0.05 | 0.12 | -1.84 | 1 | 0.066 | -0.23 |
| Total Income (PR) | 102,168.24 | 71,198.88 | **3.91** | **156.07** | **< .001** | **0.45** |
| Parent Gender | 0.92 | 0.90 | 0.70 | 1 | 0.487 | 0.09 |
| Parent Ethnicity | 0.11 | 0.13 | -0.63 | 1 | 0.527 | -0.08 |
| Parent Race | 0.26 | 0.25 | 0.24 | 1 | 0.808 | 0.03 |

# Table S24c. Demographic differences between youth who grew in youth-reported emotional stability despite adversity or without adversity

| Dependent Variable | *Growth*  *Without*  *Adversity*  *M*/% | *Growth*  *Despite*  *Adversity*  *M*/% | *t/z* | *df* | *p* | *d/h* |
| --- | --- | --- | --- | --- | --- | --- |
| Child Age | 11.58 | 12.48 | **-3.48** | **159.22** | **.001** | **-0.38** |
| Child Grade | 5.85 | 6.50 | **-2.45** | **154.14** | **.015** | **-0.27** |
| Child Gender | 0.55 | 0.55 | -0.05 | 1 | 0.959 | -0.01 |
| Child Ethnicity | 0.12 | 0.20 | **-2.02** | **1** | **0.044** | **-0.21** |
| Child Race | 0.34 | 0.31 | 0.58 | 1 | 0.560 | 0.07 |
| Parent Marital Status | 0.81 | 0.68 | **2.85** | **1** | **0.004** | **0.30** |
| Parent Education | 5.70 | 5.31 | **2.86** | **148.37** | **.005** | **0.33** |
| Other Parent Education | 5.29 | 4.79 | **2.77** | **155.59** | **.006** | **0.31** |
| School Lunch (PR) | 0.15 | 0.24 | **-2.13** | **1** | **0.033** | **-0.23** |
| Food Stamps (PR) | 0.04 | 0.16 | **-4.30** | **1** | **< .001** | **-0.41** |
| Total Income (PR) | 107,261.56 | 78,286.78 | **4.01** | **260.77** | **< .001** | **0.34** |
| Parent Gender | 0.93 | 0.89 | 1.37 | 1 | 0.171 | 0.14 |
| Parent Ethnicity | 0.10 | 0.17 | -1.89 | 1 | 0.058 | -0.20 |
| Parent Race | 0.28 | 0.27 | 0.31 | 1 | 0.757 | 0.03 |

# Table S24d. Demographic differences between youth who grew in parent-reported emotional stability despite adversity or without adversity

| Dependent Variable | *Growth*  *Without*  *Adversity*  *M*/% | *Growth*  *Despite*  *Adversity*  *M*/% | *t/z* | *df* | *p* | *d/h* |
| --- | --- | --- | --- | --- | --- | --- |
| Child Age | 11.63 | 12.73 | **-3.99** | **143.52** | **< .001** | **-0.47** |
| Child Grade | 5.94 | 6.84 | **-3.19** | **140.11** | **.002** | **-0.38** |
| Child Gender | 0.55 | 0.54 | 0.25 | 1 | 0.799 | 0.03 |
| Child Ethnicity | 0.12 | 0.16 | -0.93 | 1 | 0.353 | -0.10 |
| Child Race | 0.32 | 0.28 | 0.75 | 1 | 0.453 | 0.09 |
| Parent Marital Status | 0.80 | 0.70 | **2.12** | **1** | **0.034** | **0.24** |
| Parent Education | 5.68 | 5.42 | 1.82 | 140.90 | .071 | 0.22 |
| Other Parent Education | 5.19 | 5.01 | 0.95 | 144.11 | .343 | 0.11 |
| School Lunch (PR) | 0.16 | 0.24 | -1.69 | 1 | 0.092 | -0.19 |
| Food Stamps (PR) | 0.04 | 0.13 | **-3.30** | **1** | **0.001** | **-0.34** |
| Total Income (PR) | 103,332.39 | 81,977.20 | **2.84** | **203.07** | **.005** | **0.27** |
| Parent Gender | 0.93 | 0.89 | 1.24 | 1 | 0.216 | 0.14 |
| Parent Ethnicity | 0.10 | 0.14 | -1.12 | 1 | 0.265 | -0.12 |
| Parent Race | 0.28 | 0.24 | 0.76 | 1 | 0.450 | 0.09 |

# Table S25a. Regression of Univariate LGC Effortful Control and Emotional Stability Intercepts and Slopes on Gender (0 = Male, 1 = Female)

| Parameter | $b$ | SE | *p* | $\beta$ |
| --- | --- | --- | --- | --- |
| Youth-Report EC Intercept | 0.06 | 0.04 | 0.132 | 0.07 |
| Youth-Report EC Slope | 0.03 | 0.05 | 0.588 | 0.03 |
| **Parent-Report EC Intercept** | **0.20** | **0.04** | **0.000** | **0.19** |
| Parent-Report EC Slope | 0.06 | 0.04 | 0.107 | 0.08 |
| **Youth-Report ES Intercept** | **-0.12** | **0.04** | **0.003** | **-0.15** |
| Youth-Report ES Slope | -0.04 | 0.05 | 0.419 | -0.05 |
| Parent-Report ES Intercept | -0.04 | 0.04 | 0.326 | -0.04 |
| Parent-Report ES Slope | -0.01 | 0.04 | 0.733 | -0.02 |

# Table S25b. Regression of Bivariate LGC Effortful Control and Emotional Stability Intercepts and Slopes on Gender (0 = Male, 1 = Female)

| Parameter | $b$ | SE | *p* | $\beta$ |
| --- | --- | --- | --- | --- |
| Youth-Report EC Intercept | 0.06 | 0.04 | 0.128 | 0.07 |
| Youth-Report EC Slope | 0.02 | 0.05 | 0.638 | 0.03 |
| **Parent-Report EC Intercept** | **0.20** | **0.04** | **0.000** | **0.19** |
| Parent-Report EC Slope | 0.06 | 0.04 | 0.124 | 0.08 |
| **Youth-Report ES Intercept** | **-0.12** | **0.04** | **0.003** | **-0.15** |
| Youth-Report ES Slope | -0.04 | 0.05 | 0.380 | -0.05 |
| Parent-Report ES Intercept | -0.04 | 0.04 | 0.338 | -0.04 |
| Parent-Report ES Slope | -0.01 | 0.04 | 0.707 | -0.02 |

# Table S25c. Unstandardized Slopes for Univariate Latent Growth Curve Models of Youth and Parent-Reported Effortful Control and Emotional Stability Conditioned on Cohort and Gender

| Construct | Parameter | *b* | *SE* | *p* |
| --- | --- | --- | --- | --- |
| Effortful Control Youth-Report | Slope Intercept | 0.11 | 0.05 | 0.022 |
|  | Slope Variance | 0.15 | 0.04 | 0.000 |
| Effortful Control Parent-Report | Slope Intercept | 0.11 | 0.04 | 0.003 |
|  | Slope Variance | 0.15 | 0.03 | 0.000 |
| Emotional Stability Youth-Report | Slope Intercept | 0.39 | 0.05 | 0.000 |
|  | Slope Variance | 0.14 | 0.04 | 0.000 |
| Emotional Stability Parent-Report | Slope Intercept | 0.23 | 0.04 | 0.000 |
|  | Slope Variance | 0.15 | 0.03 | 0.000 |

# Table S25d. Correlated Change Estimates for Adversity, Effortful Control, and Emotional Stability Slopes for Bivariate Latent Growth Curve Conditioned on Cohort and Gender

| Construct | *Unstandardized*  *Covariance* | *SE* | *p* | *r* |
| --- | --- | --- | --- | --- |
| Effortful Control Youth-Report | -0.05 | 0.01 | 0.000 | -0.53 |
| Effortful Control Parent-Report | -0.03 | 0.01 | 0.002 | -0.33 |
| Emotional Stability Youth-Report | -0.05 | 0.01 | 0.000 | -0.54 |
| Emotional Stability Parent-Report | -0.02 | 0.01 | 0.077 | -0.17 |

**Supplement C: Study Materials**

**For the manuscript “Growth Following Adversity is Rare”**

# Attachment Security to Caregiver

Fraley, R. C., Heffernan, M. E., Vicary, A. M., & Brumbaugh, C. C. (2011). The experiences in close relationships—Relationship Structures Questionnaire: A method for assessing attachment orientations across relationships. *Psychological Assessment*, *23*(3), 615–625. [https://doi.org/10.1037/a0022898](https://psycnet.apa.org/doi/10.1037/a0022898)

1 = strongly disagree; 7 = strongly agree

Please answer the following 10 questions about your parent (the one who came with you):

1. It helps to turn to this person in times of need.
2. I usually discuss my problems and concerns with this person.
3. I talk things over with this person.
4. I find it easy to depend on this person.
5. I don't feel comfortable opening up to this person.
6. I prefer not to show this person how I feel deep down.
7. I often worry that this person doesn't really care for me.
8. I'm afraid that this person may abandon me.
9. I worry that this person won't care about me as much as I care about him or her.

10. I don’t trust this person.

# Selected Items from the Parenting Styles Scale

Lamborn, S. D., Mounts, N. S., Steinberg, L., & Dornbusch, S. M. (1991). Patterns of competence and adjustment among adolescents from authoritative, authoritarian, indulgent, and neglectful families. *Child Development*, *62*(5), 1049-1065.

*What do you think is usually true or usually false about your (parent)?*

1. **10. I can count on him/her to help me out if I have some kind of problem.**

Usually true = 1

Usually false = 0

1. **11. He/she keeps pushing me to do my best in whatever I do.**

Usually true = 1

Usually false = 0

1. **12. He/she keeps pushing me to think independently.**

Usually true = 1

Usually false = 0

1. **13. He/she helps me with my school work if there is something I don’t understand.**

Usually true = 1

Usually false = 0

1. **14. When he/she wants me to do something, he/she explains why.**

Usually true = 1

Usually false = 0

1. **15. When you get a poor grade in school, how often do your parents encourage you to try harder?**

Never = 0

Sometimes = 1

Usually = 1

1. **16. When you get a good grade in school, how often do your parents praise you?**

Never = 0

Sometimes = 1

Usually = 1

1. **17. How much do your parents really know who your friends are?**

Don’t know = 0

Know a Little = 1

Know a Lot = 1

*How often do these things happen in your family?*

1. **18. My parents spend time just talking with me.**

Almost every day = 1

A few times a week = 1

A few times a month = 1

Almost never = 0

1. **19. My family does something fun together.**

Almost every day = 1

A few times a week = 1

A few times a month = 1

Almost never = 0

#

# Selected Items from the Revised Peer Experiences Questionnaire (RPEQ; De Los Reyes & Prinstein, 2004)

1. “Another kid helped me when I was having a problem”
2. “Another kid stuck up for me when was being picked on or excluded.”

Never = 1

Once or twice = 2

A few times = 3

About once a week = 4

A few times a week = 5

#

# Self-Esteem

Selected items from the Rosenberg Self-Esteem Scale.

Rosenberg, Morris. 1989. Society and the Adolescent Self-Image.  Revised edition. Middletown, CT: Wesleyan University Press.

Indicate how much you agree with each of the following sentences.

1.  ON THE WHOLE, I AM SATISFIED WITH MYSELF.

A. STRONGLY AGREE

B. AGREE

C. DISAGREE

D. STRONGLY DISAGREE

2.  AT TIMES I THINK I AM NO GOOD AT ALL.

A. STRONGLY AGREE

B. AGREE

C. DISAGREE

D. STRONGLY DISAGREE

3.  I FEEL THAT I HAVE A NUMBER OF GOOD QUALITIES.

A. STRONGLY AGREE

B. AGREE

C. DISAGREE

D. STRONGLY DISAGREE

4. I AM ABLE TO DO THINGS AS WELL AS MOST OTHER PEOPLE.

A. STRONGLY AGREE

B. AGREE

C. DISAGREE

D. STRONGLY DISAGREE

5.  I FEEL I DO NOT HAVE MUCH TO BE PROUD OF.

A. STRONGLY AGREE

B. AGREE

C. DISAGREE

D. STRONGLY DISAGREE
