# Supplementary material for: Growth following adversity is rare: Evidence from a multi-informant longitudinal study of children and adolescents
Source: J Res Pers. Author manuscript; Available in PMC 2026 Jun 10. (PMC13249454; doi:10.1016/j.jrp.2025.104628)
Supplement: 3 [file NIHMS2177602-supplement-3.pdf]

Supplement B: Supplemental Results
For the manuscript "Growth Following Adversity is Rare"

Table of Contents

Note on abbreviations in the Supplemental Materials. 4

Table S1a. Descriptive Statistics, Reliability, and Manifest Correlations for Main Study Variables 5

Table S1b. Descriptive Statistics, Reliability, Test-Retest Stability and Correlation with Adversity for EATQ Facets 7

Table S2. Attrition 8

Table S3. Univariate Model Fit 11

Table S4. Initial Elevation Bias 14

Table S5. Standardized Mean Difference Between t1 and t3 for Effortful Control, Emotional Stability, and Facets 16

Table S6. Full Output for Youth and Parent Report Models for Univariate Growth in Facets 17

Table S7. Bivariate Latent Growth Curve Model Fit 28

Table S8a. Supplemental Slope-Slope and Intercept-Slope Correlations 30

Table S8b. Full Output for Youth and Parent Report Models for Bivariate Growth in Facets and Adversity 32

Table S9. Fit Indices for Univariate Latent Growth Curve Models for Effortful Control and Emotional Stability Conditioned on Cohort and Adversity Factor Scores 41

Table S10. Youth and Parent Report Estimates for Prevalence of Growth Despite Adversity (Estimates for Univariate Model Conditioned on Initial Adversity, Change in Adversity, and Cohort) 42

Table S11. Fit indices for all Research Question 04 models 43

Table S12. Univariate Latent Difference Score Models for Parenting and Prosocial 44

Table S13. Associations between Personality, Adversity, and Third Variable 45

Table S14. Regression of Personality Intercept and Slope on Adversity and Third Variable 50

Domain Level Adversity Robustness Analyses 53
Table S15. Adversity Domain Univariate Latent Growth Curve Model Fit 54
Table S16. Adversity Domain Univariate Latent Growth Curve Model Parameters 55
Table S17. Adversity Domain Bivariate Latent Growth Curve Model Fit 58
Table S18. Adversity Domain Bivariate Latent Growth Curve Model Parameters 61
Table S19a. uv.onPCR.adv.fits.indices.csv 85
Table S19b. uv.onPPR.adv.fits.indices.csv 86
Table S19c. uv.onDisc.adv.fits.indices.csv 87
Table S20a. Youth and Parent Report Estimates for Prevalence of Growth Despite Parent-Child Conflict (Estimates for Univariate Model Conditioned on Initial Adversity, Change in Adversity, and Cohort) 88
Table S20b. Youth and Parent Report Estimates for Prevalence of Growth Despite Parent-Parent Conflict (Estimates for Univariate Model Conditioned on Initial Adversity, Change in Adversity, and Cohort) 89
Table S20c. Youth and Parent Report Estimates for Prevalence of Growth Despite Discrimination/Acculturation Stress(Estimates for Univariate Model Conditioned on Initial Adversity, Change in Adversity, and Cohort) 90
Table S21. Fit indices for domain specific trivariate regression models 91
Table S22. Parameters from adversity domain trivariate regression models 92
Table S23a. Demographic differences between youth who grew or did not grow in youth-reported effortful control following adversity 97
Table S23b. Demographic differences between youth who grew or did not grow in parent-reported effortful control following adversity 98
Table S23c. Demographic differences between youth who grew or did not grow in youth-reported emotional stability following adversity 99
Table S23d. Demographic differences between youth who grew or did not grow in parent-reported emotional stability following adversity 100

Table S24a. Demographic differences between youth who grew in youth-reported effortful control despite adversity or without adversity *101*

Table S24b. Demographic differences between youth who grew in parent-reported effortful control despite adversity or without adversity *102*

Table S24c. Demographic differences between youth who grew in youth-reported emotional stability despite adversity or without adversity *103*

Table S24d. Demographic differences between youth who grew in parent-reported emotional stability despite adversity or without adversity *104*

Table S25a. Regression of Univariate LGC Effortful Control and Emotional Stability Intercepts and Slopes on Gender (0 = Male, 1 = Female) *105*

Table S25b. Regression of Bivariate LGC Effortful Control and Emotional Stability Intercepts and Slopes on Gender (0 = Male, 1 = Female) *105*

Table S25c. Unstandardized Slopes for Univariate Latent Growth Curve Models of Youth and Parent-Reported Effortful Control and Emotional Stability Conditioned on Cohort and Gender *106*

Table S25d. Correlated Change Estimates for Adversity, Effortful Control, and Emotional Stability Slopes for Bivariate Latent Growth Curve Conditioned on Cohort and Gender *106*

Attachment Security to Caregiver *107*

Selected Items from the Parenting Styles Scale *108*

Selected Items from the Revised Peer Experiences Questionnaire (RPEQ; De Los Reyes & Prinstein, 2004) *111*

Self-Esteem *112*

Note on abbreviations in the Supplemental Materials.

There are several consistent abbreviations in the output for the supplemental materials that should be kept in mind. All of the EATQ personality constructs have the same naming convention: a 2-4 letter abbreviation, followed by a “y” or “p” to denote whether the construct was youth or parent reported, followed by a number to denote the measurement occasion (1, 2, or 3). For example, “ec.y.3” would represent youth-reported effortful control at the third wave, while “ne.p.1” represents parent-reported emotional stability (i.e., reverse-scored negative emotionality) at the first wave.

Activation Control = ac
Attention = at
Inhibitory Control = ic
Effortful Control = ec
Aggression = ag
Fear = fear
Frustration = fr
Shy = shy
Negative Emotionality/Emotional Stability = ne
Avoidance = avoid
Anxiety = anx
Parenting Style = PSS
Prosocial Behavior = ProSoc
Self-Esteem = SEQ

Table S1a. Descriptive Statistics, Reliability, and Manifest Correlations for Main Study Variables

Construct	*M*	*SD*	α	1.	2.	3.	4.	5.	6.	7.	8.	9.	10.
1. EC (t1.y)	3.42	0.55	0.74										
2. EC (t2.y)	3.56	0.57	0.81	0.58									
3. EC (t3.y)	3.53	0.55	0.81	0.50	0.67								
4. EC (t1.p)	3.24	0.56	0.85	0.34	0.25	0.30							
5. EC (t2.p)	3.37	0.58	0.86	0.28	0.31	0.36	0.77						
6. EC (t3.p)	3.36	0.58	0.86	0.30	0.29	0.41	0.70	0.81					
7. ES (t1.y)	3.34	0.53	0.79	0.38	0.26	0.14	0.15	0.12	0.05				
8. ES (t2.y)	3.64	0.5	0.83	0.32	0.50	0.39	0.07	0.09	0.05	0.46			
9. ES (t3.y)	3.64	0.49	0.82	0.24	0.36	0.53	0.12	0.13	0.12	0.32	0.55		
10. ES (t1.p)	3.43	0.49	0.82	0.09	0.08	0.10	0.42	0.37	0.32	0.15	0.21	0.18	
11. ES (t2.p)	3.64	0.53	0.86	0.14	0.13	0.23	0.39	0.52	0.45	0.17	0.21	0.26	0.72
12. ES (t3.p)	3.69	0.52	0.86	0.12	0.11	0.20	0.36	0.45	0.48	0.14	0.20	0.24	0.64
13. Adv. (t1)	1.64	0.36	0.79	-0.30	-0.31	-0.30	-0.18	-0.20	-0.18	-0.18	-0.20	-0.20	-0.08
14. Adv. (t2)	1.56	0.37	0.81	-0.20	-0.33	-0.31	-0.15	-0.19	-0.20	-0.09	-0.19	-0.23	-0.11
15. Adv. (t3)	1.55	0.43	0.80	-0.14	-0.18	-0.34	-0.08	-0.15	-0.25	-0.03	-0.14	-0.29	-0.08
16. Anxiety (t1.y)	1.67	1.10	0.82	-0.22	-0.11	-0.09	-0.16	-0.13	-0.09	-0.25	-0.16	-0.07	-0.17
17. Anxiety (t2.y)	1.58	0.97	0.83	-0.19	-0.26	-0.24	-0.13	-0.21	-0.13	-0.21	-0.28	-0.23	-0.19
18. Anxiety (t3.y)	1.60	1.06	0.82	-0.16	-0.16	-0.26	-0.19	-0.26	-0.21	-0.07	-0.15	-0.25	-0.18
19. Avoidance (t1.y)	2.72	1.31	0.81	-0.26	-0.17	-0.08	-0.11	-0.07	-0.05	-0.04	-0.10	-0.04	-0.04
20. Avoidance (t2.y)	2.83	1.41	0.80	-0.23	-0.32	-0.24	-0.07	-0.11	-0.10	-0.06	-0.14	-0.13	-0.07
21. Avoidance (t3.y)	2.94	1.47	0.82	-0.20	-0.22	-0.29	-0.13	-0.15	-0.16	-0.01	-0.07	-0.18	-0.11
22. Parenting (t2.y)	0.85	0.16	0.59	0.15	0.20	0.16	0.13	0.10	0.13	0.07	0.13	0.11	0.17
23. Parenting (t3.y)	0.89	0.16	0.71	0.10	0.11	0.18	0.09	0.09	0.12	-0.01	0.06	0.10	0.14
24. Peer support (t2.y)	2.62	0.99	0.65	0.06	0.08	0.10	0.04	0.07	0.04	0.00	0.01	0.05	0.04
25. Peer support (t3.y)	2.57	0.91	0.60	0.09	0.09	0.15	0.12	0.08	0.09	-0.01	0.06	0.03	0.03

Table S1a. Descriptive Statistics, Reliability, and Manifest Correlations for Main Study Variables (continued)

Construct	11.	12.	13.	14.	15.	16.	17.	18.	19.	20.	21.	22.	23.	24.
1. EC (t1y)														
2. EC (t2y)														
3. EC (t3y)														
4. EC (t1p)														
5. EC (t2p)														
6. EC (t3p)														
7. NE (t1y)														
8. NE t2y)														
9. NE t3y)														
10. NE (t1p)														
11. NE (t2p)														
12. NE (t3p)	0.77													
13. Adv. t1	-0.14	-0.14												
14. Adv. t2	-0.18	-0.13	0.64											
15. Adv. t3	-0.15	-0.22	0.42	0.53										
16. Anxiety t1	-0.19	-0.20	0.20	0.06	0.02									
17. Anxiety t2	-0.24	-0.21	0.13	0.19	0.14	0.27								
18. Anxiety t3	-0.22	-0.17	0.11	0.11	0.20	0.22	0.47							
19. Avoidance t3	-0.09	-0.04	0.22	0.09	0.08	0.41	0.26	0.21						
20. Avoidance t2	-0.13	-0.09	0.11	0.19	0.11	0.17	0.41	0.34	0.49					
21. Avoidance t3	-0.13	-0.14	0.12	0.19	0.22	0.13	0.39	0.47	0.39	0.63				
22. Parenting t2	0.14	0.07	-0.05	-0.12	-0.06	-0.15	-0.32	-0.27	-0.27	-0.49	-0.35			
23. Parenting t3	0.13	0.11	-0.13	-0.16	-0.23	-0.13	-0.35	-0.37	-0.26	-0.38	-0.55	0.45		
24. Peer support t2	0.09	0.08	-0.06	-0.05	0.02	-0.04	-0.19	-0.12	-0.16	-0.24	-0.25	0.21	0.16	
25. Peer support t3	0.00	0.01	-0.04	-0.02	-0.06	0.05	-0.06	-0.03	-0.09	-0.14	-0.18	0.13	0.21	0.33

Table S1b. Descriptive Statistics, Reliability, Test-Retest Stability and Correlation with Adversity for EATQ Facets

Construct	*M*	*SD*	Alpha	t2 Stability	t3 Stability	Adv. t1	Adv. t2	Adv. t3
ac.y.1	3.27	0.84	0.66	0.56	0.51	-0.29	-0.21	-0.14
ac.y.2	3.3	0.86	0.76	—	0.64	-0.27	-0.28	-0.14
ac.y.3	3.16	0.84	0.77	—	—	-0.26	-0.25	-0.3
ac.p.1	3.11	0.78	0.81	0.73	0.65	-0.2	-0.18	-0.09
ac.p.2	3.21	0.78	0.82	—	0.77	-0.21	-0.22	-0.15
ac.p.3	3.13	0.82	0.84	—	—	-0.17	-0.2	-0.24
at.y.1	3.38	0.63	0.5	0.42	0.3	-0.25	-0.16	-0.1
at.y.2	3.54	0.61	0.59	—	0.58	-0.26	-0.29	-0.17
at.y.3	3.53	0.59	0.58	—	—	-0.24	-0.29	-0.3
at.p.1	3.05	0.6	0.6	0.66	0.62	-0.13	-0.13	-0.07
at.p.2	3.16	0.61	0.64	—	0.68	-0.15	-0.12	-0.12
at.p.3	3.18	0.59	0.58	—	—	-0.13	-0.12	-0.17
ic.y.1	3.63	0.63	0.31	0.45	0.38	-0.16	-0.08	-0.09
ic.y.2	3.85	0.59	0.43	—	0.48	-0.22	-0.24	-0.15
ic.y.3	3.89	0.63	0.56	—	—	-0.22	-0.2	-0.22
ic.p.1	3.64	0.58	0.51	0.6	0.52	-0.1	-0.03	0
ic.p.2	3.85	0.59	0.54	—	0.65	-0.14	-0.13	-0.1
ic.p.3	3.89	0.6	0.57	—	—	-0.15	-0.17	-0.23
ag.y.1	1.97	0.73	0.76	0.55	0.4	-0.28	-0.19	-0.14
ag.y.2	1.87	0.68	0.78	—	0.57	-0.25	-0.26	-0.16
ag.y.3	1.9	0.69	0.8	—	—	-0.24	-0.28	-0.3
ag.p.1	2.37	0.69	0.75	0.69	0.62	-0.18	-0.14	-0.13
ag.p.2	2.17	0.7	0.78	—	0.72	-0.18	-0.21	-0.18
ag.p.3	2.14	0.65	0.74	—	—	-0.16	-0.19	-0.28

fear.y.1	2.81	0.81	0.63	0.46	0.36	-0.02	0	0.04
fear.y.2	2.24	0.71	0.65	—	0.51	-0.07	-0.04	0.01
fear.y.3	2.1	0.65	0.66	—	—	-0.02	-0.06	-0.08
fear.p.1	2.48	0.71	0.64	0.65	0.58	-0.01	-0.04	-0.04
fear.p.2	2.14	0.69	0.68	—	0.67	-0.05	-0.09	-0.09
fear.p.3	2.04	0.68	0.69	—	—	-0.07	-0.04	-0.08
fr.y.1	3.18	0.72	0.71	0.44	0.32	-0.18	-0.06	-0.03
fr.y.2	2.9	0.71	0.76	—	0.49	-0.16	-0.17	-0.12
fr.y.3	2.93	0.69	0.75	—	—	-0.18	-0.19	-0.22
fr.p.1	2.93	0.67	0.77	0.64	0.58	-0.13	-0.13	-0.07
fr.p.2	2.72	0.71	0.79	—	0.66	-0.19	-0.2	-0.15
fr.p.3	2.7	0.71	0.8	—	—	-0.16	-0.15	-0.22
shy.y.1	2.57	1.02	0.75	0.48	0.44	0	0.01	0.03
shy.y.2	2.3	0.91	0.8	—	0.61	-0.04	-0.04	-0.08
shy.y.3	2.43	0.99	0.84	—	—	-0.07	-0.08	-0.14
shy.p.1	2.51	0.9	0.86	0.71	0.68	0.11	0.02	0.02
shy.p.2	2.44	0.88	0.86	—	0.76	0.02	-0.01	0
shy.p.3	2.42	0.9	0.86	—	—	0	0.01	-0.03

Table S2. Attrition

Dependent Variable	*Dropout M*	*Stay M*	*t/z*	*df*	*p*	*d/h*
Site	0.44	0.47	-0.63	1	0.526	-0.06
Child Gender	0.54	0.56	-0.55	1	0.580	-0.05
Child Ethnicity	0.21	0.10	3.82	1	0.000	0.32
Child Race	0.44	0.28	3.78	1	0.000	0.33
Child Age	12.06	11.77	1.29	254.32	.198	0.12
Child Grade	6.20	6.02	0.81	259.05	.418	0.07
Parent Gender	0.94	0.92	0.67	1	0.501	0.06
Parent Ethnicity	0.15	0.09	1.93	1	0.054	0.17
Parent Race	0.35	0.25	2.58	1	0.010	0.23
Parent Marital Status	0.73	0.77	-1.21	1	0.227	-0.11
Parent Education	5.24	5.75	-4.47	250.05	< .001***	-0.42
Other Parent Education	4.67	5.35	-4.45	244.96	< .001***	-0.43
School Lunch (PR)	0.32	0.14	4.98	1	0.000	0.42
Food Stamps (PR)	0.12	0.05	3.26	1	0.001	0.27
Total Income (PR)	88,877.78	102,382.34	-1.64	218.98	.103	-0.17
People Home (PR)	4.49	4.16	2.32	207.33	.021*	0.25
adv.1	1.70	1.62	2.10	192.64	.037*	0.23
ec.y.1	3.39	3.43	-0.87	249.66	.386	-0.08
ec.p.1	3.18	3.25	-1.27	241.90	.204	-0.12
ac.y.1	3.22	3.29	-0.88	253.75	.378	-0.08
ac.p.1	3.04	3.12	-1.09	233.17	.276	-0.11
at.y.1	3.36	3.39	-0.40	248.78	.688	-0.04
at.p.1	3.02	3.06	-0.79	251.33	.433	-0.07
ic.y.1	3.59	3.64	-0.75	256.17	.455	-0.07
ic.p.1	3.58	3.66	-1.35	252.10	.177	-0.12
ne.y.1	3.28	3.36	-1.48	246.44	.141	-0.14
ne.p.1	3.38	3.45	-1.57	264.90	.117	-0.14
ag.y.1	3.92	4.06	-1.90	241.45	.059	-0.18
ag.p.1	3.57	3.65	-1.17	241.78	.244	-0.11

Dependent Variable	*Dropout M*	*Stay M*	*t/z*	*df*	*p*	*d/h*
fear.y.1	3.17	3.20	-0.35	247.95	.724	-0.03
fear.p.1	3.46	3.53	-1.03	262.64	.306	-0.09
fr.y.1	2.74	2.85	-1.59	255.74	.112	-0.15
fr.p.1	3.06	3.08	-0.33	249.94	.742	-0.03
shy.y.1	3.44	3.43	0.10	267.44	.922	0.01
shy.p.1	3.40	3.51	-1.33	243.56	.185	-0.13
anx.y.1	1.71	1.66	0.43	242.45	.669	0.04
avoid.y.1	2.72	2.72	0.00	233.45	1.00	0.00

Note: Parent Education was originally a 9-category nominal variable (1 = 8th grade or less; 2 = some high school; 3 = finished high school; 4 = completed GED; 5 = vocational/trade/business school; 6 = some college or 2 year degree; 7 = finished 4 year degree; 8 = masters or equivalent; 9 = other advanced degree). Levels 3 and 4, and 8 and 9, were collapsed together in order to make an ordered numeric variable so that inferential tests for attrition were interpretable. Child Race and Parent Race were also originally multi-category nominal variables (descriptives are in Hankin et al., 2015) that were re-coded into binary variables for the purposes of attrition tests (1 = non-Caucasian; Caucasian = 0). Parent Marital Status was re-coded so that 0 = Not Married and 1 = Married. For both of the Gender variables, 0 = Male and 1 = Female.

Table S3. Univariate Model Fit

chisq	df	rmsea	tli	cfi	aic	bic	ModID
63.719	4	0.148	0.919	0.892	2465.248	2487.873	ec.y.uv.Mod0
24.122	1	0.184	0.875	0.958	2431.652	2467.852	ec.y.uv.Mod1
31.164	2	0.146	0.858	0.953	2379.031	2424.282	ec.y.uv.Mod2
85.888	4	0.174	0.943	0.924	2064.142	2086.723	ec.p.uv.Mod0
23.199	1	0.181	0.938	0.979	2007.453	2043.582	ec.p.uv.Mod1
25.842	2	0.132	0.934	0.978	2002.635	2047.885	ec.p.uv.Mod2
33.596	4	0.104	0.957	0.943	3963.11	3985.735	ac.y.uv.Mod0
9.13	1	0.109	0.953	0.984	3944.644	3980.845	ac.y.uv.Mod1
10.119	2	0.077	0.963	0.988	3810.239	3855.489	ac.y.uv.Mod2
33.925	4	0.105	0.976	0.968	3310.256	3332.837	ac.p.uv.Mod0
15.378	1	0.146	0.954	0.985	3297.708	3333.837	ac.p.uv.Mod1
19.765	2	0.114	0.943	0.981	3301.262	3346.513	ac.p.uv.Mod2
68.951	4	0.154	0.847	0.796	3045.127	3067.753	at.y.uv.Mod0
14.047	1	0.138	0.877	0.959	2996.223	3032.423	at.y.uv.Mod1
21.441	2	0.119	0.826	0.942	2987.608	3032.859	at.y.uv.Mod2
41.934	4	0.118	0.96	0.946	2569.137	2591.718	at.p.uv.Mod0
4.463	1	0.072	0.985	0.995	2537.666	2573.795	at.p.uv.Mod1
7.386	2	0.063	0.978	0.993	2513.224	2558.474	at.p.uv.Mod2
102.19	4	0.19	0.741	0.655	3124.009	3146.634	ic.y.uv.Mod0
17.714	1	0.157	0.824	0.941	3045.533	3081.733	ic.y.uv.Mod1
22.686	2	0.123	0.789	0.93	3042.34	3087.59	ic.y.uv.Mod2
136.946	4	0.222	0.821	0.762	2758.768	2781.349	ic.p.uv.Mod0
17.866	1	0.158	0.909	0.97	2645.688	2681.818	ic.p.uv.Mod1
18.587	2	0.11	0.915	0.972	2621.63	2666.881	ic.p.uv.Mod2
218.997	4	0.281	0.5	0.333	2520.634	2543.259	ne.y.uv.Mod0
70.896	1	0.32	0.35	0.783	2378.533	2414.733	ne.y.uv.Mod1

86.559	2	0.249	0.264	0.755	2372.982	2418.232	ne.y.uv.Mod2
249.069	4	0.301	0.795	0.727	1999.098	2021.679	ne.p.uv.Mod0
39.592	1	0.239	0.871	0.957	1795.621	1831.751	ne.p.uv.Mod1
41.186	2	0.169	0.872	0.957	1777.776	1823.026	ne.p.uv.Mod2
25.497	4	0.089	0.961	0.948	3402.268	3424.893	ag.y.uv.Mod0
5.172	1	0.078	0.97	0.99	3387.943	3424.144	ag.y.uv.Mod1
18.879	2	0.111	0.891	0.964	3349.737	3394.987	ag.y.uv.Mod2
106.028	4	0.194	0.902	0.869	3019.182	3041.762	ag.p.uv.Mod0
18.683	1	0.162	0.932	0.977	2937.836	2973.966	ag.p.uv.Mod1
20.553	2	0.117	0.929	0.976	2936.289	2981.54	ag.p.uv.Mod2
382.926	4	0.373	0.044	0	3983.429	4006.054	fear.y.uv.Mod0
69.471	1	0.317	0.309	0.77	3675.974	3712.174	fear.y.uv.Mod1
77.644	2	0.235	0.328	0.776	3644.793	3690.044	fear.y.uv.Mod2
266.55	4	0.312	0.699	0.598	3369.168	3391.749	fear.p.uv.Mod0
39.722	1	0.239	0.822	0.941	3148.34	3184.469	fear.p.uv.Mod1
40.489	2	0.168	0.834	0.945	3107.221	3152.471	fear.p.uv.Mod2
94.653	4	0.182	0.749	0.665	3625.148	3647.773	fr.y.uv.Mod0
36.417	1	0.228	0.608	0.869	3572.912	3609.112	fr.y.uv.Mod1
42.168	2	0.172	0.578	0.859	3564.752	3610.002	fr.y.uv.Mod2
104.926	4	0.193	0.88	0.84	3217.917	3240.498	fr.p.uv.Mod0
17.649	1	0.157	0.921	0.974	3136.64	3172.77	fr.p.uv.Mod1
19.421	2	0.113	0.919	0.973	3129.517	3174.768	fr.p.uv.Mod2
50.669	4	0.131	0.915	0.887	4572.885	4595.51	shy.y.uv.Mod0
33.13	1	0.217	0.766	0.922	4561.346	4597.547	shy.y.uv.Mod1
37.206	2	0.161	0.753	0.918	4550.075	4595.325	shy.y.uv.Mod2
16.364	4	0.068	0.99	0.986	3737.72	3760.301	shy.p.uv.Mod0
0.651	1	0	1.001	1	3728.007	3764.136	shy.p.uv.Mod1
2.321	2	0.015	0.999	1	3728.919	3774.169	shy.p.uv.Mod2
48.505	4	0.13	0.925	0.9	1131.982	1154.465	adv.uv.Mod0

7.15	1	0.096	0.958	0.986	1096.627	1132.601	adv.uv.Mod1
9.007	2	0.072	0.958	0.986	1042.476	1087.726	adv.uv.Mod2
54.894	4	0.138	0.906	0.875	5712.707	5735.259	avoid.uv.Mod0
0.014	1	0	1.007	1	5663.827	5699.909	avoid.uv.Mod1
4.06	2	0.039	0.986	0.995	5644.037	5689.287	avoid.uv.Mod2
21.735	4	0.081	0.92	0.893	4940.863	4963.414	anx.uv.Mod0
1.984	1	0.038	0.982	0.994	4927.112	4963.194	anx.uv.Mod1
3.596	2	0.034	0.972	0.991	4926.924	4972.174	anx.uv.Mod2

Note: Mod0 represents the No Growth model, Mod1 represents the unconditional univariate model, and Mod2 represents the conditional (on cohort) univariate model.

Table S4. Initial Elevation Bias

Construct		C1 Mean	C2 Mean	C3 Mean	W1-W3 Cohen's *d* difference	W1-W3 *p*-value
Emotional Stability (y)	C1 v. C2 (6th grade)	3.72	3.38		-0.68	0.000
	C2 v. C3 (9th grade)		3.61	3.35	-0.55	0.000
Emotional Stability (p)	C1 v. C2 (6th grade)	3.58	3.46		-0.27	0.012
	C2 v. C3 (9th grade)		3.68	3.51	-0.32	0.001
Aggression (y)	C1 v. C2 (6th grade)	4.30	4.03		-0.41	0.000
	C2 v. C3 (9th grade)		4.01	3.86	-0.21	0.033
Aggression (p)	C1 v. C2 (6th grade)	3.80	3.64		-0.25	0.016
	C2 v. C3 (9th grade)		3.83	3.68	-0.21	0.032
Fear (y)	C1 v. C2 (6th grade)	3.76	3.22		-0.73	0.000
	C2 v. C3 (9th grade)		3.97	3.41	-0.85	0.000
Fear (p)	C1 v. C2 (6th grade)	3.74	3.53		-0.31	0.003
	C2 v. C3 (9th grade)		4.04	3.70	-0.49	0.000
Frustration (y)	C1 v. C2 (6th grade)	3.21	2.87		-0.47	0.000
	C2 v. C3 (9th grade)		3.00	2.76	-0.34	0.001
Frustration (p)	C1 v. C2 (6th grade)	3.19	3.14		-0.08	0.439
	C2 v. C3 (9th grade)		3.29	3.08	-0.29	0.003
Shy (y)	C1 v. C2 (6th grade)	3.67	3.51		-0.16	0.112
	C2 v. C3 (9th grade)		3.56	3.52	-0.05	0.634
Shy (p)	C1 v. C2 (6th grade)	3.55	3.50		-0.05	0.611
	C2 v. C3 (9th grade)		3.51	3.56	0.06	0.570
Effortful Control (y)	C1 v. C2 (6th grade)	3.75	3.45		-0.52	0.000
	C2 v. C3 (9th grade)		3.48	3.27	-0.41	0.000
Effortful Control (p)	C1 v. C2 (6th grade)	3.32	3.25		-0.13	0.226
	C2 v. C3 (9th grade)		3.34	3.30	-0.08	0.410
Activation Control (y)	C1 v. C2 (6th grade)	3.66	3.33		-0.43	0.000
	C2 v. C3 (9th grade)		3.04	2.89	-0.19	0.049
Activation Control (p)	C1 v. C2 (6th grade)	3.21	3.14		-0.09	0.369

	C2 v. C3 (9th grade)		3.07	3.05	-0.03	0.793
Attention (y)	C1 v. C2 (6th grade)	3.64	3.39		-0.38	0.000
	C2 v. C3 (9th grade)		3.52	3.33	-0.34	0.001
Attention (p)	C1 v. C2 (6th grade)	3.05	3.04		-0.02	0.850
	C2 v. C3 (9th grade)		3.18	3.19	0.01	0.913
Inhibitory Control (y)	C1 v. C2 (6th grade)	3.97	3.64		-0.49	0.000
	C2 v. C3 (9th grade)		3.85	3.58	-0.50	0.000
Inhibitory Control (p)	C1 v. C2 (6th grade)	3.81	3.67		-0.24	0.019
	C2 v. C3 (9th grade)		3.91	3.77	-0.24	0.014

Note: Cohort 1 and 2 are compared when both cohorts are in the 6th grade (similarly for Cohorts 2 and 3 for 9th grade); therefore, differences at this assessment are likely due to initial elevation bias, as Cohorts 2 and 3 reliably self-report increased negative emotionality, aggression and fear.

Table S5. Standardized Mean Difference Between t1 and t3 for Effortful Control, Emotional Stability, and Facets

Construct	Std. Mean Diff.	3rd Grade Std. Mean Diff.	6th Grade Std. Mean Diff.	9th Grade Std. Mean Diff.
Effortful Control (y)	0.19	0.35	0.05	0.21
Effortful Control (p)	0.22	0.31	0.16	0.19
Activation Control (y)	-0.13	0.07	-0.36	-0.08
Activation Control (p)	0.03	0.11	-0.09	0.09
Attention (y)	0.23	0.31	0.21	0.18
Attention (p)	0.21	0.23	0.24	0.16
Inhibitory Control (y)	0.42	0.45	0.31	0.55
Inhibitory Control (p)	0.44	0.6	0.44	0.32
Negative Emotionality (y)	0.57	0.67	0.49	0.55
Negative Emotionality (p)	0.53	0.54	0.54	0.53
Aggression (y)	0.11	0.12	-0.04	0.24
Aggression (p)	0.33	0.35	0.29	0.36
Fear (y)	0.86	0.9	0.98	0.79
Fear (p)	0.63	0.6	0.79	0.54
Frustration (y)	0.33	0.45	0.18	0.38
Frustration (p)	0.34	0.31	0.25	0.46
Shy (y)	0.14	0.39	0.05	-0.03
Shy (p)	0.1	0.17	0.01	0.14

Table S6. Full Output for Youth and Parent Report Models for Univariate Growth in Facets

Parameter	Estimate	SE	PValue	StdAll	Construct	ModelName
InterceptOfIntercept	3.50	0.02	0.000	8.17	ec.y	ec.y.uv.Mod0
VarianceOfIntercept	0.18	0.01	0.000	1.00	ec.y	ec.y.uv.Mod0
InterceptOfIntercept	3.44	0.02	0.000	7.42	ec.y	ec.y.uv.Mod1
VarianceOfIntercept	0.22	0.02	0.000	1.00	ec.y	ec.y.uv.Mod1
InterceptOfSlope	0.09	0.02	0.000	0.22	ec.y	ec.y.uv.Mod1
VarianceOfSlope	0.18	0.04	0.000	1.00	ec.y	ec.y.uv.Mod1
InterceptSlopeCorr	-0.06	0.02	0.006	-0.33	ec.y	ec.y.uv.Mod1
InterceptOfIntercept	3.61	0.03	0.000	7.95	ec.y	ec.y.uv.Mod2
VarianceOfIntercept	0.19	0.02	0.000	0.92	ec.y	ec.y.uv.Mod2
InterceptOfSlope	0.12	0.04	0.002	0.31	ec.y	ec.y.uv.Mod2
VarianceOfSlope	0.15	0.04	0.000	1.00	ec.y	ec.y.uv.Mod2
InterceptSlopeCorr	-0.06	0.02	0.016	-0.33	ec.y	ec.y.uv.Mod2
InterceptOnCohort	-0.16	0.03	0.000	-0.28	ec.y	ec.y.uv.Mod2
SlopeOnCohort	-0.03	0.03	0.368	-0.05	ec.y	ec.y.uv.Mod2
InterceptOfIntercept	3.32	0.02	0.000	6.54	ec.p	ec.p.uv.Mod0
VarianceOfIntercept	0.26	0.02	0.000	1.00	ec.p	ec.p.uv.Mod0
InterceptOfIntercept	3.25	0.02	0.000	6.13	ec.p	ec.p.uv.Mod1
VarianceOfIntercept	0.28	0.02	0.000	1.00	ec.p	ec.p.uv.Mod1
InterceptOfSlope	0.11	0.02	0.000	0.28	ec.p	ec.p.uv.Mod1
VarianceOfSlope	0.15	0.03	0.000	1.00	ec.p	ec.p.uv.Mod1
InterceptSlopeCorr	-0.05	0.02	0.004	-0.26	ec.p	ec.p.uv.Mod1
InterceptOfIntercept	3.17	0.03	0.000	6.01	ec.p	ec.p.uv.Mod2
VarianceOfIntercept	0.27	0.02	0.000	0.99	ec.p	ec.p.uv.Mod2
InterceptOfSlope	0.14	0.03	0.000	0.37	ec.p	ec.p.uv.Mod2
VarianceOfSlope	0.15	0.03	0.000	1.00	ec.p	ec.p.uv.Mod2
InterceptSlopeCorr	-0.05	0.02	0.008	-0.24	ec.p	ec.p.uv.Mod2

InterceptOnCohort	0.08	0.03	0.003	0.12	ec.p	ec.p.uv.Mod2
SlopeOnCohort	-0.03	0.02	0.175	-0.07	ec.p	ec.p.uv.Mod2
InterceptOfIntercept	3.24	0.03	0.000	5.06	ac.y	ac.y.uv.Mod0
VarianceOfIntercept	0.41	0.03	0.000	1.00	ac.y	ac.y.uv.Mod0
InterceptOfIntercept	3.29	0.03	0.000	4.83	ac.y	ac.y.uv.Mod1
VarianceOfIntercept	0.47	0.05	0.000	1.00	ac.y	ac.y.uv.Mod1
InterceptOfSlope	-0.13	0.04	0.000	-0.23	ac.y	ac.y.uv.Mod1
VarianceOfSlope	0.30	0.10	0.002	1.00	ac.y	ac.y.uv.Mod1
InterceptSlopeCorr	-0.10	0.05	0.060	-0.28	ac.y	ac.y.uv.Mod1
InterceptOfIntercept	3.68	0.05	0.000	5.47	ac.y	ac.y.uv.Mod2
VarianceOfIntercept	0.36	0.05	0.000	0.80	ac.y	ac.y.uv.Mod2
InterceptOfSlope	-0.08	0.06	0.173	-0.15	ac.y	ac.y.uv.Mod2
VarianceOfSlope	0.27	0.09	0.003	0.99	ac.y	ac.y.uv.Mod2
InterceptSlopeCorr	-0.10	0.05	0.055	-0.32	ac.y	ac.y.uv.Mod2
InterceptOnCohort	-0.37	0.04	0.000	-0.44	ac.y	ac.y.uv.Mod2
SlopeOnCohort	-0.05	0.04	0.263	-0.08	ac.y	ac.y.uv.Mod2
InterceptOfIntercept	3.14	0.03	0.000	4.62	ac.p	ac.p.uv.Mod0
VarianceOfIntercept	0.46	0.03	0.000	1.00	ac.p	ac.p.uv.Mod0
InterceptOfIntercept	3.13	0.03	0.000	4.50	ac.p	ac.p.uv.Mod1
VarianceOfIntercept	0.48	0.04	0.000	1.00	ac.p	ac.p.uv.Mod1
InterceptOfSlope	0.01	0.03	0.677	0.02	ac.p	ac.p.uv.Mod1
VarianceOfSlope	0.23	0.06	0.000	1.00	ac.p	ac.p.uv.Mod1
InterceptSlopeCorr	-0.06	0.04	0.127	-0.18	ac.p	ac.p.uv.Mod1
InterceptOfIntercept	3.15	0.05	0.000	4.53	ac.p	ac.p.uv.Mod2
VarianceOfIntercept	0.48	0.04	0.000	1.00	ac.p	ac.p.uv.Mod2
InterceptOfSlope	0.02	0.05	0.719	0.03	ac.p	ac.p.uv.Mod2
VarianceOfSlope	0.23	0.07	0.000	1.00	ac.p	ac.p.uv.Mod2
InterceptSlopeCorr	-0.06	0.04	0.118	-0.18	ac.p	ac.p.uv.Mod2
InterceptOnCohort	-0.02	0.04	0.563	-0.02	ac.p	ac.p.uv.Mod2

SlopeOnCohort	0.00	0.04	0.898	-0.01	ac.p	ac.p.uv.Mod2
InterceptOfIntercept	3.49	0.02	0.000	8.44	at.y	at.y.uv.Mod0
VarianceOfIntercept	0.17	0.01	0.000	1.00	at.y	at.y.uv.Mod0
InterceptOfIntercept	3.41	0.02	0.000	7.44	at.y	at.y.uv.Mod1
VarianceOfIntercept	0.21	0.03	0.000	1.00	at.y	at.y.uv.Mod1
InterceptOfSlope	0.13	0.03	0.000	0.25	at.y	at.y.uv.Mod1
VarianceOfSlope	0.28	0.06	0.000	1.00	at.y	at.y.uv.Mod1
InterceptSlopeCorr	-0.10	0.03	0.003	-0.39	at.y	at.y.uv.Mod1
InterceptOfIntercept	3.48	0.04	0.000	7.68	at.y	at.y.uv.Mod2
VarianceOfIntercept	0.20	0.03	0.000	0.98	at.y	at.y.uv.Mod2
InterceptOfSlope	0.16	0.05	0.001	0.32	at.y	at.y.uv.Mod2
VarianceOfSlope	0.27	0.06	0.000	1.00	at.y	at.y.uv.Mod2
InterceptSlopeCorr	-0.09	0.03	0.005	-0.39	at.y	at.y.uv.Mod2
InterceptOnCohort	-0.07	0.03	0.014	-0.13	at.y	at.y.uv.Mod2
SlopeOnCohort	-0.03	0.04	0.464	-0.04	at.y	at.y.uv.Mod2
InterceptOfIntercept	3.12	0.02	0.000	6.42	at.p	at.p.uv.Mod0
VarianceOfIntercept	0.24	0.02	0.000	1.00	at.p	at.p.uv.Mod0
InterceptOfIntercept	3.06	0.02	0.000	5.82	at.p	at.p.uv.Mod1
VarianceOfIntercept	0.28	0.03	0.000	1.00	at.p	at.p.uv.Mod1
InterceptOfSlope	0.12	0.02	0.000	0.35	at.p	at.p.uv.Mod1
VarianceOfSlope	0.12	0.04	0.006	1.00	at.p	at.p.uv.Mod1
InterceptSlopeCorr	-0.06	0.03	0.019	-0.33	at.p	at.p.uv.Mod1
InterceptOfIntercept	2.91	0.04	0.000	5.59	at.p	at.p.uv.Mod2
VarianceOfIntercept	0.26	0.03	0.000	0.95	at.p	at.p.uv.Mod2
InterceptOfSlope	0.15	0.04	0.000	0.45	at.p	at.p.uv.Mod2
VarianceOfSlope	0.10	0.04	0.014	1.00	at.p	at.p.uv.Mod2
InterceptSlopeCorr	-0.05	0.02	0.042	-0.31	at.p	at.p.uv.Mod2
InterceptOnCohort	0.15	0.03	0.000	0.22	at.p	at.p.uv.Mod2
SlopeOnCohort	-0.03	0.03	0.356	-0.06	at.p	at.p.uv.Mod2

InterceptOfIntercept	3.78	0.02	0.000	9.39	ic.y	ic.y.uv.Mod0
VarianceOfIntercept	0.16	0.01	0.000	1.00	ic.y	ic.y.uv.Mod0
InterceptOfIntercept	3.66	0.02	0.000	8.71	ic.y	ic.y.uv.Mod1
VarianceOfIntercept	0.18	0.03	0.000	1.00	ic.y	ic.y.uv.Mod1
InterceptOfSlope	0.27	0.03	0.000	1.01	ic.y	ic.y.uv.Mod1
VarianceOfSlope	0.07	0.06	0.248	1.00	ic.y	ic.y.uv.Mod1
InterceptSlopeCorr	-0.02	0.03	0.497	-0.21	ic.y	ic.y.uv.Mod1
InterceptOfIntercept	3.72	0.04	0.000	8.94	ic.y	ic.y.uv.Mod2
VarianceOfIntercept	0.17	0.03	0.000	0.99	ic.y	ic.y.uv.Mod2
InterceptOfSlope	0.28	0.05	0.000	1.12	ic.y	ic.y.uv.Mod2
VarianceOfSlope	0.06	0.06	0.321	1.00	ic.y	ic.y.uv.Mod2
InterceptSlopeCorr	-0.02	0.04	0.580	-0.19	ic.y	ic.y.uv.Mod2
InterceptOnCohort	-0.06	0.03	0.035	-0.12	ic.y	ic.y.uv.Mod2
SlopeOnCohort	-0.01	0.04	0.809	-0.03	ic.y	ic.y.uv.Mod2
InterceptOfIntercept	3.79	0.02	0.000	8.29	ic.p	ic.p.uv.Mod0
VarianceOfIntercept	0.21	0.02	0.000	1.00	ic.p	ic.p.uv.Mod0
InterceptOfIntercept	3.66	0.02	0.000	7.50	ic.p	ic.p.uv.Mod1
VarianceOfIntercept	0.24	0.02	0.000	1.00	ic.p	ic.p.uv.Mod1
InterceptOfSlope	0.25	0.03	0.000	0.60	ic.p	ic.p.uv.Mod1
VarianceOfSlope	0.17	0.05	0.000	1.00	ic.p	ic.p.uv.Mod1
InterceptSlopeCorr	-0.06	0.03	0.022	-0.30	ic.p	ic.p.uv.Mod1
InterceptOfIntercept	3.51	0.04	0.000	7.15	ic.p	ic.p.uv.Mod2
VarianceOfIntercept	0.23	0.02	0.000	0.94	ic.p	ic.p.uv.Mod2
InterceptOfSlope	0.32	0.04	0.000	0.77	ic.p	ic.p.uv.Mod2
VarianceOfSlope	0.17	0.04	0.000	0.98	ic.p	ic.p.uv.Mod2
InterceptSlopeCorr	-0.06	0.03	0.026	-0.29	ic.p	ic.p.uv.Mod2
InterceptOnCohort	0.14	0.03	0.000	0.24	ic.p	ic.p.uv.Mod2
SlopeOnCohort	-0.07	0.03	0.019	-0.14	ic.p	ic.p.uv.Mod2
InterceptOfIntercept	3.56	0.02	0.000	10.31	ne.y	ne.y.uv.Mod0

VarianceOfIntercept	0.12	0.01	0.000	1.00	ne.y	ne.y.uv.Mod0
InterceptOfIntercept	3.38	0.02	0.000	8.61	ne.y	ne.y.uv.Mod1
VarianceOfIntercept	0.15	0.02	0.000	1.00	ne.y	ne.y.uv.Mod1
InterceptOfSlope	0.28	0.03	0.000	0.71	ne.y	ne.y.uv.Mod1
VarianceOfSlope	0.16	0.04	0.000	1.00	ne.y	ne.y.uv.Mod1
InterceptSlopeCorr	-0.07	0.02	0.007	-0.42	ne.y	ne.y.uv.Mod1
InterceptOfIntercept	3.38	0.03	0.000	8.59	ne.y	ne.y.uv.Mod2
VarianceOfIntercept	0.15	0.02	0.000	1.00	ne.y	ne.y.uv.Mod2
InterceptOfSlope	0.37	0.04	0.000	0.96	ne.y	ne.y.uv.Mod2
VarianceOfSlope	0.14	0.04	0.000	0.97	ne.y	ne.y.uv.Mod2
InterceptSlopeCorr	-0.06	0.02	0.008	-0.43	ne.y	ne.y.uv.Mod2
InterceptOnCohort	0.01	0.03	0.824	0.01	ne.y	ne.y.uv.Mod2
SlopeOnCohort	-0.08	0.03	0.008	-0.17	ne.y	ne.y.uv.Mod2
InterceptOfIntercept	3.59	0.02	0.000	8.21	ne.p	ne.p.uv.Mod0
VarianceOfIntercept	0.19	0.01	0.000	1.00	ne.p	ne.p.uv.Mod0
InterceptOfIntercept	3.44	0.02	0.000	7.58	ne.p	ne.p.uv.Mod1
VarianceOfIntercept	0.21	0.02	0.000	1.00	ne.p	ne.p.uv.Mod1
InterceptOfSlope	0.24	0.02	0.000	0.64	ne.p	ne.p.uv.Mod1
VarianceOfSlope	0.14	0.03	0.000	1.00	ne.p	ne.p.uv.Mod1
InterceptSlopeCorr	-0.04	0.02	0.005	-0.26	ne.p	ne.p.uv.Mod1
InterceptOfIntercept	3.34	0.03	0.000	7.32	ne.p	ne.p.uv.Mod2
VarianceOfIntercept	0.20	0.02	0.000	0.97	ne.p	ne.p.uv.Mod2
InterceptOfSlope	0.23	0.03	0.000	0.59	ne.p	ne.p.uv.Mod2
VarianceOfSlope	0.15	0.03	0.000	1.00	ne.p	ne.p.uv.Mod2
InterceptSlopeCorr	-0.05	0.02	0.002	-0.28	ne.p	ne.p.uv.Mod2
InterceptOnCohort	0.10	0.02	0.000	0.17	ne.p	ne.p.uv.Mod2
SlopeOnCohort	0.01	0.02	0.543	0.03	ne.p	ne.p.uv.Mod2
InterceptOfIntercept	4.08	0.02	0.000	7.88	ag.y	ag.y.uv.Mod0
VarianceOfIntercept	0.27	0.02	0.000	1.00	ag.y	ag.y.uv.Mod0

InterceptOfIntercept	4.04	0.03	0.000	6.62	ag.y	ag.y.uv.Mod1
VarianceOfIntercept	0.37	0.04	0.000	1.00	ag.y	ag.y.uv.Mod1
InterceptOfSlope	0.07	0.03	0.034	0.13	ag.y	ag.y.uv.Mod1
VarianceOfSlope	0.29	0.07	0.000	1.00	ag.y	ag.y.uv.Mod1
InterceptSlopeCorr	-0.16	0.04	0.000	-0.50	ag.y	ag.y.uv.Mod1
InterceptOfIntercept	4.25	0.04	0.000	7.19	ag.y	ag.y.uv.Mod2
VarianceOfIntercept	0.32	0.04	0.000	0.92	ag.y	ag.y.uv.Mod2
InterceptOfSlope	0.03	0.05	0.629	0.05	ag.y	ag.y.uv.Mod2
VarianceOfSlope	0.24	0.07	0.001	1.00	ag.y	ag.y.uv.Mod2
InterceptSlopeCorr	-0.13	0.04	0.003	-0.46	ag.y	ag.y.uv.Mod2
InterceptOnCohort	-0.20	0.03	0.000	-0.28	ag.y	ag.y.uv.Mod2
SlopeOnCohort	0.04	0.04	0.328	0.07	ag.y	ag.y.uv.Mod2
InterceptOfIntercept	3.77	0.02	0.000	6.59	ag.p	ag.p.uv.Mod0
VarianceOfIntercept	0.33	0.02	0.000	1.00	ag.p	ag.p.uv.Mod0
InterceptOfIntercept	3.65	0.03	0.000	5.77	ag.p	ag.p.uv.Mod1
VarianceOfIntercept	0.40	0.03	0.000	1.00	ag.p	ag.p.uv.Mod1
InterceptOfSlope	0.21	0.02	0.000	0.46	ag.p	ag.p.uv.Mod1
VarianceOfSlope	0.21	0.05	0.000	1.00	ag.p	ag.p.uv.Mod1
InterceptSlopeCorr	-0.11	0.03	0.001	-0.38	ag.p	ag.p.uv.Mod1
InterceptOfIntercept	3.59	0.04	0.000	5.67	ag.p	ag.p.uv.Mod2
VarianceOfIntercept	0.40	0.03	0.000	0.99	ag.p	ag.p.uv.Mod2
InterceptOfSlope	0.19	0.04	0.000	0.41	ag.p	ag.p.uv.Mod2
VarianceOfSlope	0.21	0.05	0.000	1.00	ag.p	ag.p.uv.Mod2
InterceptSlopeCorr	-0.11	0.03	0.000	-0.39	ag.p	ag.p.uv.Mod2
InterceptOnCohort	0.06	0.03	0.091	0.07	ag.p	ag.p.uv.Mod2
SlopeOnCohort	0.02	0.03	0.464	0.04	ag.p	ag.p.uv.Mod2
InterceptOfIntercept	3.69	0.03	0.000	7.76	fear.y	fear.y.uv.Mod0
VarianceOfIntercept	0.23	0.02	0.000	1.00	fear.y	fear.y.uv.Mod0
InterceptOfIntercept	3.27	0.03	0.000	5.90	fear.y	fear.y.uv.Mod1

VarianceOfIntercept	0.31	0.05	0.000	1.00	fear.y	fear.y.uv.Mod1
InterceptOfSlope	0.67	0.04	0.000	1.68	fear.y	fear.y.uv.Mod1
VarianceOfSlope	0.16	0.09	0.065	1.00	fear.y	fear.y.uv.Mod1
InterceptSlopeCorr	-0.10	0.05	0.052	-0.46	fear.y	fear.y.uv.Mod1
InterceptOfIntercept	3.04	0.05	0.000	5.29	fear.y	fear.y.uv.Mod2
VarianceOfIntercept	0.30	0.05	0.000	0.91	fear.y	fear.y.uv.Mod2
InterceptOfSlope	0.83	0.06	0.000	1.87	fear.y	fear.y.uv.Mod2
VarianceOfSlope	0.18	0.08	0.031	0.93	fear.y	fear.y.uv.Mod2
InterceptSlopeCorr	-0.11	0.05	0.037	-0.46	fear.y	fear.y.uv.Mod2
InterceptOnCohort	0.22	0.04	0.000	0.31	fear.y	fear.y.uv.Mod2
SlopeOnCohort	-0.15	0.04	0.001	-0.27	fear.y	fear.y.uv.Mod2
InterceptOfIntercept	3.79	0.03	0.000	6.88	fear.p	fear.p.uv.Mod0
VarianceOfIntercept	0.30	0.02	0.000	1.00	fear.p	fear.p.uv.Mod0
InterceptOfIntercept	3.55	0.03	0.000	6.02	fear.p	fear.p.uv.Mod1
VarianceOfIntercept	0.35	0.03	0.000	1.00	fear.p	fear.p.uv.Mod1
InterceptOfSlope	0.43	0.03	0.000	1.19	fear.p	fear.p.uv.Mod1
VarianceOfSlope	0.13	0.06	0.027	1.00	fear.p	fear.p.uv.Mod1
InterceptSlopeCorr	-0.07	0.04	0.048	-0.33	fear.p	fear.p.uv.Mod1
InterceptOfIntercept	3.34	0.04	0.000	5.70	fear.p	fear.p.uv.Mod2
VarianceOfIntercept	0.32	0.03	0.000	0.92	fear.p	fear.p.uv.Mod2
InterceptOfSlope	0.46	0.04	0.000	1.32	fear.p	fear.p.uv.Mod2
VarianceOfSlope	0.12	0.06	0.040	1.00	fear.p	fear.p.uv.Mod2
InterceptSlopeCorr	-0.06	0.04	0.077	-0.32	fear.p	fear.p.uv.Mod2
InterceptOnCohort	0.21	0.03	0.000	0.28	fear.p	fear.p.uv.Mod2
SlopeOnCohort	-0.03	0.03	0.430	-0.06	fear.p	fear.p.uv.Mod2
InterceptOfIntercept	2.99	0.02	0.000	6.53	fr.y	fr.y.uv.Mod0
VarianceOfIntercept	0.21	0.02	0.000	1.00	fr.y	fr.y.uv.Mod0
InterceptOfIntercept	2.86	0.03	0.000	5.45	fr.y	fr.y.uv.Mod1
VarianceOfIntercept	0.28	0.04	0.000	1.00	fr.y	fr.y.uv.Mod1

InterceptOfSlope	0.23	0.04	0.000	0.46	fr.y	fr.y.uv.Mod1
VarianceOfSlope	0.26	0.08	0.002	1.00	fr.y	fr.y.uv.Mod1
InterceptSlopeCorr	-0.11	0.05	0.019	-0.42	fr.y	fr.y.uv.Mod1
InterceptOfIntercept	2.92	0.04	0.000	5.60	fr.y	fr.y.uv.Mod2
VarianceOfIntercept	0.27	0.04	0.000	0.99	fr.y	fr.y.uv.Mod2
InterceptOfSlope	0.30	0.06	0.000	0.63	fr.y	fr.y.uv.Mod2
VarianceOfSlope	0.23	0.08	0.004	0.99	fr.y	fr.y.uv.Mod2
InterceptSlopeCorr	-0.11	0.05	0.025	-0.42	fr.y	fr.y.uv.Mod2
InterceptOnCohort	-0.05	0.03	0.108	-0.08	fr.y	fr.y.uv.Mod2
SlopeOnCohort	-0.07	0.04	0.105	-0.12	fr.y	fr.y.uv.Mod2
InterceptOfIntercept	3.20	0.02	0.000	5.84	fr.p	fr.p.uv.Mod0
VarianceOfIntercept	0.30	0.02	0.000	1.00	fr.p	fr.p.uv.Mod0
InterceptOfIntercept	3.09	0.03	0.000	5.31	fr.p	fr.p.uv.Mod1
VarianceOfIntercept	0.34	0.03	0.000	1.00	fr.p	fr.p.uv.Mod1
InterceptOfSlope	0.23	0.03	0.000	0.54	fr.p	fr.p.uv.Mod1
VarianceOfSlope	0.18	0.06	0.002	1.00	fr.p	fr.p.uv.Mod1
InterceptSlopeCorr	-0.06	0.04	0.067	-0.26	fr.p	fr.p.uv.Mod1
InterceptOfIntercept	3.04	0.04	0.000	5.22	fr.p	fr.p.uv.Mod2
VarianceOfIntercept	0.34	0.03	0.000	1.00	fr.p	fr.p.uv.Mod2
InterceptOfSlope	0.15	0.04	0.000	0.35	fr.p	fr.p.uv.Mod2
VarianceOfSlope	0.19	0.06	0.002	0.98	fr.p	fr.p.uv.Mod2
InterceptSlopeCorr	-0.07	0.04	0.053	-0.27	fr.p	fr.p.uv.Mod2
InterceptOnCohort	0.05	0.03	0.155	0.06	fr.p	fr.p.uv.Mod2
SlopeOnCohort	0.08	0.03	0.026	0.14	fr.p	fr.p.uv.Mod2
InterceptOfIntercept	3.58	0.03	0.000	5.13	shy.y	shy.y.uv.Mod0
VarianceOfIntercept	0.49	0.04	0.000	1.00	shy.y	shy.y.uv.Mod0
InterceptOfIntercept	3.51	0.04	0.000	5.36	shy.y	shy.y.uv.Mod1
VarianceOfIntercept	0.43	0.07	0.000	1.00	shy.y	shy.y.uv.Mod1
InterceptOfSlope	0.12	0.05	0.006	0.30	shy.y	shy.y.uv.Mod1

VarianceOfSlope	0.17	0.14	0.221	1.00	shy.y	shy.y.uv.Mod1
InterceptSlopeCorr	0.02	0.08	0.812	0.07	shy.y	shy.y.uv.Mod1
InterceptOfIntercept	3.41	0.06	0.000	5.17	shy.y	shy.y.uv.Mod2
VarianceOfIntercept	0.43	0.07	0.000	0.99	shy.y	shy.y.uv.Mod2
InterceptOfSlope	0.35	0.07	0.000	0.85	shy.y	shy.y.uv.Mod2
VarianceOfSlope	0.14	0.14	0.333	0.82	shy.y	shy.y.uv.Mod2
InterceptSlopeCorr	0.03	0.08	0.715	0.12	shy.y	shy.y.uv.Mod2
InterceptOnCohort	0.10	0.05	0.031	0.12	shy.y	shy.y.uv.Mod2
SlopeOnCohort	-0.22	0.06	0.000	-0.43	shy.y	shy.y.uv.Mod2
InterceptOfIntercept	3.53	0.03	0.000	4.64	shy.p	shy.p.uv.Mod0
VarianceOfIntercept	0.58	0.04	0.000	1.00	shy.p	shy.p.uv.Mod0
InterceptOfIntercept	3.49	0.03	0.000	4.49	shy.p	shy.p.uv.Mod1
VarianceOfIntercept	0.60	0.05	0.000	1.00	shy.p	shy.p.uv.Mod1
InterceptOfSlope	0.08	0.03	0.007	0.18	shy.p	shy.p.uv.Mod1
VarianceOfSlope	0.20	0.08	0.014	1.00	shy.p	shy.p.uv.Mod1
InterceptSlopeCorr	-0.06	0.05	0.201	-0.18	shy.p	shy.p.uv.Mod1
InterceptOfIntercept	3.42	0.05	0.000	4.39	shy.p	shy.p.uv.Mod2
VarianceOfIntercept	0.60	0.05	0.000	0.99	shy.p	shy.p.uv.Mod2
InterceptOfSlope	0.11	0.05	0.032	0.23	shy.p	shy.p.uv.Mod2
VarianceOfSlope	0.21	0.08	0.012	1.00	shy.p	shy.p.uv.Mod2
InterceptSlopeCorr	-0.06	0.05	0.184	-0.18	shy.p	shy.p.uv.Mod2
InterceptOnCohort	0.07	0.04	0.079	0.08	shy.p	shy.p.uv.Mod2
SlopeOnCohort	-0.02	0.04	0.539	-0.04	shy.p	shy.p.uv.Mod2
InterceptOfIntercept	1.60	0.01	0.000	5.54	adv	adv.uv.Mod0
VarianceOfIntercept	0.08	0.01	0.000	1.00	adv	adv.uv.Mod0
InterceptOfIntercept	1.63	0.01	0.000	4.97	adv	adv.uv.Mod1
VarianceOfIntercept	0.11	0.01	0.000	1.00	adv	adv.uv.Mod1
InterceptOfSlope	-0.10	0.02	0.000	-0.35	adv	adv.uv.Mod1
VarianceOfSlope	0.08	0.02	0.000	1.00	adv	adv.uv.Mod1

InterceptSlopeCorr	-0.04	0.01	0.002	-0.46	adv	adv.uv.Mod1
InterceptOfIntercept	1.50	0.02	0.000	4.64	adv	adv.uv.Mod2
VarianceOfIntercept	0.09	0.01	0.000	0.89	adv	adv.uv.Mod2
InterceptOfSlope	-0.03	0.03	0.365	-0.10	adv	adv.uv.Mod2
VarianceOfSlope	0.06	0.02	0.002	0.96	adv	adv.uv.Mod2
InterceptSlopeCorr	-0.03	0.01	0.017	-0.39	adv	adv.uv.Mod2
InterceptOnCohort	0.13	0.02	0.000	0.32	adv	adv.uv.Mod2
SlopeOnCohort	-0.07	0.02	0.002	-0.21	adv	adv.uv.Mod2
InterceptOfIntercept	2.81	0.05	0.000	2.81	avoid.y	avoid.uv.Mod0
VarianceOfIntercept	1.00	0.08	0.000	1.00	avoid.y	avoid.uv.Mod0
InterceptOfIntercept	2.72	0.05	0.000	2.59	avoid.y	avoid.uv.Mod1
VarianceOfIntercept	1.10	0.13	0.000	1.00	avoid.y	avoid.uv.Mod1
InterceptOfSlope	0.21	0.06	0.001	0.18	avoid.y	avoid.uv.Mod1
VarianceOfSlope	1.39	0.27	0.000	1.00	avoid.y	avoid.uv.Mod1
InterceptSlopeCorr	-0.32	0.15	0.031	-0.26	avoid.y	avoid.uv.Mod1
InterceptOfIntercept	2.45	0.08	0.000	2.37	avoid.y	avoid.uv.Mod2
VarianceOfIntercept	1.02	0.13	0.000	0.96	avoid.y	avoid.uv.Mod2
InterceptOfSlope	0.19	0.11	0.076	0.17	avoid.y	avoid.uv.Mod2
VarianceOfSlope	1.31	0.27	0.000	1.00	avoid.y	avoid.uv.Mod2
InterceptSlopeCorr	-0.28	0.15	0.055	-0.25	avoid.y	avoid.uv.Mod2
InterceptOnCohort	0.26	0.06	0.000	0.20	avoid.y	avoid.uv.Mod2
SlopeOnCohort	0.02	0.08	0.782	0.02	avoid.y	avoid.uv.Mod2
InterceptOfIntercept	1.62	0.03	0.000	2.67	anx.y	anx.uv.Mod0
VarianceOfIntercept	0.37	0.04	0.000	1.00	anx.y	anx.uv.Mod0
InterceptOfIntercept	1.65	0.04	0.000	2.89	anx.y	anx.uv.Mod1
VarianceOfIntercept	0.32	0.08	0.000	1.00	anx.y	anx.uv.Mod1
InterceptOfSlope	-0.06	0.06	0.291	-0.08	anx.y	anx.uv.Mod1
VarianceOfSlope	0.50	0.19	0.009	1.00	anx.y	anx.uv.Mod1
InterceptSlopeCorr	-0.06	0.10	0.576	-0.14	anx.y	anx.uv.Mod1

InterceptOfIntercept	1.71	0.06	0.000	2.99	anx.y	anx.uv.Mod2
VarianceOfIntercept	0.32	0.08	0.000	0.99	anx.y	anx.uv.Mod2
InterceptOfSlope	-0.21	0.09	0.023	-0.30	anx.y	anx.uv.Mod2
VarianceOfSlope	0.48	0.19	0.013	0.97	anx.y	anx.uv.Mod2
InterceptSlopeCorr	-0.05	0.10	0.623	-0.13	anx.y	anx.uv.Mod2
InterceptOnCohort	-0.06	0.05	0.232	-0.08	anx.y	anx.uv.Mod2
SlopeOnCohort	0.15	0.07	0.040	0.17	anx.y	anx.uv.Mod2

Note: Mod0 = no growth model; Mod1 = unconditional, univariate latent growth curve model; Mod2 = univariate latent growth curve model conditioned on cohort.

Table S7. Bivariate Latent Growth Curve Model Fit

chisq	df	rmsea	tli	cfi	aic	bic	ModID
48.251	7	0.093	0.92	0.963	3429.858	3520.359	ec.y_bv
56.236	9	0.088	0.908	0.961	3348.667	3457.267	ec.y_bv_onC
34.702	7	0.076	0.962	0.982	3068.737	3159.179	ec.p_bv
40.641	9	0.072	0.955	0.981	2994.378	3102.979	ec.p_bv_onC
27.401	7	0.065	0.958	0.98	4965.204	5055.704	ac.y_bv
30.066	9	0.059	0.959	0.982	4813.032	4921.632	ac.y_bv_onC
25.986	7	0.063	0.971	0.987	4357.153	4447.595	ac.p_bv
33.779	9	0.064	0.961	0.983	4304.429	4413.03	ac.p_bv_onC
30.363	7	0.07	0.941	0.972	4015.75	4106.251	at.y_bv
38.968	9	0.07	0.923	0.967	3962.11	4070.71	at.y_bv_onC
14.071	7	0.039	0.987	0.994	3622.215	3712.656	at.p_bv
19.586	9	0.042	0.981	0.992	3522.198	3630.799	at.p_bv_onC
34.407	7	0.076	0.925	0.965	4099.787	4190.287	ic.y_bv
40.892	9	0.072	0.911	0.962	4047.646	4156.247	ic.y_bv_onC
28.012	7	0.066	0.956	0.98	3718.582	3809.024	ic.p_bv
30.386	9	0.059	0.956	0.981	3624.413	3733.014	ic.p_bv_onC
81.704	7	0.125	0.806	0.909	3419.016	3509.517	ne.y_bv
101.622	9	0.123	0.761	0.897	3362.295	3470.895	ne.y_bv_onC
67.611	7	0.113	0.905	0.956	2881.304	2971.746	ne.p_bv
71.125	9	0.101	0.901	0.958	2795.069	2903.67	ne.p_bv_onC
20.514	7	0.053	0.969	0.986	4402.816	4493.317	ag.y_bv
39.045	9	0.07	0.932	0.971	4333.294	4441.895	ag.y_bv_onC
36.384	7	0.079	0.95	0.977	3999.846	4090.288	ag.p_bv
39.935	9	0.071	0.946	0.977	3931.668	4040.269	ag.p_bv_onC
79.42	7	0.123	0.792	0.903	4769.56	4860.06	fear.y_bv
89.572	9	0.115	0.779	0.905	4677.793	4786.394	fear.y_bv_onC

56.23	7	0.102	0.904	0.955	4247.935	4338.377	fear.p_bv
59.737	9	0.091	0.902	0.958	4144.291	4252.892	fear.p_bv_onC
49.171	7	0.094	0.882	0.945	4625.723	4716.224	fr.y_bv
58.542	9	0.09	0.861	0.94	4570.282	4678.883	fr.y_bv_onC
38.459	7	0.081	0.939	0.972	4211.236	4301.677	fr.p_bv
41.407	9	0.073	0.936	0.973	4137.742	4246.342	fr.p_bv_onC
43.083	7	0.087	0.91	0.958	5657.713	5748.214	shy.y_bv
50.466	9	0.082	0.896	0.955	5594.838	5703.438	shy.y_bv_onC
15.623	7	0.043	0.986	0.994	4821.939	4912.381	shy.p_bv
19.045	9	0.04	0.984	0.993	4769.894	4878.495	shy.p_bv_onC

Note: The models with the "_bv" identifier attached at the end are unconditional, while the "_bv_onC" models are conditioned on cohort.

Table S8a. Supplemental Slope-Slope and Intercept-Slope Correlations

Parameter	Estimate	SE	PValue	StdAll	Construct	ModelName
Slope_Corr_Adv_Intercept	0.01	0.01	0.542	0.04	ec.y	ec.y_bv_onC
Slope_Corr_Adv_Slope	-0.05	0.01	0.000	-0.50	ec.y	ec.y_bv_onC
Slope_Corr_Adv_Intercept	0.00	0.01	0.834	0.01	ec.p	ec.p_bv_onC
Slope_Corr_Adv_Slope	-0.03	0.01	0.003	-0.31	ec.p	ec.p_bv_onC
Slope_Corr_Adv_Intercept	0.01	0.01	0.326	0.08	ac.y	ac.y_bv_onC
Slope_Corr_Adv_Slope	-0.05	0.02	0.009	-0.35	ac.y	ac.y_bv_onC
Slope_Corr_Adv_Intercept	0.01	0.01	0.581	0.04	ac.p	ac.p_bv_onC
Slope_Corr_Adv_Slope	-0.04	0.01	0.008	-0.33	ac.p	ac.p_bv_onC
Slope_Corr_Adv_Intercept	0.01	0.01	0.258	0.08	at.y	at.y_bv_onC
Slope_Corr_Adv_Slope	-0.06	0.01	0.000	-0.47	at.y	at.y_bv_onC
Slope_Corr_Adv_Intercept	0.00	0.01	0.609	0.04	at.p	at.p_bv_onC
Slope_Corr_Adv_Slope	-0.02	0.01	0.177	-0.19	at.p	at.p_bv_onC
Slope_Corr_Adv_Intercept	-0.01	0.01	0.233	-0.18	ic.y	ic.y_bv_onC
Slope_Corr_Adv_Slope	-0.04	0.02	0.020	-0.59	ic.y	ic.y_bv_onC
Slope_Corr_Adv_Intercept	-0.01	0.01	0.372	-0.06	ic.p	ic.p_bv_onC
Slope_Corr_Adv_Slope	-0.04	0.01	0.005	-0.34	ic.p	ic.p_bv_onC
Slope_Corr_Adv_Intercept	0.00	0.01	0.570	0.04	ne.y	ne.y_bv_onC
Slope_Corr_Adv_Slope	-0.05	0.01	0.000	-0.54	ne.y	ne.y_bv_onC
Slope_Corr_Adv_Intercept	-0.01	0.01	0.194	-0.07	ne.p	ne.p_bv_onC
Slope_Corr_Adv_Slope	-0.02	0.01	0.064	-0.18	ne.p	ne.p_bv_onC
Slope_Corr_Adv_Intercept	0.00	0.01	0.740	0.03	ag.y	ag.y_bv_onC
Slope_Corr_Adv_Slope	-0.05	0.02	0.003	-0.38	ag.y	ag.y_bv_onC
Slope_Corr_Adv_Intercept	0.00	0.01	0.737	0.02	ag.p	ag.p_bv_onC
Slope_Corr_Adv_Slope	-0.04	0.01	0.002	-0.32	ag.p	ag.p_bv_onC
Slope_Corr_Adv_Intercept	0.02	0.01	0.204	0.12	fear.y	fear.y_bv_onC
Slope_Corr_Adv_Slope	-0.06	0.02	0.001	-0.53	fear.y	fear.y_bv_onC

Slope_Corr_Adv_Intercept	0.00	0.01	0.701	-0.04	fear.p	fear.p_bv_onC
Slope_Corr_Adv_Slope	-0.02	0.01	0.254	-0.18	fear.p	fear.p_bv_onC
Slope_Corr_Adv_Intercept	0.01	0.01	0.527	0.05	fr.y	fr.y_bv_onC
Slope_Corr_Adv_Slope	-0.07	0.02	0.000	-0.54	fr.y	fr.y_bv_onC
Slope_Corr_Adv_Intercept	-0.01	0.01	0.417	-0.06	fr.p	fr.p_bv_onC
Slope_Corr_Adv_Slope	-0.04	0.01	0.007	-0.35	fr.p	fr.p_bv_onC
Slope_Corr_Adv_Intercept	-0.01	0.02	0.721	-0.05	shy.y	shy.y_bv_onC
Slope_Corr_Adv_Slope	-0.03	0.02	0.114	-0.35	shy.y	shy.y_bv_onC
Slope_Corr_Adv_Intercept	-0.03	0.01	0.005	-0.22	shy.p	shy.p_bv_onC
Slope_Corr_Adv_Slope	0.03	0.02	0.037	0.27	shy.p	shy.p_bv_onC

Table S8b. Full Output for Youth and Parent Report Models for Bivariate Growth in Facets and Adversity

Parameter	Estimate	SE	PValue	StdAll	Construct	ModelName
ConstructInterceptOfIntercept	3.61	0.03	0.000	8.01	ec.y	ec.y_bv_onC
ConstructVarianceOfIntercept	0.19	0.02	0.000	0.92	ec.y	ec.y_bv_onC
ConstructInterceptOfSlope	0.11	0.04	0.006	0.28	ec.y	ec.y_bv_onC
ConstructVarianceOfSlope	0.14	0.04	0.000	1.00	ec.y	ec.y_bv_onC
AdvInterceptOfIntercept	1.50	0.02	0.000	4.62	ec.y	ec.y_bv_onC
AdvVarianceOfIntercept	0.09	0.01	0.000	0.89	ec.y	ec.y_bv_onC
AdvInterceptOfSlope	-0.03	0.03	0.337	-0.10	ec.y	ec.y_bv_onC
AdvVarianceOfSlope	0.07	0.02	0.001	0.96	ec.y	ec.y_bv_onC
ConstructInterceptOnCohort	-0.16	0.03	0.000	-0.29	ec.y	ec.y_bv_onC
ConstructSlopeOnCohort	-0.02	0.03	0.505	-0.04	ec.y	ec.y_bv_onC
AdvInterceptOnCohort	0.13	0.02	0.000	0.33	ec.y	ec.y_bv_onC
AdvSlopeOnCohort	-0.07	0.02	0.002	-0.21	ec.y	ec.y_bv_onC
Cnsrct.Intercept_Corr_Adv_Intercept	-0.05	0.01	0.000	-0.36	ec.y	ec.y_bv_onC
Cnsrct.Intercept_Corr_Adv_Slope	0.03	0.01	0.002	0.27	ec.y	ec.y_bv_onC
Cnsrct.Slope_Corr_Adv_Intercept	0.01	0.01	0.542	0.04	ec.y	ec.y_bv_onC
Cnsrct.Slope_Corr_Adv_Slope	-0.05	0.01	0.000	-0.50	ec.y	ec.y_bv_onC
ConstructInterceptOfIntercept	3.17	0.03	0.000	6.05	ec.p	ec.p_bv_onC
ConstructVarianceOfIntercept	0.27	0.02	0.000	0.99	ec.p	ec.p_bv_onC
ConstructInterceptOfSlope	0.13	0.03	0.000	0.36	ec.p	ec.p_bv_onC
ConstructVarianceOfSlope	0.14	0.03	0.000	1.00	ec.p	ec.p_bv_onC
AdvInterceptOfIntercept	1.50	0.02	0.000	4.65	ec.p	ec.p_bv_onC
AdvVarianceOfIntercept	0.09	0.01	0.000	0.89	ec.p	ec.p_bv_onC
AdvInterceptOfSlope	-0.02	0.03	0.391	-0.10	ec.p	ec.p_bv_onC
AdvVarianceOfSlope	0.06	0.02	0.002	0.95	ec.p	ec.p_bv_onC
ConstructInterceptOnCohort	0.08	0.03	0.003	0.12	ec.p	ec.p_bv_onC
ConstructSlopeOnCohort	-0.03	0.02	0.255	-0.06	ec.p	ec.p_bv_onC
AdvInterceptOnCohort	0.13	0.02	0.000	0.33	ec.p	ec.p_bv_onC
AdvSlopeOnCohort	-0.07	0.02	0.002	-0.22	ec.p	ec.p_bv_onC
Cnsrct.Intercept_Corr_Adv_Intercept	-0.05	0.01	0.000	-0.30	ec.p	ec.p_bv_onC

Cnsrct.Intercept_Corr_Adv_Slope	0.02	0.01	0.012	0.19	ec.p	ec.p_bv_onC
Cnsrct.Slope_Corr_Adv_Intercept	0.00	0.01	0.834	0.01	ec.p	ec.p_bv_onC
Cnsrct.Slope_Corr_Adv_Slope	-0.03	0.01	0.003	-0.31	ec.p	ec.p_bv_onC
ConstructInterceptOfIntercept	3.68	0.05	0.000	5.49	ac.y	ac.y_bv_onC
ConstructVarianceOfIntercept	0.36	0.05	0.000	0.80	ac.y	ac.y_bv_onC
ConstructInterceptOfSlope	-0.09	0.06	0.118	-0.18	ac.y	ac.y_bv_onC
ConstructVarianceOfSlope	0.26	0.09	0.003	1.00	ac.y	ac.y_bv_onC
AdvInterceptOfIntercept	1.50	0.02	0.000	4.62	ac.y	ac.y_bv_onC
AdvVarianceOfIntercept	0.09	0.01	0.000	0.89	ac.y	ac.y_bv_onC
AdvInterceptOfSlope	-0.02	0.03	0.388	-0.09	ac.y	ac.y_bv_onC
AdvVarianceOfSlope	0.07	0.02	0.001	0.95	ac.y	ac.y_bv_onC
ConstructInterceptOnCohort	-0.38	0.04	0.000	-0.45	ac.y	ac.y_bv_onC
ConstructSlopeOnCohort	-0.04	0.04	0.331	-0.07	ac.y	ac.y_bv_onC
AdvInterceptOnCohort	0.13	0.02	0.000	0.33	ac.y	ac.y_bv_onC
AdvSlopeOnCohort	-0.07	0.02	0.002	-0.21	ac.y	ac.y_bv_onC
Cnsrct.Intercept_Corr_Adv_Intercept	-0.06	0.01	0.000	-0.32	ac.y	ac.y_bv_onC
Cnsrct.Intercept_Corr_Adv_Slope	0.03	0.01	0.023	0.20	ac.y	ac.y_bv_onC
Cnsrct.Slope_Corr_Adv_Intercept	0.01	0.01	0.326	0.08	ac.y	ac.y_bv_onC
Cnsrct.Slope_Corr_Adv_Slope	-0.05	0.02	0.009	-0.35	ac.y	ac.y_bv_onC
ConstructInterceptOfIntercept	3.15	0.05	0.000	4.55	ac.p	ac.p_bv_onC
ConstructVarianceOfIntercept	0.48	0.04	0.000	1.00	ac.p	ac.p_bv_onC
ConstructInterceptOfSlope	0.01	0.05	0.875	0.02	ac.p	ac.p_bv_onC
ConstructVarianceOfSlope	0.21	0.06	0.001	1.00	ac.p	ac.p_bv_onC
AdvInterceptOfIntercept	1.50	0.02	0.000	4.66	ac.p	ac.p_bv_onC
AdvVarianceOfIntercept	0.09	0.01	0.000	0.89	ac.p	ac.p_bv_onC
AdvInterceptOfSlope	-0.03	0.03	0.377	-0.10	ac.p	ac.p_bv_onC
AdvVarianceOfSlope	0.06	0.02	0.003	0.95	ac.p	ac.p_bv_onC
ConstructInterceptOnCohort	-0.02	0.04	0.578	-0.02	ac.p	ac.p_bv_onC
ConstructSlopeOnCohort	0.00	0.04	0.961	0.00	ac.p	ac.p_bv_onC
AdvInterceptOnCohort	0.13	0.02	0.000	0.33	ac.p	ac.p_bv_onC
AdvSlopeOnCohort	-0.07	0.02	0.002	-0.22	ac.p	ac.p_bv_onC
Cnsrct.Intercept_Corr_Adv_Intercept	-0.06	0.01	0.000	-0.29	ac.p	ac.p_bv_onC
Cnsrct.Intercept_Corr_Adv_Slope	0.03	0.01	0.040	0.16	ac.p	ac.p_bv_onC

Cnsrct.Slope_Corr_Adv_Intercept	0.01	0.01	0.581	0.04	ac.p	ac.p_bv_onC
Cnsrct.Slope_Corr_Adv_Slope	-0.04	0.01	0.008	-0.33	ac.p	ac.p_bv_onC
ConstructInterceptOfIntercept	3.48	0.04	0.000	7.71	at.y	at.y_bv_onC
ConstructVarianceOfIntercept	0.20	0.03	0.000	0.98	at.y	at.y_bv_onC
ConstructInterceptOfSlope	0.15	0.05	0.003	0.29	at.y	at.y_bv_onC
ConstructVarianceOfSlope	0.27	0.06	0.000	1.00	at.y	at.y_bv_onC
AdvInterceptOfIntercept	1.50	0.02	0.000	4.63	at.y	at.y_bv_onC
AdvVarianceOfIntercept	0.09	0.01	0.000	0.89	at.y	at.y_bv_onC
AdvInterceptOfSlope	-0.03	0.03	0.322	-0.11	at.y	at.y_bv_onC
AdvVarianceOfSlope	0.06	0.02	0.002	0.96	at.y	at.y_bv_onC
ConstructInterceptOnCohort	-0.07	0.03	0.013	-0.13	at.y	at.y_bv_onC
ConstructSlopeOnCohort	-0.02	0.04	0.598	-0.03	at.y	at.y_bv_onC
AdvInterceptOnCohort	0.13	0.02	0.000	0.33	at.y	at.y_bv_onC
AdvSlopeOnCohort	-0.07	0.02	0.002	-0.21	at.y	at.y_bv_onC
Cnsrct.Intercept_Corr_Adv_Intercept	-0.05	0.01	0.000	-0.39	at.y	at.y_bv_onC
Cnsrct.Intercept_Corr_Adv_Slope	0.04	0.01	0.001	0.31	at.y	at.y_bv_onC
Cnsrct.Slope_Corr_Adv_Intercept	0.01	0.01	0.258	0.08	at.y	at.y_bv_onC
Cnsrct.Slope_Corr_Adv_Slope	-0.06	0.01	0.000	-0.47	at.y	at.y_bv_onC
ConstructInterceptOfIntercept	2.91	0.04	0.000	5.62	at.p	at.p_bv_onC
ConstructVarianceOfIntercept	0.25	0.02	0.000	0.95	at.p	at.p_bv_onC
ConstructInterceptOfSlope	0.14	0.04	0.000	0.45	at.p	at.p_bv_onC
ConstructVarianceOfSlope	0.10	0.04	0.021	1.00	at.p	at.p_bv_onC
AdvInterceptOfIntercept	1.50	0.02	0.000	4.65	at.p	at.p_bv_onC
AdvVarianceOfIntercept	0.09	0.01	0.000	0.89	at.p	at.p_bv_onC
AdvInterceptOfSlope	-0.02	0.03	0.386	-0.10	at.p	at.p_bv_onC
AdvVarianceOfSlope	0.06	0.02	0.002	0.95	at.p	at.p_bv_onC
ConstructInterceptOnCohort	0.15	0.03	0.000	0.23	at.p	at.p_bv_onC
ConstructSlopeOnCohort	-0.02	0.03	0.409	-0.06	at.p	at.p_bv_onC
AdvInterceptOnCohort	0.13	0.02	0.000	0.33	at.p	at.p_bv_onC
AdvSlopeOnCohort	-0.07	0.02	0.002	-0.22	at.p	at.p_bv_onC
Cnsrct.Intercept_Corr_Adv_Intercept	-0.04	0.01	0.000	-0.29	at.p	at.p_bv_onC
Cnsrct.Intercept_Corr_Adv_Slope	0.02	0.01	0.084	0.14	at.p	at.p_bv_onC
Cnsrct.Slope_Corr_Adv_Intercept	0.00	0.01	0.609	0.04	at.p	at.p_bv_onC

Cnsrct.Slope_Corr_Adv_Slope	-0.02	0.01	0.177	-0.19	at.p	at.p_bv_onC
ConstructInterceptOfIntercept	3.72	0.04	0.000	9.02	ic.y	ic.y_bv_onC
ConstructVarianceOfIntercept	0.17	0.03	0.000	0.99	ic.y	ic.y_bv_onC
ConstructInterceptOfSlope	0.26	0.05	0.000	1.12	ic.y	ic.y_bv_onC
ConstructVarianceOfSlope	0.06	0.06	0.365	1.00	ic.y	ic.y_bv_onC
AdvInterceptOfIntercept	1.50	0.02	0.000	4.64	ic.y	ic.y_bv_onC
AdvVarianceOfIntercept	0.09	0.01	0.000	0.89	ic.y	ic.y_bv_onC
AdvInterceptOfSlope	-0.03	0.03	0.337	-0.11	ic.y	ic.y_bv_onC
AdvVarianceOfSlope	0.06	0.02	0.002	0.96	ic.y	ic.y_bv_onC
ConstructInterceptOnCohort	-0.06	0.03	0.030	-0.12	ic.y	ic.y_bv_onC
ConstructSlopeOnCohort	0.00	0.04	0.935	-0.01	ic.y	ic.y_bv_onC
AdvInterceptOnCohort	0.13	0.02	0.000	0.33	ic.y	ic.y_bv_onC
AdvSlopeOnCohort	-0.07	0.02	0.002	-0.21	ic.y	ic.y_bv_onC
Cnsrct.Intercept_Corr_Adv_Intercept	-0.03	0.01	0.000	-0.24	ic.y	ic.y_bv_onC
Cnsrct.Intercept_Corr_Adv_Slope	0.02	0.01	0.063	0.19	ic.y	ic.y_bv_onC
Cnsrct.Slope_Corr_Adv_Intercept	-0.01	0.01	0.233	-0.18	ic.y	ic.y_bv_onC
Cnsrct.Slope_Corr_Adv_Slope	-0.04	0.02	0.020	-0.59	ic.y	ic.y_bv_onC
ConstructInterceptOfIntercept	3.51	0.04	0.000	7.15	ic.p	ic.p_bv_onC
ConstructVarianceOfIntercept	0.23	0.02	0.000	0.95	ic.p	ic.p_bv_onC
ConstructInterceptOfSlope	0.31	0.04	0.000	0.75	ic.p	ic.p_bv_onC
ConstructVarianceOfSlope	0.17	0.04	0.000	0.98	ic.p	ic.p_bv_onC
AdvInterceptOfIntercept	1.50	0.02	0.000	4.63	ic.p	ic.p_bv_onC
AdvVarianceOfIntercept	0.09	0.01	0.000	0.89	ic.p	ic.p_bv_onC
AdvInterceptOfSlope	-0.03	0.03	0.367	-0.10	ic.p	ic.p_bv_onC
AdvVarianceOfSlope	0.06	0.02	0.001	0.95	ic.p	ic.p_bv_onC
ConstructInterceptOnCohort	0.14	0.03	0.000	0.23	ic.p	ic.p_bv_onC
ConstructSlopeOnCohort	-0.07	0.03	0.032	-0.13	ic.p	ic.p_bv_onC
AdvInterceptOnCohort	0.13	0.02	0.000	0.33	ic.p	ic.p_bv_onC
AdvSlopeOnCohort	-0.07	0.02	0.002	-0.21	ic.p	ic.p_bv_onC
Cnsrct.Intercept_Corr_Adv_Intercept	-0.04	0.01	0.000	-0.24	ic.p	ic.p_bv_onC
Cnsrct.Intercept_Corr_Adv_Slope	0.03	0.01	0.003	0.24	ic.p	ic.p_bv_onC
Cnsrct.Slope_Corr_Adv_Intercept	-0.01	0.01	0.372	-0.06	ic.p	ic.p_bv_onC
Cnsrct.Slope_Corr_Adv_Slope	-0.04	0.01	0.005	-0.34	ic.p	ic.p_bv_onC

ConstructInterceptOfIntercept	3.38	0.03	0.000	8.55	ne.y	ne.y_bv_onC
ConstructVarianceOfIntercept	0.16	0.02	0.000	1.00	ne.y	ne.y_bv_onC
ConstructInterceptOfSlope	0.35	0.04	0.000	0.91	ne.y	ne.y_bv_onC
ConstructVarianceOfSlope	0.15	0.04	0.000	0.98	ne.y	ne.y_bv_onC
AdvInterceptOfIntercept	1.50	0.02	0.000	4.64	ne.y	ne.y_bv_onC
AdvVarianceOfIntercept	0.09	0.01	0.000	0.89	ne.y	ne.y_bv_onC
AdvInterceptOfSlope	-0.03	0.03	0.360	-0.10	ne.y	ne.y_bv_onC
AdvVarianceOfSlope	0.06	0.02	0.001	0.96	ne.y	ne.y_bv_onC
ConstructInterceptOnCohort	0.01	0.03	0.841	0.01	ne.y	ne.y_bv_onC
ConstructSlopeOnCohort	-0.07	0.03	0.018	-0.15	ne.y	ne.y_bv_onC
AdvInterceptOnCohort	0.13	0.02	0.000	0.33	ne.y	ne.y_bv_onC
AdvSlopeOnCohort	-0.07	0.02	0.002	-0.21	ne.y	ne.y_bv_onC
Cnsrct.Intercept_Corr_Adv_Intercept	-0.03	0.01	0.000	-0.29	ne.y	ne.y_bv_onC
Cnsrct.Intercept_Corr_Adv_Slope	0.03	0.01	0.001	0.29	ne.y	ne.y_bv_onC
Cnsrct.Slope_Corr_Adv_Intercept	0.00	0.01	0.570	0.04	ne.y	ne.y_bv_onC
Cnsrct.Slope_Corr_Adv_Slope	-0.05	0.01	0.000	-0.54	ne.y	ne.y_bv_onC
ConstructInterceptOfIntercept	3.34	0.03	0.000	7.36	ne.p	ne.p_bv_onC
ConstructVarianceOfIntercept	0.20	0.02	0.000	0.97	ne.p	ne.p_bv_onC
ConstructInterceptOfSlope	0.22	0.03	0.000	0.58	ne.p	ne.p_bv_onC
ConstructVarianceOfSlope	0.14	0.03	0.000	1.00	ne.p	ne.p_bv_onC
AdvInterceptOfIntercept	1.50	0.02	0.000	4.64	ne.p	ne.p_bv_onC
AdvVarianceOfIntercept	0.09	0.01	0.000	0.89	ne.p	ne.p_bv_onC
AdvInterceptOfSlope	-0.02	0.03	0.392	-0.09	ne.p	ne.p_bv_onC
AdvVarianceOfSlope	0.07	0.02	0.001	0.96	ne.p	ne.p_bv_onC
ConstructInterceptOnCohort	0.10	0.02	0.000	0.17	ne.p	ne.p_bv_onC
ConstructSlopeOnCohort	0.02	0.02	0.438	0.04	ne.p	ne.p_bv_onC
AdvInterceptOnCohort	0.13	0.02	0.000	0.33	ne.p	ne.p_bv_onC
AdvSlopeOnCohort	-0.07	0.02	0.002	-0.21	ne.p	ne.p_bv_onC
Cnsrct.Intercept_Corr_Adv_Intercept	-0.02	0.01	0.000	-0.18	ne.p	ne.p_bv_onC
Cnsrct.Intercept_Corr_Adv_Slope	0.01	0.01	0.340	0.07	ne.p	ne.p_bv_onC
Cnsrct.Slope_Corr_Adv_Intercept	-0.01	0.01	0.194	-0.07	ne.p	ne.p_bv_onC
Cnsrct.Slope_Corr_Adv_Slope	-0.02	0.01	0.064	-0.18	ne.p	ne.p_bv_onC
ConstructInterceptOfIntercept	4.25	0.04	0.000	7.15	ag.y	ag.y_bv_onC

ConstructVarianceOfIntercept	0.33	0.04	0.000	0.93	ag.y	ag.y_bv_onC
ConstructInterceptOfSlope	0.01	0.05	0.858	0.02	ag.y	ag.y_bv_onC
ConstructVarianceOfSlope	0.26	0.07	0.000	0.99	ag.y	ag.y_bv_onC
AdvInterceptOfIntercept	1.50	0.02	0.000	4.64	ag.y	ag.y_bv_onC
AdvVarianceOfIntercept	0.09	0.01	0.000	0.90	ag.y	ag.y_bv_onC
AdvInterceptOfSlope	-0.03	0.03	0.362	-0.10	ag.y	ag.y_bv_onC
AdvVarianceOfSlope	0.07	0.02	0.001	0.96	ag.y	ag.y_bv_onC
ConstructInterceptOnCohort	-0.20	0.03	0.000	-0.27	ag.y	ag.y_bv_onC
ConstructSlopeOnCohort	0.05	0.04	0.231	0.08	ag.y	ag.y_bv_onC
AdvInterceptOnCohort	0.13	0.02	0.000	0.32	ag.y	ag.y_bv_onC
AdvSlopeOnCohort	-0.07	0.02	0.002	-0.21	ag.y	ag.y_bv_onC
Cnsrct.Intercept_Corr_Adv_Intercept	-0.05	0.01	0.000	-0.31	ag.y	ag.y_bv_onC
Cnsrct.Intercept_Corr_Adv_Slope	0.02	0.01	0.076	0.16	ag.y	ag.y_bv_onC
Cnsrct.Slope_Corr_Adv_Intercept	0.00	0.01	0.740	0.03	ag.y	ag.y_bv_onC
Cnsrct.Slope_Corr_Adv_Slope	-0.05	0.02	0.003	-0.38	ag.y	ag.y_bv_onC
ConstructInterceptOfIntercept	3.59	0.04	0.000	5.68	ag.p	ag.p_bv_onC
ConstructVarianceOfIntercept	0.40	0.03	0.000	1.00	ag.p	ag.p_bv_onC
ConstructInterceptOfSlope	0.18	0.04	0.000	0.39	ag.p	ag.p_bv_onC
ConstructVarianceOfSlope	0.21	0.05	0.000	1.00	ag.p	ag.p_bv_onC
AdvInterceptOfIntercept	1.50	0.02	0.000	4.63	ag.p	ag.p_bv_onC
AdvVarianceOfIntercept	0.09	0.01	0.000	0.89	ag.p	ag.p_bv_onC
AdvInterceptOfSlope	-0.02	0.03	0.418	-0.09	ag.p	ag.p_bv_onC
AdvVarianceOfSlope	0.07	0.02	0.001	0.95	ag.p	ag.p_bv_onC
ConstructInterceptOnCohort	0.06	0.03	0.092	0.07	ag.p	ag.p_bv_onC
ConstructSlopeOnCohort	0.03	0.03	0.341	0.05	ag.p	ag.p_bv_onC
AdvInterceptOnCohort	0.13	0.02	0.000	0.33	ag.p	ag.p_bv_onC
AdvSlopeOnCohort	-0.07	0.02	0.002	-0.21	ag.p	ag.p_bv_onC
Cnsrct.Intercept_Corr_Adv_Intercept	-0.05	0.01	0.000	-0.27	ag.p	ag.p_bv_onC
Cnsrct.Intercept_Corr_Adv_Slope	0.02	0.01	0.209	0.10	ag.p	ag.p_bv_onC
Cnsrct.Slope_Corr_Adv_Intercept	0.00	0.01	0.737	0.02	ag.p	ag.p_bv_onC
Cnsrct.Slope_Corr_Adv_Slope	-0.04	0.01	0.002	-0.32	ag.p	ag.p_bv_onC
ConstructInterceptOfIntercept	3.04	0.05	0.000	5.33	fear.y	fear.y_bv_onC
ConstructVarianceOfIntercept	0.29	0.05	0.000	0.91	fear.y	fear.y_bv_onC

ConstructInterceptOfSlope	0.82	0.06	0.000	1.87	fear.y	fear.y_bv_onC
ConstructVarianceOfSlope	0.18	0.09	0.036	0.93	fear.y	fear.y_bv_onC
AdvInterceptOfIntercept	1.50	0.02	0.000	4.66	fear.y	fear.y_bv_onC
AdvVarianceOfIntercept	0.09	0.01	0.000	0.89	fear.y	fear.y_bv_onC
AdvInterceptOfSlope	-0.03	0.03	0.334	-0.11	fear.y	fear.y_bv_onC
AdvVarianceOfSlope	0.06	0.02	0.003	0.96	fear.y	fear.y_bv_onC
ConstructInterceptOnCohort	0.22	0.04	0.000	0.31	fear.y	fear.y_bv_onC
ConstructSlopeOnCohort	-0.14	0.04	0.001	-0.26	fear.y	fear.y_bv_onC
AdvInterceptOnCohort	0.13	0.02	0.000	0.33	fear.y	fear.y_bv_onC
AdvSlopeOnCohort	-0.07	0.02	0.002	-0.21	fear.y	fear.y_bv_onC
Cnsrct.Intercept_Corr_Adv_Intercept	-0.03	0.01	0.008	-0.17	fear.y	fear.y_bv_onC
Cnsrct.Intercept_Corr_Adv_Slope	0.04	0.01	0.005	0.28	fear.y	fear.y_bv_onC
Cnsrct.Slope_Corr_Adv_Intercept	0.02	0.01	0.204	0.12	fear.y	fear.y_bv_onC
Cnsrct.Slope_Corr_Adv_Slope	-0.06	0.02	0.001	-0.53	fear.y	fear.y_bv_onC
ConstructInterceptOfIntercept	3.34	0.04	0.000	5.73	fear.p	fear.p_bv_onC
ConstructVarianceOfIntercept	0.31	0.03	0.000	0.92	fear.p	fear.p_bv_onC
ConstructInterceptOfSlope	0.46	0.04	0.000	1.34	fear.p	fear.p_bv_onC
ConstructVarianceOfSlope	0.12	0.06	0.052	1.00	fear.p	fear.p_bv_onC
AdvInterceptOfIntercept	1.50	0.02	0.000	4.64	fear.p	fear.p_bv_onC
AdvVarianceOfIntercept	0.09	0.01	0.000	0.89	fear.p	fear.p_bv_onC
AdvInterceptOfSlope	-0.03	0.03	0.355	-0.10	fear.p	fear.p_bv_onC
AdvVarianceOfSlope	0.06	0.02	0.002	0.96	fear.p	fear.p_bv_onC
ConstructInterceptOnCohort	0.21	0.03	0.000	0.28	fear.p	fear.p_bv_onC
ConstructSlopeOnCohort	-0.02	0.03	0.502	-0.05	fear.p	fear.p_bv_onC
AdvInterceptOnCohort	0.13	0.02	0.000	0.33	fear.p	fear.p_bv_onC
AdvSlopeOnCohort	-0.07	0.02	0.002	-0.21	fear.p	fear.p_bv_onC
Cnsrct.Intercept_Corr_Adv_Intercept	-0.02	0.01	0.020	-0.13	fear.p	fear.p_bv_onC
Cnsrct.Intercept_Corr_Adv_Slope	0.00	0.01	0.705	0.03	fear.p	fear.p_bv_onC
Cnsrct.Slope_Corr_Adv_Intercept	0.00	0.01	0.701	-0.04	fear.p	fear.p_bv_onC
Cnsrct.Slope_Corr_Adv_Slope	-0.02	0.01	0.254	-0.18	fear.p	fear.p_bv_onC
ConstructInterceptOfIntercept	2.92	0.04	0.000	5.56	fr.y	fr.y_bv_onC
ConstructVarianceOfIntercept	0.27	0.04	0.000	0.99	fr.y	fr.y_bv_onC
ConstructInterceptOfSlope	0.29	0.06	0.000	0.59	fr.y	fr.y_bv_onC

ConstructVarianceOfSlope	0.24	0.08	0.003	0.99	fr.y	fr.y_bv_onC
AdvInterceptOfIntercept	1.50	0.02	0.000	4.60	fr.y	fr.y_bv_onC
AdvVarianceOfIntercept	0.09	0.01	0.000	0.89	fr.y	fr.y_bv_onC
AdvInterceptOfSlope	-0.03	0.03	0.360	-0.10	fr.y	fr.y_bv_onC
AdvVarianceOfSlope	0.07	0.02	0.001	0.96	fr.y	fr.y_bv_onC
ConstructInterceptOnCohort	-0.05	0.03	0.105	-0.08	fr.y	fr.y_bv_onC
ConstructSlopeOnCohort	-0.06	0.04	0.165	-0.10	fr.y	fr.y_bv_onC
AdvInterceptOnCohort	0.13	0.02	0.000	0.32	fr.y	fr.y_bv_onC
AdvSlopeOnCohort	-0.07	0.02	0.002	-0.21	fr.y	fr.y_bv_onC
Cnsrct.Intercept_Corr_Adv_Intercept	-0.04	0.01	0.000	-0.25	fr.y	fr.y_bv_onC
Cnsrct.Intercept_Corr_Adv_Slope	0.04	0.01	0.003	0.27	fr.y	fr.y_bv_onC
Cnsrct.Slope_Corr_Adv_Intercept	0.01	0.01	0.527	0.05	fr.y	fr.y_bv_onC
Cnsrct.Slope_Corr_Adv_Slope	-0.07	0.02	0.000	-0.54	fr.y	fr.y_bv_onC
ConstructInterceptOfIntercept	3.05	0.04	0.000	5.27	fr.p	fr.p_bv_onC
ConstructVarianceOfIntercept	0.33	0.03	0.000	1.00	fr.p	fr.p_bv_onC
ConstructInterceptOfSlope	0.14	0.04	0.001	0.34	fr.p	fr.p_bv_onC
ConstructVarianceOfSlope	0.17	0.06	0.004	0.98	fr.p	fr.p_bv_onC
AdvInterceptOfIntercept	1.50	0.02	0.000	4.65	fr.p	fr.p_bv_onC
AdvVarianceOfIntercept	0.09	0.01	0.000	0.89	fr.p	fr.p_bv_onC
AdvInterceptOfSlope	-0.03	0.03	0.376	-0.10	fr.p	fr.p_bv_onC
AdvVarianceOfSlope	0.06	0.02	0.001	0.96	fr.p	fr.p_bv_onC
ConstructInterceptOnCohort	0.04	0.03	0.162	0.06	fr.p	fr.p_bv_onC
ConstructSlopeOnCohort	0.08	0.03	0.016	0.16	fr.p	fr.p_bv_onC
AdvInterceptOnCohort	0.13	0.02	0.000	0.33	fr.p	fr.p_bv_onC
AdvSlopeOnCohort	-0.07	0.02	0.002	-0.21	fr.p	fr.p_bv_onC
Cnsrct.Intercept_Corr_Adv_Intercept	-0.04	0.01	0.000	-0.22	fr.p	fr.p_bv_onC
Cnsrct.Intercept_Corr_Adv_Slope	0.02	0.01	0.076	0.14	fr.p	fr.p_bv_onC
Cnsrct.Slope_Corr_Adv_Intercept	-0.01	0.01	0.417	-0.06	fr.p	fr.p_bv_onC
Cnsrct.Slope_Corr_Adv_Slope	-0.04	0.01	0.007	-0.35	fr.p	fr.p_bv_onC
ConstructInterceptOfIntercept	3.41	0.06	0.000	5.15	shy.y	shy.y_bv_onC
ConstructVarianceOfIntercept	0.43	0.07	0.000	0.99	shy.y	shy.y_bv_onC
ConstructInterceptOfSlope	0.34	0.07	0.000	0.81	shy.y	shy.y_bv_onC
ConstructVarianceOfSlope	0.14	0.14	0.304	0.84	shy.y	shy.y_bv_onC

AdvInterceptOfIntercept	1.50	0.02	0.000	4.64	shy.y	shy.y_bv_onC
AdvVarianceOfIntercept	0.09	0.01	0.000	0.89	shy.y	shy.y_bv_onC
AdvInterceptOfSlope	-0.03	0.03	0.373	-0.10	shy.y	shy.y_bv_onC
AdvVarianceOfSlope	0.06	0.02	0.002	0.95	shy.y	shy.y_bv_onC
ConstructInterceptOnCohort	0.10	0.05	0.033	0.12	shy.y	shy.y_bv_onC
ConstructSlopeOnCohort	-0.21	0.06	0.000	-0.41	shy.y	shy.y_bv_onC
AdvInterceptOnCohort	0.13	0.02	0.000	0.33	shy.y	shy.y_bv_onC
AdvSlopeOnCohort	-0.07	0.02	0.002	-0.21	shy.y	shy.y_bv_onC
Cnsrct.Intercept_Corr_Adv_Intercept	-0.01	0.01	0.363	-0.06	shy.y	shy.y_bv_onC
Cnsrct.Intercept_Corr_Adv_Slope	0.02	0.02	0.342	0.10	shy.y	shy.y_bv_onC
Cnsrct.Slope_Corr_Adv_Intercept	-0.01	0.02	0.721	-0.05	shy.y	shy.y_bv_onC
Cnsrct.Slope_Corr_Adv_Slope	-0.03	0.02	0.114	-0.35	shy.y	shy.y_bv_onC
ConstructInterceptOfIntercept	3.42	0.05	0.000	4.38	shy.p	shy.p_bv_onC
ConstructVarianceOfIntercept	0.60	0.05	0.000	0.99	shy.p	shy.p_bv_onC
ConstructInterceptOfSlope	0.11	0.05	0.031	0.23	shy.p	shy.p_bv_onC
ConstructVarianceOfSlope	0.21	0.08	0.010	1.00	shy.p	shy.p_bv_onC
AdvInterceptOfIntercept	1.50	0.02	0.000	4.60	shy.p	shy.p_bv_onC
AdvVarianceOfIntercept	0.10	0.01	0.000	0.90	shy.p	shy.p_bv_onC
AdvInterceptOfSlope	-0.03	0.03	0.343	-0.10	shy.p	shy.p_bv_onC
AdvVarianceOfSlope	0.07	0.02	0.001	0.96	shy.p	shy.p_bv_onC
ConstructInterceptOnCohort	0.07	0.04	0.078	0.08	shy.p	shy.p_bv_onC
ConstructSlopeOnCohort	-0.03	0.04	0.489	-0.05	shy.p	shy.p_bv_onC
AdvInterceptOnCohort	0.13	0.02	0.000	0.32	shy.p	shy.p_bv_onC
AdvSlopeOnCohort	-0.07	0.02	0.002	-0.20	shy.p	shy.p_bv_onC
Cnsrct.Intercept_Corr_Adv_Intercept	0.02	0.01	0.088	0.09	shy.p	shy.p_bv_onC
Cnsrct.Intercept_Corr_Adv_Slope	-0.02	0.02	0.310	-0.08	shy.p	shy.p_bv_onC
Cnsrct.Slope_Corr_Adv_Intercept	-0.03	0.01	0.005	-0.22	shy.p	shy.p_bv_onC
Cnsrct.Slope_Corr_Adv_Slope	0.03	0.02	0.037	0.27	shy.p	shy.p_bv_onC

Table S9. Fit Indices for Univariate Latent Growth Curve Models for Effortful Control and Emotional Stability Conditioned on Cohort and Adversity Factor Scores

chisq	df	rmsea	tli	cfi	ModID
34.955	5	0.094	0.896	0.957	ec.y_uv.onS_ADVandC
30.533	5	0.087	0.947	0.978	ec.p_uv.onS_ADVandC
11.436	5	0.043	0.978	0.991	ac.y_uv.onS_ADVandC
23.721	5	0.074	0.954	0.981	ac.p_uv.onS_ADVandC
25.659	5	0.078	0.878	0.949	at.y_uv.onS_ADVandC
8.809	5	0.033	0.988	0.995	at.p_uv.onS_ADVandC
24.643	5	0.076	0.859	0.941	ic.y_uv.onS_ADVandC
23.191	5	0.073	0.93	0.971	ic.p_uv.onS_ADVandC
94.998	5	0.162	0.466	0.777	ne.y_uv.onS_ADVandC
45.353	5	0.109	0.898	0.957	ne.p_uv.onS_ADVandC
24.371	5	0.075	0.912	0.963	ag.y_uv.onS_ADVandC
22.466	5	0.072	0.95	0.979	ag.p_uv.onS_ADVandC
84.967	5	0.153	0.454	0.772	fear.y_uv.onS_ADVandC
42.525	5	0.105	0.872	0.947	fear.p_uv.onS_ADVandC
49.4	5	0.114	0.675	0.865	fr.y_uv.onS_ADVandC
23.399	5	0.073	0.935	0.973	fr.p_uv.onS_ADVandC
40.66	5	0.102	0.801	0.917	shy.y_uv.onS_ADVandC
3.655	5	0	1.004	1	shy.p_uv.onS_ADVandC

Table S10. Youth and Parent Report Estimates for Prevalence of Growth Despite Adversity (Estimates for Univariate Model Conditioned on Initial Adversity, Change in Adversity, and Cohort)

	Low Adversity		% Change >/= SESOI despite baseline Adversity		% Change >/= SESOI despite increases in Adversity	
Model	Decrease	Increase	Decrease	Increase	Decrease	Increase
ec.y_bv	17% (83/488)	48% (236/488)	17% (16/93)	40% (37/93)	38% (24/63)	19% (12/63)
ec.p_bv	19% (95/488)	49% (239/488)	20% (19/93)	47% (44/93)	29% (18/63)	38% (24/63)
ac.y_bv	45% (222/488)	16% (76/488)	38% (35/93)	15% (14/93)	65% (41/63)	5% (3/63)
ac.p_bv	25% (121/488)	30% (146/488)	25% (23/93)	25% (23/93)	43% (27/63)	19% (12/63)
at.y_bv	21% (101/488)	51% (251/488)	11% (10/93)	55% (51/93)	40% (25/63)	32% (20/63)
at.p_bv	8% (40/488)	49% (241/488)	4% (4/93)	57% (53/93)	10% (6/63)	43% (27/63)
ic.y_bv	0% (0/488)	96% (468/488)	0% (0/93)	72% (67/93)	5% (3/63)	73% (46/63)
ic.p_bv	8% (40/488)	74% (360/488)	9% (8/93)	57% (53/93)	16% (10/63)	44% (28/63)
ne.y_bv	3% (16/488)	81% (397/488)	5% (5/93)	76% (71/93)	19% (12/63)	49% (31/63)
ne.p_bv	11% (54/488)	72% (353/488)	13% (12/93)	66% (61/93)	16% (10/63)	54% (34/63)
ag.y_bv	18% (88/488)	40% (194/488)	23% (21/93)	44% (41/93)	46% (29/63)	16% (10/63)
ag.p_bv	14% (66/488)	61% (299/488)	12% (11/93)	67% (62/93)	30% (19/63)	35% (22/63)
fear.y_bv	0% (0/488)	99% (484/488)	0% (0/93)	100% (93/93)	0% (0/63)	95% (60/63)
fear.p_bv	0% (1/488)	96% (467/488)	0% (0/93)	95% (88/93)	0% (0/63)	89% (56/63)
fr.y_bv	5% (25/488)	67% (327/488)	10% (9/93)	65% (60/93)	25% (16/63)	37% (23/63)
fr.p_bv	5% (24/488)	69% (339/488)	5% (5/93)	58% (54/93)	21% (13/63)	40% (25/63)
shy.y_bv	7% (34/488)	39% (192/488)	16% (15/93)	17% (16/93)	13% (8/63)	33% (21/63)
shy.p_bv	14% (69/488)	35% (171/488)	31% (29/93)	12% (11/93)	8% (5/63)	46% (29/63)

Table S11. Fit indices for all Research Question 04 models

chisq	df	rmsea	tli	cfi	ModID
0	0	0	1	1	Univariate PSS
0	0	0	1	1	Univariate ProSoc
0	0	0	1	1	Univariate Self Esteem
75.555	21	0.062	0.939	0.971	associative_ec.p_anx.y
97.385	21	0.073	0.905	0.956	associative_ne.p_anx.y
63.028	21	0.054	0.958	0.98	associative_ec.p_avoid.y
91.301	21	0.07	0.923	0.964	associative_ne.p_avoid.y
54.66	13	0.069	0.936	0.977	associative_ec.p_PSS.y
84.802	13	0.09	0.878	0.956	associative_ne.p_PSS.y
46.414	13	0.061	0.946	0.98	associative_ec.p_ProSoc.y
76.193	13	0.084	0.885	0.958	associative_ne.p_ProSoc.y
42.827	13	0.058	0.955	0.984	associative_ec.p_SEQ.y
76.038	13	0.084	0.894	0.962	associative_ne.p_SEQ.y
140.886	30	0.074	0.913	0.942	regression_ec.p_anx.y
161.443	30	0.08	0.886	0.924	regression_ne.p_anx.y
124.015	30	0.068	0.934	0.956	regression_ec.p_avoid.y
150.334	30	0.077	0.907	0.938	regression_ne.p_avoid.y
241.594	22	0.121	0.8	0.878	regression_ec.p_PSS.y
268.517	22	0.128	0.752	0.848	regression_ne.p_PSS.y
329.392	22	0.143	0.705	0.82	regression_ec.p_ProSoc.y
353.156	22	0.149	0.643	0.782	regression_ne.p_ProSoc.y
283.227	22	0.132	0.766	0.857	regression_ec.p_SEQ.y
315.679	22	0.14	0.709	0.822	regression_ne.p_SEQ.y

Table S12. Univariate Latent Difference Score Models for Parenting and Prosocial

Construct	Parameter	Estimate	SE	PValue
Parenting Style	Level	0.85	0.01	0.000
	Level Variance	0.02	0.00	0.000
	Slope	0.03	0.01	0.000
	Slope Variance	0.03	0.00	0.000
Prosocial	Level	2.62	0.04	0.000
	Level Variance	0.99	0.06	0.000
	Slope	-0.04	0.05	0.434
	Slope Variance	1.21	0.08	0.000
Self Esteem	Level	1.70	0.02	0.000
	Level Variance	0.23	0.01	0.000
	Slope	-0.11	0.02	0.000
	Slope Variance	0.29	0.02	0.000

Table S13. Associations between Personality, Adversity, and Third Variable

Parameter	*r*	*p*-value	Personality	Factor
I_Temperment_Corr_S_Temperment	-0.21	0.041	ec.p	anx.y
I_Temperment_Corr_I_Adv	-0.31	0.000	ec.p	anx.y
I_Temperment_Corr_S_Adv	0.19	0.013	ec.p	anx.y
I_Temperment_Corr_I_Factor	-0.30	0.000	ec.p	anx.y
I_Temperment_Corr_S_Factor	-0.07	0.433	ec.p	anx.y
S_Temperment_Corr_I_Adv	0.01	0.840	ec.p	anx.y
S_Temperment_Corr_S_Adv	-0.33	0.003	ec.p	anx.y
S_Temperment_Corr_I_Factor	0.15	0.116	ec.p	anx.y
**S_Temperment_Corr_S_Factor**	**-0.24**	**0.028**	**ec.p**	**anx.y**
I_Adv_Corr_S_Adv	-0.38	0.018	ec.p	anx.y
I_Adv_Corr_I_Factor	0.44	0.000	ec.p	anx.y
I_Adv_Corr_S_Factor	-0.20	0.032	ec.p	anx.y
S_Adv_Corr_I_Factor	-0.32	0.009	ec.p	anx.y
S_Adv_Corr_S_Factor	0.51	0.001	ec.p	anx.y
I_Factor_Corr_S_Factor	-0.21	0.380	ec.p	anx.y
I_Temperment_Corr_S_Temperment	-0.26	0.008	ne.p	anx.y
I_Temperment_Corr_I_Adv	-0.18	0.000	ne.p	anx.y
I_Temperment_Corr_S_Adv	0.07	0.368	ne.p	anx.y
I_Temperment_Corr_I_Factor	-0.34	0.000	ne.p	anx.y
I_Temperment_Corr_S_Factor	-0.03	0.762	ne.p	anx.y
S_Temperment_Corr_I_Adv	-0.08	0.157	ne.p	anx.y
S_Temperment_Corr_S_Adv	-0.18	0.075	ne.p	anx.y
S_Temperment_Corr_I_Factor	-0.08	0.342	ne.p	anx.y
**S_Temperment_Corr_S_Factor**	**0.04**	**0.676**	**ne.p**	**anx.y**
I_Adv_Corr_S_Adv	-0.38	0.014	ne.p	anx.y
I_Adv_Corr_I_Factor	0.45	0.000	ne.p	anx.y
I_Adv_Corr_S_Factor	-0.22	0.019	ne.p	anx.y
S_Adv_Corr_I_Factor	-0.33	0.006	ne.p	anx.y
S_Adv_Corr_S_Factor	0.54	0.000	ne.p	anx.y

I_Factor_Corr_S_Factor	-0.20	0.417	ne.p	anx.y
I_Temperment_Corr_S_Temperment	-0.23	0.013	ec.p	avoid.y
I_Temperment_Corr_I_Adv	-0.30	0.000	ec.p	avoid.y
I_Temperment_Corr_S_Adv	0.19	0.012	ec.p	avoid.y
I_Temperment_Corr_I_Factor	-0.16	0.002	ec.p	avoid.y
I_Temperment_Corr_S_Factor	-0.03	0.616	ec.p	avoid.y
S_Temperment_Corr_I_Adv	0.01	0.826	ec.p	avoid.y
S_Temperment_Corr_S_Adv	-0.30	0.003	ec.p	avoid.y
S_Temperment_Corr_I_Factor	0.09	0.175	ec.p	avoid.y
**S_Temperment_Corr_S_Factor**	**-0.16**	**0.023**	**ec.p**	**avoid.y**
I_Adv_Corr_S_Adv	-0.39	0.013	ec.p	avoid.y
I_Adv_Corr_I_Factor	0.24	0.000	ec.p	avoid.y
I_Adv_Corr_S_Factor	-0.10	0.131	ec.p	avoid.y
S_Adv_Corr_I_Factor	-0.17	0.053	ec.p	avoid.y
S_Adv_Corr_S_Factor	0.46	0.000	ec.p	avoid.y
I_Factor_Corr_S_Factor	-0.27	0.031	ec.p	avoid.y
I_Temperment_Corr_S_Temperment	-0.27	0.004	ne.p	avoid.y
I_Temperment_Corr_I_Adv	-0.18	0.000	ne.p	avoid.y
I_Temperment_Corr_S_Adv	0.07	0.342	ne.p	avoid.y
I_Temperment_Corr_I_Factor	-0.10	0.052	ne.p	avoid.y
I_Temperment_Corr_S_Factor	-0.04	0.493	ne.p	avoid.y
S_Temperment_Corr_I_Adv	-0.07	0.199	ne.p	avoid.y
S_Temperment_Corr_S_Adv	-0.17	0.071	ne.p	avoid.y
S_Temperment_Corr_I_Factor	0.03	0.654	ne.p	avoid.y
**S_Temperment_Corr_S_Factor**	**-0.15**	**0.030**	**ne.p**	**avoid.y**
I_Adv_Corr_S_Adv	-0.40	0.011	ne.p	avoid.y
I_Adv_Corr_I_Factor	0.24	0.000	ne.p	avoid.y
I_Adv_Corr_S_Factor	-0.11	0.112	ne.p	avoid.y
S_Adv_Corr_I_Factor	-0.17	0.055	ne.p	avoid.y
S_Adv_Corr_S_Factor	0.46	0.000	ne.p	avoid.y
I_Factor_Corr_S_Factor	-0.26	0.035	ne.p	avoid.y
I_Temperment_Corr_S_Temperment	-0.24	0.008	ec.p	PSS.y
I_Temperment_Corr_I_Adv	-0.30	0.000	ec.p	PSS.y

I_Temperment_Corr_S_Adv	0.18	0.013	ec.p	PSS.y
I_Temperment_Corr_I_Factor	0.15	0.001	ec.p	PSS.y
I_Temperment_Corr_S_Factor	-0.05	0.274	ec.p	PSS.y
S_Temperment_Corr_I_Adv	0.01	0.837	ec.p	PSS.y
S_Temperment_Corr_S_Adv	-0.30	0.003	ec.p	PSS.y
S_Temperment_Corr_I_Factor	-0.02	0.748	ec.p	PSS.y
**S_Temperment_Corr_S_Factor**	**0.07**	**0.190**	**ec.p**	**PSS.y**
I_Adv_Corr_S_Adv	-0.39	0.013	ec.p	PSS.y
I_Adv_Corr_I_Factor	-0.07	0.171	ec.p	PSS.y
I_Adv_Corr_S_Factor	-0.05	0.318	ec.p	PSS.y
S_Adv_Corr_I_Factor	-0.05	0.447	ec.p	PSS.y
S_Adv_Corr_S_Factor	-0.18	0.043	ec.p	PSS.y
I_Factor_Corr_S_Factor	-0.49	0.000	ec.p	PSS.y
I_Temperment_Corr_S_Temperment	-0.26	0.005	ne.p	PSS.y
I_Temperment_Corr_I_Adv	-0.18	0.000	ne.p	PSS.y
I_Temperment_Corr_S_Adv	0.07	0.317	ne.p	PSS.y
I_Temperment_Corr_I_Factor	0.20	0.000	ne.p	PSS.y
I_Temperment_Corr_S_Factor	-0.04	0.360	ne.p	PSS.y
S_Temperment_Corr_I_Adv	-0.07	0.204	ne.p	PSS.y
S_Temperment_Corr_S_Adv	-0.19	0.048	ne.p	PSS.y
S_Temperment_Corr_I_Factor	-0.13	0.009	ne.p	PSS.y
**S_Temperment_Corr_S_Factor**	**0.12**	**0.017**	**ne.p**	**PSS.y**
I_Adv_Corr_S_Adv	-0.39	0.012	ne.p	PSS.y
I_Adv_Corr_I_Factor	-0.05	0.298	ne.p	PSS.y
I_Adv_Corr_S_Factor	-0.06	0.249	ne.p	PSS.y
S_Adv_Corr_I_Factor	-0.06	0.352	ne.p	PSS.y
S_Adv_Corr_S_Factor	-0.17	0.047	ne.p	PSS.y
I_Factor_Corr_S_Factor	-0.49	0.000	ne.p	PSS.y
I_Temperment_Corr_S_Temperment	-0.24	0.009	ec.p	ProSoc.y
I_Temperment_Corr_I_Adv	-0.30	0.000	ec.p	ProSoc.y
I_Temperment_Corr_S_Adv	0.19	0.012	ec.p	ProSoc.y
I_Temperment_Corr_I_Factor	0.06	0.200	ec.p	ProSoc.y
I_Temperment_Corr_S_Factor	0.05	0.288	ec.p	ProSoc.y

S_Temperment_Corr_I_Adv	0.01	0.837	ec.p	ProSoc.y
S_Temperment_Corr_S_Adv	-0.31	0.003	ec.p	ProSoc.y
S_Temperment_Corr_I_Factor	0.00	0.974	ec.p	ProSoc.y
**S_Temperment_Corr_S_Factor**	**-0.03**	**0.583**	**ec.p**	**ProSoc.y**
I_Adv_Corr_S_Adv	-0.38	0.021	ec.p	ProSoc.y
I_Adv_Corr_I_Factor	-0.08	0.100	ec.p	ProSoc.y
I_Adv_Corr_S_Factor	0.04	0.439	ec.p	ProSoc.y
S_Adv_Corr_I_Factor	0.08	0.289	ec.p	ProSoc.y
S_Adv_Corr_S_Factor	-0.04	0.628	ec.p	ProSoc.y
I_Factor_Corr_S_Factor	-0.63	0.000	ec.p	ProSoc.y
I_Temperment_Corr_S_Temperment	-0.27	0.004	ne.p	ProSoc.y
I_Temperment_Corr_I_Adv	-0.18	0.000	ne.p	ProSoc.y
I_Temperment_Corr_S_Adv	0.07	0.357	ne.p	ProSoc.y
I_Temperment_Corr_I_Factor	0.05	0.260	ne.p	ProSoc.y
I_Temperment_Corr_S_Factor	-0.02	0.630	ne.p	ProSoc.y
S_Temperment_Corr_I_Adv	-0.08	0.188	ne.p	ProSoc.y
S_Temperment_Corr_S_Adv	-0.18	0.067	ne.p	ProSoc.y
S_Temperment_Corr_I_Factor	0.06	0.274	ne.p	ProSoc.y
**S_Temperment_Corr_S_Factor**	**-0.06**	**0.274**	**ne.p**	**ProSoc.y**
I_Adv_Corr_S_Adv	-0.39	0.017	ne.p	ProSoc.y
I_Adv_Corr_I_Factor	-0.08	0.108	ne.p	ProSoc.y
I_Adv_Corr_S_Factor	0.05	0.384	ne.p	ProSoc.y
S_Adv_Corr_I_Factor	0.08	0.279	ne.p	ProSoc.y
S_Adv_Corr_S_Factor	-0.07	0.441	ne.p	ProSoc.y
I_Factor_Corr_S_Factor	-0.63	0.000	ne.p	ProSoc.y
I_Temperment_Corr_S_Temperment	-0.24	0.011	ec.p	SEQ.y
I_Temperment_Corr_I_Adv	-0.30	0.000	ec.p	SEQ.y
I_Temperment_Corr_S_Adv	0.18	0.013	ec.p	SEQ.y
I_Temperment_Corr_I_Factor	-0.15	0.000	ec.p	SEQ.y
I_Temperment_Corr_S_Factor	0.03	0.519	ec.p	SEQ.y
S_Temperment_Corr_I_Adv	0.02	0.783	ec.p	SEQ.y
S_Temperment_Corr_S_Adv	-0.31	0.002	ec.p	SEQ.y
S_Temperment_Corr_I_Factor	-0.05	0.294	ec.p	SEQ.y

S_Temperment_Corr_S_Factor	-0.09	0.108	ec.p	SEQ.y
I_Adv_Corr_S_Adv	-0.39	0.012	ec.p	SEQ.y
I_Adv_Corr_I_Factor	0.29	0.000	ec.p	SEQ.y
I_Adv_Corr_S_Factor	-0.09	0.073	ec.p	SEQ.y
S_Adv_Corr_I_Factor	-0.17	0.012	ec.p	SEQ.y
S_Adv_Corr_S_Factor	0.37	0.000	ec.p	SEQ.y
I_Factor_Corr_S_Factor	-0.51	0.000	ec.p	SEQ.y
I_Temperment_Corr_S_Temperment	-0.27	0.005	ne.p	SEQ.y
I_Temperment_Corr_I_Adv	-0.18	0.000	ne.p	SEQ.y
I_Temperment_Corr_S_Adv	0.07	0.347	ne.p	SEQ.y
I_Temperment_Corr_I_Factor	-0.13	0.002	ne.p	SEQ.y
I_Temperment_Corr_S_Factor	0.01	0.904	ne.p	SEQ.y
S_Temperment_Corr_I_Adv	-0.07	0.203	ne.p	SEQ.y
S_Temperment_Corr_S_Adv	-0.18	0.054	ne.p	SEQ.y
S_Temperment_Corr_I_Factor	-0.05	0.299	ne.p	SEQ.y
S_Temperment_Corr_S_Factor	-0.08	0.135	ne.p	SEQ.y
I_Adv_Corr_S_Adv	-0.39	0.011	ne.p	SEQ.y
I_Adv_Corr_I_Factor	0.29	0.000	ne.p	SEQ.y
I_Adv_Corr_S_Factor	-0.09	0.071	ne.p	SEQ.y
S_Adv_Corr_I_Factor	-0.18	0.009	ne.p	SEQ.y
S_Adv_Corr_S_Factor	0.36	0.000	ne.p	SEQ.y
I_Factor_Corr_S_Factor	-0.51	0.000	ne.p	SEQ.y

Note: “Factor” refers to the exploratory third variable.

Table S14. Regression of Personality Intercept and Slope on Adversity and Third Variable

Parameter	Estimate	SE	PValue	StdAll	Personality Construct	Factor	ModelName
S_Temperment_ON_I_Adv	-0.04	0.09	0.673	-0.04	ec.p	anx.y	tri_adv_ec.p_anx.y
S_Temperment_ON_S_Adv	-0.77	0.49	0.113	-0.44	ec.p	anx.y	tri_adv_ec.p_anx.y
S_Temperment_ON_I_Factor	0.14	0.07	0.037	0.27	ec.p	anx.y	tri_adv_ec.p_anx.y
S_Temperment_ON_S_Factor	-0.16	0.07	0.024	-0.36	ec.p	anx.y	tri_adv_ec.p_anx.y
S_Temperment_ON_Cohort	-0.05	0.05	0.369	-0.13	ec.p	anx.y	tri_adv_ec.p_anx.y
I_Temperment_ON_I_Adv	-0.47	0.09	0.000	-0.28	ec.p	anx.y	tri_adv_ec.p_anx.y
I_Temperment_ON_I_Factor	-0.30	0.07	0.000	-0.32	ec.p	anx.y	tri_adv_ec.p_anx.y
I_Temperment_ON_Cohort	0.13	0.03	0.000	0.20	ec.p	anx.y	tri_adv_ec.p_anx.y
S_Temperment_ON_I_Adv	-0.10	0.09	0.226	-0.12	ne.p	anx.y	tri_adv_ne.p_anx.y
S_Temperment_ON_S_Adv	-0.72	0.48	0.138	-0.44	ne.p	anx.y	tri_adv_ne.p_anx.y
S_Temperment_ON_I_Factor	0.01	0.06	0.866	0.02	ne.p	anx.y	tri_adv_ne.p_anx.y
S_Temperment_ON_S_Factor	-0.01	0.06	0.920	-0.01	ne.p	anx.y	tri_adv_ne.p_anx.y
S_Temperment_ON_Cohort	-0.02	0.05	0.733	-0.05	ne.p	anx.y	tri_adv_ne.p_anx.y
I_Temperment_ON_I_Adv	-0.23	0.08	0.004	-0.16	ne.p	anx.y	tri_adv_ne.p_anx.y
I_Temperment_ON_I_Factor	-0.31	0.06	0.000	-0.40	ne.p	anx.y	tri_adv_ne.p_anx.y
I_Temperment_ON_Cohort	0.11	0.03	0.000	0.20	ne.p	anx.y	tri_adv_ne.p_anx.y
S_Temperment_ON_I_Adv	-0.02	0.09	0.847	-0.02	ec.p	avoid.y	tri_adv_ec.p_avoid.y
S_Temperment_ON_S_Adv	-0.77	0.48	0.108	-0.46	ec.p	avoid.y	tri_adv_ec.p_avoid.y
S_Temperment_ON_I_Factor	0.04	0.03	0.147	0.14	ec.p	avoid.y	tri_adv_ec.p_avoid.y
S_Temperment_ON_S_Factor	-0.07	0.03	0.043	-0.23	ec.p	avoid.y	tri_adv_ec.p_avoid.y
S_Temperment_ON_Cohort	-0.09	0.05	0.051	-0.27	ec.p	avoid.y	tri_adv_ec.p_avoid.y
I_Temperment_ON_I_Adv	-0.53	0.09	0.000	-0.32	ec.p	avoid.y	tri_adv_ec.p_avoid.y
I_Temperment_ON_I_Factor	-0.08	0.03	0.005	-0.16	ec.p	avoid.y	tri_adv_ec.p_avoid.y
I_Temperment_ON_Cohort	0.18	0.03	0.000	0.28	ec.p	avoid.y	tri_adv_ec.p_avoid.y
S_Temperment_ON_I_Adv	-0.10	0.08	0.221	-0.11	ne.p	avoid.y	tri_adv_ne.p_avoid.y
S_Temperment_ON_S_Adv	-0.56	0.44	0.205	-0.33	ne.p	avoid.y	tri_adv_ne.p_avoid.y
S_Temperment_ON_I_Factor	0.03	0.03	0.359	0.09	ne.p	avoid.y	tri_adv_ne.p_avoid.y
S_Temperment_ON_S_Factor	-0.07	0.03	0.025	-0.25	ne.p	avoid.y	tri_adv_ne.p_avoid.y
S_Temperment_ON_Cohort	-0.01	0.04	0.769	-0.04	ne.p	avoid.y	tri_adv_ne.p_avoid.y
I_Temperment_ON_I_Adv	-0.30	0.08	0.000	-0.21	ne.p	avoid.y	tri_adv_ne.p_avoid.y
I_Temperment_ON_I_Factor	-0.06	0.03	0.027	-0.13	ne.p	avoid.y	tri_adv_ne.p_avoid.y
I_Temperment_ON_Cohort	0.15	0.03	0.000	0.29	ne.p	avoid.y	tri_adv_ne.p_avoid.y

S_Temperment_ON_I_Adv	0.00	0.09	0.971	0.00	ec.p	PSS.y	tri_adv_ec.p_PSS.y
S_Temperment_ON_S_Adv	-0.88	0.50	0.078	-0.53	ec.p	PSS.y	tri_adv_ec.p_PSS.y
S_Temperment_ON_I_Factor	-0.02	0.14	0.884	-0.01	ec.p	PSS.y	tri_adv_ec.p_PSS.y
S_Temperment_ON_S_Factor	0.09	0.13	0.503	0.05	ec.p	PSS.y	tri_adv_ec.p_PSS.y
S_Temperment_ON_Cohort	-0.09	0.05	0.057	-0.26	ec.p	PSS.y	tri_adv_ec.p_PSS.y
I_Temperment_ON_I_Adv	-0.56	0.09	0.000	-0.33	ec.p	PSS.y	tri_adv_ec.p_PSS.y
I_Temperment_ON_I_Factor	0.41	0.15	0.005	0.13	ec.p	PSS.y	tri_adv_ec.p_PSS.y
I_Temperment_ON_Cohort	0.16	0.03	0.000	0.26	ec.p	PSS.y	tri_adv_ec.p_PSS.y
S_Temperment_ON_I_Adv	-0.10	0.08	0.216	-0.11	ne.p	PSS.y	tri_adv_ne.p_PSS.y
S_Temperment_ON_S_Adv	-0.70	0.46	0.134	-0.40	ne.p	PSS.y	tri_adv_ne.p_PSS.y
S_Temperment_ON_I_Factor	-0.29	0.13	0.028	-0.16	ne.p	PSS.y	tri_adv_ne.p_PSS.y
S_Temperment_ON_S_Factor	0.13	0.12	0.271	0.08	ne.p	PSS.y	tri_adv_ne.p_PSS.y
S_Temperment_ON_Cohort	-0.02	0.04	0.662	-0.06	ne.p	PSS.y	tri_adv_ne.p_PSS.y
I_Temperment_ON_I_Adv	-0.31	0.08	0.000	-0.21	ne.p	PSS.y	tri_adv_ne.p_PSS.y
I_Temperment_ON_I_Factor	0.56	0.12	0.000	0.21	ne.p	PSS.y	tri_adv_ne.p_PSS.y
I_Temperment_ON_Cohort	0.14	0.02	0.000	0.27	ne.p	PSS.y	tri_adv_ne.p_PSS.y
S_Temperment_ON_I_Adv	0.00	0.09	0.978	0.00	ec.p	ProSoc.y	tri_adv_ec.p_ProSoc.y
S_Temperment_ON_S_Adv	-0.93	0.52	0.074	-0.56	ec.p	ProSoc.y	tri_adv_ec.p_ProSoc.y
S_Temperment_ON_I_Factor	0.00	0.02	0.916	0.01	ec.p	ProSoc.y	tri_adv_ec.p_ProSoc.y
S_Temperment_ON_S_Factor	0.00	0.02	0.934	0.01	ec.p	ProSoc.y	tri_adv_ec.p_ProSoc.y
S_Temperment_ON_Cohort	-0.09	0.05	0.056	-0.28	ec.p	ProSoc.y	tri_adv_ec.p_ProSoc.y
I_Temperment_ON_I_Adv	-0.57	0.09	0.000	-0.34	ec.p	ProSoc.y	tri_adv_ec.p_ProSoc.y
I_Temperment_ON_I_Factor	0.02	0.02	0.300	0.05	ec.p	ProSoc.y	tri_adv_ec.p_ProSoc.y
I_Temperment_ON_Cohort	0.16	0.03	0.000	0.25	ec.p	ProSoc.y	tri_adv_ec.p_ProSoc.y
S_Temperment_ON_I_Adv	-0.09	0.08	0.299	-0.10	ne.p	ProSoc.y	tri_adv_ne.p_ProSoc.y
S_Temperment_ON_S_Adv	-0.76	0.49	0.119	-0.46	ne.p	ProSoc.y	tri_adv_ne.p_ProSoc.y
S_Temperment_ON_I_Factor	0.01	0.02	0.519	0.06	ne.p	ProSoc.y	tri_adv_ne.p_ProSoc.y
S_Temperment_ON_S_Factor	0.00	0.02	0.820	-0.02	ne.p	ProSoc.y	tri_adv_ne.p_ProSoc.y
S_Temperment_ON_Cohort	-0.02	0.05	0.612	-0.07	ne.p	ProSoc.y	tri_adv_ne.p_ProSoc.y
I_Temperment_ON_I_Adv	-0.33	0.08	0.000	-0.23	ne.p	ProSoc.y	tri_adv_ne.p_ProSoc.y
I_Temperment_ON_I_Factor	0.02	0.02	0.247	0.05	ne.p	ProSoc.y	tri_adv_ne.p_ProSoc.y
I_Temperment_ON_Cohort	0.14	0.03	0.000	0.26	ne.p	ProSoc.y	tri_adv_ne.p_ProSoc.y
S_Temperment_ON_I_Adv	0.05	0.09	0.596	0.05	ec.p	SEQ.y	tri_adv_ec.p_SEQ.y
S_Temperment_ON_S_Adv	-0.89	0.53	0.094	-0.53	ec.p	SEQ.y	tri_adv_ec.p_SEQ.y
S_Temperment_ON_I_Factor	-0.10	0.04	0.029	-0.17	ec.p	SEQ.y	tri_adv_ec.p_SEQ.y
S_Temperment_ON_S_Factor	-0.10	0.04	0.017	-0.19	ec.p	SEQ.y	tri_adv_ec.p_SEQ.y

S_Temperment_ON_Cohort	-0.09	0.05	0.085	-0.25	ec.p	SEQ.y	tri_adv_ec.p_SEQ.y
I_Temperment_ON_I_Adv	-0.53	0.09	0.000	-0.32	ec.p	SEQ.y	tri_adv_ec.p_SEQ.y
I_Temperment_ON_I_Factor	-0.10	0.04	0.021	-0.10	ec.p	SEQ.y	tri_adv_ec.p_SEQ.y
I_Temperment_ON_Cohort	0.16	0.03	0.000	0.25	ec.p	SEQ.y	tri_adv_ec.p_SEQ.y
S_Temperment_ON_I_Adv	-0.06	0.09	0.516	-0.06	ne.p	SEQ.y	tri_adv_ne.p_SEQ.y
S_Temperment_ON_S_Adv	-0.70	0.49	0.153	-0.42	ne.p	SEQ.y	tri_adv_ne.p_SEQ.y
S_Temperment_ON_I_Factor	-0.08	0.04	0.050	-0.16	ne.p	SEQ.y	tri_adv_ne.p_SEQ.y
S_Temperment_ON_S_Factor	-0.10	0.04	0.012	-0.20	ne.p	SEQ.y	tri_adv_ne.p_SEQ.y
S_Temperment_ON_Cohort	-0.01	0.05	0.799	-0.04	ne.p	SEQ.y	tri_adv_ne.p_SEQ.y
I_Temperment_ON_I_Adv	-0.29	0.08	0.000	-0.21	ne.p	SEQ.y	tri_adv_ne.p_SEQ.y
I_Temperment_ON_I_Factor	-0.09	0.04	0.017	-0.11	ne.p	SEQ.y	tri_adv_ne.p_SEQ.y
I_Temperment_ON_Cohort	0.13	0.02	0.000	0.26	ne.p	SEQ.y	tri_adv_ne.p_SEQ.y

Note: “Factor” refers to the exploratory third variable.

Domain Level Adversity Robustness Analyses

As a robustness check, we examined how our main results differed when we examined each of the individual 16 adversity domains in isolation. First, we ran univariate unconditional latent growth curve models for each of the adversity domains. Model fit indices are reported in Table S15 and model parameters of interest are reported in Table S16. The variance of the slope parameter was only significant for the domains of parent-child relationship stress ("parentchild.relationship.adv"), stress related to the relationship between the youth's parents ("parentparent.relationship.adv") and discrimination/acculturation stress ("discrimination.adv"), which should be kept in mind when interpreting additional output.

Though the slope variance was mostly non-significant, indicating that reliable individual differences in change were not present at the level of individual adversity domains, we still computed bivariate latent growth curve models for all adversity domains and parent and youth-reported effortful control and emotional stability to get a comprehensive picture of how the size of the slope-slope correlations compared to what we found in the main analyses. Model fit indices are reported in Table S17 and model parameters of interest are reported in Table S18. All of the bivariate models that used body image (referred to as "body" in the output) have negative variances for the body image slope and should be dis-regarded.

There was negative correlated change between parent-child relationship stress and youth-reported effortful control (r = -0.39, p = .003) and emotional stability (r = -0.39, p = .002). The variance of the parent-child relationship stress slopes was consistently significant in all models. There was no correlated change for the other two domains where the slope variance was initially significant. There were also consistent patterns of correlated change for academic stress, peer stress, and violence. Though these estimates should be interpreted cautiously, we highlight them here as potentially interesting direction for future research. Change in academic stress was negatively associated with growth in youth and parent reported effortful control (r = **-0.79**, p < **.000**; r = **-0.50**, p < **.000**) and emotional stability (r = **-0.57**, p < **.000**; r = **-0.29**, p = **.008**). Change in peer stress was negatively associated with growth in youth and parent-reported effortful control (r = **-0.29**, p = **.022**; r = **-0.43**, p < **.000**) and emotional stability (r = **-0.51**, p = **.001**; r = **-0.25**, p = **.026**). Change in stress from exposure to violence was negatively associated with growth in youth and parent reported effortful control (r = **-0.37**, p = **.025**; r = **-0.31**, p = **.026**) and parent-reported emotional stability (r = **-0.31**, p = **.029**).

Because of the significant slope variance was limited to just three domains, we decided to only run further analyses for RQs 3 and 4 with those domains. Fit indices for the supplemental RQ 3 models are reported in Tables S19a-c.The proportions on participants who grew in low-adversity, initial adversity, and increasing adversity conditions are reported for parent-child relationship stress (Table S20a), parent-parent relationship stress (Table S20b), and discrimination/acculturation stress (Table S20c). We also re-ran the trivariate regression models for each of these three adversity domains. The fit indices for the models are reported in Table S21, and the model parameters are reported in Table S22.

Table S15. Adversity Domain Univariate Latent Growth Curve Model Fit

chisq	df	rmsea	tli	cfi	ModID
7.838	1	0.102	0.904	0.968	academic.adv
7.764	1	0.101	0.836	0.945	behavioral.adv
0.815	1	0	1.002	1	peer.adv
2.284	1	0.044	0.986	0.995	parentchild.relationship.adv
0.718	1	0	1.004	1	parentparent.relationship.adv
0.53	1	0	1.008	1	household.relationship.adv
1.061	1	0.01	0.999	1	body.adv
2.54	1	0.048	0.98	0.993	romantic.adv
1.711	1	0.033	0.986	0.995	neighborhood.adv
4.779	1	0.075	0.907	0.969	health.self.adv
0.022	1	0	1.031	1	health.other.adv
9.591	1	0.114	0.889	0.963	finance.adv
12.068	1	0.129	0.494	0.831	legal.adv
4.81	1	0.076	0.879	0.96	violence.adv
0.377	1	0	1.013	1	discrimination.adv
0.132	1	0	1.056	1	activities.adv

Table S16. Adversity Domain Univariate Latent Growth Curve Model Parameters

Parameter	Estimate	SE	PValue	StdAll	Model
AdvInterceptOfIntercept	1.79	0.03	0.000	3.46	academic.adv
AdvVarianceOfIntercept	0.27	0.05	0.000	1.00	academic.adv
AdvInterceptOfSlope	0.05	0.04	0.147	0.14	academic.adv
AdvVarianceOfSlope	0.14	0.10	0.160	1.00	academic.adv
AdvInterceptOfIntercept	1.42	0.03	0.000	3.19	behavioral.adv
AdvVarianceOfIntercept	0.20	0.04	0.000	1.00	behavioral.adv
AdvInterceptOfSlope	-0.25	0.03	0.000	-1.21	behavioral.adv
AdvVarianceOfSlope	0.04	0.07	0.561	1.00	behavioral.adv
AdvInterceptOfIntercept	1.90	0.03	0.000	3.54	peer.adv
AdvVarianceOfIntercept	0.29	0.05	0.000	1.00	peer.adv
AdvInterceptOfSlope	-0.22	0.04	0.000	-0.57	peer.adv
AdvVarianceOfSlope	0.14	0.09	0.097	1.00	peer.adv
AdvInterceptOfIntercept	1.95	0.03	0.000	4.08	parentchild.relationship.adv
AdvVarianceOfIntercept	0.23	0.04	0.000	1.00	parentchild.relationship.adv
AdvInterceptOfSlope	-0.05	0.04	0.159	-0.11	parentchild.relationship.adv
**AdvVarianceOfSlope**	**0.21**	**0.09**	**0.025**	**1.00**	**parentchild.relationship.adv**
AdvInterceptOfIntercept	1.84	0.03	0.000	3.05	parentparent.relationship.adv
AdvVarianceOfIntercept	0.36	0.06	0.000	1.00	parentparent.relationship.adv
AdvInterceptOfSlope	-0.11	0.04	0.009	-0.16	parentparent.relationship.adv
**AdvVarianceOfSlope**	**0.43**	**0.11**	**0.000**	**1.00**	**parentparent.relationship.adv**
AdvInterceptOfIntercept	2.06	0.03	0.000	4.43	household.relationship.adv
AdvVarianceOfIntercept	0.22	0.04	0.000	1.00	household.relationship.adv
AdvInterceptOfSlope	-0.14	0.04	0.000	-0.46	household.relationship.adv
AdvVarianceOfSlope	0.09	0.09	0.327	1.00	household.relationship.adv
AdvInterceptOfIntercept	1.59	0.03	0.000	3.66	body.adv
AdvVarianceOfIntercept	0.19	0.04	0.000	1.00	body.adv
AdvInterceptOfSlope	0.00	0.03	0.998	NA	body.adv
AdvVarianceOfSlope	-0.02	0.09	0.784	NA	body.adv
AdvInterceptOfIntercept	1.38	0.02	0.000	3.40	romantic.adv
AdvVarianceOfIntercept	0.16	0.03	0.000	1.00	romantic.adv

AdvInterceptOfSlope	0.03	0.03	0.292	0.12	romantic.adv
AdvVarianceOfSlope	0.06	0.06	0.324	1.00	romantic.adv
AdvInterceptOfIntercept	1.52	0.03	0.000	3.50	neighborhood.adv
AdvVarianceOfIntercept	0.19	0.04	0.000	1.00	neighborhood.adv
AdvInterceptOfSlope	0.02	0.03	0.551	0.07	neighborhood.adv
AdvVarianceOfSlope	0.08	0.08	0.307	1.00	neighborhood.adv
AdvInterceptOfIntercept	1.49	0.03	0.000	3.81	health.self.adv
AdvVarianceOfIntercept	0.15	0.04	0.000	1.00	health.self.adv
AdvInterceptOfSlope	-0.10	0.03	0.006	-0.63	health.self.adv
AdvVarianceOfSlope	0.02	0.08	0.776	1.00	health.self.adv
AdvInterceptOfIntercept	2.08	0.04	0.000	3.41	health.other.adv
AdvVarianceOfIntercept	0.37	0.09	0.000	1.00	health.other.adv
AdvInterceptOfSlope	-0.26	0.05	0.000	-0.55	health.other.adv
AdvVarianceOfSlope	0.23	0.18	0.205	1.00	health.other.adv
AdvInterceptOfIntercept	1.53	0.03	0.000	3.66	finance.adv
AdvVarianceOfIntercept	0.17	0.04	0.000	1.00	finance.adv
AdvInterceptOfSlope	-0.07	0.04	0.076	-0.16	finance.adv
AdvVarianceOfSlope	0.18	0.10	0.060	1.00	finance.adv
AdvInterceptOfIntercept	1.54	0.03	0.000	4.04	legal.adv
AdvVarianceOfIntercept	0.14	0.06	0.012	1.00	legal.adv
AdvInterceptOfSlope	-0.19	0.04	0.000	-0.64	legal.adv
AdvVarianceOfSlope	0.09	0.12	0.482	1.00	legal.adv
AdvInterceptOfIntercept	1.43	0.03	0.000	3.72	violence.adv
AdvVarianceOfIntercept	0.15	0.04	0.000	1.00	violence.adv
AdvInterceptOfSlope	-0.23	0.03	0.000	-0.79	violence.adv
AdvVarianceOfSlope	0.08	0.08	0.313	1.00	violence.adv
AdvInterceptOfIntercept	1.22	0.02	0.000	5.05	discrimination.adv
AdvVarianceOfIntercept	0.06	0.02	0.002	1.00	discrimination.adv
AdvInterceptOfSlope	-0.01	0.03	0.833	-0.01	discrimination.adv
**AdvVarianceOfSlope**	**0.14**	**0.04**	**0.001**	**1.00**	**discrimination.adv**
AdvInterceptOfIntercept	1.35	0.02	0.000	5.91	activities.adv
AdvVarianceOfIntercept	0.05	0.02	0.010	1.00	activities.adv
AdvInterceptOfSlope	-0.10	0.02	0.000	-1.45	activities.adv

AdvVarianceOfSlope	0.00	0.04	0.909	1.00	activities.adv

Table S17. Adversity Domain Bivariate Latent Growth Curve Model Fit

chisq	df	rmsea	tli	cfi	ModID
39.678	7	0.083	0.927	0.966	academic.adv_ec.y
41.195	7	0.085	0.898	0.952	behavioral.adv_ec.y
34.55	7	0.076	0.928	0.966	peer.adv_ec.y
50.21	7	0.095	0.901	0.954	parentchild.relationship.adv_ec.y
33.672	7	0.075	0.93	0.967	parentparent.relationship.adv_ec.y
40.118	7	0.083	0.908	0.957	household.relationship.adv_ec.y
27.244	7	0.065	0.949	0.976	body.adv_ec.y
44.915	7	0.089	0.903	0.955	romantic.adv_ec.y
30.124	7	0.07	0.932	0.968	neighborhood.adv_ec.y
31.709	7	0.072	0.922	0.963	health.self.adv_ec.y
29.581	7	0.069	0.926	0.965	health.other.adv_ec.y
40.516	7	0.084	0.913	0.959	finance.adv_ec.y
36.487	7	0.079	0.9	0.953	legal.adv_ec.y
39.232	7	0.082	0.896	0.952	violence.adv_ec.y
32.419	7	0.073	0.924	0.964	discrimination.adv_ec.y
25.663	7	0.063	0.935	0.97	activities.adv_ec.y
42.416	7	0.086	0.948	0.976	academic.adv_ec.p
40.8	7	0.084	0.943	0.973	behavioral.adv_ec.p
30.474	7	0.07	0.963	0.983	peer.adv_ec.p
29.86	7	0.069	0.965	0.984	parentchild.relationship.adv_ec.p
33.207	7	0.074	0.958	0.98	parentparent.relationship.adv_ec.p
25.034	7	0.062	0.97	0.986	household.relationship.adv_ec.p
30.887	7	0.071	0.962	0.982	body.adv_ec.p
29.322	7	0.068	0.964	0.983	romantic.adv_ec.p
30.17	7	0.07	0.96	0.981	neighborhood.adv_ec.p
31.089	7	0.071	0.957	0.98	health.self.adv_ec.p
25.353	7	0.062	0.967	0.984	health.other.adv_ec.p
38.655	7	0.082	0.949	0.976	finance.adv_ec.p
38.576	7	0.081	0.941	0.973	legal.adv_ec.p
30.452	7	0.07	0.958	0.98	violence.adv_ec.p

25.362	7	0.062	0.968	0.985	discrimination.adv_ec.p
29.701	7	0.069	0.957	0.98	activities.adv_ec.p
84.274	7	0.127	0.709	0.864	academic.adv_ne.y
80.949	7	0.124	0.647	0.835	behavioral.adv_ne.y
80.03	7	0.124	0.747	0.882	peer.adv_ne.y
85.877	7	0.129	0.744	0.881	parentchild.relationship.adv_ne.y
74.539	7	0.119	0.742	0.879	parentparent.relationship.adv_ne.y
80.512	7	0.124	0.704	0.862	household.relationship.adv_ne.y
79.196	7	0.123	0.756	0.886	body.adv_ne.y
86.497	7	0.129	0.701	0.86	romantic.adv_ne.y
87.191	7	0.13	0.654	0.838	neighborhood.adv_ne.y
82.068	7	0.125	0.638	0.831	health.self.adv_ne.y
73.485	7	0.118	0.656	0.839	health.other.adv_ne.y
84.818	7	0.128	0.709	0.864	finance.adv_ne.y
85.272	7	0.128	0.58	0.804	legal.adv_ne.y
86.084	7	0.129	0.61	0.818	violence.adv_ne.y
74.453	7	0.119	0.696	0.858	discrimination.adv_ne.y
73.298	7	0.118	0.637	0.83	activities.adv_ne.y
58.544	7	0.104	0.904	0.955	academic.adv_ne.p
68.35	7	0.114	0.876	0.942	behavioral.adv_ne.p
45.931	7	0.09	0.928	0.966	peer.adv_ne.p
50.508	7	0.096	0.923	0.964	parentchild.relationship.adv_ne.p
44.06	7	0.088	0.93	0.967	parentparent.relationship.adv_ne.p
48.272	7	0.093	0.921	0.963	household.relationship.adv_ne.p
45.715	7	0.09	0.928	0.966	body.adv_ne.p
47.446	7	0.092	0.923	0.964	romantic.adv_ne.p
42.33	7	0.086	0.928	0.966	neighborhood.adv_ne.p
55.755	7	0.101	0.898	0.953	health.self.adv_ne.p
49.012	7	0.094	0.91	0.958	health.other.adv_ne.p
60.347	7	0.106	0.9	0.953	finance.adv_ne.p
57.84	7	0.103	0.887	0.947	legal.adv_ne.p
50.17	7	0.095	0.908	0.957	violence.adv_ne.p
41.192	7	0.085	0.93	0.967	discrimination.adv_ne.p

51.942	7	0.097	0.899	0.953	activities.adv_ne.p

Table S18. Adversity Domain Bivariate Latent Growth Curve Model Parameters

Parameter	Estimate	SE	PValue	StdAll	Construct	Adv
ConstructInterceptOfIntercept	3.44	0.02	0.000	7.59	ec.y	academic.adv
ConstructVarianceOfIntercept	0.21	0.02	0.000	1.00	ec.y	academic.adv
ConstructInterceptOfSlope	0.09	0.02	0.000	0.23	ec.y	academic.adv
ConstructVarianceOfSlope	0.15	0.04	0.000	1.00	ec.y	academic.adv
AdvInterceptOfIntercept	1.79	0.03	0.000	3.52	ec.y	academic.adv
AdvVarianceOfIntercept	0.26	0.05	0.000	1.00	ec.y	academic.adv
AdvInterceptOfSlope	0.05	0.04	0.158	0.14	ec.y	academic.adv
AdvVarianceOfSlope	0.13	0.09	0.161	1.00	ec.y	academic.adv
Cnsrct.Intercept_Corr_Adv_Intercept	-0.15	0.02	0.000	-0.65	ec.y	academic.adv
Cnsrct.Intercept_Corr_Adv_Slope	0.03	0.02	0.082	0.21	ec.y	academic.adv
**Cnsrct.Slope_Corr_Adv_Intercept**	**0.05**	**0.02**	**0.003**	**0.26**	**ec.y**	**academic.adv**
**Cnsrct.Slope_Corr_Adv_Slope**	**-0.11**	**0.02**	**0.000**	**-0.79**	**ec.y**	**academic.adv**
ConstructInterceptOfIntercept	3.44	0.02	0.000	7.53	ec.y	behavioral.adv
ConstructVarianceOfIntercept	0.21	0.02	0.000	1.00	ec.y	behavioral.adv
ConstructInterceptOfSlope	0.09	0.02	0.000	0.23	ec.y	behavioral.adv
ConstructVarianceOfSlope	0.16	0.04	0.000	1.00	ec.y	behavioral.adv
AdvInterceptOfIntercept	1.42	0.03	0.000	3.14	ec.y	behavioral.adv
AdvVarianceOfIntercept	0.20	0.04	0.000	1.00	ec.y	behavioral.adv
AdvInterceptOfSlope	-0.25	0.03	0.000	-1.03	ec.y	behavioral.adv
AdvVarianceOfSlope	0.06	0.07	0.409	1.00	ec.y	behavioral.adv
Cnsrct.Intercept_Corr_Adv_Intercept	-0.08	0.01	0.000	-0.36	ec.y	behavioral.adv
Cnsrct.Intercept_Corr_Adv_Slope	0.04	0.02	0.013	0.39	ec.y	behavioral.adv
Cnsrct.Slope_Corr_Adv_Intercept	0.02	0.02	0.246	0.10	ec.y	behavioral.adv
Cnsrct.Slope_Corr_Adv_Slope	-0.03	0.02	0.170	-0.27	ec.y	behavioral.adv
ConstructInterceptOfIntercept	3.44	0.02	0.000	7.38	ec.y	peer.adv
ConstructVarianceOfIntercept	0.22	0.02	0.000	1.00	ec.y	peer.adv
ConstructInterceptOfSlope	0.09	0.02	0.000	0.21	ec.y	peer.adv
ConstructVarianceOfSlope	0.18	0.04	0.000	1.00	ec.y	peer.adv
AdvInterceptOfIntercept	1.90	0.03	0.000	3.52	ec.y	peer.adv
AdvVarianceOfIntercept	0.29	0.05	0.000	1.00	ec.y	peer.adv

AdvInterceptOfSlope	-0.22	0.04	0.000	-0.56	ec.y	peer.adv
AdvVarianceOfSlope	0.15	0.09	0.078	1.00	ec.y	peer.adv
Cnsrct.Intercept_Corr_Adv_Intercept	-0.08	0.02	0.000	-0.32	ec.y	peer.adv
Cnsrct.Intercept_Corr_Adv_Slope	0.05	0.02	0.009	0.28	ec.y	peer.adv
Cnsrct.Slope_Corr_Adv_Intercept	0.02	0.02	0.207	0.10	ec.y	peer.adv
**Cnsrct.Slope_Corr_Adv_Slope**	**-0.05**	**0.02**	**0.022**	**-0.29**	**ec.y**	**peer.adv**
ConstructInterceptOfIntercept	3.44	0.02	0.000	7.43	ec.y	parentchild.relationship.adv
ConstructVarianceOfIntercept	0.21	0.02	0.000	1.00	ec.y	parentchild.relationship.adv
ConstructInterceptOfSlope	0.09	0.02	0.000	0.22	ec.y	parentchild.relationship.adv
ConstructVarianceOfSlope	0.17	0.04	0.000	1.00	ec.y	parentchild.relationship.adv
AdvInterceptOfIntercept	1.95	0.03	0.000	4.24	ec.y	parentchild.relationship.adv
AdvVarianceOfIntercept	0.21	0.04	0.000	1.00	ec.y	parentchild.relationship.adv
AdvInterceptOfSlope	-0.05	0.04	0.152	-0.13	ec.y	parentchild.relationship.adv
AdvVarianceOfSlope	0.16	0.09	0.073	1.00	ec.y	parentchild.relationship.adv
Cnsrct.Intercept_Corr_Adv_Intercept	-0.09	0.02	0.000	-0.41	ec.y	parentchild.relationship.adv
Cnsrct.Intercept_Corr_Adv_Slope	0.02	0.02	0.240	0.13	ec.y	parentchild.relationship.adv
Cnsrct.Slope_Corr_Adv_Intercept	-0.01	0.02	0.448	-0.07	ec.y	parentchild.relationship.adv
**Cnsrct.Slope_Corr_Adv_Slope**	**-0.06**	**0.02**	**0.003**	**-0.39**	**ec.y**	**parentchild.relationship.adv**
ConstructInterceptOfIntercept	3.44	0.02	0.000	7.47	ec.y	parentparent.relationship.adv
ConstructVarianceOfIntercept	0.21	0.02	0.000	1.00	ec.y	parentparent.relationship.adv
ConstructInterceptOfSlope	0.09	0.02	0.000	0.22	ec.y	parentparent.relationship.adv
ConstructVarianceOfSlope	0.17	0.04	0.000	1.00	ec.y	parentparent.relationship.adv
AdvInterceptOfIntercept	1.85	0.03	0.000	3.04	ec.y	parentparent.relationship.adv
AdvVarianceOfIntercept	0.37	0.06	0.000	1.00	ec.y	parentparent.relationship.adv
AdvInterceptOfSlope	-0.11	0.04	0.009	-0.16	ec.y	parentparent.relationship.adv
**AdvVarianceOfSlope**	**0.44**	**0.11**	**0.000**	**1.00**	**ec.y**	**parentparent.relationship.adv**
Cnsrct.Intercept_Corr_Adv_Intercept	-0.07	0.02	0.000	-0.25	ec.y	parentparent.relationship.adv
Cnsrct.Intercept_Corr_Adv_Slope	0.02	0.02	0.478	0.05	ec.y	parentparent.relationship.adv
Cnsrct.Slope_Corr_Adv_Intercept	0.00	0.02	0.868	-0.01	ec.y	parentparent.relationship.adv
Cnsrct.Slope_Corr_Adv_Slope	-0.03	0.02	0.237	-0.11	ec.y	parentparent.relationship.adv
ConstructInterceptOfIntercept	3.44	0.02	0.000	7.45	ec.y	household.relationship.adv
ConstructVarianceOfIntercept	0.21	0.02	0.000	1.00	ec.y	household.relationship.adv
ConstructInterceptOfSlope	0.09	0.02	0.000	0.22	ec.y	household.relationship.adv

ConstructVarianceOfSlope	0.17	0.04	0.000	1.00	ec.y	household.relationship.adv
AdvInterceptOfIntercept	2.06	0.03	0.000	4.51	ec.y	household.relationship.adv
AdvVarianceOfIntercept	0.21	0.04	0.000	1.00	ec.y	household.relationship.adv
AdvInterceptOfSlope	-0.14	0.04	0.000	-0.51	ec.y	household.relationship.adv
AdvVarianceOfSlope	0.07	0.09	0.419	1.00	ec.y	household.relationship.adv
Cnsrct.Intercept_Corr_Adv_Intercept	-0.07	0.02	0.000	-0.32	ec.y	household.relationship.adv
Cnsrct.Intercept_Corr_Adv_Slope	0.01	0.02	0.544	0.10	ec.y	household.relationship.adv
Cnsrct.Slope_Corr_Adv_Intercept	0.01	0.02	0.575	0.05	ec.y	household.relationship.adv
Cnsrct.Slope_Corr_Adv_Slope	-0.03	0.02	0.152	-0.27	ec.y	household.relationship.adv
ConstructInterceptOfIntercept	3.44	0.02	0.000	7.44	ec.y	body.adv
ConstructVarianceOfIntercept	0.21	0.02	0.000	1.00	ec.y	body.adv
ConstructInterceptOfSlope	0.09	0.02	0.000	0.22	ec.y	body.adv
ConstructVarianceOfSlope	0.17	0.04	0.000	1.00	ec.y	body.adv
AdvInterceptOfIntercept	1.60	0.03	0.000	3.67	ec.y	body.adv
AdvVarianceOfIntercept	0.19	0.04	0.000	1.00	ec.y	body.adv
AdvInterceptOfSlope	0.00	0.03	0.998	NA	ec.y	body.adv
AdvVarianceOfSlope	-0.03	0.09	0.762	NA	ec.y	body.adv
Cnsrct.Intercept_Corr_Adv_Intercept	-0.06	0.02	0.000	-0.31	ec.y	body.adv
Cnsrct.Intercept_Corr_Adv_Slope	0.02	0.02	0.431	0.20	ec.y	body.adv
Cnsrct.Slope_Corr_Adv_Intercept	-0.02	0.02	0.361	-0.08	ec.y	body.adv
Cnsrct.Slope_Corr_Adv_Slope	-0.03	0.02	0.174	-0.40	ec.y	body.adv
ConstructInterceptOfIntercept	3.44	0.02	0.000	7.63	ec.y	romantic.adv
ConstructVarianceOfIntercept	0.20	0.02	0.000	1.00	ec.y	romantic.adv
ConstructInterceptOfSlope	0.09	0.02	0.000	0.23	ec.y	romantic.adv
ConstructVarianceOfSlope	0.15	0.04	0.000	1.00	ec.y	romantic.adv
AdvInterceptOfIntercept	1.38	0.02	0.000	3.37	ec.y	romantic.adv
AdvVarianceOfIntercept	0.17	0.03	0.000	1.00	ec.y	romantic.adv
AdvInterceptOfSlope	0.03	0.03	0.299	0.11	ec.y	romantic.adv
AdvVarianceOfSlope	0.08	0.06	0.205	1.00	ec.y	romantic.adv
Cnsrct.Intercept_Corr_Adv_Intercept	-0.07	0.01	0.000	-0.35	ec.y	romantic.adv
Cnsrct.Intercept_Corr_Adv_Slope	0.01	0.02	0.453	0.09	ec.y	romantic.adv
Cnsrct.Slope_Corr_Adv_Intercept	0.01	0.01	0.430	0.07	ec.y	romantic.adv
Cnsrct.Slope_Corr_Adv_Slope	-0.03	0.02	0.086	-0.27	ec.y	romantic.adv

ConstructInterceptOfIntercept	3.44	0.02	0.000	7.50	ec.y	neighborhood.adv
ConstructVarianceOfIntercept	0.21	0.02	0.000	1.00	ec.y	neighborhood.adv
ConstructInterceptOfSlope	0.09	0.02	0.000	0.23	ec.y	neighborhood.adv
ConstructVarianceOfSlope	0.16	0.04	0.000	1.00	ec.y	neighborhood.adv
AdvInterceptOfIntercept	1.52	0.03	0.000	3.51	ec.y	neighborhood.adv
AdvVarianceOfIntercept	0.19	0.04	0.000	1.00	ec.y	neighborhood.adv
AdvInterceptOfSlope	0.02	0.03	0.543	0.08	ec.y	neighborhood.adv
AdvVarianceOfSlope	0.08	0.08	0.335	1.00	ec.y	neighborhood.adv
Cnsrct.Intercept_Corr_Adv_Intercept	-0.03	0.01	0.018	-0.17	ec.y	neighborhood.adv
Cnsrct.Intercept_Corr_Adv_Slope	0.00	0.02	0.851	0.03	ec.y	neighborhood.adv
Cnsrct.Slope_Corr_Adv_Intercept	-0.01	0.02	0.372	-0.08	ec.y	neighborhood.adv
Cnsrct.Slope_Corr_Adv_Slope	-0.01	0.02	0.578	-0.10	ec.y	neighborhood.adv
ConstructInterceptOfIntercept	3.44	0.02	0.000	7.42	ec.y	health.self.adv
ConstructVarianceOfIntercept	0.21	0.02	0.000	1.00	ec.y	health.self.adv
ConstructInterceptOfSlope	0.09	0.02	0.000	0.22	ec.y	health.self.adv
ConstructVarianceOfSlope	0.18	0.04	0.000	1.00	ec.y	health.self.adv
AdvInterceptOfIntercept	1.49	0.03	0.000	3.84	ec.y	health.self.adv
AdvVarianceOfIntercept	0.15	0.04	0.000	1.00	ec.y	health.self.adv
AdvInterceptOfSlope	-0.09	0.03	0.006	-0.66	ec.y	health.self.adv
AdvVarianceOfSlope	0.02	0.08	0.795	1.00	ec.y	health.self.adv
Cnsrct.Intercept_Corr_Adv_Intercept	-0.02	0.01	0.268	-0.09	ec.y	health.self.adv
Cnsrct.Intercept_Corr_Adv_Slope	0.00	0.02	0.812	0.07	ec.y	health.self.adv
Cnsrct.Slope_Corr_Adv_Intercept	-0.03	0.02	0.100	-0.16	ec.y	health.self.adv
Cnsrct.Slope_Corr_Adv_Slope	0.03	0.02	0.125	0.51	ec.y	health.self.adv
ConstructInterceptOfIntercept	3.44	0.02	0.000	7.43	ec.y	health.other.adv
ConstructVarianceOfIntercept	0.21	0.02	0.000	1.00	ec.y	health.other.adv
ConstructInterceptOfSlope	0.09	0.02	0.000	0.22	ec.y	health.other.adv
ConstructVarianceOfSlope	0.17	0.04	0.000	1.00	ec.y	health.other.adv
AdvInterceptOfIntercept	2.08	0.04	0.000	3.47	ec.y	health.other.adv
AdvVarianceOfIntercept	0.36	0.09	0.000	1.00	ec.y	health.other.adv
AdvInterceptOfSlope	-0.27	0.05	0.000	-0.59	ec.y	health.other.adv
AdvVarianceOfSlope	0.21	0.18	0.267	1.00	ec.y	health.other.adv
Cnsrct.Intercept_Corr_Adv_Intercept	-0.04	0.02	0.051	-0.15	ec.y	health.other.adv

Cnsrct.Intercept_Corr_Adv_Slope	0.07	0.03	0.023	0.31	ec.y	health.other.adv
Cnsrct.Slope_Corr_Adv_Intercept	0.00	0.02	0.871	0.01	ec.y	health.other.adv
Cnsrct.Slope_Corr_Adv_Slope	-0.06	0.03	0.063	-0.31	ec.y	health.other.adv
ConstructInterceptOfIntercept	3.44	0.02	0.000	7.42	ec.y	finance.adv
ConstructVarianceOfIntercept	0.22	0.02	0.000	1.00	ec.y	finance.adv
ConstructInterceptOfSlope	0.09	0.02	0.000	0.22	ec.y	finance.adv
ConstructVarianceOfSlope	0.18	0.04	0.000	1.00	ec.y	finance.adv
AdvInterceptOfIntercept	1.53	0.03	0.000	3.74	ec.y	finance.adv
AdvVarianceOfIntercept	0.17	0.04	0.000	1.00	ec.y	finance.adv
AdvInterceptOfSlope	-0.07	0.04	0.079	-0.17	ec.y	finance.adv
AdvVarianceOfSlope	0.16	0.10	0.092	1.00	ec.y	finance.adv
Cnsrct.Intercept_Corr_Adv_Intercept	-0.06	0.02	0.000	-0.33	ec.y	finance.adv
Cnsrct.Intercept_Corr_Adv_Slope	0.02	0.02	0.300	0.12	ec.y	finance.adv
Cnsrct.Slope_Corr_Adv_Intercept	-0.03	0.02	0.055	-0.18	ec.y	finance.adv
Cnsrct.Slope_Corr_Adv_Slope	0.01	0.02	0.585	0.07	ec.y	finance.adv
ConstructInterceptOfIntercept	3.44	0.02	0.000	7.44	ec.y	legal.adv
ConstructVarianceOfIntercept	0.21	0.02	0.000	1.00	ec.y	legal.adv
ConstructInterceptOfSlope	0.09	0.02	0.000	0.22	ec.y	legal.adv
ConstructVarianceOfSlope	0.17	0.04	0.000	1.00	ec.y	legal.adv
AdvInterceptOfIntercept	1.54	0.03	0.000	4.13	ec.y	legal.adv
AdvVarianceOfIntercept	0.14	0.06	0.017	1.00	ec.y	legal.adv
AdvInterceptOfSlope	-0.19	0.04	0.000	-0.67	ec.y	legal.adv
AdvVarianceOfSlope	0.08	0.12	0.518	1.00	ec.y	legal.adv
Cnsrct.Intercept_Corr_Adv_Intercept	-0.06	0.02	0.002	-0.32	ec.y	legal.adv
Cnsrct.Intercept_Corr_Adv_Slope	0.02	0.02	0.339	0.17	ec.y	legal.adv
Cnsrct.Slope_Corr_Adv_Intercept	0.01	0.02	0.577	0.07	ec.y	legal.adv
Cnsrct.Slope_Corr_Adv_Slope	-0.02	0.03	0.351	-0.21	ec.y	legal.adv
ConstructInterceptOfIntercept	3.44	0.02	0.000	7.46	ec.y	violence.adv
ConstructVarianceOfIntercept	0.21	0.02	0.000	1.00	ec.y	violence.adv
ConstructInterceptOfSlope	0.09	0.02	0.000	0.21	ec.y	violence.adv
ConstructVarianceOfSlope	0.18	0.04	0.000	1.00	ec.y	violence.adv
AdvInterceptOfIntercept	1.44	0.03	0.000	3.67	ec.y	violence.adv
AdvVarianceOfIntercept	0.15	0.04	0.000	1.00	ec.y	violence.adv

AdvInterceptOfSlope	-0.23	0.03	0.000	-0.75	ec.y	violence.adv
AdvVarianceOfSlope	0.09	0.08	0.250	1.00	ec.y	violence.adv
Cnsrct.Intercept_Corr_Adv_Intercept	-0.04	0.02	0.004	-0.24	ec.y	violence.adv
Cnsrct.Intercept_Corr_Adv_Slope	0.05	0.02	0.014	0.33	ec.y	violence.adv
Cnsrct.Slope_Corr_Adv_Intercept	0.01	0.02	0.473	0.08	ec.y	violence.adv
**Cnsrct.Slope_Corr_Adv_Slope**	**-0.05**	**0.02**	**0.025**	**-0.37**	**ec.y**	**violence.adv**
ConstructInterceptOfIntercept	3.44	0.02	0.000	7.49	ec.y	discrimination.adv
ConstructVarianceOfIntercept	0.21	0.02	0.000	1.00	ec.y	discrimination.adv
ConstructInterceptOfSlope	0.09	0.02	0.000	0.22	ec.y	discrimination.adv
ConstructVarianceOfSlope	0.17	0.04	0.000	1.00	ec.y	discrimination.adv
AdvInterceptOfIntercept	1.22	0.02	0.000	5.13	ec.y	discrimination.adv
AdvVarianceOfIntercept	0.06	0.02	0.003	1.00	ec.y	discrimination.adv
AdvInterceptOfSlope	-0.01	0.03	0.815	-0.02	ec.y	discrimination.adv
**AdvVarianceOfSlope**	**0.13**	**0.04**	**0.002**	**1.00**	**ec.y**	**discrimination.adv**
Cnsrct.Intercept_Corr_Adv_Intercept	-0.03	0.01	0.002	-0.29	ec.y	discrimination.adv
Cnsrct.Intercept_Corr_Adv_Slope	0.03	0.01	0.047	0.17	ec.y	discrimination.adv
Cnsrct.Slope_Corr_Adv_Intercept	0.00	0.01	0.740	0.04	ec.y	discrimination.adv
Cnsrct.Slope_Corr_Adv_Slope	-0.02	0.02	0.144	-0.15	ec.y	discrimination.adv
ConstructInterceptOfIntercept	3.44	0.02	0.000	7.42	ec.y	activities.adv
ConstructVarianceOfIntercept	0.21	0.02	0.000	1.00	ec.y	activities.adv
ConstructInterceptOfSlope	0.09	0.02	0.000	0.22	ec.y	activities.adv
ConstructVarianceOfSlope	0.17	0.04	0.000	1.00	ec.y	activities.adv
AdvInterceptOfIntercept	1.35	0.02	0.000	5.95	ec.y	activities.adv
AdvVarianceOfIntercept	0.05	0.02	0.010	1.00	ec.y	activities.adv
AdvInterceptOfSlope	-0.10	0.02	0.000	-2.11	ec.y	activities.adv
AdvVarianceOfSlope	0.00	0.04	0.957	1.00	ec.y	activities.adv
Cnsrct.Intercept_Corr_Adv_Intercept	-0.03	0.01	0.002	-0.28	ec.y	activities.adv
Cnsrct.Intercept_Corr_Adv_Slope	0.02	0.01	0.068	1.09	ec.y	activities.adv
Cnsrct.Slope_Corr_Adv_Intercept	-0.01	0.01	0.421	-0.09	ec.y	activities.adv
Cnsrct.Slope_Corr_Adv_Slope	-0.02	0.01	0.260	-0.78	ec.y	activities.adv
ConstructInterceptOfIntercept	3.25	0.02	0.000	6.26	ec.p	academic.adv
ConstructVarianceOfIntercept	0.27	0.02	0.000	1.00	ec.p	academic.adv
ConstructInterceptOfSlope	0.11	0.02	0.000	0.30	ec.p	academic.adv

ConstructVarianceOfSlope	0.12	0.03	0.000	1.00	ec.p	academic.adv
AdvInterceptOfIntercept	1.79	0.03	0.000	3.38	ec.p	academic.adv
AdvVarianceOfIntercept	0.28	0.05	0.000	1.00	ec.p	academic.adv
AdvInterceptOfSlope	0.05	0.04	0.164	0.12	ec.p	academic.adv
AdvVarianceOfSlope	0.17	0.09	0.070	1.00	ec.p	academic.adv
Cnsrct.Intercept_Corr_Adv_Intercept	-0.14	0.02	0.000	-0.50	ec.p	academic.adv
Cnsrct.Intercept_Corr_Adv_Slope	0.02	0.02	0.290	0.10	ec.p	academic.adv
Cnsrct.Slope_Corr_Adv_Intercept	0.01	0.01	0.661	0.03	ec.p	academic.adv
**Cnsrct.Slope_Corr_Adv_Slope**	**-0.07**	**0.02**	**0.000**	**-0.50**	**ec.p**	**academic.adv**
ConstructInterceptOfIntercept	3.25	0.02	0.000	6.24	ec.p	behavioral.adv
ConstructVarianceOfIntercept	0.27	0.02	0.000	1.00	ec.p	behavioral.adv
ConstructInterceptOfSlope	0.11	0.02	0.000	0.30	ec.p	behavioral.adv
ConstructVarianceOfSlope	0.13	0.03	0.000	1.00	ec.p	behavioral.adv
AdvInterceptOfIntercept	1.42	0.03	0.000	3.14	ec.p	behavioral.adv
AdvVarianceOfIntercept	0.20	0.03	0.000	1.00	ec.p	behavioral.adv
AdvInterceptOfSlope	-0.25	0.03	0.000	-1.01	ec.p	behavioral.adv
AdvVarianceOfSlope	0.06	0.07	0.390	1.00	ec.p	behavioral.adv
Cnsrct.Intercept_Corr_Adv_Intercept	-0.07	0.01	0.000	-0.31	ec.p	behavioral.adv
Cnsrct.Intercept_Corr_Adv_Slope	0.01	0.02	0.720	0.05	ec.p	behavioral.adv
Cnsrct.Slope_Corr_Adv_Intercept	0.00	0.01	0.995	0.00	ec.p	behavioral.adv
Cnsrct.Slope_Corr_Adv_Slope	-0.01	0.01	0.318	-0.17	ec.p	behavioral.adv
ConstructInterceptOfIntercept	3.25	0.02	0.000	6.18	ec.p	peer.adv
ConstructVarianceOfIntercept	0.28	0.02	0.000	1.00	ec.p	peer.adv
ConstructInterceptOfSlope	0.11	0.02	0.000	0.28	ec.p	peer.adv
ConstructVarianceOfSlope	0.14	0.03	0.000	1.00	ec.p	peer.adv
AdvInterceptOfIntercept	1.91	0.03	0.000	3.50	ec.p	peer.adv
AdvVarianceOfIntercept	0.30	0.04	0.000	1.00	ec.p	peer.adv
AdvInterceptOfSlope	-0.22	0.04	0.000	-0.54	ec.p	peer.adv
AdvVarianceOfSlope	0.16	0.08	0.055	1.00	ec.p	peer.adv
Cnsrct.Intercept_Corr_Adv_Intercept	-0.10	0.02	0.000	-0.35	ec.p	peer.adv
Cnsrct.Intercept_Corr_Adv_Slope	0.06	0.02	0.003	0.27	ec.p	peer.adv
Cnsrct.Slope_Corr_Adv_Intercept	0.03	0.01	0.019	0.16	ec.p	peer.adv
**Cnsrct.Slope_Corr_Adv_Slope**	**-0.07**	**0.02**	**0.000**	**-0.43**	**ec.p**	**peer.adv**

ConstructInterceptOfIntercept	3.25	0.02	0.000	6.18	ec.p	parentchild.relationship.adv
ConstructVarianceOfIntercept	0.28	0.02	0.000	1.00	ec.p	parentchild.relationship.adv
ConstructInterceptOfSlope	0.11	0.02	0.000	0.29	ec.p	parentchild.relationship.adv
ConstructVarianceOfSlope	0.14	0.03	0.000	1.00	ec.p	parentchild.relationship.adv
AdvInterceptOfIntercept	1.95	0.03	0.000	4.04	ec.p	parentchild.relationship.adv
AdvVarianceOfIntercept	0.23	0.04	0.000	1.00	ec.p	parentchild.relationship.adv
AdvInterceptOfSlope	-0.05	0.04	0.162	-0.11	ec.p	parentchild.relationship.adv
**AdvVarianceOfSlope**	**0.22**	**0.09**	**0.015**	**1.00**	**ec.p**	**parentchild.relationship.adv**
Cnsrct.Intercept_Corr_Adv_Intercept	-0.07	0.02	0.000	-0.28	ec.p	parentchild.relationship.adv
Cnsrct.Intercept_Corr_Adv_Slope	0.00	0.02	0.837	-0.02	ec.p	parentchild.relationship.adv
Cnsrct.Slope_Corr_Adv_Intercept	0.00	0.01	0.909	0.01	ec.p	parentchild.relationship.adv
Cnsrct.Slope_Corr_Adv_Slope	-0.02	0.02	0.181	-0.13	ec.p	parentchild.relationship.adv
ConstructInterceptOfIntercept	3.25	0.02	0.000	6.13	ec.p	parentparent.relationship.adv
ConstructVarianceOfIntercept	0.28	0.02	0.000	1.00	ec.p	parentparent.relationship.adv
ConstructInterceptOfSlope	0.11	0.02	0.000	0.28	ec.p	parentparent.relationship.adv
ConstructVarianceOfSlope	0.15	0.03	0.000	1.00	ec.p	parentparent.relationship.adv
AdvInterceptOfIntercept	1.85	0.03	0.000	3.06	ec.p	parentparent.relationship.adv
AdvVarianceOfIntercept	0.36	0.06	0.000	1.00	ec.p	parentparent.relationship.adv
AdvInterceptOfSlope	-0.11	0.04	0.008	-0.17	ec.p	parentparent.relationship.adv
**AdvVarianceOfSlope**	**0.44**	**0.11**	**0.000**	**1.00**	**ec.p**	**parentparent.relationship.adv**
Cnsrct.Intercept_Corr_Adv_Intercept	-0.04	0.02	0.025	-0.12	ec.p	parentparent.relationship.adv
Cnsrct.Intercept_Corr_Adv_Slope	0.04	0.02	0.102	0.11	ec.p	parentparent.relationship.adv
**Cnsrct.Slope_Corr_Adv_Intercept**	**-0.03**	**0.01**	**0.042**	**-0.13**	**ec.p**	**parentparent.relationship.adv**
Cnsrct.Slope_Corr_Adv_Slope	-0.02	0.02	0.295	-0.08	ec.p	parentparent.relationship.adv
ConstructInterceptOfIntercept	3.25	0.02	0.000	6.14	ec.p	household.relationship.adv
ConstructVarianceOfIntercept	0.28	0.02	0.000	1.00	ec.p	household.relationship.adv
ConstructInterceptOfSlope	0.11	0.02	0.000	0.28	ec.p	household.relationship.adv
ConstructVarianceOfSlope	0.15	0.03	0.000	1.00	ec.p	household.relationship.adv
AdvInterceptOfIntercept	2.06	0.03	0.000	4.42	ec.p	household.relationship.adv
AdvVarianceOfIntercept	0.22	0.04	0.000	1.00	ec.p	household.relationship.adv
AdvInterceptOfSlope	-0.14	0.04	0.000	-0.45	ec.p	household.relationship.adv
AdvVarianceOfSlope	0.09	0.09	0.330	1.00	ec.p	household.relationship.adv
Cnsrct.Intercept_Corr_Adv_Intercept	-0.03	0.02	0.043	-0.13	ec.p	household.relationship.adv

Cnsrct.Intercept_Corr_Adv_Slope	-0.04	0.02	0.088	-0.23	ec.p	household.relationship.adv
Cnsrct.Slope_Corr_Adv_Intercept	0.00	0.01	0.825	0.02	ec.p	household.relationship.adv
Cnsrct.Slope_Corr_Adv_Slope	-0.01	0.02	0.676	-0.06	ec.p	household.relationship.adv
ConstructInterceptOfIntercept	3.25	0.02	0.000	6.14	ec.p	body.adv
ConstructVarianceOfIntercept	0.28	0.02	0.000	1.00	ec.p	body.adv
ConstructInterceptOfSlope	0.11	0.02	0.000	0.28	ec.p	body.adv
ConstructVarianceOfSlope	0.15	0.03	0.000	1.00	ec.p	body.adv
AdvInterceptOfIntercept	1.59	0.03	0.000	3.63	ec.p	body.adv
AdvVarianceOfIntercept	0.19	0.04	0.000	1.00	ec.p	body.adv
AdvInterceptOfSlope	0.00	0.03	0.985	NA	ec.p	body.adv
AdvVarianceOfSlope	-0.03	0.09	0.751	NA	ec.p	body.adv
Cnsrct.Intercept_Corr_Adv_Intercept	0.01	0.02	0.409	0.05	ec.p	body.adv
Cnsrct.Intercept_Corr_Adv_Slope	-0.02	0.02	0.375	-0.19	ec.p	body.adv
Cnsrct.Slope_Corr_Adv_Intercept	0.00	0.01	0.816	-0.02	ec.p	body.adv
**Cnsrct.Slope_Corr_Adv_Slope**	**-0.03**	**0.02**	**0.046**	**-0.50**	**ec.p**	**body.adv**
ConstructInterceptOfIntercept	3.25	0.02	0.000	6.15	ec.p	romantic.adv
ConstructVarianceOfIntercept	0.28	0.02	0.000	1.00	ec.p	romantic.adv
ConstructInterceptOfSlope	0.11	0.02	0.000	0.28	ec.p	romantic.adv
ConstructVarianceOfSlope	0.15	0.03	0.000	1.00	ec.p	romantic.adv
AdvInterceptOfIntercept	1.38	0.02	0.000	3.41	ec.p	romantic.adv
AdvVarianceOfIntercept	0.16	0.03	0.000	1.00	ec.p	romantic.adv
AdvInterceptOfSlope	0.03	0.03	0.288	0.13	ec.p	romantic.adv
AdvVarianceOfSlope	0.06	0.06	0.352	1.00	ec.p	romantic.adv
Cnsrct.Intercept_Corr_Adv_Intercept	-0.02	0.01	0.109	-0.10	ec.p	romantic.adv
Cnsrct.Intercept_Corr_Adv_Slope	0.00	0.02	0.921	0.01	ec.p	romantic.adv
Cnsrct.Slope_Corr_Adv_Intercept	0.00	0.01	0.719	-0.03	ec.p	romantic.adv
Cnsrct.Slope_Corr_Adv_Slope	0.01	0.01	0.623	0.07	ec.p	romantic.adv
ConstructInterceptOfIntercept	3.25	0.02	0.000	6.15	ec.p	neighborhood.adv
ConstructVarianceOfIntercept	0.28	0.02	0.000	1.00	ec.p	neighborhood.adv
ConstructInterceptOfSlope	0.11	0.02	0.000	0.28	ec.p	neighborhood.adv
ConstructVarianceOfSlope	0.15	0.03	0.000	1.00	ec.p	neighborhood.adv
AdvInterceptOfIntercept	1.52	0.03	0.000	3.52	ec.p	neighborhood.adv
AdvVarianceOfIntercept	0.19	0.04	0.000	1.00	ec.p	neighborhood.adv

AdvInterceptOfSlope	0.02	0.03	0.533	0.08	ec.p	neighborhood.adv
AdvVarianceOfSlope	0.07	0.08	0.360	1.00	ec.p	neighborhood.adv
Cnsrct.Intercept_Corr_Adv_Intercept	-0.01	0.01	0.397	-0.06	ec.p	neighborhood.adv
Cnsrct.Intercept_Corr_Adv_Slope	-0.02	0.02	0.253	-0.15	ec.p	neighborhood.adv
Cnsrct.Slope_Corr_Adv_Intercept	-0.01	0.01	0.386	-0.07	ec.p	neighborhood.adv
Cnsrct.Slope_Corr_Adv_Slope	0.01	0.02	0.566	0.09	ec.p	neighborhood.adv
ConstructInterceptOfIntercept	3.25	0.02	0.000	6.14	ec.p	health.self.adv
ConstructVarianceOfIntercept	0.28	0.02	0.000	1.00	ec.p	health.self.adv
ConstructInterceptOfSlope	0.11	0.02	0.000	0.28	ec.p	health.self.adv
ConstructVarianceOfSlope	0.15	0.03	0.000	1.00	ec.p	health.self.adv
AdvInterceptOfIntercept	1.49	0.03	0.000	3.80	ec.p	health.self.adv
AdvVarianceOfIntercept	0.15	0.04	0.000	1.00	ec.p	health.self.adv
AdvInterceptOfSlope	-0.10	0.03	0.006	-0.61	ec.p	health.self.adv
AdvVarianceOfSlope	0.02	0.08	0.758	1.00	ec.p	health.self.adv
Cnsrct.Intercept_Corr_Adv_Intercept	0.00	0.01	0.749	-0.02	ec.p	health.self.adv
Cnsrct.Intercept_Corr_Adv_Slope	0.01	0.02	0.769	0.07	ec.p	health.self.adv
Cnsrct.Slope_Corr_Adv_Intercept	0.00	0.01	0.719	-0.03	ec.p	health.self.adv
Cnsrct.Slope_Corr_Adv_Slope	-0.01	0.02	0.632	-0.13	ec.p	health.self.adv
ConstructInterceptOfIntercept	3.25	0.02	0.000	6.14	ec.p	health.other.adv
ConstructVarianceOfIntercept	0.28	0.02	0.000	1.00	ec.p	health.other.adv
ConstructInterceptOfSlope	0.11	0.02	0.000	0.28	ec.p	health.other.adv
ConstructVarianceOfSlope	0.15	0.03	0.000	1.00	ec.p	health.other.adv
AdvInterceptOfIntercept	2.08	0.04	0.000	3.44	ec.p	health.other.adv
AdvVarianceOfIntercept	0.37	0.09	0.000	1.00	ec.p	health.other.adv
AdvInterceptOfSlope	-0.27	0.05	0.000	-0.56	ec.p	health.other.adv
AdvVarianceOfSlope	0.22	0.18	0.224	1.00	ec.p	health.other.adv
Cnsrct.Intercept_Corr_Adv_Intercept	0.01	0.02	0.661	0.03	ec.p	health.other.adv
Cnsrct.Intercept_Corr_Adv_Slope	0.01	0.03	0.803	0.03	ec.p	health.other.adv
**Cnsrct.Slope_Corr_Adv_Intercept**	**-0.05**	**0.02**	**0.008**	**-0.21**	**ec.p**	**health.other.adv**
Cnsrct.Slope_Corr_Adv_Slope	0.04	0.03	0.157	0.19	ec.p	health.other.adv
ConstructInterceptOfIntercept	3.25	0.02	0.000	6.14	ec.p	finance.adv
ConstructVarianceOfIntercept	0.28	0.02	0.000	1.00	ec.p	finance.adv
ConstructInterceptOfSlope	0.11	0.02	0.000	0.28	ec.p	finance.adv

ConstructVarianceOfSlope	0.15	0.03	0.000	1.00	ec.p	finance.adv
AdvInterceptOfIntercept	1.53	0.03	0.000	3.66	ec.p	finance.adv
AdvVarianceOfIntercept	0.17	0.04	0.000	1.00	ec.p	finance.adv
AdvInterceptOfSlope	-0.07	0.04	0.085	-0.16	ec.p	finance.adv
AdvVarianceOfSlope	0.19	0.10	0.053	1.00	ec.p	finance.adv
Cnsrct.Intercept_Corr_Adv_Intercept	-0.03	0.02	0.049	-0.14	ec.p	finance.adv
Cnsrct.Intercept_Corr_Adv_Slope	-0.02	0.02	0.452	-0.07	ec.p	finance.adv
Cnsrct.Slope_Corr_Adv_Intercept	0.00	0.01	0.752	-0.03	ec.p	finance.adv
Cnsrct.Slope_Corr_Adv_Slope	0.02	0.02	0.264	0.12	ec.p	finance.adv
ConstructInterceptOfIntercept	3.25	0.02	0.000	6.11	ec.p	legal.adv
ConstructVarianceOfIntercept	0.28	0.02	0.000	1.00	ec.p	legal.adv
ConstructInterceptOfSlope	0.11	0.02	0.000	0.27	ec.p	legal.adv
ConstructVarianceOfSlope	0.15	0.03	0.000	1.00	ec.p	legal.adv
AdvInterceptOfIntercept	1.54	0.03	0.000	4.03	ec.p	legal.adv
AdvVarianceOfIntercept	0.15	0.06	0.012	1.00	ec.p	legal.adv
AdvInterceptOfSlope	-0.19	0.04	0.000	-0.63	ec.p	legal.adv
AdvVarianceOfSlope	0.09	0.12	0.466	1.00	ec.p	legal.adv
Cnsrct.Intercept_Corr_Adv_Intercept	-0.06	0.02	0.001	-0.28	ec.p	legal.adv
Cnsrct.Intercept_Corr_Adv_Slope	0.05	0.02	0.037	0.31	ec.p	legal.adv
Cnsrct.Slope_Corr_Adv_Intercept	0.01	0.02	0.551	0.06	ec.p	legal.adv
Cnsrct.Slope_Corr_Adv_Slope	-0.01	0.02	0.571	-0.10	ec.p	legal.adv
ConstructInterceptOfIntercept	3.25	0.02	0.000	6.13	ec.p	violence.adv
ConstructVarianceOfIntercept	0.28	0.02	0.000	1.00	ec.p	violence.adv
ConstructInterceptOfSlope	0.11	0.02	0.000	0.28	ec.p	violence.adv
ConstructVarianceOfSlope	0.15	0.03	0.000	1.00	ec.p	violence.adv
AdvInterceptOfIntercept	1.44	0.03	0.000	3.62	ec.p	violence.adv
AdvVarianceOfIntercept	0.16	0.04	0.000	1.00	ec.p	violence.adv
AdvInterceptOfSlope	-0.23	0.03	0.000	-0.75	ec.p	violence.adv
AdvVarianceOfSlope	0.09	0.08	0.242	1.00	ec.p	violence.adv
Cnsrct.Intercept_Corr_Adv_Intercept	-0.06	0.02	0.000	-0.30	ec.p	violence.adv
Cnsrct.Intercept_Corr_Adv_Slope	0.04	0.02	0.056	0.23	ec.p	violence.adv
Cnsrct.Slope_Corr_Adv_Intercept	0.02	0.01	0.259	0.10	ec.p	violence.adv
**Cnsrct.Slope_Corr_Adv_Slope**	**-0.04**	**0.02**	**0.026**	**-0.31**	**ec.p**	**violence.adv**

ConstructInterceptOfIntercept	3.25	0.02	0.000	6.13	ec.p	discrimination.adv
ConstructVarianceOfIntercept	0.28	0.02	0.000	1.00	ec.p	discrimination.adv
ConstructInterceptOfSlope	0.11	0.02	0.000	0.28	ec.p	discrimination.adv
ConstructVarianceOfSlope	0.15	0.03	0.000	1.00	ec.p	discrimination.adv
AdvInterceptOfIntercept	1.22	0.02	0.000	5.03	ec.p	discrimination.adv
AdvVarianceOfIntercept	0.06	0.02	0.002	1.00	ec.p	discrimination.adv
AdvInterceptOfSlope	-0.01	0.03	0.829	-0.02	ec.p	discrimination.adv
**AdvVarianceOfSlope**	**0.13**	**0.04**	**0.002**	**1.00**	**ec.p**	**discrimination.adv**
Cnsrct.Intercept_Corr_Adv_Intercept	0.01	0.01	0.466	0.06	ec.p	discrimination.adv
Cnsrct.Intercept_Corr_Adv_Slope	-0.01	0.01	0.647	-0.03	ec.p	discrimination.adv
Cnsrct.Slope_Corr_Adv_Intercept	0.00	0.01	0.767	-0.03	ec.p	discrimination.adv
Cnsrct.Slope_Corr_Adv_Slope	-0.01	0.01	0.338	-0.08	ec.p	discrimination.adv
ConstructInterceptOfIntercept	3.25	0.02	0.000	6.14	ec.p	activities.adv
ConstructVarianceOfIntercept	0.28	0.02	0.000	1.00	ec.p	activities.adv
ConstructInterceptOfSlope	0.11	0.02	0.000	0.28	ec.p	activities.adv
ConstructVarianceOfSlope	0.15	0.03	0.000	1.00	ec.p	activities.adv
AdvInterceptOfIntercept	1.35	0.02	0.000	5.86	ec.p	activities.adv
AdvVarianceOfIntercept	0.05	0.02	0.009	1.00	ec.p	activities.adv
AdvInterceptOfSlope	-0.10	0.02	0.000	-1.21	ec.p	activities.adv
AdvVarianceOfSlope	0.01	0.04	0.869	1.00	ec.p	activities.adv
Cnsrct.Intercept_Corr_Adv_Intercept	0.00	0.01	0.889	0.01	ec.p	activities.adv
Cnsrct.Intercept_Corr_Adv_Slope	0.01	0.01	0.612	0.15	ec.p	activities.adv
Cnsrct.Slope_Corr_Adv_Intercept	-0.01	0.01	0.533	-0.06	ec.p	activities.adv
Cnsrct.Slope_Corr_Adv_Slope	0.00	0.01	0.779	-0.10	ec.p	activities.adv
ConstructInterceptOfIntercept	3.38	0.02	0.000	8.66	ne.y	academic.adv
ConstructVarianceOfIntercept	0.15	0.02	0.000	1.00	ne.y	academic.adv
ConstructInterceptOfSlope	0.28	0.03	0.000	0.72	ne.y	academic.adv
ConstructVarianceOfSlope	0.15	0.04	0.000	1.00	ne.y	academic.adv
AdvInterceptOfIntercept	1.79	0.03	0.000	3.50	ne.y	academic.adv
AdvVarianceOfIntercept	0.26	0.05	0.000	1.00	ne.y	academic.adv
AdvInterceptOfSlope	0.05	0.04	0.133	0.15	ne.y	academic.adv
AdvVarianceOfSlope	0.13	0.10	0.185	1.00	ne.y	academic.adv
Cnsrct.Intercept_Corr_Adv_Intercept	-0.05	0.01	0.000	-0.25	ne.y	academic.adv

Cnsrct.Intercept_Corr_Adv_Slope	0.02	0.02	0.324	0.13	ne.y	academic.adv
Cnsrct.Slope_Corr_Adv_Intercept	0.03	0.02	0.048	0.17	ne.y	academic.adv
**Cnsrct.Slope_Corr_Adv_Slope**	**-0.08**	**0.02**	**0.000**	**-0.57**	**ne.y**	**academic.adv**
ConstructInterceptOfIntercept	3.38	0.02	0.000	8.61	ne.y	behavioral.adv
ConstructVarianceOfIntercept	0.15	0.02	0.000	1.00	ne.y	behavioral.adv
ConstructInterceptOfSlope	0.28	0.03	0.000	0.72	ne.y	behavioral.adv
ConstructVarianceOfSlope	0.16	0.04	0.000	1.00	ne.y	behavioral.adv
AdvInterceptOfIntercept	1.42	0.03	0.000	3.19	ne.y	behavioral.adv
AdvVarianceOfIntercept	0.20	0.04	0.000	1.00	ne.y	behavioral.adv
AdvInterceptOfSlope	-0.25	0.03	0.000	-1.23	ne.y	behavioral.adv
AdvVarianceOfSlope	0.04	0.07	0.576	1.00	ne.y	behavioral.adv
Cnsrct.Intercept_Corr_Adv_Intercept	-0.04	0.01	0.005	-0.21	ne.y	behavioral.adv
Cnsrct.Intercept_Corr_Adv_Slope	0.03	0.02	0.050	0.40	ne.y	behavioral.adv
Cnsrct.Slope_Corr_Adv_Intercept	0.02	0.02	0.352	0.09	ne.y	behavioral.adv
Cnsrct.Slope_Corr_Adv_Slope	-0.02	0.02	0.230	-0.30	ne.y	behavioral.adv
ConstructInterceptOfIntercept	3.38	0.02	0.000	8.53	ne.y	peer.adv
ConstructVarianceOfIntercept	0.16	0.02	0.000	1.00	ne.y	peer.adv
ConstructInterceptOfSlope	0.28	0.03	0.000	0.71	ne.y	peer.adv
ConstructVarianceOfSlope	0.16	0.04	0.000	1.00	ne.y	peer.adv
AdvInterceptOfIntercept	1.90	0.03	0.000	3.55	ne.y	peer.adv
AdvVarianceOfIntercept	0.29	0.04	0.000	1.00	ne.y	peer.adv
AdvInterceptOfSlope	-0.22	0.04	0.000	-0.59	ne.y	peer.adv
AdvVarianceOfSlope	0.13	0.09	0.116	1.00	ne.y	peer.adv
Cnsrct.Intercept_Corr_Adv_Intercept	-0.08	0.01	0.000	-0.39	ne.y	peer.adv
Cnsrct.Intercept_Corr_Adv_Slope	0.06	0.02	0.002	0.40	ne.y	peer.adv
Cnsrct.Slope_Corr_Adv_Intercept	0.02	0.02	0.361	0.08	ne.y	peer.adv
**Cnsrct.Slope_Corr_Adv_Slope**	**-0.07**	**0.02**	**0.001**	**-0.51**	**ne.y**	**peer.adv**
ConstructInterceptOfIntercept	3.38	0.02	0.000	8.58	ne.y	parentchild.relationship.adv
ConstructVarianceOfIntercept	0.16	0.02	0.000	1.00	ne.y	parentchild.relationship.adv
ConstructInterceptOfSlope	0.28	0.03	0.000	0.71	ne.y	parentchild.relationship.adv
ConstructVarianceOfSlope	0.15	0.04	0.000	1.00	ne.y	parentchild.relationship.adv
AdvInterceptOfIntercept	1.95	0.03	0.000	4.13	ne.y	parentchild.relationship.adv
AdvVarianceOfIntercept	0.22	0.04	0.000	1.00	ne.y	parentchild.relationship.adv

AdvInterceptOfSlope	-0.05	0.04	0.180	-0.11	ne.y	parentchild.relationship.adv
**AdvVarianceOfSlope**	**0.20**	**0.09**	**0.027**	**1.00**	**ne.y**	**parentchild.relationship.adv**
Cnsrct.Intercept_Corr_Adv_Intercept	-0.05	0.01	0.000	-0.28	ne.y	parentchild.relationship.adv
Cnsrct.Intercept_Corr_Adv_Slope	0.02	0.02	0.284	0.11	ne.y	parentchild.relationship.adv
Cnsrct.Slope_Corr_Adv_Intercept	-0.01	0.02	0.460	-0.07	ne.y	parentchild.relationship.adv
**Cnsrct.Slope_Corr_Adv_Slope**	**-0.07**	**0.02**	**0.002**	**-0.39**	**ne.y**	**parentchild.relationship.adv**
ConstructInterceptOfIntercept	3.38	0.02	0.000	8.62	ne.y	parentparent.relationship.adv
ConstructVarianceOfIntercept	0.15	0.02	0.000	1.00	ne.y	parentparent.relationship.adv
ConstructInterceptOfSlope	0.28	0.03	0.000	0.72	ne.y	parentparent.relationship.adv
ConstructVarianceOfSlope	0.15	0.04	0.000	1.00	ne.y	parentparent.relationship.adv
AdvInterceptOfIntercept	1.84	0.03	0.000	3.04	ne.y	parentparent.relationship.adv
AdvVarianceOfIntercept	0.37	0.06	0.000	1.00	ne.y	parentparent.relationship.adv
AdvInterceptOfSlope	-0.11	0.04	0.010	-0.16	ne.y	parentparent.relationship.adv
**AdvVarianceOfSlope**	**0.44**	**0.11**	**0.000**	**1.00**	**ne.y**	**parentparent.relationship.adv**
Cnsrct.Intercept_Corr_Adv_Intercept	-0.04	0.02	0.007	-0.18	ne.y	parentparent.relationship.adv
Cnsrct.Intercept_Corr_Adv_Slope	0.02	0.02	0.470	0.06	ne.y	parentparent.relationship.adv
Cnsrct.Slope_Corr_Adv_Intercept	0.01	0.02	0.647	0.04	ne.y	parentparent.relationship.adv
Cnsrct.Slope_Corr_Adv_Slope	-0.03	0.03	0.199	-0.13	ne.y	parentparent.relationship.adv
ConstructInterceptOfIntercept	3.38	0.02	0.000	8.61	ne.y	household.relationship.adv
ConstructVarianceOfIntercept	0.15	0.02	0.000	1.00	ne.y	household.relationship.adv
ConstructInterceptOfSlope	0.28	0.03	0.000	0.72	ne.y	household.relationship.adv
ConstructVarianceOfSlope	0.16	0.04	0.000	1.00	ne.y	household.relationship.adv
AdvInterceptOfIntercept	2.06	0.03	0.000	4.47	ne.y	household.relationship.adv
AdvVarianceOfIntercept	0.21	0.04	0.000	1.00	ne.y	household.relationship.adv
AdvInterceptOfSlope	-0.13	0.04	0.000	-0.47	ne.y	household.relationship.adv
AdvVarianceOfSlope	0.08	0.09	0.378	1.00	ne.y	household.relationship.adv
Cnsrct.Intercept_Corr_Adv_Intercept	-0.05	0.01	0.001	-0.28	ne.y	household.relationship.adv
Cnsrct.Intercept_Corr_Adv_Slope	0.01	0.02	0.653	0.08	ne.y	household.relationship.adv
Cnsrct.Slope_Corr_Adv_Intercept	0.02	0.02	0.381	0.08	ne.y	household.relationship.adv
**Cnsrct.Slope_Corr_Adv_Slope**	**-0.05**	**0.02**	**0.041**	**-0.41**	**ne.y**	**household.relationship.adv**
ConstructInterceptOfIntercept	3.38	0.02	0.000	8.77	ne.y	body.adv
ConstructVarianceOfIntercept	0.15	0.02	0.000	1.00	ne.y	body.adv
ConstructInterceptOfSlope	0.28	0.03	0.000	0.75	ne.y	body.adv

ConstructVarianceOfSlope	0.14	0.04	0.000	1.00	ne.y	body.adv
AdvInterceptOfIntercept	1.59	0.03	0.000	3.60	ne.y	body.adv
AdvVarianceOfIntercept	0.20	0.04	0.000	1.00	ne.y	body.adv
AdvInterceptOfSlope	0.00	0.03	0.962	NA	ne.y	body.adv
AdvVarianceOfSlope	-0.01	0.09	0.938	NA	ne.y	body.adv
Cnsrct.Intercept_Corr_Adv_Intercept	-0.05	0.01	0.000	-0.30	ne.y	body.adv
Cnsrct.Intercept_Corr_Adv_Slope	0.02	0.02	0.204	0.71	ne.y	body.adv
Cnsrct.Slope_Corr_Adv_Intercept	-0.03	0.02	0.102	-0.17	ne.y	body.adv
Cnsrct.Slope_Corr_Adv_Slope	-0.04	0.02	0.068	-1.23	ne.y	body.adv
ConstructInterceptOfIntercept	3.38	0.02	0.000	8.69	ne.y	romantic.adv
ConstructVarianceOfIntercept	0.15	0.02	0.000	1.00	ne.y	romantic.adv
ConstructInterceptOfSlope	0.28	0.03	0.000	0.74	ne.y	romantic.adv
ConstructVarianceOfSlope	0.15	0.04	0.000	1.00	ne.y	romantic.adv
AdvInterceptOfIntercept	1.38	0.02	0.000	3.38	ne.y	romantic.adv
AdvVarianceOfIntercept	0.17	0.03	0.000	1.00	ne.y	romantic.adv
AdvInterceptOfSlope	0.03	0.03	0.284	0.12	ne.y	romantic.adv
AdvVarianceOfSlope	0.07	0.06	0.290	1.00	ne.y	romantic.adv
Cnsrct.Intercept_Corr_Adv_Intercept	-0.01	0.01	0.385	-0.06	ne.y	romantic.adv
Cnsrct.Intercept_Corr_Adv_Slope	0.00	0.02	0.882	-0.02	ne.y	romantic.adv
**Cnsrct.Slope_Corr_Adv_Intercept**	**-0.03**	**0.01**	**0.047**	**-0.19**	**ne.y**	**romantic.adv**
Cnsrct.Slope_Corr_Adv_Slope	0.01	0.02	0.455	0.14	ne.y	romantic.adv
ConstructInterceptOfIntercept	3.38	0.02	0.000	8.62	ne.y	neighborhood.adv
ConstructVarianceOfIntercept	0.15	0.02	0.000	1.00	ne.y	neighborhood.adv
ConstructInterceptOfSlope	0.28	0.03	0.000	0.71	ne.y	neighborhood.adv
ConstructVarianceOfSlope	0.16	0.04	0.000	1.00	ne.y	neighborhood.adv
AdvInterceptOfIntercept	1.52	0.03	0.000	3.50	ne.y	neighborhood.adv
AdvVarianceOfIntercept	0.19	0.04	0.000	1.00	ne.y	neighborhood.adv
AdvInterceptOfSlope	0.02	0.03	0.532	0.08	ne.y	neighborhood.adv
AdvVarianceOfSlope	0.08	0.08	0.329	1.00	ne.y	neighborhood.adv
Cnsrct.Intercept_Corr_Adv_Intercept	-0.01	0.01	0.444	-0.06	ne.y	neighborhood.adv
Cnsrct.Intercept_Corr_Adv_Slope	0.00	0.02	0.884	0.02	ne.y	neighborhood.adv
Cnsrct.Slope_Corr_Adv_Intercept	-0.02	0.02	0.293	-0.10	ne.y	neighborhood.adv
Cnsrct.Slope_Corr_Adv_Slope	-0.02	0.02	0.463	-0.14	ne.y	neighborhood.adv

ConstructInterceptOfIntercept	3.38	0.02	0.000	8.63	ne.y	health.self.adv
ConstructVarianceOfIntercept	0.15	0.02	0.000	1.00	ne.y	health.self.adv
ConstructInterceptOfSlope	0.28	0.03	0.000	0.72	ne.y	health.self.adv
ConstructVarianceOfSlope	0.15	0.04	0.000	1.00	ne.y	health.self.adv
AdvInterceptOfIntercept	1.49	0.03	0.000	3.83	ne.y	health.self.adv
AdvVarianceOfIntercept	0.15	0.04	0.000	1.00	ne.y	health.self.adv
AdvInterceptOfSlope	-0.09	0.03	0.006	-0.71	ne.y	health.self.adv
AdvVarianceOfSlope	0.02	0.08	0.825	1.00	ne.y	health.self.adv
Cnsrct.Intercept_Corr_Adv_Intercept	0.00	0.01	0.886	0.01	ne.y	health.self.adv
Cnsrct.Intercept_Corr_Adv_Slope	-0.01	0.02	0.475	-0.24	ne.y	health.self.adv
Cnsrct.Slope_Corr_Adv_Intercept	-0.02	0.02	0.355	-0.10	ne.y	health.self.adv
Cnsrct.Slope_Corr_Adv_Slope	0.01	0.02	0.583	0.22	ne.y	health.self.adv
ConstructInterceptOfIntercept	3.38	0.02	0.000	8.60	ne.y	health.other.adv
ConstructVarianceOfIntercept	0.15	0.02	0.000	1.00	ne.y	health.other.adv
ConstructInterceptOfSlope	0.28	0.03	0.000	0.72	ne.y	health.other.adv
ConstructVarianceOfSlope	0.15	0.04	0.000	1.00	ne.y	health.other.adv
AdvInterceptOfIntercept	2.08	0.04	0.000	3.42	ne.y	health.other.adv
AdvVarianceOfIntercept	0.37	0.09	0.000	1.00	ne.y	health.other.adv
AdvInterceptOfSlope	-0.27	0.05	0.000	-0.56	ne.y	health.other.adv
AdvVarianceOfSlope	0.23	0.18	0.218	1.00	ne.y	health.other.adv
Cnsrct.Intercept_Corr_Adv_Intercept	-0.02	0.02	0.340	-0.08	ne.y	health.other.adv
Cnsrct.Intercept_Corr_Adv_Slope	0.03	0.03	0.204	0.18	ne.y	health.other.adv
Cnsrct.Slope_Corr_Adv_Intercept	0.00	0.02	0.857	0.02	ne.y	health.other.adv
Cnsrct.Slope_Corr_Adv_Slope	-0.05	0.03	0.139	-0.26	ne.y	health.other.adv
ConstructInterceptOfIntercept	3.38	0.02	0.000	8.60	ne.y	finance.adv
ConstructVarianceOfIntercept	0.15	0.02	0.000	1.00	ne.y	finance.adv
ConstructInterceptOfSlope	0.28	0.03	0.000	0.70	ne.y	finance.adv
ConstructVarianceOfSlope	0.16	0.04	0.000	1.00	ne.y	finance.adv
AdvInterceptOfIntercept	1.53	0.03	0.000	3.70	ne.y	finance.adv
AdvVarianceOfIntercept	0.17	0.04	0.000	1.00	ne.y	finance.adv
AdvInterceptOfSlope	-0.07	0.04	0.084	-0.17	ne.y	finance.adv
AdvVarianceOfSlope	0.17	0.10	0.082	1.00	ne.y	finance.adv
Cnsrct.Intercept_Corr_Adv_Intercept	-0.03	0.01	0.045	-0.17	ne.y	finance.adv

Cnsrct.Intercept_Corr_Adv_Slope	0.00	0.02	0.991	0.00	ne.y	finance.adv
Cnsrct.Slope_Corr_Adv_Intercept	-0.03	0.02	0.124	-0.16	ne.y	finance.adv
Cnsrct.Slope_Corr_Adv_Slope	0.00	0.02	0.880	-0.02	ne.y	finance.adv
ConstructInterceptOfIntercept	3.38	0.02	0.000	8.63	ne.y	legal.adv
ConstructVarianceOfIntercept	0.15	0.02	0.000	1.00	ne.y	legal.adv
ConstructInterceptOfSlope	0.28	0.03	0.000	0.71	ne.y	legal.adv
ConstructVarianceOfSlope	0.15	0.04	0.000	1.00	ne.y	legal.adv
AdvInterceptOfIntercept	1.54	0.03	0.000	4.14	ne.y	legal.adv
AdvVarianceOfIntercept	0.14	0.06	0.017	1.00	ne.y	legal.adv
AdvInterceptOfSlope	-0.19	0.04	0.000	-0.69	ne.y	legal.adv
AdvVarianceOfSlope	0.07	0.12	0.547	1.00	ne.y	legal.adv
Cnsrct.Intercept_Corr_Adv_Intercept	-0.04	0.02	0.009	-0.29	ne.y	legal.adv
Cnsrct.Intercept_Corr_Adv_Slope	0.05	0.02	0.020	0.48	ne.y	legal.adv
Cnsrct.Slope_Corr_Adv_Intercept	-0.01	0.02	0.746	-0.04	ne.y	legal.adv
Cnsrct.Slope_Corr_Adv_Slope	-0.04	0.03	0.187	-0.33	ne.y	legal.adv
ConstructInterceptOfIntercept	3.38	0.02	0.000	8.69	ne.y	violence.adv
ConstructVarianceOfIntercept	0.15	0.02	0.000	1.00	ne.y	violence.adv
ConstructInterceptOfSlope	0.28	0.03	0.000	0.72	ne.y	violence.adv
ConstructVarianceOfSlope	0.15	0.04	0.000	1.00	ne.y	violence.adv
AdvInterceptOfIntercept	1.43	0.03	0.000	3.65	ne.y	violence.adv
AdvVarianceOfIntercept	0.15	0.04	0.000	1.00	ne.y	violence.adv
AdvInterceptOfSlope	-0.23	0.03	0.000	-0.74	ne.y	violence.adv
AdvVarianceOfSlope	0.09	0.08	0.248	1.00	ne.y	violence.adv
Cnsrct.Intercept_Corr_Adv_Intercept	-0.02	0.01	0.159	-0.13	ne.y	violence.adv
Cnsrct.Intercept_Corr_Adv_Slope	0.03	0.02	0.116	0.23	ne.y	violence.adv
Cnsrct.Slope_Corr_Adv_Intercept	-0.01	0.02	0.478	-0.08	ne.y	violence.adv
Cnsrct.Slope_Corr_Adv_Slope	-0.03	0.02	0.160	-0.26	ne.y	violence.adv
ConstructInterceptOfIntercept	3.38	0.02	0.000	8.67	ne.y	discrimination.adv
ConstructVarianceOfIntercept	0.15	0.02	0.000	1.00	ne.y	discrimination.adv
ConstructInterceptOfSlope	0.28	0.03	0.000	0.72	ne.y	discrimination.adv
ConstructVarianceOfSlope	0.15	0.04	0.000	1.00	ne.y	discrimination.adv
AdvInterceptOfIntercept	1.22	0.02	0.000	5.12	ne.y	discrimination.adv
AdvVarianceOfIntercept	0.06	0.02	0.003	1.00	ne.y	discrimination.adv

AdvInterceptOfSlope	-0.01	0.03	0.822	-0.02	ne.y	discrimination.adv
**AdvVarianceOfSlope**	**0.13**	**0.04**	**0.002**	**1.00**	**ne.y**	**discrimination.adv**
Cnsrct.Intercept_Corr_Adv_Intercept	-0.02	0.01	0.015	-0.24	ne.y	discrimination.adv
Cnsrct.Intercept_Corr_Adv_Slope	0.02	0.01	0.150	0.13	ne.y	discrimination.adv
Cnsrct.Slope_Corr_Adv_Intercept	-0.01	0.01	0.411	-0.10	ne.y	discrimination.adv
Cnsrct.Slope_Corr_Adv_Slope	0.01	0.02	0.615	0.06	ne.y	discrimination.adv
ConstructInterceptOfIntercept	3.38	0.02	0.000	8.64	ne.y	activities.adv
ConstructVarianceOfIntercept	0.15	0.02	0.000	1.00	ne.y	activities.adv
ConstructInterceptOfSlope	0.28	0.03	0.000	0.74	ne.y	activities.adv
ConstructVarianceOfSlope	0.15	0.04	0.000	1.00	ne.y	activities.adv
AdvInterceptOfIntercept	1.35	0.02	0.000	5.89	ne.y	activities.adv
AdvVarianceOfIntercept	0.05	0.02	0.009	1.00	ne.y	activities.adv
AdvInterceptOfSlope	-0.10	0.02	0.000	-1.67	ne.y	activities.adv
AdvVarianceOfSlope	0.00	0.04	0.932	1.00	ne.y	activities.adv
Cnsrct.Intercept_Corr_Adv_Intercept	-0.01	0.01	0.269	-0.11	ne.y	activities.adv
Cnsrct.Intercept_Corr_Adv_Slope	0.01	0.01	0.607	0.27	ne.y	activities.adv
Cnsrct.Slope_Corr_Adv_Intercept	-0.02	0.01	0.125	-0.19	ne.y	activities.adv
Cnsrct.Slope_Corr_Adv_Slope	-0.02	0.01	0.216	-0.77	ne.y	activities.adv
ConstructInterceptOfIntercept	3.45	0.02	0.000	7.65	ne.p	academic.adv
ConstructVarianceOfIntercept	0.20	0.02	0.000	1.00	ne.p	academic.adv
ConstructInterceptOfSlope	0.24	0.02	0.000	0.66	ne.p	academic.adv
ConstructVarianceOfSlope	0.13	0.03	0.000	1.00	ne.p	academic.adv
AdvInterceptOfIntercept	1.79	0.03	0.000	3.37	ne.p	academic.adv
AdvVarianceOfIntercept	0.28	0.05	0.000	1.00	ne.p	academic.adv
AdvInterceptOfSlope	0.05	0.04	0.137	0.13	ne.p	academic.adv
AdvVarianceOfSlope	0.17	0.10	0.073	1.00	ne.p	academic.adv
Cnsrct.Intercept_Corr_Adv_Intercept	-0.05	0.01	0.000	-0.22	ne.p	academic.adv
Cnsrct.Intercept_Corr_Adv_Slope	0.02	0.02	0.254	0.10	ne.p	academic.adv
Cnsrct.Slope_Corr_Adv_Intercept	0.00	0.01	0.874	-0.01	ne.p	academic.adv
**Cnsrct.Slope_Corr_Adv_Slope**	**-0.04**	**0.02**	**0.008**	**-0.29**	**ne.p**	**academic.adv**
ConstructInterceptOfIntercept	3.45	0.02	0.000	7.74	ne.p	behavioral.adv
ConstructVarianceOfIntercept	0.20	0.02	0.000	1.00	ne.p	behavioral.adv
ConstructInterceptOfSlope	0.24	0.02	0.000	0.69	ne.p	behavioral.adv

ConstructVarianceOfSlope	0.12	0.03	0.000	1.00	ne.p	behavioral.adv
AdvInterceptOfIntercept	1.42	0.03	0.000	3.21	ne.p	behavioral.adv
AdvVarianceOfIntercept	0.20	0.03	0.000	1.00	ne.p	behavioral.adv
AdvInterceptOfSlope	-0.24	0.03	0.000	-1.25	ne.p	behavioral.adv
AdvVarianceOfSlope	0.04	0.07	0.595	1.00	ne.p	behavioral.adv
Cnsrct.Intercept_Corr_Adv_Intercept	-0.03	0.01	0.008	-0.17	ne.p	behavioral.adv
Cnsrct.Intercept_Corr_Adv_Slope	-0.01	0.02	0.636	-0.08	ne.p	behavioral.adv
Cnsrct.Slope_Corr_Adv_Intercept	0.01	0.01	0.297	0.08	ne.p	behavioral.adv
Cnsrct.Slope_Corr_Adv_Slope	-0.02	0.01	0.095	-0.36	ne.p	behavioral.adv
ConstructInterceptOfIntercept	3.44	0.02	0.000	7.64	ne.p	peer.adv
ConstructVarianceOfIntercept	0.20	0.02	0.000	1.00	ne.p	peer.adv
ConstructInterceptOfSlope	0.24	0.02	0.000	0.65	ne.p	peer.adv
ConstructVarianceOfSlope	0.14	0.03	0.000	1.00	ne.p	peer.adv
AdvInterceptOfIntercept	1.90	0.03	0.000	3.52	ne.p	peer.adv
AdvVarianceOfIntercept	0.29	0.05	0.000	1.00	ne.p	peer.adv
AdvInterceptOfSlope	-0.22	0.04	0.000	-0.56	ne.p	peer.adv
AdvVarianceOfSlope	0.15	0.09	0.076	1.00	ne.p	peer.adv
Cnsrct.Intercept_Corr_Adv_Intercept	-0.06	0.01	0.000	-0.26	ne.p	peer.adv
Cnsrct.Intercept_Corr_Adv_Slope	0.05	0.02	0.004	0.27	ne.p	peer.adv
Cnsrct.Slope_Corr_Adv_Intercept	0.01	0.01	0.457	0.05	ne.p	peer.adv
**Cnsrct.Slope_Corr_Adv_Slope**	**-0.04**	**0.02**	**0.026**	**-0.25**	**ne.p**	**peer.adv**
ConstructInterceptOfIntercept	3.44	0.02	0.000	7.62	ne.p	parentchild.relationship.adv
ConstructVarianceOfIntercept	0.20	0.02	0.000	1.00	ne.p	parentchild.relationship.adv
ConstructInterceptOfSlope	0.24	0.02	0.000	0.64	ne.p	parentchild.relationship.adv
ConstructVarianceOfSlope	0.14	0.03	0.000	1.00	ne.p	parentchild.relationship.adv
AdvInterceptOfIntercept	1.95	0.03	0.000	4.13	ne.p	parentchild.relationship.adv
AdvVarianceOfIntercept	0.22	0.04	0.000	1.00	ne.p	parentchild.relationship.adv
AdvInterceptOfSlope	-0.05	0.04	0.173	-0.11	ne.p	parentchild.relationship.adv
**AdvVarianceOfSlope**	**0.20**	**0.09**	**0.028**	**1.00**	**ne.p**	**parentchild.relationship.adv**
Cnsrct.Intercept_Corr_Adv_Intercept	-0.05	0.01	0.000	-0.25	ne.p	parentchild.relationship.adv
Cnsrct.Intercept_Corr_Adv_Slope	-0.01	0.02	0.709	-0.03	ne.p	parentchild.relationship.adv
Cnsrct.Slope_Corr_Adv_Intercept	-0.02	0.01	0.219	-0.09	ne.p	parentchild.relationship.adv
Cnsrct.Slope_Corr_Adv_Slope	0.00	0.02	0.838	-0.02	ne.p	parentchild.relationship.adv

ConstructInterceptOfIntercept	3.44	0.02	0.000	7.59	ne.p	parentparent.relationship.adv
ConstructVarianceOfIntercept	0.21	0.02	0.000	1.00	ne.p	parentparent.relationship.adv
ConstructInterceptOfSlope	0.24	0.02	0.000	0.64	ne.p	parentparent.relationship.adv
ConstructVarianceOfSlope	0.14	0.03	0.000	1.00	ne.p	parentparent.relationship.adv
AdvInterceptOfIntercept	1.85	0.03	0.000	3.06	ne.p	parentparent.relationship.adv
AdvVarianceOfIntercept	0.36	0.06	0.000	1.00	ne.p	parentparent.relationship.adv
AdvInterceptOfSlope	-0.11	0.04	0.009	-0.16	ne.p	parentparent.relationship.adv
**AdvVarianceOfSlope**	**0.43**	**0.11**	**0.000**	**1.00**	**ne.p**	**parentparent.relationship.adv**
Cnsrct.Intercept_Corr_Adv_Intercept	-0.02	0.02	0.245	-0.06	ne.p	parentparent.relationship.adv
Cnsrct.Intercept_Corr_Adv_Slope	0.00	0.02	0.940	-0.01	ne.p	parentparent.relationship.adv
Cnsrct.Slope_Corr_Adv_Intercept	-0.02	0.01	0.194	-0.08	ne.p	parentparent.relationship.adv
Cnsrct.Slope_Corr_Adv_Slope	0.01	0.02	0.682	0.03	ne.p	parentparent.relationship.adv
ConstructInterceptOfIntercept	3.45	0.02	0.000	7.67	ne.p	household.relationship.adv
ConstructVarianceOfIntercept	0.20	0.02	0.000	1.00	ne.p	household.relationship.adv
ConstructInterceptOfSlope	0.24	0.02	0.000	0.67	ne.p	household.relationship.adv
ConstructVarianceOfSlope	0.13	0.03	0.000	1.00	ne.p	household.relationship.adv
AdvInterceptOfIntercept	2.06	0.03	0.000	4.45	ne.p	household.relationship.adv
AdvVarianceOfIntercept	0.21	0.04	0.000	1.00	ne.p	household.relationship.adv
AdvInterceptOfSlope	-0.13	0.04	0.000	-0.47	ne.p	household.relationship.adv
AdvVarianceOfSlope	0.08	0.09	0.372	1.00	ne.p	household.relationship.adv
Cnsrct.Intercept_Corr_Adv_Intercept	-0.03	0.01	0.037	-0.14	ne.p	household.relationship.adv
Cnsrct.Intercept_Corr_Adv_Slope	-0.04	0.02	0.044	-0.28	ne.p	household.relationship.adv
Cnsrct.Slope_Corr_Adv_Intercept	-0.01	0.01	0.260	-0.09	ne.p	household.relationship.adv
Cnsrct.Slope_Corr_Adv_Slope	-0.02	0.02	0.246	-0.19	ne.p	household.relationship.adv
ConstructInterceptOfIntercept	3.44	0.02	0.000	7.58	ne.p	body.adv
ConstructVarianceOfIntercept	0.21	0.02	0.000	1.00	ne.p	body.adv
ConstructInterceptOfSlope	0.24	0.02	0.000	0.64	ne.p	body.adv
ConstructVarianceOfSlope	0.14	0.03	0.000	1.00	ne.p	body.adv
AdvInterceptOfIntercept	1.59	0.03	0.000	3.66	ne.p	body.adv
AdvVarianceOfIntercept	0.19	0.04	0.000	1.00	ne.p	body.adv
AdvInterceptOfSlope	0.00	0.03	0.964	NA	ne.p	body.adv
AdvVarianceOfSlope	-0.02	0.09	0.795	NA	ne.p	body.adv
Cnsrct.Intercept_Corr_Adv_Intercept	-0.01	0.01	0.693	-0.03	ne.p	body.adv

Cnsrct.Intercept_Corr_Adv_Slope	0.00	0.02	0.945	0.02	ne.p	body.adv
Cnsrct.Slope_Corr_Adv_Intercept	0.00	0.01	0.885	0.01	ne.p	body.adv
**Cnsrct.Slope_Corr_Adv_Slope**	**-0.03**	**0.02**	**0.049**	**-0.54**	**ne.p**	**body.adv**
ConstructInterceptOfIntercept	3.44	0.02	0.000	7.58	ne.p	romantic.adv
ConstructVarianceOfIntercept	0.21	0.02	0.000	1.00	ne.p	romantic.adv
ConstructInterceptOfSlope	0.24	0.02	0.000	0.64	ne.p	romantic.adv
ConstructVarianceOfSlope	0.14	0.03	0.000	1.00	ne.p	romantic.adv
AdvInterceptOfIntercept	1.38	0.02	0.000	3.39	ne.p	romantic.adv
AdvVarianceOfIntercept	0.16	0.03	0.000	1.00	ne.p	romantic.adv
AdvInterceptOfSlope	0.03	0.03	0.283	0.12	ne.p	romantic.adv
AdvVarianceOfSlope	0.06	0.06	0.318	1.00	ne.p	romantic.adv
Cnsrct.Intercept_Corr_Adv_Intercept	0.01	0.01	0.502	0.04	ne.p	romantic.adv
Cnsrct.Intercept_Corr_Adv_Slope	-0.01	0.01	0.441	-0.09	ne.p	romantic.adv
Cnsrct.Slope_Corr_Adv_Intercept	-0.01	0.01	0.361	-0.07	ne.p	romantic.adv
Cnsrct.Slope_Corr_Adv_Slope	0.01	0.01	0.666	0.06	ne.p	romantic.adv
ConstructInterceptOfIntercept	3.44	0.02	0.000	7.57	ne.p	neighborhood.adv
ConstructVarianceOfIntercept	0.21	0.02	0.000	1.00	ne.p	neighborhood.adv
ConstructInterceptOfSlope	0.24	0.02	0.000	0.63	ne.p	neighborhood.adv
ConstructVarianceOfSlope	0.14	0.03	0.000	1.00	ne.p	neighborhood.adv
AdvInterceptOfIntercept	1.52	0.03	0.000	3.51	ne.p	neighborhood.adv
AdvVarianceOfIntercept	0.19	0.04	0.000	1.00	ne.p	neighborhood.adv
AdvInterceptOfSlope	0.02	0.03	0.537	0.08	ne.p	neighborhood.adv
AdvVarianceOfSlope	0.08	0.08	0.322	1.00	ne.p	neighborhood.adv
Cnsrct.Intercept_Corr_Adv_Intercept	0.01	0.01	0.266	0.07	ne.p	neighborhood.adv
Cnsrct.Intercept_Corr_Adv_Slope	-0.01	0.02	0.679	-0.05	ne.p	neighborhood.adv
Cnsrct.Slope_Corr_Adv_Intercept	-0.01	0.01	0.482	-0.05	ne.p	neighborhood.adv
Cnsrct.Slope_Corr_Adv_Slope	-0.01	0.02	0.364	-0.13	ne.p	neighborhood.adv
ConstructInterceptOfIntercept	3.44	0.02	0.000	7.54	ne.p	health.self.adv
ConstructVarianceOfIntercept	0.21	0.02	0.000	1.00	ne.p	health.self.adv
ConstructInterceptOfSlope	0.24	0.02	0.000	0.63	ne.p	health.self.adv
ConstructVarianceOfSlope	0.15	0.03	0.000	1.00	ne.p	health.self.adv
AdvInterceptOfIntercept	1.49	0.03	0.000	3.77	ne.p	health.self.adv
AdvVarianceOfIntercept	0.16	0.04	0.000	1.00	ne.p	health.self.adv

AdvInterceptOfSlope	-0.10	0.03	0.005	-0.57	ne.p	health.self.adv
AdvVarianceOfSlope	0.03	0.08	0.717	1.00	ne.p	health.self.adv
Cnsrct.Intercept_Corr_Adv_Intercept	0.02	0.01	0.080	0.12	ne.p	health.self.adv
Cnsrct.Intercept_Corr_Adv_Slope	-0.01	0.02	0.710	-0.08	ne.p	health.self.adv
Cnsrct.Slope_Corr_Adv_Intercept	-0.01	0.01	0.525	-0.05	ne.p	health.self.adv
Cnsrct.Slope_Corr_Adv_Slope	0.00	0.02	0.786	0.06	ne.p	health.self.adv
ConstructInterceptOfIntercept	3.44	0.02	0.000	7.55	ne.p	health.other.adv
ConstructVarianceOfIntercept	0.21	0.02	0.000	1.00	ne.p	health.other.adv
ConstructInterceptOfSlope	0.24	0.02	0.000	0.63	ne.p	health.other.adv
ConstructVarianceOfSlope	0.14	0.03	0.000	1.00	ne.p	health.other.adv
AdvInterceptOfIntercept	2.08	0.04	0.000	3.46	ne.p	health.other.adv
AdvVarianceOfIntercept	0.36	0.09	0.000	1.00	ne.p	health.other.adv
AdvInterceptOfSlope	-0.27	0.05	0.000	-0.58	ne.p	health.other.adv
AdvVarianceOfSlope	0.21	0.18	0.263	1.00	ne.p	health.other.adv
Cnsrct.Intercept_Corr_Adv_Intercept	0.02	0.02	0.258	0.08	ne.p	health.other.adv
Cnsrct.Intercept_Corr_Adv_Slope	0.01	0.02	0.746	0.04	ne.p	health.other.adv
Cnsrct.Slope_Corr_Adv_Intercept	-0.03	0.02	0.059	-0.15	ne.p	health.other.adv
Cnsrct.Slope_Corr_Adv_Slope	0.00	0.02	0.979	0.00	ne.p	health.other.adv
ConstructInterceptOfIntercept	3.44	0.02	0.000	7.61	ne.p	finance.adv
ConstructVarianceOfIntercept	0.20	0.02	0.000	1.00	ne.p	finance.adv
ConstructInterceptOfSlope	0.24	0.02	0.000	0.64	ne.p	finance.adv
ConstructVarianceOfSlope	0.14	0.03	0.000	1.00	ne.p	finance.adv
AdvInterceptOfIntercept	1.53	0.03	0.000	3.70	ne.p	finance.adv
AdvVarianceOfIntercept	0.17	0.04	0.000	1.00	ne.p	finance.adv
AdvInterceptOfSlope	-0.07	0.04	0.080	-0.16	ne.p	finance.adv
AdvVarianceOfSlope	0.18	0.10	0.067	1.00	ne.p	finance.adv
Cnsrct.Intercept_Corr_Adv_Intercept	-0.03	0.01	0.047	-0.14	ne.p	finance.adv
Cnsrct.Intercept_Corr_Adv_Slope	0.02	0.02	0.320	0.10	ne.p	finance.adv
Cnsrct.Slope_Corr_Adv_Intercept	-0.01	0.01	0.495	-0.06	ne.p	finance.adv
Cnsrct.Slope_Corr_Adv_Slope	-0.03	0.02	0.146	-0.17	ne.p	finance.adv
ConstructInterceptOfIntercept	3.44	0.02	0.000	7.57	ne.p	legal.adv
ConstructVarianceOfIntercept	0.21	0.02	0.000	1.00	ne.p	legal.adv
ConstructInterceptOfSlope	0.24	0.02	0.000	0.63	ne.p	legal.adv

ConstructVarianceOfSlope	0.14	0.03	0.000	1.00	ne.p	legal.adv
AdvInterceptOfIntercept	1.54	0.03	0.000	4.08	ne.p	legal.adv
AdvVarianceOfIntercept	0.14	0.06	0.014	1.00	ne.p	legal.adv
AdvInterceptOfSlope	-0.19	0.04	0.000	-0.65	ne.p	legal.adv
AdvVarianceOfSlope	0.08	0.12	0.499	1.00	ne.p	legal.adv
Cnsrct.Intercept_Corr_Adv_Intercept	-0.02	0.02	0.108	-0.14	ne.p	legal.adv
Cnsrct.Intercept_Corr_Adv_Slope	0.01	0.02	0.560	0.09	ne.p	legal.adv
Cnsrct.Slope_Corr_Adv_Intercept	0.00	0.02	0.868	-0.02	ne.p	legal.adv
Cnsrct.Slope_Corr_Adv_Slope	-0.01	0.02	0.709	-0.07	ne.p	legal.adv
ConstructInterceptOfIntercept	3.44	0.02	0.000	7.60	ne.p	violence.adv
ConstructVarianceOfIntercept	0.21	0.02	0.000	1.00	ne.p	violence.adv
ConstructInterceptOfSlope	0.24	0.02	0.000	0.65	ne.p	violence.adv
ConstructVarianceOfSlope	0.14	0.03	0.000	1.00	ne.p	violence.adv
AdvInterceptOfIntercept	1.43	0.03	0.000	3.74	ne.p	violence.adv
AdvVarianceOfIntercept	0.15	0.04	0.000	1.00	ne.p	violence.adv
AdvInterceptOfSlope	-0.23	0.03	0.000	-0.75	ne.p	violence.adv
AdvVarianceOfSlope	0.09	0.08	0.271	1.00	ne.p	violence.adv
Cnsrct.Intercept_Corr_Adv_Intercept	-0.05	0.01	0.001	-0.26	ne.p	violence.adv
Cnsrct.Intercept_Corr_Adv_Slope	0.03	0.02	0.053	0.23	ne.p	violence.adv
Cnsrct.Slope_Corr_Adv_Intercept	0.01	0.01	0.335	0.09	ne.p	violence.adv
**Cnsrct.Slope_Corr_Adv_Slope**	**-0.03**	**0.02**	**0.029**	**-0.31**	**ne.p**	**violence.adv**
ConstructInterceptOfIntercept	3.44	0.02	0.000	7.59	ne.p	discrimination.adv
ConstructVarianceOfIntercept	0.21	0.02	0.000	1.00	ne.p	discrimination.adv
ConstructInterceptOfSlope	0.24	0.02	0.000	0.64	ne.p	discrimination.adv
ConstructVarianceOfSlope	0.14	0.03	0.000	1.00	ne.p	discrimination.adv
AdvInterceptOfIntercept	1.22	0.02	0.000	5.01	ne.p	discrimination.adv
AdvVarianceOfIntercept	0.06	0.02	0.002	1.00	ne.p	discrimination.adv
AdvInterceptOfSlope	0.00	0.03	0.889	-0.01	ne.p	discrimination.adv
**AdvVarianceOfSlope**	**0.13**	**0.04**	**0.001**	**1.00**	**ne.p**	**discrimination.adv**
Cnsrct.Intercept_Corr_Adv_Intercept	0.01	0.01	0.304	0.08	ne.p	discrimination.adv
Cnsrct.Intercept_Corr_Adv_Slope	-0.02	0.01	0.126	-0.11	ne.p	discrimination.adv
Cnsrct.Slope_Corr_Adv_Intercept	0.01	0.01	0.336	0.09	ne.p	discrimination.adv
Cnsrct.Slope_Corr_Adv_Slope	-0.02	0.01	0.156	-0.12	ne.p	discrimination.adv

ConstructInterceptOfIntercept	3.44	0.02	0.000	7.58	ne.p	activities.adv
ConstructVarianceOfIntercept	0.21	0.02	0.000	1.00	ne.p	activities.adv
ConstructInterceptOfSlope	0.24	0.02	0.000	0.64	ne.p	activities.adv
ConstructVarianceOfSlope	0.14	0.03	0.000	1.00	ne.p	activities.adv
AdvInterceptOfIntercept	1.35	0.02	0.000	5.86	ne.p	activities.adv
AdvVarianceOfIntercept	0.05	0.02	0.009	1.00	ne.p	activities.adv
AdvInterceptOfSlope	-0.10	0.02	0.000	-1.00	ne.p	activities.adv
AdvVarianceOfSlope	0.01	0.04	0.817	1.00	ne.p	activities.adv
Cnsrct.Intercept_Corr_Adv_Intercept	0.00	0.01	0.861	0.01	ne.p	activities.adv
Cnsrct.Intercept_Corr_Adv_Slope	-0.01	0.01	0.407	-0.21	ne.p	activities.adv
Cnsrct.Slope_Corr_Adv_Intercept	0.00	0.01	0.590	-0.05	ne.p	activities.adv
Cnsrct.Slope_Corr_Adv_Slope	-0.01	0.01	0.384	-0.25	ne.p	activities.adv

Table S19a. uv.onPCR.adv.fits.indices.csv

chisq	df	rmsea	tli	cfi	ModID
34.606	5	0.093	0.897	0.957	ec.y_uv.onPCR.adv
30.664	5	0.087	0.946	0.977	ec.p_uv.onPCR.adv
12.817	5	0.048	0.973	0.989	ac.y_uv.onPCR.adv
21.978	5	0.071	0.958	0.982	ac.p_uv.onPCR.adv
22.824	5	0.072	0.89	0.954	at.y_uv.onPCR.adv
13.037	5	0.049	0.975	0.989	at.p_uv.onPCR.adv
24.468	5	0.076	0.865	0.944	ic.y_uv.onPCR.adv
19.619	5	0.065	0.943	0.976	ic.p_uv.onPCR.adv
93.248	5	0.161	0.471	0.779	ne.y_uv.onPCR.adv
43.814	5	0.107	0.903	0.96	ne.p_uv.onPCR.adv
26.657	5	0.08	0.901	0.959	ag.y_uv.onPCR.adv
22.441	5	0.072	0.95	0.979	ag.p_uv.onPCR.adv
78.811	5	0.147	0.478	0.783	fear.y_uv.onPCR.adv
44.953	5	0.108	0.865	0.944	fear.p_uv.onPCR.adv
44.585	5	0.108	0.704	0.877	fr.y_uv.onPCR.adv
20.113	5	0.067	0.947	0.978	fr.p_uv.onPCR.adv
41.638	5	0.104	0.798	0.916	shy.y_uv.onPCR.adv
4.137	5	0	1.002	1	shy.p_uv.onPCR.adv

Table S19b. uv.onPPR.adv.fits.indices.csv

chisq	df	rmsea	tli	cfi	ModID
37.183	5	0.097	0.881	0.95	ec.y_uv.onPPR.adv
30.328	5	0.086	0.945	0.977	ec.p_uv.onPPR.adv
12.016	5	0.045	0.975	0.99	ac.y_uv.onPPR.adv
23.179	5	0.073	0.954	0.981	ac.p_uv.onPPR.adv
22.799	5	0.072	0.879	0.95	at.y_uv.onPPR.adv
7.616	5	0.028	0.992	0.996	at.p_uv.onPPR.adv
32.986	5	0.091	0.793	0.914	ic.y_uv.onPPR.adv
25.019	5	0.077	0.92	0.966	ic.p_uv.onPPR.adv
88.322	5	0.156	0.436	0.765	ne.y_uv.onPPR.adv
42.42	5	0.105	0.902	0.959	ne.p_uv.onPPR.adv
20.122	5	0.067	0.924	0.968	ag.y_uv.onPPR.adv
22.187	5	0.071	0.948	0.978	ag.p_uv.onPPR.adv
77.77	5	0.146	0.479	0.783	fear.y_uv.onPPR.adv
42.975	5	0.106	0.869	0.945	fear.p_uv.onPPR.adv
44.211	5	0.107	0.678	0.866	fr.y_uv.onPPR.adv
20.72	5	0.068	0.941	0.975	fr.p_uv.onPPR.adv
39.174	5	0.1	0.808	0.92	shy.y_uv.onPPR.adv
2.36	5	0	1.007	1	shy.p_uv.onPPR.adv

Table S19c. uv.onDisc.adv.fits.indices.csv

chisq	df	rmsea	tli	cfi	ModID
38.465	5	0.099	0.87	0.946	ec.y_uv.onDisc.adv
25.929	5	0.078	0.954	0.981	ec.p_uv.onDisc.adv
13.71	5	0.051	0.968	0.987	ac.y_uv.onDisc.adv
20.417	5	0.067	0.96	0.983	ac.p_uv.onDisc.adv
28.892	5	0.084	0.833	0.93	at.y_uv.onDisc.adv
8.159	5	0.03	0.99	0.996	at.p_uv.onDisc.adv
30.003	5	0.086	0.8	0.917	ic.y_uv.onDisc.adv
19.01	5	0.064	0.942	0.976	ic.p_uv.onDisc.adv
88.555	5	0.157	0.428	0.762	ne.y_uv.onDisc.adv
43.751	5	0.107	0.899	0.958	ne.p_uv.onDisc.adv
21.743	5	0.07	0.914	0.964	ag.y_uv.onDisc.adv
21.065	5	0.069	0.951	0.979	ag.p_uv.onDisc.adv
81.231	5	0.15	0.465	0.777	fear.y_uv.onDisc.adv
43.125	5	0.106	0.87	0.946	fear.p_uv.onDisc.adv
46.682	5	0.111	0.66	0.858	fr.y_uv.onDisc.adv
21.91	5	0.07	0.937	0.974	fr.p_uv.onDisc.adv
44.088	5	0.107	0.782	0.909	shy.y_uv.onDisc.adv
17.126	5	0.06	0.969	0.987	shy.p_uv.onDisc.adv

Table S20a. Youth and Parent Report Estimates for Prevalence of Growth Despite Parent-Child Conflict (Estimates for Univariate Model Conditioned on Initial Adversity, Change in Adversity, and Cohort)

	Low Adversity		% Change >/= SESOI despite baseline Adversity		% Change >/= SESOI despite increases in Adversity	
Model	Decrease	Increase	Decrease	Increase	Decrease	Increase
ec.y_bv	16% (63/404)	50% (203/404)	22% (34/155)	41% (63/155)	38% (43/114)	22% (25/114)
ec.p_bv	20% (82/404)	49% (196/404)	17% (26/155)	49% (76/155)	23% (26/114)	47% (54/114)
ac.y_bv	41% (167/404)	17% (67/404)	46% (72/155)	14% (21/155)	68% (77/114)	7% (8/114)
ac.p_bv	27% (111/404)	28% (115/404)	17% (26/155)	32% (49/155)	32% (36/114)	23% (26/114)
at.y_bv	17% (70/404)	54% (218/404)	22% (34/155)	45% (69/155)	34% (39/114)	37% (42/114)
at.p_bv	6% (24/404)	48% (195/404)	6% (9/155)	53% (82/155)	11% (12/114)	50% (57/114)
ic.y_bv	0% (0/404)	97% (393/404)	0% (0/155)	85% (131/155)	0% (0/114)	71% (81/114)
ic.p_bv	7% (27/404)	71% (286/404)	11% (17/155)	60% (93/155)	11% (13/114)	59% (67/114)
ne.y_bv	3% (11/404)	83% (336/404)	5% (7/155)	75% (117/155)	12% (14/114)	59% (67/114)
ne.p_bv	11% (44/404)	72% (291/404)	13% (20/155)	64% (99/155)	14% (16/114)	63% (72/114)
ag.y_bv	14% (56/404)	45% (182/404)	28% (44/155)	34% (52/155)	51% (58/114)	21% (24/114)
ag.p_bv	13% (53/404)	62% (252/404)	16% (25/155)	52% (81/155)	20% (23/114)	50% (57/114)
fear.y_bv	0% (0/404)	100% (402/404)	0% (0/155)	100% (155/155)	0% (0/114)	99% (113/114)
fear.p_bv	0% (1/404)	96% (387/404)	0% (0/155)	95% (147/155)	1% (1/114)	92% (105/114)
fr.y_bv	5% (20/404)	69% (279/404)	10% (16/155)	58% (90/155)	25% (29/114)	32% (37/114)
fr.p_bv	6% (25/404)	69% (278/404)	7% (11/155)	61% (95/155)	12% (14/114)	49% (56/114)
shy.y_bv	5% (19/404)	41% (167/404)	14% (21/155)	28% (44/155)	16% (18/114)	28% (32/114)
shy.p_bv	14% (57/404)	33% (135/404)	18% (28/155)	19% (29/155)	9% (10/114)	35% (40/114)

Table S20b. Youth and Parent Report Estimates for Prevalence of Growth Despite Parent-Parent Conflict (Estimates for Univariate Model Conditioned on Initial Adversity, Change in Adversity, and Cohort)

	Low Adversity		% Change >/= SESOI despite baseline Adversity		% Change >/= SESOI despite increases in Adversity	
Model	Decrease	Increase	Decrease	Increase	Decrease	Increase
ec.y_bv	15% (55/379)	49% (185/379)	21% (24/117)	40% (47/117)	29% (34/118)	35% (41/118)
ec.p_bv	20% (76/379)	51% (195/379)	26% (30/117)	32% (38/117)	31% (36/118)	40% (47/118)
ac.y_bv	45% (169/379)	16% (60/379)	41% (48/117)	17% (20/117)	59% (70/118)	8% (10/118)
ac.p_bv	23% (88/379)	32% (122/379)	36% (42/117)	21% (24/117)	42% (49/118)	19% (23/118)
at.y_bv	18% (70/379)	55% (208/379)	20% (23/117)	40% (47/117)	29% (34/118)	44% (52/118)
at.p_bv	8% (29/379)	50% (188/379)	9% (11/117)	41% (48/117)	13% (15/118)	51% (60/118)
ic.y_bv	0% (0/379)	98% (372/379)	0% (0/117)	97% (113/117)	0% (0/118)	92% (109/118)
ic.p_bv	6% (23/379)	73% (275/379)	14% (16/117)	60% (70/117)	11% (13/118)	58% (69/118)
ne.y_bv	2% (6/379)	82% (309/379)	4% (5/117)	83% (97/117)	9% (11/118)	68% (80/118)
ne.p_bv	12% (44/379)	72% (272/379)	12% (14/117)	65% (76/117)	17% (20/118)	65% (77/118)
ag.y_bv	15% (58/379)	40% (152/379)	22% (26/117)	41% (48/117)	35% (41/118)	19% (23/118)
ag.p_bv	14% (53/379)	62% (234/379)	12% (14/117)	59% (69/117)	19% (23/118)	55% (65/118)
fear.y_bv	0% (0/379)	99% (377/379)	0% (0/117)	100% (117/117)	0% (0/118)	98% (116/118)
fear.p_bv	0% (1/379)	95% (361/379)	0% (0/117)	90% (105/117)	1% (1/118)	94% (111/118)
fr.y_bv	5% (20/379)	67% (254/379)	8% (9/117)	59% (69/117)	10% (12/118)	53% (63/118)
fr.p_bv	7% (25/379)	68% (259/379)	5% (6/117)	65% (76/117)	13% (15/118)	59% (70/118)
shy.y_bv	7% (25/379)	37% (140/379)	7% (8/117)	47% (55/117)	13% (15/118)	35% (41/118)
shy.p_bv	15% (58/379)	33% (125/379)	17% (20/117)	22% (26/117)	14% (16/118)	34% (40/118)

Table S20c. Youth and Parent Report Estimates for Prevalence of Growth Despite Discrimination/Acculturation Stress(Estimates for Univariate Model Conditioned on Initial Adversity, Change in Adversity, and Cohort)

	Low Adversity		% Change >/= SESOI despite baseline Adversity		% Change >/= SESOI despite increases in Adversity	
Model	Decrease	Increase	Decrease	Increase	Decrease	Increase
ec.y_bv	18% (92/517)	47% (243/517)	17% (5/30)	37% (11/30)	28% (31/109)	37% (40/109)
ec.p_bv	20% (104/517)	50% (257/517)	20% (6/30)	47% (14/30)	28% (30/109)	42% (46/109)
ac.y_bv	44% (229/517)	16% (84/517)	40% (12/30)	20% (6/30)	60% (65/109)	15% (16/109)
ac.p_bv	25% (130/517)	30% (154/517)	27% (8/30)	27% (8/30)	37% (40/109)	24% (26/109)
at.y_bv	19% (99/517)	53% (272/517)	23% (7/30)	40% (12/30)	28% (31/109)	44% (48/109)
at.p_bv	8% (42/517)	52% (270/517)	13% (4/30)	23% (7/30)	13% (14/109)	43% (47/109)
ic.y_bv	0% (0/517)	95% (492/517)	0% (0/30)	93% (28/30)	0% (0/109)	98% (107/109)
ic.p_bv	8% (42/517)	71% (365/517)	7% (2/30)	70% (21/30)	14% (15/109)	58% (63/109)
ne.y_bv	4% (21/517)	77% (398/517)	3% (1/30)	80% (24/30)	2% (2/109)	82% (89/109)
ne.p_bv	13% (66/517)	70% (364/517)	10% (3/30)	70% (21/30)	13% (14/109)	65% (71/109)
ag.y_bv	20% (102/517)	37% (191/517)	20% (6/30)	33% (10/30)	19% (21/109)	42% (46/109)
ag.p_bv	14% (70/517)	60% (309/517)	10% (3/30)	63% (19/30)	17% (19/109)	57% (62/109)
fear.y_bv	0% (0/517)	99% (510/517)	0% (0/30)	97% (29/30)	0% (0/109)	100% (109/109)
fear.p_bv	0% (2/517)	95% (492/517)	0% (0/30)	100% (30/30)	0% (0/109)	94% (103/109)
fr.y_bv	9% (44/517)	64% (330/517)	13% (4/30)	43% (13/30)	6% (6/109)	66% (72/109)
fr.p_bv	7% (35/517)	68% (352/517)	13% (4/30)	67% (20/30)	10% (11/109)	53% (58/109)
shy.y_bv	7% (35/517)	38% (196/517)	7% (2/30)	40% (12/30)	17% (18/109)	33% (36/109)
shy.p_bv	15% (77/517)	32% (166/517)	13% (4/30)	20% (6/30)	19% (21/109)	26% (28/109)

Table S21. Fit indices for domain specific trivariate regression models

chisq	df	rmsea	tli	cfi	ModID
150.618	30	0.077	0.894	0.929	tri_PCR.adv_ec.p_anx.y
164.676	30	0.081	0.868	0.912	tri_PCR.adv_ne.p_anx.y
135.903	30	0.072	0.918	0.945	tri_PCR.adv_ec.p_avoid.y
157.795	30	0.079	0.891	0.927	tri_PCR.adv_ne.p_avoid.y
296.753	22	0.135	0.725	0.832	tri_PCR.adv_ec.p_PSS.y
313.256	22	0.139	0.677	0.803	tri_PCR.adv_ne.p_PSS.y
312.097	22	0.139	0.679	0.804	tri_PCR.adv_ec.p_ProSoc.y
332.52	22	0.144	0.612	0.763	tri_PCR.adv_ne.p_ProSoc.y
268.382	22	0.128	0.749	0.847	tri_PCR.adv_ec.p_SEQ.y
296.704	22	0.135	0.688	0.809	tri_PCR.adv_ne.p_SEQ.y
106.023	30	0.061	0.928	0.952	tri_PPR.adv_ec.p_anx.y
108.097	30	0.062	0.915	0.944	tri_PPR.adv_ne.p_anx.y
111.135	30	0.063	0.933	0.955	tri_PPR.adv_ec.p_avoid.y
116.898	30	0.065	0.92	0.946	tri_PPR.adv_ne.p_avoid.y
229.885	22	0.118	0.77	0.859	tri_PPR.adv_ec.p_PSS.y
239.033	22	0.12	0.728	0.834	tri_PPR.adv_ne.p_PSS.y
323.007	22	0.142	0.647	0.784	tri_PPR.adv_ec.p_ProSoc.y
325.878	22	0.142	0.586	0.747	tri_PPR.adv_ne.p_ProSoc.y
241.008	22	0.121	0.76	0.853	tri_PPR.adv_ec.p_SEQ.y
255.232	22	0.125	0.708	0.821	tri_PPR.adv_ne.p_SEQ.y
61.977	30	0.04	0.968	0.979	tri_Disc.adv_ec.p_anx.y
75.054	30	0.047	0.949	0.966	tri_Disc.adv_ne.p_anx.y
64.753	30	0.041	0.97	0.98	tri_Disc.adv_ec.p_avoid.y
80.094	30	0.049	0.951	0.968	tri_Disc.adv_ne.p_avoid.y
194.334	22	0.107	0.798	0.876	tri_Disc.adv_ec.p_PSS.y
211.856	22	0.112	0.749	0.847	tri_Disc.adv_ne.p_PSS.y
316.613	22	0.14	0.639	0.78	tri_Disc.adv_ec.p_ProSoc.y
328.31	22	0.143	0.569	0.737	tri_Disc.adv_ne.p_ProSoc.y
209.54	22	0.112	0.782	0.867	tri_Disc.adv_ec.p_SEQ.y
232.309	22	0.118	0.723	0.831	tri_Disc.adv_ne.p_SEQ.y

Table S22. Parameters from adversity domain trivariate regression models

Parameter	Estimate	SE	PValue	StdAll	ModelName
S_Temperment_ON_I_Factor	0.14	0.07	0.048	0.26	tri_PCR.adv_ec.p_anx.y
S_Temperment_ON_S_Factor	-0.16	0.07	0.027	-0.35	tri_PCR.adv_ec.p_anx.y
S_Temperment_ON_Cohort	0.00	0.03	0.959	0.00	tri_PCR.adv_ec.p_anx.y
I_Temperment_ON_I_Factor	-0.29	0.07	0.000	-0.31	tri_PCR.adv_ec.p_anx.y
I_Temperment_ON_Cohort	0.10	0.03	0.001	0.16	tri_PCR.adv_ec.p_anx.y
S_Temperment_ON_I_Factor	0.01	0.07	0.834	0.03	tri_PCR.adv_ne.p_anx.y
S_Temperment_ON_S_Factor	-0.01	0.06	0.913	-0.02	tri_PCR.adv_ne.p_anx.y
S_Temperment_ON_Cohort	0.02	0.03	0.349	0.08	tri_PCR.adv_ne.p_anx.y
I_Temperment_ON_I_Factor	-0.28	0.06	0.000	-0.37	tri_PCR.adv_ne.p_anx.y
I_Temperment_ON_Cohort	0.10	0.03	0.000	0.20	tri_PCR.adv_ne.p_anx.y
S_Temperment_ON_I_Factor	0.04	0.03	0.202	0.12	tri_PCR.adv_ec.p_avoid.y
S_Temperment_ON_S_Factor	-0.07	0.03	0.046	-0.22	tri_PCR.adv_ec.p_avoid.y
S_Temperment_ON_Cohort	-0.04	0.03	0.085	-0.13	tri_PCR.adv_ec.p_avoid.y
I_Temperment_ON_I_Factor	-0.07	0.03	0.019	-0.13	tri_PCR.adv_ec.p_avoid.y
I_Temperment_ON_Cohort	0.14	0.03	0.000	0.23	tri_PCR.adv_ec.p_avoid.y
S_Temperment_ON_I_Factor	0.02	0.03	0.426	0.08	tri_PCR.adv_ne.p_avoid.y
S_Temperment_ON_S_Factor	-0.07	0.03	0.034	-0.24	tri_PCR.adv_ne.p_avoid.y
S_Temperment_ON_Cohort	0.02	0.02	0.503	0.05	tri_PCR.adv_ne.p_avoid.y
I_Temperment_ON_I_Factor	-0.04	0.03	0.133	-0.09	tri_PCR.adv_ne.p_avoid.y
I_Temperment_ON_Cohort	0.14	0.02	0.000	0.26	tri_PCR.adv_ne.p_avoid.y
S_Temperment_ON_I_Factor	-0.02	0.15	0.893	-0.01	tri_PCR.adv_ec.p_PSS.y
S_Temperment_ON_S_Factor	0.08	0.13	0.536	0.05	tri_PCR.adv_ec.p_PSS.y
S_Temperment_ON_Cohort	-0.04	0.03	0.147	-0.10	tri_PCR.adv_ec.p_PSS.y
I_Temperment_ON_I_Factor	0.33	0.15	0.029	0.10	tri_PCR.adv_ec.p_PSS.y
I_Temperment_ON_Cohort	0.13	0.03	0.000	0.21	tri_PCR.adv_ec.p_PSS.y
S_Temperment_ON_I_Factor	-0.34	0.14	0.014	-0.19	tri_PCR.adv_ne.p_PSS.y
S_Temperment_ON_S_Factor	0.09	0.13	0.481	0.05	tri_PCR.adv_ne.p_PSS.y
S_Temperment_ON_Cohort	0.02	0.02	0.376	0.06	tri_PCR.adv_ne.p_PSS.y
I_Temperment_ON_I_Factor	0.49	0.13	0.000	0.18	tri_PCR.adv_ne.p_PSS.y
I_Temperment_ON_Cohort	0.13	0.02	0.000	0.25	tri_PCR.adv_ne.p_PSS.y

S_Temperment_ON_I_Factor	0.00	0.02	0.981	0.00	tri_PCR.adv_ec.p_ProSoc.y
S_Temperment_ON_S_Factor	0.00	0.02	0.973	0.00	tri_PCR.adv_ec.p_ProSoc.y
S_Temperment_ON_Cohort	-0.04	0.03	0.152	-0.10	tri_PCR.adv_ec.p_ProSoc.y
I_Temperment_ON_I_Factor	0.03	0.02	0.273	0.05	tri_PCR.adv_ec.p_ProSoc.y
I_Temperment_ON_Cohort	0.13	0.03	0.000	0.20	tri_PCR.adv_ec.p_ProSoc.y
S_Temperment_ON_I_Factor	0.01	0.02	0.595	0.05	tri_PCR.adv_ne.p_ProSoc.y
S_Temperment_ON_S_Factor	-0.01	0.02	0.685	-0.04	tri_PCR.adv_ne.p_ProSoc.y
S_Temperment_ON_Cohort	0.02	0.02	0.370	0.06	tri_PCR.adv_ne.p_ProSoc.y
I_Temperment_ON_I_Factor	0.02	0.02	0.254	0.05	tri_PCR.adv_ne.p_ProSoc.y
I_Temperment_ON_Cohort	0.13	0.02	0.000	0.25	tri_PCR.adv_ne.p_ProSoc.y
S_Temperment_ON_I_Factor	-0.11	0.05	0.017	-0.18	tri_PCR.adv_ec.p_SEQ.y
S_Temperment_ON_S_Factor	-0.10	0.04	0.010	-0.20	tri_PCR.adv_ec.p_SEQ.y
S_Temperment_ON_Cohort	-0.03	0.03	0.259	-0.08	tri_PCR.adv_ec.p_SEQ.y
I_Temperment_ON_I_Factor	-0.10	0.04	0.018	-0.10	tri_PCR.adv_ec.p_SEQ.y
I_Temperment_ON_Cohort	0.12	0.03	0.000	0.20	tri_PCR.adv_ec.p_SEQ.y
S_Temperment_ON_I_Factor	-0.09	0.04	0.048	-0.16	tri_PCR.adv_ne.p_SEQ.y
S_Temperment_ON_S_Factor	-0.10	0.04	0.010	-0.20	tri_PCR.adv_ne.p_SEQ.y
S_Temperment_ON_Cohort	0.03	0.02	0.208	0.09	tri_PCR.adv_ne.p_SEQ.y
I_Temperment_ON_I_Factor	-0.08	0.04	0.038	-0.09	tri_PCR.adv_ne.p_SEQ.y
I_Temperment_ON_Cohort	0.13	0.02	0.000	0.25	tri_PCR.adv_ne.p_SEQ.y
S_Temperment_ON_I_Factor	0.17	0.07	0.013	0.31	tri_PPR.adv_ec.p_anx.y
S_Temperment_ON_S_Factor	-0.18	0.07	0.012	-0.39	tri_PPR.adv_ec.p_anx.y
S_Temperment_ON_Cohort	0.01	0.03	0.769	0.02	tri_PPR.adv_ec.p_anx.y
I_Temperment_ON_I_Factor	-0.37	0.07	0.000	-0.39	tri_PPR.adv_ec.p_anx.y
I_Temperment_ON_Cohort	0.06	0.03	0.039	0.10	tri_PPR.adv_ec.p_anx.y
S_Temperment_ON_I_Factor	0.01	0.06	0.818	0.03	tri_PPR.adv_ne.p_anx.y
S_Temperment_ON_S_Factor	-0.03	0.06	0.656	-0.06	tri_PPR.adv_ne.p_anx.y
S_Temperment_ON_Cohort	0.02	0.03	0.413	0.06	tri_PPR.adv_ne.p_anx.y
I_Temperment_ON_I_Factor	-0.34	0.07	0.000	-0.43	tri_PPR.adv_ne.p_anx.y
I_Temperment_ON_Cohort	0.07	0.03	0.004	0.14	tri_PPR.adv_ne.p_anx.y
S_Temperment_ON_I_Factor	0.05	0.03	0.082	0.17	tri_PPR.adv_ec.p_avoid.y
S_Temperment_ON_S_Factor	-0.08	0.03	0.025	-0.26	tri_PPR.adv_ec.p_avoid.y
S_Temperment_ON_Cohort	-0.04	0.02	0.113	-0.12	tri_PPR.adv_ec.p_avoid.y

I_Temperment_ON_I_Factor	-0.11	0.03	0.000	-0.20	tri_PPR.adv_ec.p_avoid.y
I_Temperment_ON_Cohort	0.11	0.03	0.000	0.18	tri_PPR.adv_ec.p_avoid.y
S_Temperment_ON_I_Factor	0.03	0.03	0.366	0.08	tri_PPR.adv_ne.p_avoid.y
S_Temperment_ON_S_Factor	-0.08	0.03	0.012	-0.27	tri_PPR.adv_ne.p_avoid.y
S_Temperment_ON_Cohort	0.01	0.02	0.651	0.03	tri_PPR.adv_ne.p_avoid.y
I_Temperment_ON_I_Factor	-0.07	0.03	0.010	-0.15	tri_PPR.adv_ne.p_avoid.y
I_Temperment_ON_Cohort	0.11	0.02	0.000	0.22	tri_PPR.adv_ne.p_avoid.y
S_Temperment_ON_I_Factor	-0.03	0.14	0.826	-0.02	tri_PPR.adv_ec.p_PSS.y
S_Temperment_ON_S_Factor	0.10	0.13	0.441	0.06	tri_PPR.adv_ec.p_PSS.y
S_Temperment_ON_Cohort	-0.03	0.02	0.234	-0.08	tri_PPR.adv_ec.p_PSS.y
I_Temperment_ON_I_Factor	0.46	0.15	0.003	0.14	tri_PPR.adv_ec.p_PSS.y
I_Temperment_ON_Cohort	0.09	0.03	0.001	0.15	tri_PPR.adv_ec.p_PSS.y
S_Temperment_ON_I_Factor	-0.26	0.13	0.055	-0.14	tri_PPR.adv_ne.p_PSS.y
S_Temperment_ON_S_Factor	0.19	0.12	0.126	0.11	tri_PPR.adv_ne.p_PSS.y
S_Temperment_ON_Cohort	0.01	0.02	0.559	0.04	tri_PPR.adv_ne.p_PSS.y
I_Temperment_ON_I_Factor	0.57	0.13	0.000	0.21	tri_PPR.adv_ne.p_PSS.y
I_Temperment_ON_Cohort	0.10	0.02	0.000	0.20	tri_PPR.adv_ne.p_PSS.y
S_Temperment_ON_I_Factor	0.00	0.02	0.924	0.01	tri_PPR.adv_ec.p_ProSoc.y
S_Temperment_ON_S_Factor	0.00	0.02	0.941	0.01	tri_PPR.adv_ec.p_ProSoc.y
S_Temperment_ON_Cohort	-0.03	0.02	0.239	-0.08	tri_PPR.adv_ec.p_ProSoc.y
I_Temperment_ON_I_Factor	0.03	0.02	0.164	0.07	tri_PPR.adv_ec.p_ProSoc.y
I_Temperment_ON_Cohort	0.08	0.03	0.001	0.14	tri_PPR.adv_ec.p_ProSoc.y
S_Temperment_ON_I_Factor	0.02	0.02	0.487	0.06	tri_PPR.adv_ne.p_ProSoc.y
S_Temperment_ON_S_Factor	0.00	0.02	0.852	-0.02	tri_PPR.adv_ne.p_ProSoc.y
S_Temperment_ON_Cohort	0.02	0.02	0.486	0.05	tri_PPR.adv_ne.p_ProSoc.y
I_Temperment_ON_I_Factor	0.03	0.02	0.167	0.07	tri_PPR.adv_ne.p_ProSoc.y
I_Temperment_ON_Cohort	0.10	0.02	0.000	0.18	tri_PPR.adv_ne.p_ProSoc.y
S_Temperment_ON_I_Factor	-0.09	0.04	0.048	-0.16	tri_PPR.adv_ec.p_SEQ.y
S_Temperment_ON_S_Factor	-0.10	0.04	0.009	-0.21	tri_PPR.adv_ec.p_SEQ.y
S_Temperment_ON_Cohort	-0.02	0.02	0.485	-0.05	tri_PPR.adv_ec.p_SEQ.y
I_Temperment_ON_I_Factor	-0.15	0.04	0.000	-0.15	tri_PPR.adv_ec.p_SEQ.y
I_Temperment_ON_Cohort	0.09	0.03	0.001	0.14	tri_PPR.adv_ec.p_SEQ.y
S_Temperment_ON_I_Factor	-0.10	0.04	0.024	-0.17	tri_PPR.adv_ne.p_SEQ.y

S_Temperment_ON_S_Factor	-0.11	0.04	0.004	-0.22	tri_PPR.adv_ne.p_SEQ.y
S_Temperment_ON_Cohort	0.03	0.02	0.211	0.09	tri_PPR.adv_ne.p_SEQ.y
I_Temperment_ON_I_Factor	-0.12	0.04	0.002	-0.14	tri_PPR.adv_ne.p_SEQ.y
I_Temperment_ON_Cohort	0.10	0.02	0.000	0.19	tri_PPR.adv_ne.p_SEQ.y
S_Temperment_ON_I_Factor	0.14	0.07	0.039	0.25	tri_Disc.adv_ec.p_anx.y
S_Temperment_ON_S_Factor	-0.17	0.07	0.017	-0.37	tri_Disc.adv_ec.p_anx.y
S_Temperment_ON_Cohort	0.00	0.04	0.951	-0.01	tri_Disc.adv_ec.p_anx.y
I_Temperment_ON_I_Factor	-0.37	0.07	0.000	-0.40	tri_Disc.adv_ec.p_anx.y
I_Temperment_ON_Cohort	0.06	0.04	0.120	0.09	tri_Disc.adv_ec.p_anx.y
S_Temperment_ON_I_Factor	-0.01	0.06	0.906	-0.01	tri_Disc.adv_ne.p_anx.y
S_Temperment_ON_S_Factor	-0.01	0.06	0.798	-0.03	tri_Disc.adv_ne.p_anx.y
S_Temperment_ON_Cohort	-0.03	0.04	0.519	-0.08	tri_Disc.adv_ne.p_anx.y
I_Temperment_ON_I_Factor	-0.34	0.06	0.000	-0.44	tri_Disc.adv_ne.p_anx.y
I_Temperment_ON_Cohort	0.10	0.03	0.005	0.18	tri_Disc.adv_ne.p_anx.y
S_Temperment_ON_I_Factor	0.04	0.03	0.126	0.14	tri_Disc.adv_ec.p_avoid.y
S_Temperment_ON_S_Factor	-0.08	0.03	0.018	-0.26	tri_Disc.adv_ec.p_avoid.y
S_Temperment_ON_Cohort	-0.05	0.04	0.229	-0.13	tri_Disc.adv_ec.p_avoid.y
I_Temperment_ON_I_Factor	-0.11	0.03	0.000	-0.21	tri_Disc.adv_ec.p_avoid.y
I_Temperment_ON_Cohort	0.12	0.04	0.001	0.19	tri_Disc.adv_ec.p_avoid.y
S_Temperment_ON_I_Factor	0.02	0.03	0.371	0.08	tri_Disc.adv_ne.p_avoid.y
S_Temperment_ON_S_Factor	-0.08	0.03	0.013	-0.26	tri_Disc.adv_ne.p_avoid.y
S_Temperment_ON_Cohort	-0.03	0.04	0.467	-0.08	tri_Disc.adv_ne.p_avoid.y
I_Temperment_ON_I_Factor	-0.07	0.03	0.005	-0.16	tri_Disc.adv_ne.p_avoid.y
I_Temperment_ON_Cohort	0.14	0.03	0.000	0.27	tri_Disc.adv_ne.p_avoid.y
S_Temperment_ON_I_Factor	0.02	0.14	0.873	0.01	tri_Disc.adv_ec.p_PSS.y
S_Temperment_ON_S_Factor	0.15	0.13	0.242	0.09	tri_Disc.adv_ec.p_PSS.y
S_Temperment_ON_Cohort	-0.04	0.04	0.325	-0.11	tri_Disc.adv_ec.p_PSS.y
I_Temperment_ON_I_Factor	0.49	0.15	0.001	0.15	tri_Disc.adv_ec.p_PSS.y
I_Temperment_ON_Cohort	0.10	0.04	0.008	0.15	tri_Disc.adv_ec.p_PSS.y
S_Temperment_ON_I_Factor	-0.25	0.13	0.062	-0.13	tri_Disc.adv_ne.p_PSS.y
S_Temperment_ON_S_Factor	0.19	0.12	0.113	0.11	tri_Disc.adv_ne.p_PSS.y
S_Temperment_ON_Cohort	-0.03	0.04	0.442	-0.08	tri_Disc.adv_ne.p_PSS.y
I_Temperment_ON_I_Factor	0.59	0.13	0.000	0.22	tri_Disc.adv_ne.p_PSS.y

I_Temperment_ON_Cohort	0.13	0.03	0.000	0.24	tri_Disc.adv_ne.p_PSS.y
S_Temperment_ON_I_Factor	0.00	0.02	0.937	-0.01	tri_Disc.adv_ec.p_ProSoc.y
S_Temperment_ON_S_Factor	0.00	0.02	0.921	-0.01	tri_Disc.adv_ec.p_ProSoc.y
S_Temperment_ON_Cohort	-0.04	0.04	0.302	-0.11	tri_Disc.adv_ec.p_ProSoc.y
I_Temperment_ON_I_Factor	0.03	0.02	0.171	0.07	tri_Disc.adv_ec.p_ProSoc.y
I_Temperment_ON_Cohort	0.09	0.04	0.016	0.14	tri_Disc.adv_ec.p_ProSoc.y
S_Temperment_ON_I_Factor	0.01	0.02	0.556	0.05	tri_Disc.adv_ne.p_ProSoc.y
S_Temperment_ON_S_Factor	-0.01	0.02	0.762	-0.02	tri_Disc.adv_ne.p_ProSoc.y
S_Temperment_ON_Cohort	-0.03	0.04	0.459	-0.08	tri_Disc.adv_ne.p_ProSoc.y
I_Temperment_ON_I_Factor	0.03	0.02	0.188	0.06	tri_Disc.adv_ne.p_ProSoc.y
I_Temperment_ON_Cohort	0.12	0.03	0.000	0.23	tri_Disc.adv_ne.p_ProSoc.y
S_Temperment_ON_I_Factor	-0.10	0.04	0.020	-0.18	tri_Disc.adv_ec.p_SEQ.y
S_Temperment_ON_S_Factor	-0.11	0.04	0.004	-0.22	tri_Disc.adv_ec.p_SEQ.y
S_Temperment_ON_Cohort	-0.03	0.04	0.492	-0.08	tri_Disc.adv_ec.p_SEQ.y
I_Temperment_ON_I_Factor	-0.16	0.04	0.000	-0.16	tri_Disc.adv_ec.p_SEQ.y
I_Temperment_ON_Cohort	0.08	0.04	0.018	0.14	tri_Disc.adv_ec.p_SEQ.y
S_Temperment_ON_I_Factor	-0.10	0.04	0.016	-0.18	tri_Disc.adv_ne.p_SEQ.y
S_Temperment_ON_S_Factor	-0.11	0.04	0.003	-0.22	tri_Disc.adv_ne.p_SEQ.y
S_Temperment_ON_Cohort	-0.02	0.04	0.691	-0.05	tri_Disc.adv_ne.p_SEQ.y
I_Temperment_ON_I_Factor	-0.12	0.04	0.001	-0.14	tri_Disc.adv_ne.p_SEQ.y
I_Temperment_ON_Cohort	0.12	0.03	0.000	0.23	tri_Disc.adv_ne.p_SEQ.y

Table S23a. Demographic differences between youth who grew or did not grow in youth-reported effortful control following adversity

Dependent Variable	*No Growth* *M*/%	*Growth* *M*/%	*t/z*	*df*	*p*	*d/h*
Child Age	12.21	12.55	-0.83	92.87	.411	-0.14
Child Grade	6.23	6.61	-0.87	86.32	.385	-0.16
Child Gender	0.59	0.51	0.89	1	0.375	0.15
Child Ethnicity	0.17	0.14	0.47	1	0.637	0.08
Child Race	0.32	0.22	1.18	1	0.237	0.21
Parent Marital Status	0.67	0.65	0.25	1	0.806	0.04
Parent Education	5.58	5.12	**2.08**	**93.01**	**.041**	**0.36**
Other Parent Education	4.81	5.04	-0.85	96.96	.397	-0.15
School Lunch (PR)	0.29	0.22	0.83	1	0.405	0.15
Food Stamps (PR)	0.18	0.10	1.30	1	0.193	0.24
Total Income (PR)	85,324.05	70,835.62	1.65	127.59	.102	0.26
Parent Gender	0.89	0.86	0.64	1	0.520	0.11
Parent Ethnicity	0.14	0.14	0.02	1	0.982	0.00
Parent Race	0.26	0.22	0.47	1	0.639	0.08

Table S23b. Demographic differences between youth who grew or did not grow in parent-reported effortful control following adversity

Dependent Variable	*No Growth* *M*/%	*Growth* *M*/%	*t/z*	*df*	*p*	*d/h*
Child Age	12.43	12.19	0.62	141.35	.538	0.10
Child Grade	6.39	6.31	0.20	138.24	.843	0.03
Child Gender	0.54	0.59	-0.58	1	0.560	-0.09
Child Ethnicity	0.18	0.15	0.49	1	0.625	0.08
Child Race	0.28	0.29	-0.16	1	0.873	-0.03
Parent Marital Status	0.72	0.60	1.50	1	0.135	0.24
Parent Education	5.40	5.47	-0.35	150.79	.729	-0.06
Other Parent Education	4.94	4.81	0.51	138.89	.611	0.08
School Lunch (PR)	0.29	0.24	0.82	1	0.414	0.13
Food Stamps (PR)	0.19	0.12	1.16	1	0.248	0.19
Total Income (PR)	88,288.80	71,198.88	1.85	142.99	.066	0.30
Parent Gender	0.87	0.90	-0.53	1	0.595	-0.09
Parent Ethnicity	0.15	0.13	0.36	1	0.718	0.06
Parent Race	0.25	0.25	-0.04	1	0.967	-0.01

Table S23c. Demographic differences between youth who grew or did not grow in youth-reported emotional stability following adversity

Dependent Variable	*No Growth M/%*	*Growth M/%*	*t/z*	*df*	*p*	*d/h*
Child Age	12.01	12.48	-1.12	96.48	.265	-0.20
Child Grade	6.06	6.50	-1.07	100.10	.289	-0.18
Child Gender	0.58	0.55	0.27	1	0.791	0.05
Child Ethnicity	0.10	0.20	-1.61	1	0.106	-0.29
Child Race	0.25	0.31	-0.74	1	0.461	-0.13
Parent Marital Status	0.63	0.68	-0.60	1	0.546	-0.10
Parent Education	5.67	5.31	1.70	103.04	.093	0.29
Other Parent Education	5.06	4.79	0.96	101.17	.337	0.17
School Lunch (PR)	0.33	0.24	1.18	1	0.237	0.20
Food Stamps (PR)	0.16	0.16	-0.02	1	0.980	0.00
Total Income (PR)	85,214.29	78,286.78	0.65	82.22	.517	0.12
Parent Gender	0.87	0.89	-0.45	1	0.656	-0.08
Parent Ethnicity	0.10	0.17	-1.20	1	0.228	-0.21
Parent Race	0.21	0.27	-0.76	1	0.449	-0.13

Table S23d. Demographic differences between youth who grew or did not grow in parent-reported emotional stability following adversity

Dependent Variable	*No Growth* *M*/%	*Growth* *M*/%	*t/z*	*df*	*p*	*d/h*
Child Age	11.70	12.73	**-2.69**	**129.80**	**.008**	**-0.44**
Child Grade	5.60	6.84	**-3.22**	**133.53**	**.002**	**-0.52**
Child Gender	0.60	0.54	0.76	1	0.448	0.13
Child Ethnicity	0.17	0.16	0.09	1	0.930	0.01
Child Race	0.30	0.28	0.27	1	0.785	0.05
Parent Marital Status	0.62	0.70	-1.05	1	0.292	-0.17
Parent Education	5.45	5.42	0.14	117.62	.887	0.02
Other Parent Education	4.68	5.01	-1.25	125.13	.214	-0.21
School Lunch (PR)	0.32	0.24	1.09	1	0.275	0.18
Food Stamps (PR)	0.20	0.13	1.15	1	0.250	0.19
Total Income (PR)	78,544.51	81,977.20	-0.35	114.92	.728	-0.06
Parent Gender	0.86	0.89	-0.52	1	0.602	-0.09
Parent Ethnicity	0.15	0.14	0.18	1	0.860	0.03
Parent Race	0.27	0.24	0.42	1	0.674	0.07

Table S24a. Demographic differences between youth who grew in youth-reported effortful control despite adversity or without adversity

Dependent Variable	*Growth Without Adversity M/%*	*Growth Despite Adversity M/%*	*t/z*	*df*	*p*	*d/h*
Child Age	11.37	12.55	**-3.11**	**71.34**	**.003**	**-0.48**
Child Grade	5.63	6.61	**-2.43**	**67.01**	**.018**	**-0.40**
Child Gender	0.56	0.51	0.68	1	0.494	0.11
Child Ethnicity	0.13	0.14	-0.30	1	0.765	-0.05
Child Race	0.32	0.22	1.29	1	0.195	0.21
Parent Marital Status	0.83	0.65	**2.90**	**1**	**0.004**	**0.42**
Parent Education	5.60	5.12	**2.42**	**68.64**	**.018**	**0.39**
Other Parent Education	5.31	5.04	1.11	68.59	.270	0.17
School Lunch (PR)	0.15	0.22	-1.31	1	0.192	-0.19
Food Stamps (PR)	0.04	0.10	-1.68	1	0.092	-0.23
Total Income (PR)	103,135.51	70,835.62	**3.85**	**139.68**	**< .001**	**0.41**
Parent Gender	0.92	0.86	1.47	1	0.142	0.21
Parent Ethnicity	0.10	0.14	-0.93	1	0.351	-0.14
Parent Race	0.26	0.22	0.57	1	0.566	0.09

Table S24b. Demographic differences between youth who grew in parent-reported effortful control despite adversity or without adversity

Dependent Variable	*Growth Without Adversity M/%*	*Growth Despite Adversity M/%*	*t/z*	*df*	*p*	*d/h*
Child Age	11.39	12.19	**-2.40**	**106.98**	**.018**	**-0.33**
Child Grade	5.70	6.31	-1.76	104.10	.081	-0.25
Child Gender	0.59	0.59	0.03	1	0.980	0.00
Child Ethnicity	0.15	0.15	0.07	1	0.942	0.01
Child Race	0.30	0.29	0.05	1	0.962	0.01
Parent Marital Status	0.79	0.60	**3.22**	**1**	**0.001**	**0.42**
Parent Education	5.68	5.47	1.27	123.20	.207	0.16
Other Parent Education	5.27	4.81	**2.03**	**106.12**	**.044**	**0.28**
School Lunch (PR)	0.17	0.24	-1.26	1	0.206	-0.17
Food Stamps (PR)	0.05	0.12	-1.84	1	0.066	-0.23
Total Income (PR)	102,168.24	71,198.88	**3.91**	**156.07**	**< .001**	**0.45**
Parent Gender	0.92	0.90	0.70	1	0.487	0.09
Parent Ethnicity	0.11	0.13	-0.63	1	0.527	-0.08
Parent Race	0.26	0.25	0.24	1	0.808	0.03

Table S24c. Demographic differences between youth who grew in youth-reported emotional stability despite adversity or without adversity

Dependent Variable	*Growth Without Adversity M/%*	*Growth Despite Adversity M/%*	*t/z*	*df*	*p*	*d/h*
Child Age	11.58	12.48	**-3.48**	**159.22**	**.001**	**-0.38**
Child Grade	5.85	6.50	**-2.45**	**154.14**	**.015**	**-0.27**
Child Gender	0.55	0.55	-0.05	1	0.959	-0.01
Child Ethnicity	0.12	0.20	**-2.02**	**1**	**0.044**	**-0.21**
Child Race	0.34	0.31	0.58	1	0.560	0.07
Parent Marital Status	0.81	0.68	**2.85**	**1**	**0.004**	**0.30**
Parent Education	5.70	5.31	**2.86**	**148.37**	**.005**	**0.33**
Other Parent Education	5.29	4.79	**2.77**	**155.59**	**.006**	**0.31**
School Lunch (PR)	0.15	0.24	**-2.13**	**1**	**0.033**	**-0.23**
Food Stamps (PR)	0.04	0.16	**-4.30**	**1**	**< .001**	**-0.41**
Total Income (PR)	107,261.56	78,286.78	**4.01**	**260.77**	**< .001**	**0.34**
Parent Gender	0.93	0.89	1.37	1	0.171	0.14
Parent Ethnicity	0.10	0.17	-1.89	1	0.058	-0.20
Parent Race	0.28	0.27	0.31	1	0.757	0.03

Table S24d. Demographic differences between youth who grew in parent-reported emotional stability despite adversity or without adversity

Dependent Variable	*Growth Without Adversity M/%*	*Growth Despite Adversity M/%*	*t/z*	*df*	*p*	*d/h*
Child Age	11.63	12.73	**-3.99**	**143.52**	**< .001**	**-0.47**
Child Grade	5.94	6.84	**-3.19**	**140.11**	**.002**	**-0.38**
Child Gender	0.55	0.54	0.25	1	0.799	0.03
Child Ethnicity	0.12	0.16	-0.93	1	0.353	-0.10
Child Race	0.32	0.28	0.75	1	0.453	0.09
Parent Marital Status	0.80	0.70	**2.12**	**1**	**0.034**	**0.24**
Parent Education	5.68	5.42	1.82	140.90	.071	0.22
Other Parent Education	5.19	5.01	0.95	144.11	.343	0.11
School Lunch (PR)	0.16	0.24	-1.69	1	0.092	-0.19
Food Stamps (PR)	0.04	0.13	**-3.30**	**1**	**0.001**	**-0.34**
Total Income (PR)	103,332.39	81,977.20	**2.84**	**203.07**	**.005**	**0.27**
Parent Gender	0.93	0.89	1.24	1	0.216	0.14
Parent Ethnicity	0.10	0.14	-1.12	1	0.265	-0.12
Parent Race	0.28	0.24	0.76	1	0.450	0.09

Table S25a. Regression of Univariate LGC Effortful Control and Emotional Stability Intercepts and Slopes on Gender (0 = Male, 1 = Female)

Parameter	*b*	SE	*p*	*β*
Youth-Report EC Intercept	0.06	0.04	0.132	0.07
Youth-Report EC Slope	0.03	0.05	0.588	0.03
**Parent-Report EC Intercept**	**0.20**	**0.04**	**0.000**	**0.19**
Parent-Report EC Slope	0.06	0.04	0.107	0.08
**Youth-Report ES Intercept**	**-0.12**	**0.04**	**0.003**	**-0.15**
Youth-Report ES Slope	-0.04	0.05	0.419	-0.05
Parent-Report ES Intercept	-0.04	0.04	0.326	-0.04
Parent-Report ES Slope	-0.01	0.04	0.733	-0.02

Table S25b. Regression of Bivariate LGC Effortful Control and Emotional Stability Intercepts and Slopes on Gender (0 = Male, 1 = Female)

Parameter	*b*	SE	*p*	*β*
Youth-Report EC Intercept	0.06	0.04	0.128	0.07
Youth-Report EC Slope	0.02	0.05	0.638	0.03
**Parent-Report EC Intercept**	**0.20**	**0.04**	**0.000**	**0.19**
Parent-Report EC Slope	0.06	0.04	0.124	0.08
**Youth-Report ES Intercept**	**-0.12**	**0.04**	**0.003**	**-0.15**
Youth-Report ES Slope	-0.04	0.05	0.380	-0.05
Parent-Report ES Intercept	-0.04	0.04	0.338	-0.04
Parent-Report ES Slope	-0.01	0.04	0.707	-0.02

Table S25c. Unstandardized Slopes for Univariate Latent Growth Curve Models of Youth and Parent-Reported Effortful Control and Emotional Stability Conditioned on Cohort and Gender

Construct	Parameter	*b*	*SE*	*p*
Effortful Control Youth-Report	Slope Intercept	0.11	0.05	0.022
	Slope Variance	0.15	0.04	0.000
Effortful Control Parent-Report	Slope Intercept	0.11	0.04	0.003
	Slope Variance	0.15	0.03	0.000
Emotional Stability Youth-Report	Slope Intercept	0.39	0.05	0.000
	Slope Variance	0.14	0.04	0.000
Emotional Stability Parent-Report	Slope Intercept	0.23	0.04	0.000
	Slope Variance	0.15	0.03	0.000

Table S25d. Correlated Change Estimates for Adversity, Effortful Control, and Emotional Stability Slopes for Bivariate Latent Growth Curve Conditioned on Cohort and Gender

Construct	*Unstandardized Covariance*	*SE*	*p*	*r*
Effortful Control Youth-Report	-0.05	0.01	0.000	-0.53
Effortful Control Parent-Report	-0.03	0.01	0.002	-0.33
Emotional Stability Youth-Report	-0.05	0.01	0.000	-0.54
Emotional Stability Parent-Report	-0.02	0.01	0.077	-0.17

Supplement C: Study Materials
For the manuscript "Growth Following Adversity is Rare"

Attachment Security to Caregiver

Fraley, R. C., Heffernan, M. E., Vicary, A. M., & Brumbaugh, C. C. (2011). The experiences in close relationships—Relationship Structures Questionnaire: A method for assessing attachment orientations across relationships. *Psychological Assessment*, *23*(3), 615–625. https://doi.org/10.1037/a0022898

1 = strongly disagree; 7 = strongly agree

Please answer the following 10 questions about your parent (the one who came with you):

1. It helps to turn to this person in times of need.
2. I usually discuss my problems and concerns with this person.
3. I talk things over with this person.
4. I find it easy to depend on this person.
5. I don't feel comfortable opening up to this person.
6. I prefer not to show this person how I feel deep down.
7. I often worry that this person doesn't really care for me.
8. I'm afraid that this person may abandon me.
9. I worry that this person won't care about me as much as I care about him or her.
10. I don't trust this person.

Selected Items from the Parenting Styles Scale

Lamborn, S. D., Mounts, N. S., Steinberg, L., & Dornbusch, S. M. (1991). Patterns of competence and adjustment among adolescents from authoritative, authoritarian, indulgent, and neglectful families. *Child Development*, *62*(5), 1049-1065.

What do you think is usually true or usually false about your (parent)?

1. **10. I can count on him/her to help me out if I have some kind of problem.**

Usually true = 1
Usually false = 0

2. **11. He/she keeps pushing me to do my best in whatever I do.**

Usually true = 1
Usually false = 0

3. **12. He/she keeps pushing me to think independently.**

Usually true = 1
Usually false = 0

4. **13. He/she helps me with my school work if there is something I don’t understand.**

Usually true = 1
Usually false = 0

5. **14. When he/she wants me to do something, he/she explains why.**

Usually true = 1
Usually false = 0

6. **15. When you get a poor grade in school, how often do your parents encourage you to try harder?**

Never = 0
Sometimes = 1
Usually = 1

7. **16. When you get a good grade in school, how often do your parents praise you?**

Never = 0
Sometimes = 1
Usually = 1

8. **17. How much do your parents really know who your friends are?**

Don’t know = 0
Know a Little = 1
Know a Lot = 1

How often do these things happen in your family?

9. **18. My parents spend time just talking with me.**

Almost every day = 1
A few times a week = 1
A few times a month = 1
Almost never = 0

10. **19. My family does something fun together.**

Almost every day = 1
A few times a week = 1
A few times a month = 1
Almost never = 0

Selected Items from the Revised Peer Experiences Questionnaire (RPEQ; De Los Reyes & Prinstein, 2004)

1. "Another kid helped me when I was having a problem"
2. "Another kid stuck up for me when was being picked on or excluded."

Never = 1
Once or twice = 2
A few times = 3
About once a week = 4
A few times a week = 5

Self-Esteem

Selected items from the Rosenberg Self-Esteem Scale.

Rosenberg, Morris. 1989. *Society and the Adolescent Self-Image*. Revised edition. Middletown, CT: Wesleyan University Press.

Indicate how much you agree with each of the following sentences.

1. ON THE WHOLE, I AM SATISFIED WITH MYSELF.

    A. STRONGLY AGREE
    B. AGREE
    C. DISAGREE
    D. STRONGLY DISAGREE

2. AT TIMES I THINK I AM NO GOOD AT ALL.

    A. STRONGLY AGREE
    B. AGREE
    C. DISAGREE
    D. STRONGLY DISAGREE

3. I FEEL THAT I HAVE A NUMBER OF GOOD QUALITIES.

    A. STRONGLY AGREE
    B. AGREE
    C. DISAGREE
    D. STRONGLY DISAGREE

4. I AM ABLE TO DO THINGS AS WELL AS MOST OTHER PEOPLE.

    A. STRONGLY AGREE
    B. AGREE

C. DISAGREE
D. STRONGLY DISAGREE

5. I FEEL I DO NOT HAVE MUCH TO BE PROUD OF.

A. STRONGLY AGREE
B. AGREE
C. DISAGREE
D. STRONGLY DISAGREE
