# Supplementary material for: Growth following adversity is rare: Evidence from a multi-informant longitudinal study of children and adolescents
Source: J Res Pers. Author manuscript; Available in PMC 2026 Jun 10. (PMC13249454; doi:10.1016/j.jrp.2025.104628)
Supplement: 4 [file NIHMS2177602-supplement-4.pdf]

# Supplement C: Study Materials
# For the manuscript "Growth Following Adversity is Rare"

Table of Contents

*Attachment Security to Caregiver* ........................................ *2*

*Selected Items from the Parenting Styles Scale* ........................................ *3*

*Selected Items from the Revised Peer Experiences Questionnaire (RPEQ; De Los Reyes & Prinstein, 2004)* ........................................ *5*

*Self-Esteem* ........................................ *6*

**Attachment Security to Caregiver**

Fraley, R. C., Heffernan, M. E., Vicary, A. M., & Brumbaugh, C. C. (2011). The experiences in close relationships—Relationship Structures Questionnaire: A method for assessing attachment orientations across relationships. *Psychological Assessment*, *23*(3), 615–625. https://doi.org/10.1037/a0022898

1 = strongly disagree; 7 = strongly agree

Please answer the following 10 questions about your parent (the one who came with you):

1. It helps to turn to this person in times of need.
2. I usually discuss my problems and concerns with this person.
3. I talk things over with this person.
4. I find it easy to depend on this person.
5. I don't feel comfortable opening up to this person.
6. I prefer not to show this person how I feel deep down.
7. I often worry that this person doesn't really care for me.
8. I'm afraid that this person may abandon me.
9. I worry that this person won't care about me as much as I care about him or her.
10. I don’t trust this person.

**Selected Items from the Parenting Styles Scale**

Lamborn, S. D., Mounts, N. S., Steinberg, L., & Dornbusch, S. M. (1991). Patterns of competence and adjustment among adolescents from authoritative, authoritarian, indulgent, and neglectful families. *Child Development*, *62*(5), 1049-1065.

*What do you think is usually true or usually false about your (parent)?*

1. **10. I can count on him/her to help me out if I have some kind of problem.**

Usually true = 1
Usually false = 0

2. **11. He/she keeps pushing me to do my best in whatever I do.**

Usually true = 1
Usually false = 0

3. **12. He/she keeps pushing me to think independently.**

Usually true = 1
Usually false = 0

4. **13. He/she helps me with my school work if there is something I don't understand.**

Usually true = 1
Usually false = 0

5. **14. When he/she wants me to do something, he/she explains why.**

Usually true = 1
Usually false = 0

6. **15. When you get a poor grade in school, how often do your parents encourage you to try harder?**

Never = 0
Sometimes = 1
Usually = 1

7. **16. When you get a good grade in school, how often do your parents praise you?**

Never = 0
Sometimes = 1
Usually = 1

8. **17. How much do your parents really know who your friends are?**

Don't know = 0
Know a Little = 1
Know a Lot = 1

*How often do these things happen in your family?*

9. **18. My parents spend time just talking with me.**

Almost every day = 1
A few times a week = 1
A few times a month = 1
Almost never = 0

10. **19. My family does something fun together.**

Almost every day = 1
A few times a week = 1
A few times a month = 1
Almost never = 0

**Selected Items from the Revised Peer Experiences Questionnaire (RPEQ; De Los Reyes & Prinstein, 2004)**

1. "Another kid helped me when I was having a problem"
2. "Another kid stuck up for me when was being picked on or excluded."

Never = 1
Once or twice = 2
A few times = 3
About once a week = 4
A few times a week = 5

**Self-Esteem**

Selected items from the Rosenberg Self-Esteem Scale.

Rosenberg, Morris. 1989. *Society and the Adolescent Self-Image*. Revised edition. Middletown, CT: Wesleyan University Press.

Indicate how much you agree with each of the following sentences.

1. ON THE WHOLE, I AM SATISFIED WITH MYSELF.

   A. STRONGLY AGREE
   B. AGREE
   C. DISAGREE
   D. STRONGLY DISAGREE

2. AT TIMES I THINK I AM NO GOOD AT ALL.

   A. STRONGLY AGREE
   B. AGREE
   C. DISAGREE
   D. STRONGLY DISAGREE

3. I FEEL THAT I HAVE A NUMBER OF GOOD QUALITIES.

   A. STRONGLY AGREE
   B. AGREE
   C. DISAGREE
   D. STRONGLY DISAGREE

4. I AM ABLE TO DO THINGS AS WELL AS MOST OTHER PEOPLE.

   A. STRONGLY AGREE
   B. AGREE
   C. DISAGREE
   D. STRONGLY DISAGREE

5. I FEEL I DO NOT HAVE MUCH TO BE PROUD OF.

   A. STRONGLY AGREE
   B. AGREE
   C. DISAGREE
   D. STRONGLY DISAGREE
